# Dimerization of confined Brønsted acids in enantioselective organocatalytic reactions†

**DOI:** 10.1039/d3sc03769j

**Published:** 2023-09-18

**Authors:** Ingolf Harden, Frank Neese, Giovanni Bistoni

**Affiliations:** a Max-Planck-Institut für Kohlenforschung Kaiser-Wilhelm Platz 1 45470 Mülheim an der Ruhr Germany; b Department of Chemistry, Biology and Biotechnology, University of Perugia Via Elce di Sotto, 8 06123 Perugia Italy giovanni.bistoni@unipg.it

## Abstract

The formation of Brønsted acid aggregates in the course of asymmetric organocatalytic reactions is often overlooked in mechanistic studies, even though it might have a deep impact on the stereo-controlling factors of the transformations. In this work, we shed light on the influence of the catalyst structure and reaction conditions on the spontaneity of the aggregation process for popular chiral organocatalysts derived from phosphoric acids using high-level quantum mechanical calculations. Our study encompasses small and sterically unhindered chiral phosphoric acids as well as large and “confined” imidodiphosphates and imidodiphosphorimidates. These systems have recently proven particularly effective in promoting a large number of highly relevant asymmetric transformations. While cooperative catalytic effects of sterically less hindered chiral phosphoric acid catalysts are well appreciated in literature, it is found that the formation of catalyst dimers in solution is possible for both standard and confined catalysts. The spontaneity of the aggregation process depends on reaction conditions like solvent polarity, polarizability, temperature, the nature of the interaction with the substrate, as well as the catalyst architecture. Finally, it is shown that, at low temperatures (153 K), the aggregation process can profoundly influence the reaction kinetics and selectivity.

## Abstract 2

We shed light on the influence of the catalyst structure and reaction conditions on the spontaneity of the dimerization process for popular chiral organocatalysts derived from phosphoric acids using high-level quantum mechanical calculations.

## Graphical abstract

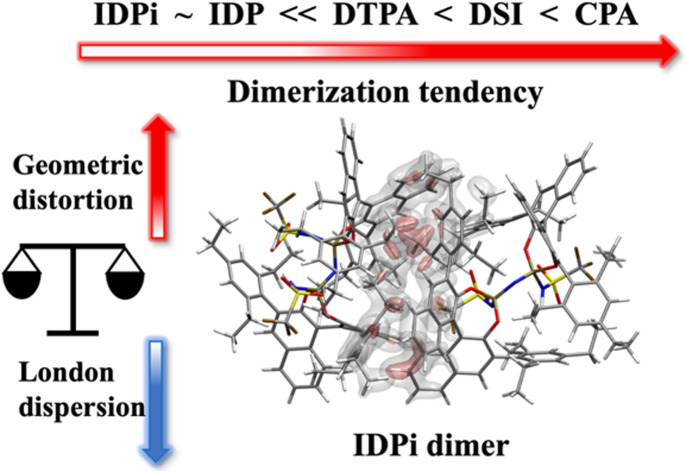

## Introduction

Chiral Brønsted acids derived from BINOL are commonly used as organocatalysts to promote a broad range of highly selective reactions involved in the synthesis of many valuable chemical entities, such as pharmaceuticals and agrochemicals.^1^

They can be grouped into families with different structure and properties, such as (i) chiral phosphoric acids^2–6^ (CPA), (ii) dithiophosphoric acids^7–9^ (DTPA), (iii) disulfonimides^10,11^ (DSI); (iv) imidodiphosphates^12–14^ (IDP) and (v) imidodiphosphorimidates^15,16^ (IDPi). In particular, IDPi recently demonstrated remarkable activity and selectivity toward a large number of highly relevant stereoselective transformations,^17–23^ thanks to their confined active site, high acidity and modular architecture, which offers ample opportunities for catalyst design.

Mechanistic insights are instrumental for catalyst optimization and typically require synergistic experimental and computational efforts.^23–31^ Of paramount importance in this context is understanding the influence of the catalyst structure on the selectivity of the transformation and the role played by noncovalent interactions and steric effects, which might be capable of favoring/disfavoring only specific reaction pathways.^32–34^

Among the computational studies that addressed this problem, our group^35^ examined Diels–Alder reactions between cinnamate esters and cyclopentadiene and found that the stereoselectivity of the IDPi catalyst is governed by the balance of repulsive short-range steric effects and attractive long-range dispersion interactions. Wheeler and coworkers^36^ investigated the stereoselective synthesis of 2,3-dihydroquinazolinones catalyzed by chiral SPINOL-phosphoric acids and found that the stereoselectivity is influenced by π-stacking interactions between the substrate and the aryl ligands of the catalyst, as well as by hydrogen bonds between the substrate and the catalyst's core. The Houk group found^37^ stereodivergence for the CPA catalyzed allylation of aldehydes with β-akenyl allylic boronates for different substrates that was explained by noncovalent C–H–π interactions. Recently, we proposed a dispersion-driven induced fit model for the stereoselective intramolecular etherification of 4-phenylpent-4-en-1-ol, in which both catalyst distortion and the catalyst–substrate interaction are mainly governed by London dispersion.^38^

All previously discussed reactions assumed a monoacidic pathway, meaning that only one catalyst molecule is involved in the rate- and/or stereo-determining step. However, it is important to note that sterically unhindered phosphoric acids tend to spontaneously aggregate in solution. This aggregation can have a significant impact on the intricate network of covalent and noncovalent interactions that govern the essential properties of similar transformations. For example, Malm *et al.*^39^ demonstrated that multimers of diphenyl phosphoric acid with quinaldine (Qu) are formed in solution of aprotic solvents *via* NMR spectroscopy, while Detering and coworkers^40^ identified phosphoric acid trimers in CDF_3_ + CDF_2_Cl (1 : 3) solutions. Gschwind and coworkers presented evidence for the formation of CPA/imine aggregates in which imine molecules are nested between two CPAs.^41,42^

When these aggregation processes occur, one possible consequence is a change in the electronic properties of the active site, which can have a notable impact on the catalyst's activity. For example, the groups of List and Thiel^43^ observed that heterodimerization of CPAs with small carboxylic acids increased the CPA acidity, resulting in increased reaction rates for epoxide ring opening reactions.

Moreover, cooperative effects between multiple catalyst molecules can also profoundly influence the selectivity of a chemical transformation. Niemeyer and coworkers^44^ investigated acid–acid interactions in catenated and catalytically active CPA dimers and found inversed stereoselectivities compared to the monoacidic pathway for the transfer-hydrogenation of 2-phenylquinoline. For the non-catenated CPAs,^45^ the monomeric and dimeric pathways compete, with the latter showing increased stereoselectivity. Toste and coworkers^46^ investigated the stereoselective CPA-catalyzed fluorination of allylic alcohols with Selectfluor and found reversal of stereoselectivity for catalysts with aryl substituents of increasing size. This effect was explained with dimerization processes taking place for the less sterically demanding catalysts. Recently, the groups of Yoon and Toste^47^ proposed a mechanism for stereoselective [2 + 2] photocycloadditions of vinylpyridines with styrene in which cooperative stereoinduction of the Ir-containing photocatalyst and the CPA catalyst determines the stereoselective outcome. Recently, Gschwind and coworkers investigated the key factors leading to the separation between the monomeric and dimeric pathways for the CPA-catalyzed transfer hydrogenation of imines. They found that high catalyst loadings, low temperatures and high absolute concentrations favor the dimeric pathway, leading to inversed stereoselectivity.^48^

**Scheme 1:**
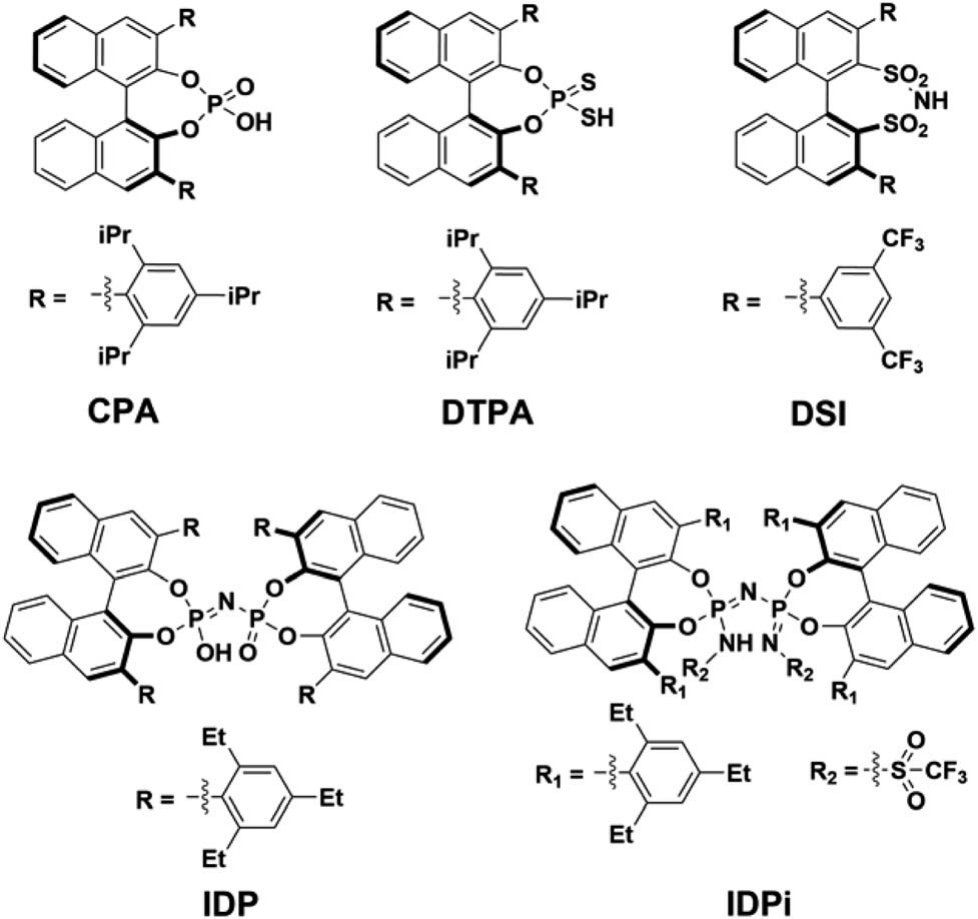
Lewis structures of acids considered in this work. The identifiers of the structures in the further text are given below each Lewis structure and refer to the class name of the respective acid. CPA = chiral phosphoric acid, DTPA = dithiophosphoric acid, DSI = disulfonimide, IDP = imidodiphosphate, IDPi = imidodiphosphorimidate.

In contrast, for “confined” catalysts like IDP or IDPi, dimerization in aprotic solvents has been largely overlooked, as well as its influence on the catalytic activity in the realm of counter-anion directed catalysis^49,50^ (ACDC). The aim of this work is bridging this knowledge gap by investigating the acid dimerization process in solution using state-of-the-art computational techniques for representative BINOL-derived acids (Scheme 1). In addition, the impact of a possible Brønsted acid dimerization on the activity of an asymmetric transformation is also examined in detail using the recently^51^ developed IDPi catalyzed stereoselective C–C coupling reaction of silyl nitronates with silyl ketene acetals as a case study.

## Methodology

Unless otherwise specified, all calculations were carried out with the ORCA^52,53^ program package version 5.0.3.^54^

For studying the catalyst aggregation process, an initial structural ensemble (SE) encompassing a large number of monomers and dimers for the catalysts shown in Scheme 1 was generated using the Conformer–Rotamer Ensemble Sampling Tool^55–57^ (CREST) at the GFN-FF^58^ level of theory, as implemented in the Extended Tight-Binding Program Package^59^ (xTB). For CPA, DTPA and DSI, all unique conformers in the SE were optimized with the Perdew–Burke–Ernzerhof^60^ (PBE) functional in combination with the Ahlrichs double-zeta def2-SVP^61^ basis set. Grimme's D3 dispersion correction^62^ was invoked together with Becke–Johnson damping.^63^ The resolution of identity^64^ was used in the split-RJ^65^ version together with the appropriate Coulomb-fitting auxiliary basis set^66^ to speed up the calculations. This protocol is abbreviated as PBE-D3/def2-SVP. For the larger catalytic systems (IDP and IDPi), optimization of all found dimer conformers was computationally too demanding, and hence only the lowest-in-energy conformers (in a range of 2.8 kcal mol^−1^) were optimized. Some of the conformers with higher energy were also included in the optimization step, *i.e.*, those with unique and significant structural features. Importantly, for IDP and IDPi, different starting structures were used for the conformer sampling procedure to better explore the chemical space. For the most stable conformer identified, frequency calculations were carried out to compute thermal corrections. Final single point energies were obtained using the range-separated ωB97M-V^67,68^ functional together with the def2-TZVP basis set and the RIJCOSX^69,70^ approximation. Solvation corrections were computed at the B3LYP-D3/def2-TZVP^60,71–73^ level of theory using the SMD model (toluene).^74^ The final Gibbs free energy *G* was obtained *via*

1 *G* = *E*^ωB97M-V^_SP_ + *G*^B3LYP^_solv_ + *G*^PBE^_thermo_.

The dimerization energy of each catalyst (Δ*G*_dim_) was computed using a supramolecular approach, by subtracting from the energy of the most stable dimer the energy of the catalysts in their geometric ground state. Importantly, the key findings discussed in this work (*e.g.*, the sign of Δ*G*_dim_ indicating the spontaneity of the process under the experimental conditions) are essentially independent by the nature of the specific exchange correlation functional employed and/or by the level of theory used for incorporating solvation effects, as detailed in the ESI (see Tables S1 and S4†).

For studying the IDPi-catalyzed stereoselective C–C coupling reaction of silyl nitronates, a different protocol was used due to the increased system size. In particular, the initial GFN-FF/CREST conformational sampling on the ion pair/transition state involving two catalysts was performed by freezing the one-catalyst ion pair/transition state to its geometric ground state. Geometry optimizations of the most stable one- and two-catalyst ion pair conformers and transition states were performed at the r^2^SCAN-3c/GFN2-xTB level of theory using an multiscale ONIOM^75^ approach, as implemented in ORCA 5.0.3. As a note of caution, it is worth emphasizing here that complete exploration of the conformational space for these challenging systems with state-of-the-art computational protocols is not possible due to the challenging system size. Hence, the conformer sampling was used to identify the optimal position of a second catalyst molecule, keeping all other atoms frozen to the same position they have in the corresponding structures along the monomeric pathway. In all cases, the catalyst molecules were treated at the GFN2-xTB level of theory, while the silyl nitronate and the nucleophile were treated at the DFT level. Solvation effects were treated with the implicit ALPB solvation model.^76^ Numerical frequencies were obtained at the same level of theory. The reactant and product states were obtained from intrinsic reaction coordinate^77^ (IRC) calculations and subsequent geometry optimizations. Importantly, optimizing the entire systems at the r^2^SCAN-3c level does not change the associated electronic energy variations, which demonstrate that our results on the aggregation process are robust and to a large extent independent by the chosen level of electronic structure theory (see ESI, Section S10†). Final single point energies including Grimme's geometrical counterpoise correction^78^ (gCP) were obtained at the ωB97M-V/def2-TZVP/SMD(CH_2_Cl_2_) level of theory.

## Results and discussion

### Spontaneous and non-spontaneous dimerization processes in solution

**Fig. 1:**
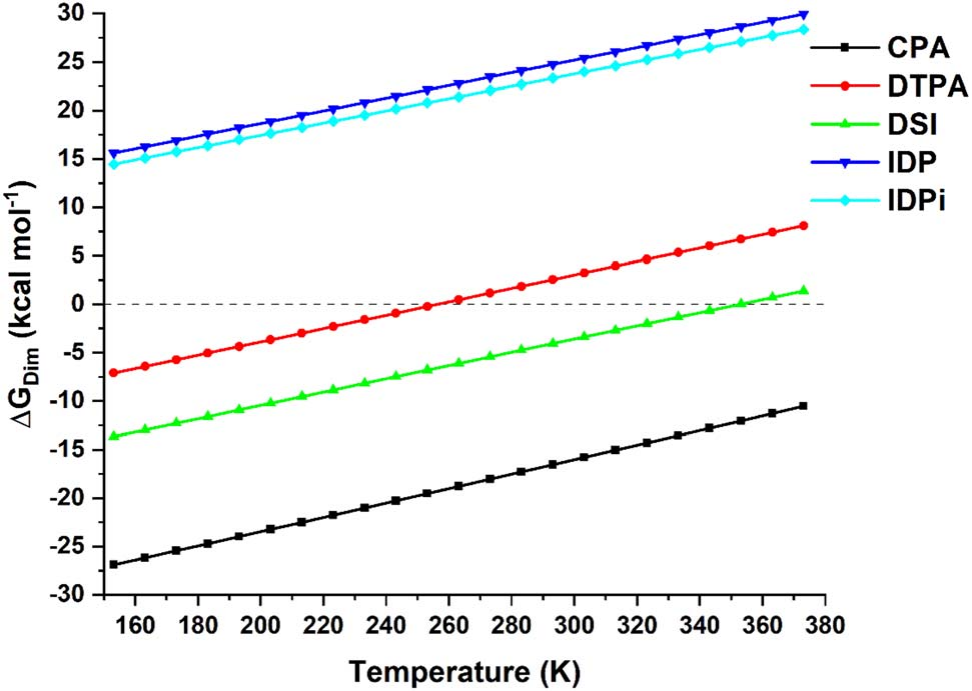
Temperature dependence of the dimerization free energy in toluene for the catalysts studied in this work. Note that the boiling point of toluene is *ca.* 384 K.

The temperature dependence of the computed dimerization free energy Δ*G*_dim_ in a commonly used aprotic solvent in organocatalysis (toluene in the present example) for the catalysts shown in Scheme 1 is illustrated in Fig. 1; as expected for a bimolecular process, the entropic term is largely responsible for the temperature dependence of the dimerization free energy.

Importantly, CPA shows negative Δ*G*_dim_ at all temperatures and it is thus expected to form stable dimers in solution. In contrast, DSI and DTPA feature a much larger (less negative) Δ*G*_dim_ than CPA. While DTPA and DSI are still expected to form stable dimers under mild reactions conditions, our calculations suggest that dimerization should not be favorable at higher temperatures. In particular, Δ*G*_dim_ = 0 for *T* = 256 K for DTPA and for *T* = 354 K for DSI (note that a computational error of 2 kcal mol^−1^ in the dimerization energy estimate would lead to variations of these temperatures of about +/− 30 K). The confined catalysts IDP and IDPi show a completely different behavior, with positive free dimerization energies at all temperatures. Hence, these catalysts are not expected to dimerize under experimental conditions.

It is important to emphasize here that whether or not catalyst aggregation plays a significant role in a chemical process, *e.g.*, by influencing its selectivity, also depends on other factors like the size of the substituents, solvent polarity, reaction kinetics and catalyst concentration, as it has been suggested in literature^45^ for a CPA catalyzed transformation. For example, Zhang *et al.*^79,80^ performed kinetic studies for the disulfonimide catalyzed cyanosilylation of Napthaldehyde at room temperature and with diethyl ether solvent and found an average reaction order for the catalyst of 1.23, resembling a first order influence of the catalyst on the rate constant. Moreover, Gschwind and coworkers did not find evidence supporting the formation of DSI-imine aggregates containing more than one DSI molecule in CD_2_Cl_2_ solution at 180 K, while they were able to detect the formation of CPA-imine ion pair dimers *via* NMR spectroscopy.^81^

However, aside from effects associated with specific reaction conditions, our results demonstrate that dimer stability follows the trend:

2 CPA > DSI > DTPA ≫ IDP ∼ IDPi

which is also expected to correlate with the probability of observing cooperativity effects in reactions catalyzed by these systems assuming similar experimental variables. A thorough analysis of the key acid–acid and acid-solvent noncovalent interactions responsible for these trends is provided in the following sections.

### Effect of solute–solvent interactions on the dimerization process

The solvation contribution to Δ*G*_dim_ comprises of electrostatic and non-electrostatic components, both of which were modeled using the SMD scheme.^82^ In the present case, both effects favor dimer dissociation, as shown in the ESI (see Tables S4 and S6†). On the one hand, electrostatic solute–solvent interactions strongly stabilize the non-interacting monomers. Thus, solvent polarity is one of the factors that can be used to experimentally control the spontaneity of the catalyst aggregation process, especially for catalysts like DTPA and DSI with relatively small dimerization free energies. On the other hand, solute–solvent dispersion interactions increase with increasing solvent accessible surface (SAS), and hence they also preferentially stabilize the monomers. Importantly, changing the shape and the polarizability of the solvent could have an impact on the strength of the solute–solvent dispersion interaction, thus affecting the energetics and hence the spontaneity of the aggregation process.^82^ Hence, for systems like CPA, DTPA and DSI, effects like temperature, solvent polarity and polarizability can tip the scale on whether catalyst dimers are formed in solution or not. In contrast, for the larger IDP and IDPi catalysts, the formation of supramolecular aggregates is unlikely under standard experimental conditions. As discussed in the ESI (see Section S6†), the solvation contribution to the free dimerization energy is fairly independent of the density functional. To understand the origin of these differences, a detailed analysis of the key intermolecular interactions in these dimers is carried out in the next section.

### Effect of intermolecular catalyst–catalyst interactions on the dimerization process

**Fig. 2:**
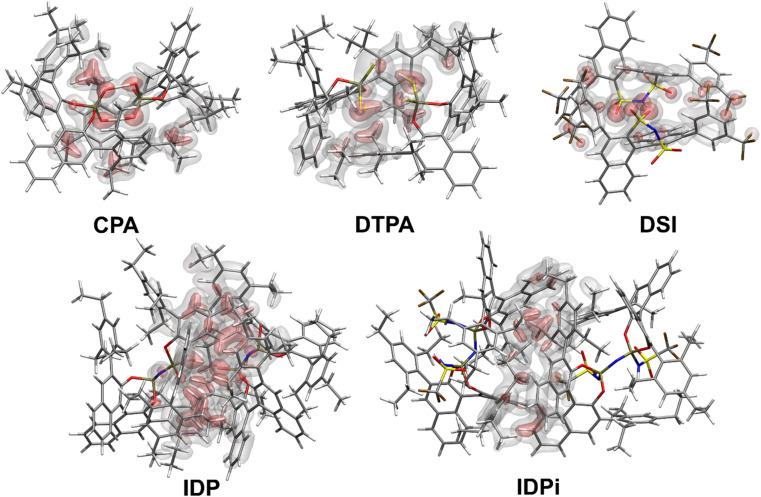
Structures and spatial analysis of dispersion forces for the most stable Brønsted acids dimers considered in this work. The dispersion interaction density (DID) plots were computed at the HFLD/Silver level. Density cutoffs: red surface 0.05 kcal mol^−1^ bohr^−3^, grey surface 0.01 kcal mol^−1^ bohr^−3^.

Fig. 2 shows the most stable conformers found from the sampling procedure for all the catalysts studied in this work. The corresponding dispersion interaction density (DID)^83,84^ plots at the HFLD/Silver level^85^ are also shown and provide a spatial analysis of the role played by London dispersion forces acting between the monomers.

Structural analysis revealed the presence of two intermolecular hydrogen bonds in the CPA and DTPA dimers, while DSI and IDP feature only one hydrogen bond in their respective dimers. The most stable IDPi dimer does not show hydrogen bonds. While the difference between Δ*G*_dim_ for CPA and DSI (Fig. 1) can be largely attributed to the relative strength of the corresponding O–H hydrogen (CPA) and S–H (DTPA) hydrogen bonds, understanding the relative stability of the other aggregates considered in this work is less straightforward and requires a deeper computational analysis.

**Fig. 3:**
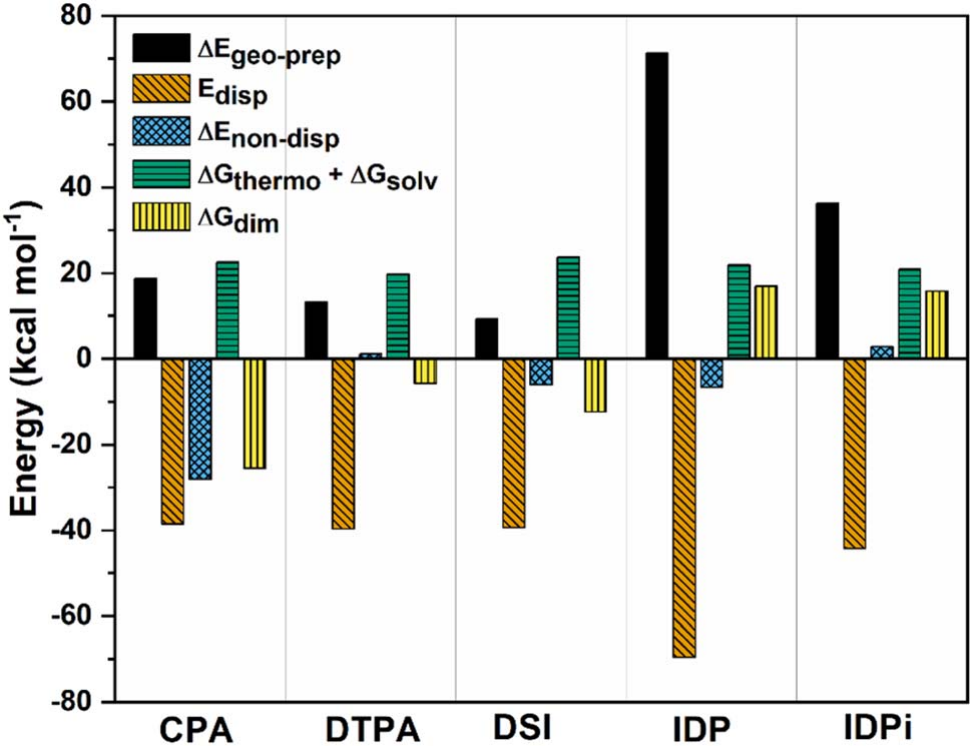
Decomposition of Δ*G*_dim_ into various contributions at the ωB97M-V/def2-TZVP level of theory at 173 K for CPA, DTPA, DSI, IDP and IDPi. All energies are in kcal mol^−1^.

To achieve this goal, Δ*G*_dim_ at 173 K was decomposed into various terms:

3 Δ*G*_dim_ = Δ*E*_geo-prep_ + *E*_disp_ + Δ*E*_non-disp_ + Δ*G*_thermo_ + Δ*G*_solv_

where Δ*E*_geo-prep_ is the geometric preparation energy, which is the energy needed in order to distort the monomers' geometries from their equilibrium structure to the one they have in the dimer, and hence quantifies the geometrical “strain” of the monomers in the dimer; *E*_disp_ is the monomer–monomer intermolecular dispersion energy stabilizing the dimer; Δ*E*_non-disp_ is the non-dispersive interaction between the monomers, including for example hydrogen bond (stabilizing) and charge penetration (destabilizing) effects; Δ*G*_thermo_ and Δ*G*_solv_ are correction terms that account for thermal effects (including entropy, which favors the dissociation) and solvation, respectively. The decomposition of Δ*G*_dim_ for the systems considered in this work can be found in Fig. 3.

Δ*G*_thermo_and Δ*G*_solv_ are roughly constant along the series and, as already discussed, favor dimer dissociation. The other repulsive contribution to the interaction is the geometric preparation associated with the catalysts' strain in the adduct, which to a large extent increases with the size of the interacting monomers. For small catalysts (CPA, DTPA, and DSI) the geometric preparation is comparable to the contribution from entropy and solvation, while its importance increases significantly for IDP and IDPi.

For CPA, the non-dispersive interaction Δ*E*_non-disp_ is large and negative. It amounts to −28.1 kcal mol^−1^ and can be attributed to two strong intermolecular O–H hydrogen bonds that stabilize the dimeric structure. In DTPA, on the other hand, the S–H hydrogen bonds are much weaker. Accordingly, Δ*E*_non-disp_ is slightly positive in this case due to balance of attractive (weak hydrogen bonds, electrostatic) and repulsive (charge penetration, steric repulsion) interactions. For all other dimers, Δ*E*_non-disp_ vanishes, making London dispersion *E*_disp_ the dominant energy component stabilizing the dimer.

Irrespective of the level of theory used (see Fig. S2†), CPA, DTPA, DSI and IDPi show qualitatively similar *E*_disp_ energies, even though IDPi is much larger, and hence more polarizable, than the other catalysts considered in this work. This seemingly counterintuitive effect can be understood by analyzing the DID plots shown in Fig. 2. As illustrated by the red (inner) surfaces in this figure, the strongest dispersive interactions in the dimers typically involve the catalysts' core. However, for IDPi, the catalysts' core does not contribute significantly to the interaction, probably due to steric effects originating from the substituents at the nitrogen position (R_2_). Such bulky groups are not present in the IDP core, and hence the corresponding dimer shows an intermolecular hydrogen bond and a very large *E*_disp_ value. However, the large value of *E*_disp_ in IDP is entirely compensated by the corresponding increase in Δ*E*_geo-prep_, *i.e.*, catalyst distortion increases to allow for an optimal interaction between the monomers. For these reasons, IDP and IDPi show comparable dimerization energies. It is worth mentioning here that the geometric preparation of bulky catalytic systems like IDP and IDPi is determined to a large extent by the strength of intra-catalyst dispersive interactions.^38^ For example, in the case of IDP, the active site is shielded by the ligands in the ground state geometric structure. Hence, significant structural rearrangement is required to allow the direct interaction between the active centers in the dimer. Similarly, for IDPi, the orientation of the 3,3′ aryl ligands is perturbed upon the formation of the dimer. Such structural rearrangements occur at the expense of intra-catalyst dispersion interactions, resulting in increased energy.

Taken together, these results suggest that the relative stability of the smaller CPA, DSI and DTPA Brønsted acid dimers is determined by the nature and number of hydrogen bonds involving the XH groups (X = S/O) in the catalysts' core. In the IDP and IDPi dimers, steric hindrance prohibits the formation of strong hydrogen bonds. In addition, intermolecular dispersion forces are fully counteracted by the repulsive catalyst strain. For these reasons, the dimers of these catalysts are unstable in solution. While different substituents might influence the magnitude of Δ*G*_dim_, we do not anticipate significant variations in these trends for the standard substituents commonly used in organocatalysis.

### Dimerization processes under catalytic conditions

**Scheme 2:**
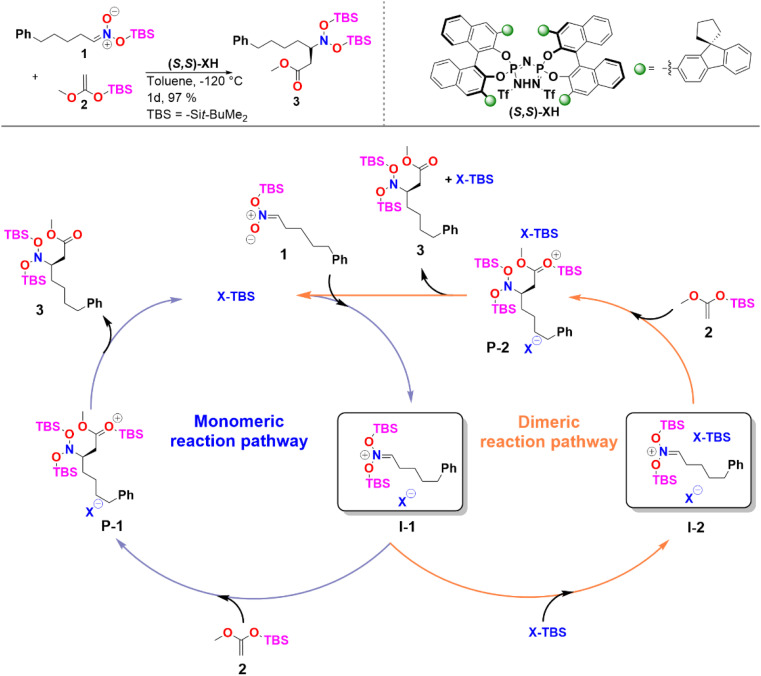
Reaction considered in this work and proposed catalytic cycle. See ref. 51 for experimental details.

In the case of sterically unhindered phosphoric acids, the influence of catalyst dimerization on the mechanism and selectivity of organocatalytic transformations has already been extensively studied.^45,48^ In contrast, for asymmetric reactions catalyzed by bulky systems like IDPi, the role played by catalyst dimerization is not yet fully understood. To help fill this gap, we selected as a case study, the C–C bond formation of silyl nitronates with silyl ketene acetals catalyzed by IDPi.^51^ The proposed mechanism for the reaction is outlined in Scheme 2.

**Fig. 4:**
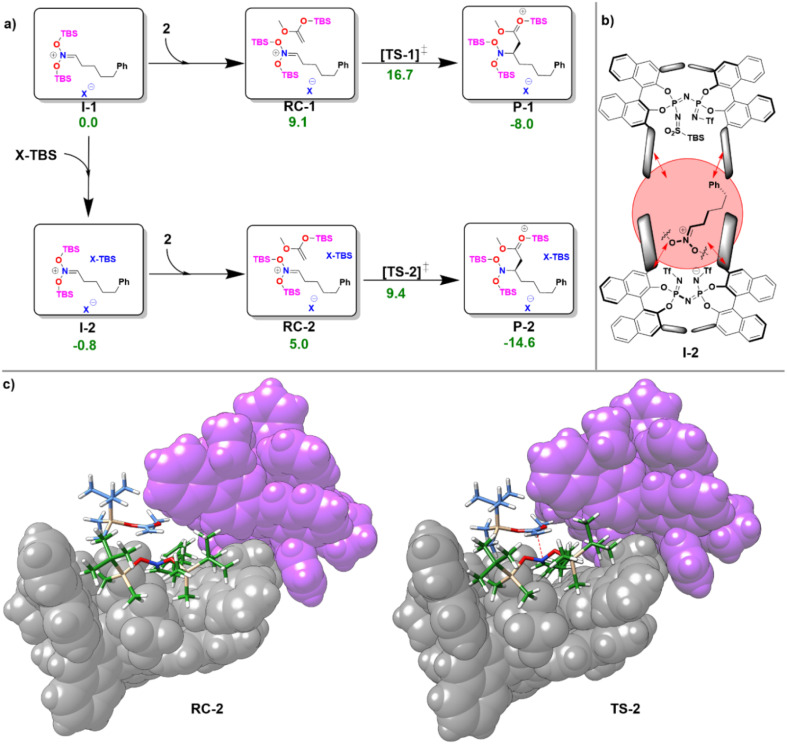
(a) Computed free energy profile for the reaction of the monomeric (I-1) and dimeric (I-2) ion pair with the nucleophile 2 at 153 K. All free energies are relative to that of I-1. All energies are in kcal mol^−1^. (b) Lewis structure of I-2. (c) 3D structures of RC-2 and TS-2. For both structures, the atoms of the X-TBS molecule are shown in purple.

In the first step of the monomeric reaction pathway, the catalyst XH is silylated at room temperature to form X-TBS, which is the catalytically active species entering the catalytic cycle. The key step of the reaction is the formation of the chiral ion pair (I-1), which consists of the catalyst anion X^−^ and the doubly silylated nitronate cation. I-1 is attacked by the nucleophile 2, which leads to the product P-1 in a single step. After release of X-TBS from P-1, the final product 3 is formed. In the dimeric reaction pathway, a second X-TBS molecule enters the catalytic cycle after the formation of I-1, leading to the formation of a second ion pair involving two catalyst molecules I-2. The computed monomeric and dimeric reaction profiles at 153 K, which is the experimental temperature for the C–C coupling reaction,^51^ are shown in Fig. 4 together with the 3D structures of the dimeric reactant and transition state.

Remarkably enough, the aggregation of I-1 with X-TBS to give I-2 features a free reaction energy (Δ*G*_R_) close to zero (−0.8 kcal mol^−1^). The small value of Δ*G*_R_ suggests that the spontaneity of this process might change for different substituents and/or for different reaction conditions. Experimentally, the NMR characterization of the ion pair containing *tert*-Butyldimethylsilyl (*E*)-(2-phenylethylidene)azinate was carried out at 193 K and no catalyst dimerization was observed.^51^ Stimulated by these findings, we re-computed Δ*G*_R_ at 193 K and found a positive value (1.9 kcal mol^−1^), which is consistent with the experimental results. These results provide further evidence for the fundamental role played by temperature in this context. In addition, it should be noted that the use of different substituents at the silyl nitronate could also influence the geometric match between the monomers, thus affecting the energy of the dimerization process.

Analysis of the noncovalent interactions between the three main fragments in I-2 (X^−^, bis-silyl nitronate, X-TBS) at the HFLD/Silver level revealed that electrostatic forces dominate the X^−^–nitronate interaction, while dispersive interactions are the dominant contribution to the interaction between the nitronate and X-TBS (see ESI, Table S8†). Importantly, the geometric preparation of the interacting fragments in I-2 (computed with respect to their geometries in I-1 or, for X-TBS, with respect to its equilibrium geometry) was found to be relatively small (−1.2 kcal mol^−1^ for X^−^, 8.8 kcal mol^−1^ for the nitronate, 1.2 kcal mol^−1^ for X-TBS at the ωB97M-V level of theory), indicating that strong interactions between the fragments can be realized without large structural changes (see Fig. 4b). This behavior is in striking contrast to our findings for the IDP and IDPi dimers (Fig. 3), which also feature strong dispersive interactions which are, however, counteracted by geometric preparation, thus making dimerization of these systems unlikely.

It can be concluded that the formation of aggregates of confined phosphoric acid derivatives (X^−^ and X-TBS in the present example) under reaction conditions might be favored by the presence of substrates (the positively charged nitronate in this case) and/or reaction additives that sit between the two catalysts and act as a “glue” between them through electrostatic and dispersion forces.

As can be seen from Fig. 4c, for the dimeric complexes including the nucleophile (RC-2 and TS-2) the dual catalyst pocket opens up in order to facilitate interaction between the nucleophile and the nitronate. The dimeric transition state (TS-2) features a smaller relative free energy than its monomeric counterpart (TS-1). As detailed in the ESI (see Table S9†), the relative stabilization of TS-2 occurs irrespective of the specific nature of the exchange correlation functional employed. However, the complex nature of the system and of its interaction with the environment effectively limit the confidence of quantitative computational estimates of reaction barriers in this case (the difference in the activation barrier between the pathways ranges from 7.3 kcal mol^−1^ (ωB97M-V) to 3.1 kcal mol^−1^ (ωB97M-D4)). The relatively small energy difference between the two pathways suggests that experimental variables like temperature and solvent nature are likely crucial to determine the actual mechanistic details of the transformation, *e.g.*, the reaction order with respect to the catalyst.

## Conclusions

We present evidence that the formation of aggregates of chiral phosphoric acid derivatives in solution is in principle possible for both standard (*e.g.*, CPA, DTPA and DSI) and confined (*e.g.*, IDP, and IDPi) catalysts, but it depends heavily on the reaction conditions; this might have a profound influence on the stereo-controlling factors of potentially a large number of organocatalytic transformations.

For CPA in solvents with low dielectric constants like toluene, the aggregation process is expected to be spontaneous at all temperatures (below the boiling point of the solvent). For DTPA and DSI, the aggregation process is less favorable, being spontaneous in toluene under mild reaction conditions, while higher temperatures and more polar solvents promote dissociation.

By contrast, for confined catalysts like IDPi, geometric strain makes the aggregation process much more challenging, and hence this process requires specific reaction conditions to become spontaneous. In the case study considered here, we showed that a positively charged molecule (nitronate) sitting between the phosphoric acid derivatives (the catalyst anion and the neutral silylated catalyst) drastically reduces aggregation energy and might favor the aggregation process through electrostatic and dispersion forces, while minimizing at the same time the geometrical strain. Remarkably enough, mechanistic investigations on the C–C coupling reaction with silyl ketene acetals indicate a faster reaction for the dimeric pathway at low temperatures (153 K), suggesting that cooperative catalytic effects between Brønsted acids might play an important role even for bulky and sterically demanding catalytic systems like IDPi.

## Data availability

The Cartesian coordinates of all structures, the results of benchmark calculations on the influence of electronic structure method, solvation scheme, dispersive interactions and basis set size on aggregation energies and reaction barriers as well as a detailed analysis of the key noncovalent interactions are provided in the ESI.†

## Author contributions

I. H. performed all the calculations. G. B. conceived and directed the project. I. H. and G. B. wrote the original draft of the manuscript and prepared the figures. I. H., F. N. and G. B. contributed to the data analysis and to the writing of the manuscript.

## Conflicts of interest

There are no conflicts to declare.

## Supplementary Material

SC-014-D3SC03769J-s001
