# Supplementary material for: Dimerization of confined Brønsted acids in enantioselective organocatalytic reactions
Source: Chem Sci. 2023 Sep 18;14(38):10580–90. doi: 10.1039/d3sc03769j (PMC10548523; doi:10.1039/d3sc03769j)
Supplement: SC-014-D3SC03769J-s001 [file SC-014-D3SC03769J-s001.pdf]

Electronic Supplementary Material (ESI) for Chemical Science.
This journal is © The Royal Society of Chemistry 2023

# Dimerization of Confined Brønsted Acids in Enantioselective Organocatalytic Reactions– Supporting Information

Ingolf Harden [a], Frank Neese [a], Giovanni Bistoni*[b]

[a]*Max-Planck-Institut für Kohlenforschung, Kaiser-Wilhelm Platz 1, 45470 Mülheim an der Ruhr (Germany)*

[b]*Department of Chemistry, Biology and Biotechnology, University of Perugia Via Elce di Sotto, 8, 06123 Perugia (Italy). E-mail: giovanni.bistoni@unipg.it*

**Table of Contents**

S1 Most stable dimer structures ....2
S2 Effect of the electronic structure method on the dimerization electronic energy ....3
S3 Effect of the dispersion correction scheme on the dimerization energy ....4
S4 Dispersion vs non-dispersion contributions to the dimerization energy ....5
S5 Effect of the solvent on the dimerization energy. ....7
S6 Effect of the DFT functional on the solvation energy ....10
S7 Decomposition of $\Delta G_{corr}$ into thermochemical and solvation corrections ....10
S8 Basis set extrapolation for DTPA ....11
S9 Noncovalent interaction analysis in I-2 ....12
S10 Comparison of r$^2$SCAN-3c and r$^2$SCAN-3c/GFN2-xTB optimized structures ....13
S11 Reaction energies at various levels of theory ....14
S12 Reaction barriers at different temperatures ....15
S13 References ....16
S14 XYZ Coordinates ....16

## S1 Most stable dimer structures

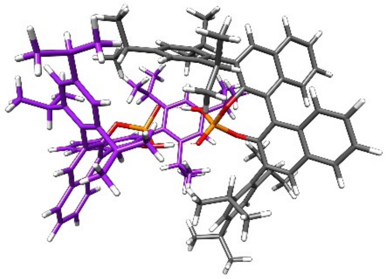

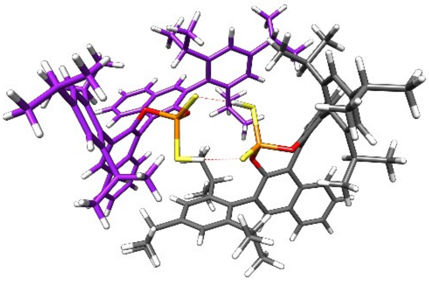

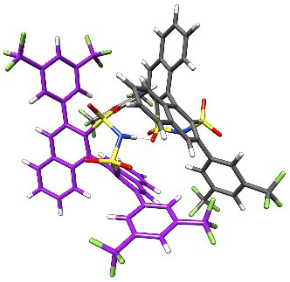

| | CPA | DTPA | DSI |
|---|---|---|---|
| **$\Delta H_{Dim}$ (kcal mol$^{-1}$)** | **-37.9** | **-17.1** | **-23.4** |
| **$\Delta G_{Dim}$ (kcal mol$^{-1}$)** | **-25.5** | **-5.7** | **-12.3** |

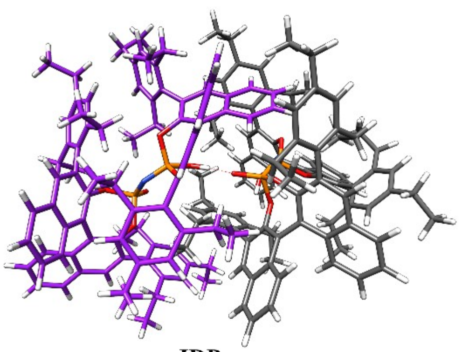

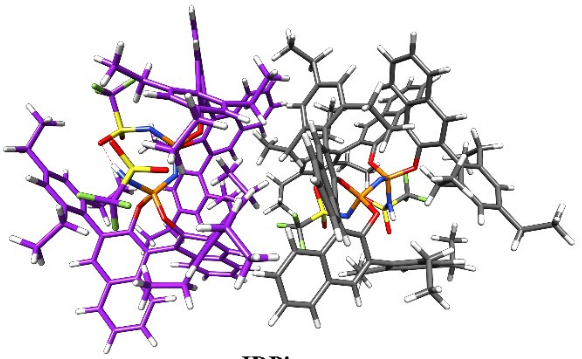

| | IDP | IDPi |
|---|---|---|
| **$\Delta H_{Dim}$ (kcal mol$^{-1}$)** | **6.0** | **5.2** |
| **$\Delta G_{Dim}$ (kcal mol$^{-1}$)** | **16.9** | **15.7** |

**Figure S1.** Most stable dimer conformers for **CPA, DTPA, DSI, IDP** and **IDPi** together with the corresponding dimerization enthalpies ($\Delta H_{dim}$) and free dimerization energies ($\Delta G_{dim}$) at the ωB97M-V/def2-TZVP level at 173 K. All energies are in kcal mol$^{-1}$. For a discussion of the structural features, see main text.

## S2 Effect of the electronic structure method on the dimerization electronic energy

**Table S1**. Dimerization energies $\Delta E_{dim}$ for different acids (**CPA, DTPA, DSI, IDP, IDPi**) at various levels of theory *in vacuo*. All energies are in kcal mol$^{-1}$. All DFT calculations were carried out with the def2-TZVP basis set. HFLD calculations were carried out with the def2-TZVP(-f) basis set and NormalPNO settings with a TCutPairs cutoff of 1e-05. †: Geometric preparation energy was calculated at the ωB97M-V level of theory. *: Basis set extrapolation with the def2-TZVP and def2-QZVP basis sets was carried out, TightPNO settings were used.

| | **CPA** | **DTPA** | **DSI** | **IDP** | **IDPi** |
|---|---|---|---|---|---|
| B3LYP-D3BJ | -46.7 | -26.7 | -33.0 | -4.1 | -5.1 |
| B3LYP-ABC | -43.7 | -23.6 | -30.0 | -0.4 | -2.0 |
| M06-2x-D3 | -48.7 | -22.6 | -31.2 | -3.9 | -2.4 |
| ωB97M-V | -48.1 | -25.5 | -36.0 | -5.0 | -5.2 |
| HFLD† | -40.7 | -32.9* | -31.0 | -6.4 | 0.4 |

As can be seen from **Table S1**, fairly similar dimerization energies are obtained for the different density functionals. Interestingly, the three-body dispersion correction used in the B3LYP-ABC calculations increase the dimerization energy by a relatively constant amount between 3.0 kcal mol$^{-1}$ and 3.7 kcal mol$^{-1}$. In addition, interaction energies obtained by HFLD are systematically smaller than their DFT counterparts. For **DTPA**, we noticed a more pronounced basis set incompleteness error. Hence, HFLD calculations were carried out with a larger basis set and TightPNO settings. **DTPA** features two S-H hydrogen bonds and it is suspected that the smaller def2-TZVP(-f) basis set is not appropriate for describing the extended charge distribution of sulfur sufficiently well.

**S3 Effect of the dispersion correction scheme on the dimerization energy**

For the **DTPA** dimer, the electronic dimerization energy was computed using different approximations for the London dispersion correction within the density functional theory framework. ωB97M/def2-TZVP calculations were carried out with D3, D4 [1] and NL dispersion corrections, respectively. For the NL calculations, the VV10 kernel was used in a post-SCF fashion, as detailed in the main text.

**Table S2**. Electronic dimerization energy of **DTPA** at the ωB97M/def2-TZVP level of theory. Dispersion corrections were included at the D3BJ/D4/VV10 level, respectively. All energies are in kcal $mol^{-1}$.

| | $\Delta E_{dim}$ |
|---|---|
| ωB97M-D3BJ | -22.6 |
| ωB97M-D4 | -18.2 |
| ωB97M-V | -25.5 |

Interestingly, deviations between the different dispersion schemes are relatively large. This is especially true for D4 which differs much larger from D3 and VV10 than those two differ from each other.

**S4 Dispersion vs non-dispersion contributions to the dimerization energy**

**Table S3**. Decomposition of the interaction energies between the monomers in their dimer geometries for different acids (**CPA, DTPA, DSI, IDP, IDPi**) at various levels of theory. All energies are in kcal mol$^{-1}$. *: Basis set extrapolation with def2-TZVP and def2-QZVP was carried out, TightPNO settings were used.

| | | **CPA** | **DTPA** | **DSI** | **IDP** | **IDPi** |
|---|---|---|---|---|---|---|
| B3LYP-D3BJ | $E_{dim}^{disp}$ | -47.0 | -51.9 | -45.6 | -86.3 | -53.3 |
| | $E_{dim}^{non-disp}$ | -19.2 | 11.2 | 3.0 | 12.2 | 14.9 |
| B3LYP-ABC | $E_{dim}^{disp}$ | -43.9 | -48.8 | -42.7 | -80.3 | -49.5 |
| | $E_{dim}^{non-disp}$ | -19.2 | 11.2 | 3.0 | 12.2 | 14.9 |
| ωB97M-V | $E_{dim}^{disp}$ | -38.6 | -39.7 | -39.4 | -69.6 | -44.3 |
| | $E_{dim}^{non-disp}$ | -28.1 | 1.1 | -6.0 | -6.6 | 2.8 |
| HFLD | $E_{dim}^{disp}$ | -46.3 | -71.9* | -49.3 | -87.0 | -53.3 |
| | $E_{dim}^{non-disp}$ | -12.1 | 25.7* | 8.9 | 24.2 | 21.1 |

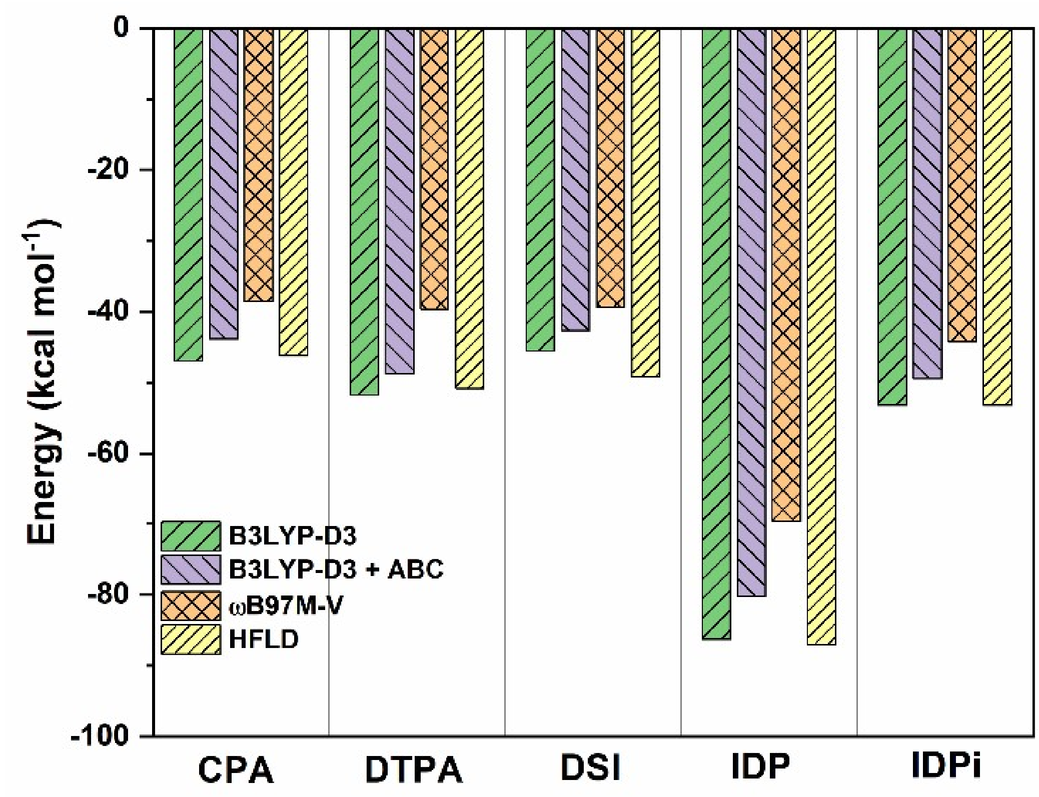


**Figure S2**. London dispersion contribution to $\Delta G_{Dim}$ at various levels of theory. All energies are in kcal mol$^{-1}$.

**S5 Effect of the solvent on the dimerization energy.**

In this section the influence of different solvation schemes (C-PCM vs. SMD) and the influence of the solvent on dimerization energies are discussed. The C-PCM calculations were performed using the gaussian charge scheme [2] as implemented in ORCA.

**Table S4**. Dimerization energies for various acids (**CPA, DTPA, DSI, IDP, IDPi**) with the C-PCM and SMD solvation schemes. All calculations were carried out at the B3LYP-D3BJ/def2-TZVP level of theory. All energies are in kcal mol$^{-1}$.

| | $\Delta E_{dim}$ (C-PCM) | $\Delta E_{dim}$ (SMD) | $\Delta\Delta E_{dim}$ (SMD – C-PCM) |
|---|---|---|---|
| **CPA** | -42.2 | -37.7 | 4.5 |
| **DTPA** | -24.3 | -18.7 | 5.6 |
| **DSI** | -26.5 | -22.2 | 4.3 |
| **IDP** | -0.8 | 6.2 | 7.0 |
| **IDPi** | -3.0 | 4.6 | 7.6 |

As can be seen from **Table S4**, the solvation scheme has significant influence on the dimerization energies. For all acid dimers, SMD predicts larger (closer to zero) dimerization energies. This effect increases with the size of the acid and is therefore largest for **IDPi**. Comparing the dimerization energies in solution (**Table S4**) with the dimerization energies *in vacuo* (**Table S1**), it becomes evident that implicit solvation favors dissociation for all acid dimers. The association process decreases the solvent accessible surface and therefore consequently reduces the interactions between solute and solvent. SMD takes into account short-range interactions like London dispersion between solute and solvent. These interactions are important for an accurate description of the dimerization energy as well.

The influence of the solvation scheme and solvent was also studied for the aggregation of **I-1** and **X-TBS** to form **I-2** (see **Figure 4**, main text). In **Figure S3**, the reaction energy for the Reaction **I-1** + **X-TBS** -> **I-2** is shown as a function of the dielectric constant. In practice, calculations for five different solvents (toluene, acetone, water, dichloromethane, methanol) were carried out.

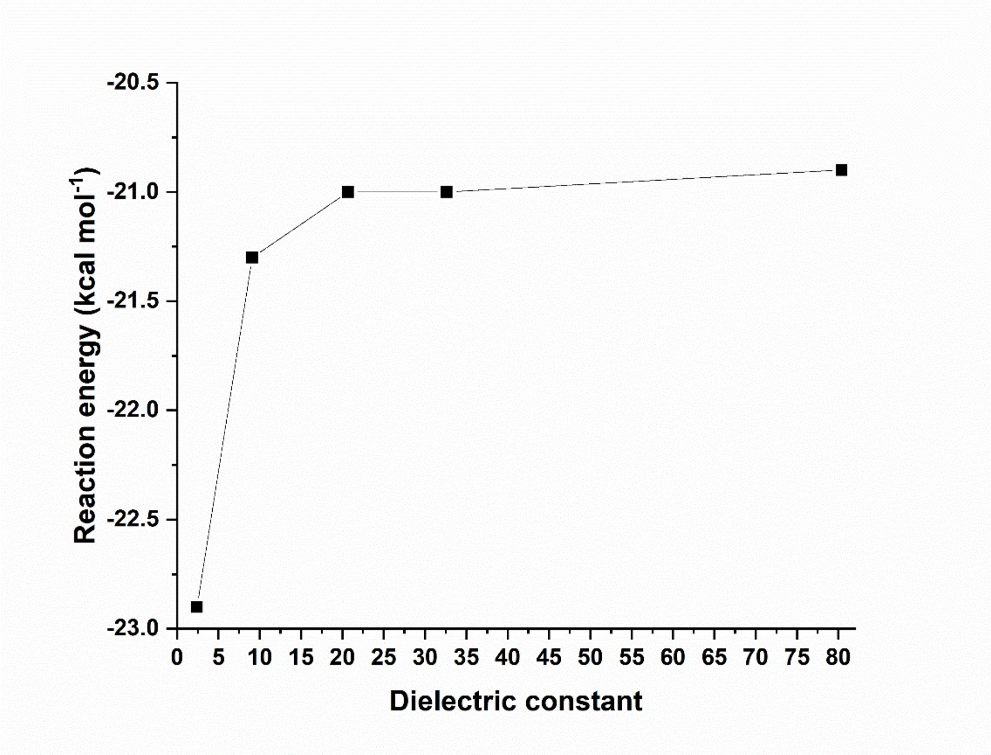


**Figure S3.** Electronic reaction energy for the association of **I-1** with **X-TBS** for five different solvents (toluene, acetone, water, dichloromethane, methanol) at the ωB97M-V/def2-TZVP level of theory. All energies are in kcal mol$^{-1}$. Free solvation enthalpies were calculated using the C-PCM model.

Increasing solvent polarity decreases the reaction energy in absolute value. Interestingly, the reaction energy changes most for the change from toluene ($\varepsilon = 2.4$) to dichloromethane ($\varepsilon = 9.1$). Increasing the polarity of the solvent further only has minor effects on the reaction energy.

It should be kept in mind, however, that the description of the solute-solvent interaction with the C-PCM scheme misses the dispersive interactions between solute and solvent. For toluene and dichloromethane additional calculations with the SMD model were carried out.

**Table S5**. Electronic reaction energy for the association of **I-1** with **X-TBS** at the ωB97M-V/def2-TZVP level of theory with C-PCM and SMD (toluene and dichloromethane, respectively). All energies are in kcal $mol^{-1}$.

| | **Reaction energy** |
|---|---|
| C-PCM(Toluene) | -22.9 |
| SMD(Toluene) | -18.8 |
| C-PCM($CH_2Cl_2$) | -21.3 |
| SMD($CH_2Cl_2$) | -18.0 |

Like for the acid dimers, the SMD model favors dissociation much stronger than the C-PCM model, as can be seen from **Table S5**. However, the change in free energies when replacing toluene by dichloromethane is smaller for SMD (1.6 kcal $mol^{-1}$ for C-PCM, 0.8 kcal $mol^{-1}$ for SMD).

## S6 Effect of the DFT functional on the solvation energy

For **CPA**, the free solvation energies were calculated using the C-PCM model and toluene as solvent at the B3LYP-D3/def2-TZVP and ωB97M-V/def2-TZVP levels of theory. The solvation contribution to the free dimerization energy amounts to 4.5 kcal mol$^{-1}$ at the B3LYP-D3 level and 4.2 kcal mol$^{-1}$ at the ωB97M-V level. The difference in contributions is only 0.3 kcal mol$^{-1}$, thus showing that the implicit solvation correction is fairly independent of the underlying density functional used and that the choice of the implicit solvation model (C-PCM vs. SMD) is much more important in this regard.

## S7 Decomposition of $\Delta G_{corr}$ into thermochemical and solvation corrections

In **Table S6**, we show the decomposition of the correction term $\Delta G_{corr}$ that was introduced in **Figure 3** into its contributions from implicit solvation and thermochemistry.

**Table S6**. Decomposition of the correction term $\Delta G_{corr}$ to the free dimerization energy at 173 K. Thermochemical corrections were computed at the PBE-D3/def2-SVP level of theory, solvation corrections were computed at the B3LYP-D3/def2-TZVP/SMD(Toluene) level of theory. All energies are in kcal mol$^{-1}$.

| | $\Delta G_{corr}$ | $\Delta G_{solv}$ | $\Delta G_{thermo}$ |
|---|---|---|---|
| **CPA** | 22.6 | 9.0 | 13.6 |
| **DTPA** | 19.7 | 8.0 | 11.7 |
| **DSI** | 23.7 | 10.7 | 13.0 |
| **IDP** | 21.9 | 10.3 | 11.6 |
| **IDPi** | 21.0 | 9.7 | 11.3 |

Interestingly, implicit solvation and thermochemistry both contribute significantly to $\Delta G_{corr}$, with thermochemical corrections slightly being more important. It is noted here that thermochemical

corrections in **Table S6** were computed at 173 K. The contribution from implicit solvation is roughly temperature independent so that at lower temperatures $\Delta G_{corr}$ should be dominated by solvation effects. As has been discussed before (**Table S4, Table S5**), solute-solvent dispersive interactions play an important role for the magnitude of $\Delta G_{solv}$.

**S8 Basis set extrapolation for DTPA**

For the electronic dimerization energy of **DTPA**, basis set extrapolation was carried out. For achieving this, single point energy calculations of the monomer and the dimer were carried out at the ωB97M-V with the def2-SVP, def2-TZVP and def2-QZVP basis sets, respectively. The def2-TZVP and def2-QZVP were used further to compute the (3/4) extrapolated dimerization energy. The results are shown in **Table S7**.

**Table S7**. Electronic dimerization energy of **DTPA** at the ωB97M-V level of theory for different basis set sizes. All energies are in kcal mol$^{-1}$.

| **Basis set** | **$\Delta E_{dim}$** |
|---|---|
| def2-SVP | -32.2 |
| def2-TZVP | -25.5 |
| def2-QZVP | -24.0 |
| (3/4)-extrapolation | -23.6 |

As can be seen from **Table S7**, basis sets of at least triple-zeta quality are required to get a reasonable accurate description of the dimerization energy.

**S9 Noncovalent interaction analysis in I-2**

**I-2** consists of three individual molecules. The catalyst anion **X**, the nitronate cation **N** and the activated catalyst molecule **X-TBS**. **X** and **N** already form a dimer in **I-1**. In the first step, the geometric preparation energies of the fragments in **I-2** were computed. For **X** and **N,** the corresponding geometries in **I-1** were taken as reference. For **X-TBS** the equilibrium structure was taken as reference. The geometric preparation energies were calculated at the ωB97M-V/def2-TZVP level of theory using the r$^2$SCAN-3c/xTB optimized geometries (see main text for details) and account for -1.2 kcal mol$^{-1}$ for **X**, 8.7 kcal mol$^{-1}$ for **N** and 1.2 kcal mol$^{-1}$ for **X-TBS**. Interestingly, the overall geometric preparation is very small, especially compared to the **IDPi** dimer (see **Figure 3**, main text). This indicates that all three fragments do not have to distort much to optimize their interaction. The low geometric preparation is therefore one of the driving forces that possibly might lead to dimerization. Dispersive interactions between the fragments were calculated at the HFLD level of theory. For the computation of the non-dispersive interactions, Hartree-Fock single point calculations of each fragment and each pair of fragments were carried out.

**Table S8**. Fragment-pairwise dispersive and non-dispersive interactions in **I-2** at the HFLD/def2-TZVP(-f) level of theory. All energies are in kcal mol$^{-1}$.

| **Fragment pair** | $E_{disp}$ | $\Delta E_{non\text{-}disp}$ |
|---|---|---|
| **X, N** | -47.5 | -50.6 |
| **X, X-TBS** | -5.7 | 7.3 |
| **N, X-TBS** | -18.4 | -2.4 |

As expected, very strong dispersive and non-dispersive interactions arise between the catalyst anion **X** and the nitronate **N** in **I-2,** which are the driving force for the formation of **I-1** in the first place. Interestingly, **X** and **X-TBS** feature very weak dispersive interactions, while non-dispersive

interactions are repulsive and slightly larger in magnitude leading to a weak overall repulsion between **X** and **X-TBS**. The nitronate **N** and **X-TBS** are held together by strong dispersive interactions and weak attractive non-dispersive interactions. In contrast to the **IDP** and **IDPi** acid dimers case (**Figure 3**), in **I-2** the nitronate seems to act as "glue" between **X** and **X-TBS**, allowing for strong intermolecular interactions without the need of significant distortions. These much more favorable interactions lead to free dimerization energies of approximately 0 kcal $mol^{-1}$ at 153 K (see **Figure 4**).

**S10 Comparison of $r^2$SCAN-3c and $r^2$SCAN-3c/GFN2-xTB optimized structures**

Geometry optimizations of the conformers of the species in **Figure 4** were performed at the $r^2$SCAN-3c/GFN2-xTB level of theory using the multiscale approach in ORCA, as discussed in the main text. Final single point energies were obtained at various levels of theory. The aggregation energy of **I-2** was recomputed using $r^2$SCAN-3c optimized structures. Computation of the formation energies of **I-2** *in vacuo* gives -22.9 kcal $mol^{-1}$ for the $r^2$SCAN-3c/ GFN2-xTB optimized structures and -22.2 kcal $mol^{-1}$ for the $r^2$SCAN-3c optimized structures at the ωB97M-V/def2-TZVP level of theory, thus justifying the use of the computationally less demanding ONIOM approach.

## S11 Reaction energies at various levels of theory

In **Table S9** the relative free energies of the species **I-1, RC-1, TS-1, P-1, I-2, RC-2, TS-2** and **P-2** are given at various levels at theory.

**Table S9**. Computed free energies for the reaction of the monomeric (**I-1**) and dimeric (**I-2**) ion pair with the nucleophile **2** at different levels of theory. Geometries and thermal corrections at 153 K were obtained at the $r^2$SCAN-3c/xTB level of theory. Final single point energy calculations with the i) B3LYP-D3, ii) B3LYP-D3 + ABC, iii) ωB97M-V and iv) ωB97M-D4 functionals were carried out with the def2-TZVP basis set and the SMD solvation model ($CH_2Cl_2$). The gCP correction was included in all cases. All free energies are relative to that of **I-1**. All energies are in kcal $mol^{-1}$.

| | B3LYP-D3 | B3LYP-D3 + ABC | ωB97M-V | ωB97M-D4 |
|---|---|---|---|---|
| **I-1** | 0.0 | 0.0 | 0.0 | 0.0 |
| **RC-1** | 8.3 | 9.6 | 9.1 | 11.1 |
| **TS-1** | 14.2 | 15.7 | 16.7 | 19.5 |
| **P-1** | -2.1 | -0.8 | -8.0 | -3.8 |
| **I-2** | -0.8 | 2.2 | -0.8 | 4.1 |
| **RC-2** | 4.6 | 8.5 | 5.0 | 11.2 |
| **TS-2** | 7.8 | 11.8 | 9.4 | 16.4 |
| **P-2** | -8.2 | -4.2 | -14.6 | -6.1 |

Remarkably enough, the aggregation of **I-1** with **X-TBS** to give **I-2** features a free reaction energy ( $\Delta G_R$) close to zero. While B3LYP-D3 and ωB97M-V predict slightly negative $\Delta G_R$ values, B3LYP-D3 + ABC and ωB97M-D4 predict a slightly endergonic association process. The direct comparison of B3LYP-D3 and B3LYP-D3 + ABC results reveals that many-body dispersion corrections favor dissociation. The small value of $\Delta G_R$ suggests that the spontaneity of this process might change for

different substituents and/or for different reaction conditions. The difference in the activation barrier between the pathways ranges from 7.3 kcal/mol (ωB97M-V) to 3.1 kcal $mol^{-1}$ (ωB97M-D4). While the significant variations observed with different functionals as well as the complex nature of the system and of its interaction with the environment effectively limit the confidence of quantitative computational estimates of reaction barriers in this case, the relatively small energy difference between the two pathways suggest that experimental variables like temperature and solvent nature are likely crucial to determine the actual mechanistic details of the transformation*, e.g.,* the reaction order with respect to the catalyst.

### S12 Reaction barriers at different temperatures

In **Table S10**, the activation barriers for the monomeric and dimeric pathways are shown for different temperatures.

**Table S10**. Computed reaction barriers for the reaction of the monomeric (**TS-1**) and dimeric (**TS-2**) pathway at the ωB97M-V/def2-TZVP level of theory at different temperatures. All energies are in kcal $mol^{-1}$.

| **Temperature (K)** | $\Delta G^{\ddagger}$ **(TS-1)** | $\Delta G^{\ddagger}$ **(TS-2)** | $\Delta\Delta G^{\ddagger}$ **(TS-2 – TS-1)** |
|---|---|---|---|
| **153.15** | 16.7 | 9.4 | -7.3 |
| **173.15** | 17.9 | 11.9 | -6.0 |
| **193.15** | 19.1 | 14.5 | -4.7 |

The temperature has large impact on the reaction barriers with higher temperatures favoring the monomeric pathway. The large deviations in activation barriers found for different exchange-correlation functionals have no influence on their temperature dependence as the temperature dependence is governed by entropy.

## S13 References


[1] E. Caldeweyher, S. Ehlert, A. Hansen, H. Neugebauer, S. Spicher, C. Bannwarth, S. Grimme, *The Journal of Chemical Physics* **2019**, *150*, 154122.

[2] M. Garcia-Ratés, F. Neese, *Journal of Computational Chemistry* **2020**, *41*, 922-939.


## S14 XYZ Coordinates

112

CPA Monomer

| | | | |
|---|---|---|---|
| C | 3.77206190407251 | -0.76791343120771 | -1.31986945817998 |
| C | 3.57215151681695 | -0.36983400716490 | 0.02888725472592 |
| C | 4.48643144913036 | -1.95138747751393 | -1.57844273097015 |
| C | 3.26428241126354 | 0.06137615752097 | -2.49604742878370 |
| C | 2.82273563664126 | 0.88770202084147 | 0.33003820507075 |
| C | 4.07168139381630 | -1.17182343990755 | 1.09307084959380 |
| C | 5.00308238527750 | -2.75248726493136 | -0.54878439152298 |
| H | 4.64865905087704 | -2.26516544771246 | -2.62277413164037 |
| C | 1.42176548393041 | 0.97043738100766 | 0.06715931018331 |
| C | 3.43738439492038 | 2.01096475770045 | 0.87045213924009 |
| C | 4.78327741215445 | -2.34152533759199 | 0.77665578988166 |
| C | 3.86581871486868 | -0.80277776937886 | 2.56032716078570 |
| O | 0.80627938473644 | -0.15279321823500 | -0.48426045400421 |
| C | 0.65545592193924 | 2.12074952738620 | 0.29742623315667 |
| H | 4.52126472946647 | 1.98822425148254 | 1.05831291454771 |
| C | 2.70158863784362 | 3.17532677726885 | 1.22979147503887 |
| H | 5.16864263426800 | -2.96086627862879 | 1.60151057653383 |
| P | -0.12617071188605 | -1.03102980432856 | 0.56226509760586 |
| C | 1.28465210728079 | 3.23157916180568 | 0.96569187771354 |
| C | -0.75329554439614 | 2.16083628205807 | -0.17872169329135 |
| C | 3.33380693226152 | 4.28240343945534 | 1.86972747055896 |
| O | -1.21946660789673 | 0.12092180034618 | 1.01908932386301 |
| C | 0.55684043315626 | 4.37397072480891 | 1.41264957214706 |
| C | -1.23724036148562 | 3.19913643829828 | -1.05918920449995 |
| C | -1.62373943399955 | 1.11449726406293 | 0.13655344838315 |
| H | 4.41787733756708 | 4.23089292556622 | 2.05557098439809 |
| C | 2.60165453904865 | 5.38999581306897 | 2.26755984129424 |
| C | 1.19914694510203 | 5.42638254654848 | 2.04884857698478 |
| H | -0.53027662445944 | 4.40767885509376 | 1.25679223577180 |
| C | -0.39979721073673 | 4.23439739284282 | -1.57191550850517 |
| C | -2.61981662477761 | 3.17716333538283 | -1.47560163962237 |
| C | -2.94751404558790 | 1.00181127855290 | -0.38959511356822 |
| H | 3.10124115702538 | 6.23384804697141 | 2.76673043794408 |
| H | 0.61491536226271 | 6.29337236220078 | 2.39239600606250 |
| H | 0.66746755859822 | 4.23498278103835 | -1.31524202572489 |
| C | -0.91081627737541 | 5.23016519933087 | -2.39209310182958 |
| C | -3.11790788811056 | 4.22712735166270 | -2.30258529380387 |
| C | -3.44130699646630 | 2.06644939638327 | -1.13012271943989 |

| | | | |
|---|---|---|---|
| H | -0.24097183395737 | 6.01460066339285 | -2.77541329257547 |
| C | -2.28511931706120 | 5.24190014328851 | -2.74652344825240 |
| H | -4.17810940020602 | 4.20201452772625 | -2.59904441153552 |
| H | -4.47106720925836 | 2.02710286047187 | -1.51569937520632 |
| H | -2.68049763734937 | 6.04269780910975 | -3.38930517921965 |
| C | 5.77137189680737 | -4.02908062900901 | -0.86006753914608 |
| C | 5.06723900777689 | -5.27017185563708 | -0.28637425976049 |
| C | 7.23007758440006 | -3.94150550643640 | -0.38019645120984 |
| C | 3.32895932982029 | -1.97645816158457 | 3.39457358114700 |
| C | 5.16271972716898 | -0.23690006256139 | 3.16742358177603 |
| C | 4.43304968515079 | 0.63466054820768 | -3.31647127003289 |
| C | 2.28744547945512 | -0.73212308116463 | -3.37994567751399 |
| O | 0.54282734927302 | -1.65772326239544 | 1.72848910526806 |
| O | -0.88885978016511 | -2.01398628561292 | -0.46001191441578 |
| H | 2.70583825871528 | 0.92640125911032 | -2.08498635634613 |
| H | 3.09537795355363 | -0.00671659683441 | 2.59702441611154 |
| C | -3.65715470204687 | -0.30735277276205 | -0.25050872093694 |
| H | 5.78702886936174 | -4.13245686431817 | -1.96742828308729 |
| H | 5.02784459129868 | -5.23237449922945 | 0.82204836455633 |
| H | 5.60571956660607 | -6.19790260018773 | -0.57032330609383 |
| H | 4.02463738047061 | -5.34778288988776 | -0.65392984933165 |
| H | 7.74600305960005 | -3.06347496568900 | -0.81765180489523 |
| H | 7.79685621396003 | -4.85270918249294 | -0.66272145470848 |
| H | 7.28237407279643 | -3.84431680310434 | 0.72428669033332 |
| H | 3.11989061596138 | -1.64047173707356 | 4.43114164201410 |
| H | 4.06147312153231 | -2.80765829414651 | 3.46361271900936 |
| H | 2.38727166148810 | -2.35960579312914 | 2.95900116499834 |
| H | 5.96996753207175 | -0.99895254139128 | 3.15418939004387 |
| H | 5.00429581808242 | 0.07121784880032 | 4.22157056189530 |
| H | 5.53228740270038 | 0.64333765020952 | 2.60414661742767 |
| H | 4.05860738564227 | 1.28176553043375 | -4.13655607387394 |
| H | 5.03816250939807 | -0.17391258864137 | -3.77664308155276 |
| H | 5.11134751286369 | 1.23994524302725 | -2.68194035024651 |
| H | 1.88357364903075 | -0.08813704062627 | -4.18854069989673 |
| H | 1.43660745718628 | -1.11328084392396 | -2.78279666639012 |
| H | 2.78655539276057 | -1.59764802213373 | -3.86333953315574 |
| H | -1.85320962796387 | -1.80190796561549 | -0.55714126100205 |
| C | -3.70277062640039 | -1.18242123682849 | -1.37950052648531 |
| C | -4.27864371695826 | -2.45943247966267 | -1.21450725510417 |
| C | -4.80354959339675 | -2.89498902842746 | 0.01271354503013 |
| C | -4.74925698384428 | -2.00985935923481 | 1.10362555476502 |
| C | -4.17806403417813 | -0.73042709702897 | 1.00184779027266 |
| C | -3.18482047443545 | -0.76519522570645 | -2.75754788311490 |
| C | -4.14641112578349 | 0.17865738398684 | 2.22423731429427 |
| C | -5.37144352322869 | -4.29838778740329 | 0.16104463168498 |
| C | -6.82463922306735 | -4.28623122743673 | 0.66139675634777 |
| C | -4.47086155631540 | -5.16031069339073 | 1.06374840215550 |
| C | -5.56594630529776 | 0.65049681602583 | 2.58600072535317 |
| C | -3.44397405067566 | -0.48359848847512 | 3.42061704006131 |
| C | -2.37518100681717 | -1.85860037571876 | -3.47345363276345 |

| | | | |
|---|---|---|---|
| C | -4.35317736377458 | -0.29103462078547 | -3.64176366981084 |
| H | -4.31160664633725 | -3.14556442172085 | -2.07514167456757 |
| H | -5.15024662178176 | -2.33172034482912 | 2.07758488551429 |
| H | -2.50622551681087 | 0.09929013663453 | -2.60949747251881 |
| H | -3.56204249395537 | 1.08101907040901 | 1.95209112037736 |
| H | -5.36650145206733 | -4.75418205426724 | -0.85317271338730 |
| H | -6.89605558909982 | -3.86063328271793 | 1.68380767850677 |
| H | -7.23416429275995 | -5.31609499342219 | 0.70342423812463 |
| H | -7.47634380559936 | -3.68200313274611 | -0.00083136203458 |
| H | -4.44595174475492 | -4.76169416464637 | 2.09921431105714 |
| H | -3.42795699781407 | -5.18167578439495 | 0.68953241010730 |
| H | -4.84368451146804 | -6.20387908752419 | 1.11389884158266 |
| H | -6.05121415089440 | 1.16326884979828 | 1.73119476880640 |
| H | -5.53767541070472 | 1.35740745381483 | 3.44041006146846 |
| H | -6.21307864887388 | -0.20310560409171 | 2.87723556260664 |
| H | -2.41760821854020 | -0.80212530755104 | 3.15464856081744 |
| H | -4.00282276675895 | -1.37231274161017 | 3.78034444987778 |
| H | -3.37368825523292 | 0.22699063527779 | 4.26920538989512 |
| H | -1.95078568209950 | -1.45616395784644 | -4.41527632629038 |
| H | -3.00237089979771 | -2.73161346089553 | -3.74838958146584 |
| H | -1.53364984669026 | -2.22075094969694 | -2.85161857133909 |
| H | -4.91850955903085 | 0.53430670817135 | -3.16691381862112 |
| H | -5.06694758737486 | -1.12021531755679 | -3.82879108987383 |
| H | -3.98195777905224 | 0.06736717912285 | -4.62367714392336 |

224

CPA Dimer

| | | | |
|---|---|---|---|
| C | -4.87772110050053 | -3.17876804662211 | -1.37836515055914 |
| C | -4.24182695234095 | -2.18621845822683 | -2.17176654333993 |
| C | -4.46484046083738 | -4.51730238612002 | -1.51355395453418 |
| C | -5.97017568827634 | -2.80960608709437 | -0.37786102468794 |
| C | -4.77697207918740 | -0.78608008053002 | -2.14538813748309 |
| C | -3.20339597174328 | -2.54389591714373 | -3.07200833275128 |
| C | -3.46778484888298 | -4.90337043086576 | -2.42359168385378 |
| H | -4.94989678932885 | -5.29520363651606 | -0.90306163244126 |
| C | -4.32559876379049 | 0.21754602277246 | -1.23492273720258 |
| C | -5.82752402620362 | -0.45691609113840 | -2.99587952028514 |
| C | -2.84689433382499 | -3.89900425421216 | -3.18542103070342 |
| C | -2.52718566589469 | -1.49933653567164 | -3.95541889795508 |
| O | -3.27536792691675 | -0.12008489353050 | -0.37326372224905 |
| C | -4.93422110120065 | 1.47508995002742 | -1.12429963730634 |
| H | -6.21097692166916 | -1.22722573207386 | -3.68234668917619 |
| C | -6.41867764470879 | 0.83480213300569 | -3.01259564905936 |
| H | -2.05626628541294 | -4.17650376173396 | -3.89854529498698 |
| P | -1.82969346688731 | 0.64870853106457 | -0.47830156250167 |
| C | -5.95996593109504 | 1.82846419624484 | -2.07579100910996 |
| C | -4.51064060848885 | 2.45166790429661 | -0.08524884853018 |
| C | -7.44551618899552 | 1.16915827322203 | -3.94460148044421 |
| O | -2.25804923979669 | 2.18593788048409 | -0.83201597516459 |
| C | -6.51823293721300 | 3.13966184150044 | -2.15919765198307 |
| C | -5.44284348714005 | 3.05233506219504 | 0.83454529720954 |

| | | | |
|---|---|---|---|
| C | -3.17161559367517 | 2.82658858322883 | 0.00201057705918 |
| H | -7.79020698487119 | 0.39279172304225 | -4.64510710516142 |
| C | -7.98501268089056 | 2.44468686753917 | -3.98049937734338 |
| C | -7.50397208082823 | 3.43896999641140 | -3.08806577088404 |
| H | -6.14939146388115 | 3.92344311012736 | -1.48454104920379 |
| C | -6.79449701611655 | 2.61694375892204 | 0.95932812616120 |
| C | -4.98197397254918 | 4.12188923038881 | 1.68578513489714 |
| C | -2.67399098550915 | 3.85118052031716 | 0.86578486414673 |
| H | -8.77246158120029 | 2.69377911058869 | -4.70761621372876 |
| H | -7.91162605420293 | 4.45995284020414 | -3.13697102359051 |
| H | -7.14348637202928 | 1.77707941125455 | 0.34268661671260 |
| C | -7.66096640004574 | 3.22934141987622 | 1.85369346389405 |
| C | -5.90475000402616 | 4.74298106116442 | 2.57880266930082 |
| C | -3.61250725368488 | 4.51201788377698 | 1.65125657975188 |
| H | -8.69764026479835 | 2.87058227791508 | 1.94056071831196 |
| C | -7.21939296915553 | 4.31087394846350 | 2.66075719416818 |
| H | -5.54449458973117 | 5.56475739266458 | 3.21710012557055 |
| H | -3.28321597554823 | 5.33258495633703 | 2.30504668020324 |
| H | -7.91786146358961 | 4.79397643971230 | 3.36046792539227 |
| C | -3.11647099317182 | -6.37333077269671 | -2.61254359174985 |
| C | -1.60852850388689 | -6.65020892157498 | -2.52069661354500 |
| C | -3.69637318415603 | -6.89615893410851 | -3.93957811264034 |
| C | -1.02029703230355 | -1.73800530349892 | -4.13271047477975 |
| C | -3.22711322703644 | -1.40962308961398 | -5.32427285468564 |
| C | -7.13235139851298 | -3.81194346058444 | -0.34450557039031 |
| C | -5.37135523378107 | -2.60228062935126 | 1.02471385014470 |
| O | -0.92168968159238 | 0.17711113590698 | -1.59313629548294 |
| O | -1.30794270397635 | 0.54786475761775 | 1.00034470484754 |
| H | -6.39041334735520 | -1.83498011063036 | -0.70135796404714 |
| H | -2.64001935239374 | -0.51820465941915 | -3.45053796881858 |
| C | -1.20136172239046 | 4.07784121481942 | 0.94961447509936 |
| H | -3.61158898444863 | -6.92974705498128 | -1.78642536185221 |
| H | -1.19339243948385 | -6.31063641833083 | -1.55145152379413 |
| H | -1.04788614788010 | -6.13243942692693 | -3.32618340869793 |
| H | -1.40219935719381 | -7.73537843107244 | -2.62128646659966 |
| H | -4.79131296835783 | -6.73264940301436 | -3.99208511361177 |
| H | -3.50071415656450 | -7.98171199516306 | -4.06106750447303 |
| H | -3.23787727288870 | -6.37005507368657 | -4.80280694744010 |
| H | -0.50863232194724 | -1.85512029125492 | -3.15920732157452 |
| H | -0.55948270597353 | -0.87393091945960 | -4.65338355055325 |
| H | -0.81204208538862 | -2.63722854305449 | -4.74895415537926 |
| H | -3.16366214945973 | -2.38050806940281 | -5.85867102787346 |
| H | -2.74833071209446 | -0.63668359383047 | -5.96017312255764 |
| H | -4.29910819533605 | -1.15006939095788 | -5.22242549369419 |
| H | -7.94909881760355 | -3.43127588567163 | 0.30196772904841 |
| H | -6.82420378672610 | -4.79406642731509 | 0.06991160657442 |
| H | -7.54814775229928 | -3.98809962904934 | -1.35693861936212 |
| H | -6.14905105965947 | -2.27974384977933 | 1.74785016780265 |
| H | -4.57549674872011 | -1.83368677673922 | 1.01133729578774 |
| H | -4.92546373454478 | -3.54646538839841 | 1.40262520868345 |

| | | | |
|---|---|---|---|
| H | -0.25754682397228 | 0.56403244518613 | 1.08252528545274 |
| C | -0.51405971056692 | 3.79506267600538 | 2.16572881471334 |
| C | 0.89027370376547 | 3.84418117033504 | 2.17529474803601 |
| C | 1.64044644655143 | 4.14650266931593 | 1.02943316542940 |
| C | 0.93916889318744 | 4.46712999251366 | -0.14493784582102 |
| C | -0.46375406778893 | 4.45175755844572 | -0.20901786083036 |
| C | -1.23998564351177 | 3.44960324169135 | 3.46300345991987 |
| C | -1.15300163935291 | 4.91634200351451 | -1.48913142752086 |
| C | 3.16011954677742 | 4.17000491672755 | 1.04932411074105 |
| C | 3.77381270830648 | 3.07966702915118 | 1.93387047104765 |
| C | 3.67396261283232 | 5.56549388486053 | 1.44808660898463 |
| C | -1.02788296847680 | 6.44609446168740 | -1.62156141014466 |
| C | -0.64022095528599 | 4.20360118572367 | -2.74878992451821 |
| C | -0.61342484189512 | 2.26521119793800 | 4.20940312881323 |
| C | -1.31972715386419 | 4.68253253752312 | 4.38262665772273 |
| H | 1.41852815666120 | 3.60303039731566 | 3.10842626409554 |
| H | 1.51460438386682 | 4.72954177507750 | -1.04747715292403 |
| H | -2.27542427615073 | 3.15308304912281 | 3.20101351804092 |
| H | -2.23451025050470 | 4.69017442426297 | -1.39605609185254 |
| H | 3.49659096289981 | 3.97416839471966 | 0.00870196863277 |
| H | 3.57062379823114 | 3.25477456571005 | 3.01115028616874 |
| H | 4.87268014599770 | 3.06235362823531 | 1.80346596960053 |
| H | 3.38155583392739 | 2.07909150272577 | 1.67047822511626 |
| H | 4.78304063337012 | 5.60104712066755 | 1.41519659846853 |
| H | 3.35552431830693 | 5.82227030619532 | 2.48021443743414 |
| H | 3.28334221246422 | 6.35235071720554 | 0.77187055068323 |
| H | -1.44020104130223 | 6.96314954805023 | -0.73182090334090 |
| H | -1.57174125464966 | 6.81092546217282 | -2.51752497170117 |
| H | 0.03508350896945 | 6.74978558906014 | -1.72221982841205 |
| H | 0.42448654347231 | 4.44115384348084 | -2.95027754736050 |
| H | -1.22119644015818 | 4.52380439107109 | -3.63780533561594 |
| H | -0.73424546336075 | 3.10550950846472 | -2.65200769091397 |
| H | -1.24126947503263 | 1.98527051721815 | 5.08021534819970 |
| H | 0.39512893226538 | 2.50689587921354 | 4.60246143535878 |
| H | -0.52207641114941 | 1.37482689132413 | 3.55997355085386 |
| H | -0.30351946955736 | 5.00597948965904 | 4.69059830920119 |
| H | -1.89763892683971 | 4.45486242043945 | 5.30243103502485 |
| H | -1.79782694064100 | 5.54718019004639 | 3.88002263670761 |
| H | 3.50093585550073 | 5.82356795543272 | -1.73191141346759 |
| H | -4.14685440133761 | -1.89046613760730 | 3.76353548352761 |
| H | 4.13167313403293 | 5.88331076231808 | -3.40827908156540 |
| H | -3.65598438425984 | 0.00705681003511 | 2.19304562775367 |
| H | -3.03508026312769 | -0.01418678792080 | 5.95491710992632 |
| C | 4.38379969555645 | 6.08859583139605 | -2.34729097024656 |
| H | 1.26614849399065 | 1.16191402881319 | -4.10748955651853 |
| H | 1.62049043567783 | 2.03393815106311 | -2.58698034757284 |
| H | 4.54599570034849 | 7.18146039933980 | -2.25780499419431 |
| C | 2.02138510596622 | 1.78611212834301 | -3.58797500429751 |
| H | -3.08420018304422 | 0.99806579127062 | 3.55548181866632 |
| C | -3.76771058117384 | 0.17041095454511 | 3.28052593635287 |

| | | | |
|---|---|---|---|
| C | -3.43264634994651 | -1.10029126677377 | 4.08560216332998 |
| C | -3.64700582089461 | -0.86384932780335 | 5.58622717016619 |
| H | 2.11262199554033 | 2.73225523861517 | -4.16110029701634 |
| H | -4.70665189681135 | -0.60888347823923 | 5.79008260029048 |
| H | -1.01407801047308 | -0.55751208823671 | 5.31850915270811 |
| H | 4.23084304697564 | 1.76929854350248 | -5.36520758392650 |
| H | 7.02799218741920 | 6.75993140303489 | -2.81593057195940 |
| C | 5.63546496730086 | 5.32284421837145 | -1.90306834176411 |
| H | 3.85931537400397 | 3.70244477072178 | -3.30263693874254 |
| H | 2.25135232596578 | -2.05877696520237 | 5.14006028933520 |
| C | -2.03986170342173 | -1.61910282515358 | 3.74356859050642 |
| H | 5.88444177515811 | 5.65898981786867 | -0.87162724102620 |
| C | -0.89516026409354 | -1.23544033857984 | 4.46471468748649 |
| H | 4.89397347934395 | 0.19763144572435 | -4.82314152424888 |
| H | -4.80800686433420 | 0.50296466679299 | 3.47859694522786 |
| O | 1.21447792141010 | 0.52342653374629 | 1.09036621246182 |
| C | 3.96895066765183 | 0.80513902998302 | -4.88104260078631 |
| C | 6.83781177543777 | 5.66674742954871 | -2.80320241550508 |
| H | -3.38332316246774 | -1.75706902168256 | 6.18762628665336 |
| C | 3.35817347721949 | 1.03845297618278 | -3.48736224217968 |
| H | 6.64711501408259 | 5.33894337753098 | -3.84633450951482 |
| H | 0.48735647368635 | -0.11548121417246 | -1.50160374590824 |
| C | 4.50839221209006 | 3.13186744037014 | -2.62368913632383 |
| H | 3.14315951551903 | 0.04660224183557 | -3.04338000737916 |
| C | 1.60833134038962 | -1.17974423573937 | 4.92801651374195 |
| C | 5.44732936273729 | 3.81164100919412 | -1.83213873578573 |
| O | 1.49602914732757 | -0.39100446534200 | -1.36743150945675 |
| H | 0.62398306679511 | -1.24217697926025 | 6.90009189552711 |
| C | -1.86266440786385 | -2.48947832795395 | 2.65928263525137 |
| C | 0.39637784054535 | -1.68340500145855 | 4.13368146357705 |
| H | -2.74449746978384 | -2.80807248234832 | 2.08425293857479 |
| H | 2.88169378099965 | -0.66147235255371 | 3.19695984134110 |
| H | 3.31826109408483 | 0.17139224507357 | 4.71409382942256 |
| C | 4.36167472754791 | 1.73231811396469 | -2.57424220999328 |
| P | 1.91495749222099 | -0.43152270496525 | 0.14919330800822 |
| C | 1.24855583839120 | -0.56293273264757 | 6.28576661991621 |
| C | 2.46368551566743 | -0.19387687573142 | 4.10804208630199 |
| H | 0.70021040741930 | 0.39438632887979 | 6.17095958399487 |
| H | 3.25218723853821 | 0.27393551868641 | -5.54097002593613 |
| H | 7.76018928416863 | 5.15913810528997 | -2.45734040830556 |
| H | -0.89435843849515 | -4.04752192397898 | -1.02881661886077 |
| C | -0.59898615657789 | -2.98018729327131 | 2.28811417079843 |
| C | 0.54217101606053 | -2.57944261375816 | 3.03050860003809 |
| C | 6.27516912847997 | 3.04726143064963 | -0.98850435911404 |
| O | 3.53556984488100 | -0.21964790799917 | 0.23665681414385 |
| O | 1.72935609124275 | -2.01465552413365 | 0.51141210254348 |
| H | 2.17345446441039 | -0.34082973481599 | 6.85494606837151 |
| C | 5.19796243352320 | 0.98981569051146 | -1.70499705726077 |
| H | 1.86809203715061 | 0.67916159177488 | 3.77946458010496 |
| H | 7.02385432875609 | 3.56905127165569 | -0.37090605081331 |

| | | | |
|---|---|---|---|
| C | -1.06732183402696 | -3.36378276458528 | -0.17534219167278 |
| H | -1.10117096837962 | -6.02597059993328 | 0.65873555288941 |
| C | 6.18352553602509 | 1.64732469362949 | -0.91500476381935 |
| C | 1.90715557154807 | -3.06796370178118 | 2.66610371195431 |
| C | -0.51575835844129 | -3.96451346776194 | 1.12715550039439 |
| C | 2.52538708615673 | -2.66298645756328 | 1.44377683621168 |
| C | 4.33531429149794 | -1.08457379013892 | -0.52660257744436 |
| H | -0.58045172631964 | -2.40246225808180 | -0.41866057509584 |
| C | -1.22735780227615 | -5.28463368527602 | 1.47465574366210 |
| C | 5.08218947020266 | -0.49932915775986 | -1.59380943913654 |
| H | -2.16006738725403 | -3.18558080798000 | -0.12054173374707 |
| H | -2.31739973853081 | -5.12810724633534 | 1.61463665371503 |
| C | 2.67154468479294 | -3.85075032507982 | 3.52389402732642 |
| C | 3.85424327477510 | -2.92561425848615 | 1.10946276134446 |
| H | 9.25240044266982 | 0.67720122274400 | 0.47475362121758 |
| H | 2.21527828277199 | -4.22986862849254 | 4.45116454407651 |
| H | 0.55301802013792 | -4.20723411548177 | 0.96192357246153 |
| C | 4.41742985077960 | -2.43794836366995 | -0.17992982221591 |
| C | 7.13048622782391 | 0.85419162035636 | -0.01328105517837 |
| H | -0.82580118410438 | -5.72842573575275 | 2.40783044493983 |
| C | 8.57993445498173 | 1.35836882666321 | -0.08555444359408 |
| H | 8.93990489676780 | 1.41497936718504 | -1.13251034998267 |
| C | 5.80056044248813 | -1.35649091351173 | -2.41841105579862 |
| H | 7.13524527770961 | -0.18742926947139 | -0.39506088346342 |
| C | 4.04532037322253 | -4.12779535895521 | 3.27306396490877 |
| C | 4.67157615712240 | -3.61611121000848 | 2.07736978048963 |
| H | 6.40288652339095 | -0.93025523684450 | -3.23521448750818 |
| C | 6.64241229795781 | 0.79353305660440 | 1.44539165778841 |
| C | 5.09760043263948 | -3.32343672085189 | -1.09298374487135 |
| H | 5.59585438317540 | 0.44189753526876 | 1.51732915642106 |
| C | 4.83275654326013 | -4.85614923525672 | 4.21361028841654 |
| C | 6.07764922172071 | -3.79803909825615 | 1.92096716125062 |
| C | 5.78915217015320 | -2.76740980576438 | -2.22981383357769 |
| H | 6.69063287490763 | 1.79529752011745 | 1.92054395071034 |
| H | 8.69297177838609 | 2.36630474950082 | 0.36487591423816 |
| H | 4.33854237488185 | -5.25109456879633 | 5.11488078240010 |
| H | 6.57782646809305 | -3.37228453049798 | 1.04083158766195 |
| C | 6.19143568871306 | -5.04374046394085 | 4.01440535419984 |
| C | 6.81858001435822 | -4.49371627647974 | 2.86584658166906 |
| C | 5.07862413422634 | -4.74125027811306 | -0.93740085932032 |
| H | 7.27986557794379 | 0.10738462684295 | 2.04045374978051 |
| H | 4.52451362398239 | -5.17679545627910 | -0.09457874457923 |
| C | 6.46246778414266 | -3.64372337406489 | -3.13124952405542 |
| H | 6.78779720043065 | -5.60143524673852 | 4.75209044983209 |
| H | 7.90312220788034 | -4.61516963401987 | 2.72431640310870 |
| H | 6.99503274974766 | -3.20553064783718 | -3.98959735798424 |
| C | 5.73006229600460 | -5.56722921472443 | -1.84174118608197 |
| C | 6.43852737985748 | -5.01656656648745 | -2.94215742733129 |
| H | 5.69186426816048 | -6.65875803376720 | -1.70749659842617 |
| H | 6.95682896544053 | -5.68176497522559 | -3.64908825139990 |

112
DTPA Monomer

| | | | |
|---|---|---|---|
| C | 4.16532168423123 | 0.49376485588485 | 0.95148470375306 |
| C | 3.75524143008467 | 0.24803751380287 | -0.38540452327482 |
| C | 4.92072710790202 | 1.64668445464548 | 1.22906886223834 |
| C | 3.86735326450897 | -0.48820001556233 | 2.08001734239147 |
| C | 2.91411340242230 | -0.94364619673000 | -0.70883079006360 |
| C | 4.12116197188437 | 1.15358547731744 | -1.42316417844026 |
| C | 5.28321006435924 | 2.56099835440660 | 0.22867278558226 |
| H | 5.24205651717781 | 1.84329934993069 | 2.26480496010044 |
| C | 1.56593496238742 | -1.03189977033763 | -0.23958448432324 |
| C | 3.37803930413707 | -1.98404976162907 | -1.50482415810583 |
| C | 4.87996882139560 | 2.28759377605882 | -1.08907422442963 |
| C | 3.73671741150987 | 0.92788813815902 | -2.88481409897075 |
| O | 1.13633002858601 | -0.05075939850987 | 0.64951040997818 |
| C | 0.69493050738217 | -2.07423431160429 | -0.57196834064021 |
| H | 4.42313020545351 | -1.96924902007445 | -1.84799002337424 |
| C | 2.53024499831785 | -3.04085019810499 | -1.93963232030800 |
| H | 5.15543498730799 | 2.99136969643958 | -1.88978506441569 |
| P | 0.04370740370768 | 1.10320996646793 | 0.15302850754853 |
| C | 1.15470378699210 | -3.07270783366721 | -1.50781001246369 |
| C | -0.67537715869115 | -2.12521476099559 | 0.00447694049360 |
| C | 3.00531931968226 | -4.04622691125181 | -2.83300318811562 |
| O | -1.03634428722115 | 0.14888288279260 | -0.68048028184970 |
| C | 0.29992937876991 | -4.07556736357867 | -2.05472569699912 |
| C | -1.17803202990811 | -3.30614349694389 | 0.66238307063573 |
| C | -1.52172479228774 | -1.01586251657822 | -0.09772358501028 |
| H | 4.06137203746114 | -4.01453848795656 | -3.14312069518679 |
| C | 2.15560459705238 | -5.02716521558909 | -3.31914276967098 |
| C | 0.78807380934758 | -5.02769005765764 | -2.93816388283045 |
| H | -0.76261350369905 | -4.08268190762494 | -1.77760562938449 |
| C | -0.35044950192787 | -4.42083341775941 | 0.98940960615739 |
| C | -2.57015138970881 | -3.35574895544238 | 1.03242430718692 |
| C | -2.89912079842372 | -1.02548799396783 | 0.30050402680244 |
| H | 2.53273450027730 | -5.79321987057418 | -4.01325926320044 |
| H | 0.10607377682756 | -5.78599426767724 | -3.35152057257488 |
| H | 0.72153564736099 | -4.37701518822018 | 0.75369651392721 |
| C | -0.87821576717339 | -5.54485136165962 | 1.60853499734928 |
| C | -3.08581452709645 | -4.53455055480515 | 1.64822845952936 |
| C | -3.39804842364569 | -2.22038309062913 | 0.80673965657062 |
| H | -0.21788963167510 | -6.38880099530574 | 1.85878145692678 |
| C | -2.26011395576692 | -5.61230534645191 | 1.92726533791386 |
| H | -4.15400527940131 | -4.56281669365024 | 1.91415594506979 |
| H | -4.46202202539724 | -2.28744106960652 | 1.07442823366517 |
| H | -2.66887630484794 | -6.51286692516476 | 2.40971619185169 |
| C | 6.07279592950195 | 3.81774405640546 | 0.56430766640824 |
| C | 5.24887527605577 | 5.08550738425485 | 0.28033980455632 |
| C | 7.42749288098943 | 3.85461895260571 | -0.16205091491928 |
| C | 3.14941287461565 | 2.17872261311555 | -3.55694091537279 |
| C | 4.94047662249640 | 0.40133357169073 | -3.68794431414445 |

| | | | |
|---|---|---|---|
| C | 5.14987160510533 | -1.22811724706706 | 2.50169354977003 |
| C | 3.17973770868862 | 0.18168329598821 | 3.27950358031310 |
| S | 0.57958904135190 | 2.62257036716243 | -0.89378597946267 |
| S | -0.72060971849949 | 1.42209088330986 | 2.09621893215449 |
| H | 3.17065948619131 | -1.25533491934812 | 1.68683259005311 |
| H | 2.94806167405690 | 0.14896153805982 | -2.90643199849597 |
| C | -3.72778171491163 | 0.21348831323595 | 0.18712483214996 |
| H | 6.27714853905180 | 3.78663657569270 | 1.65714053307638 |
| H | 5.02742708089493 | 5.18374485994949 | -0.80277093760963 |
| H | 5.80011691904090 | 5.99614009929542 | 0.59370705217490 |
| H | 4.27935498439914 | 5.06310069784183 | 0.81671513082708 |
| H | 8.03233978179495 | 2.95433708811577 | 0.06641778100033 |
| H | 8.01279399152936 | 4.74925488458623 | 0.13494997700770 |
| H | 7.29172946460240 | 3.89633982717628 | -1.26286546536332 |
| H | 2.28802031756632 | 2.56853706928741 | -2.98158671391990 |
| H | 2.79957927914967 | 1.93363166987144 | -4.58060855656373 |
| H | 3.89918440782198 | 2.99095568887792 | -3.65431838964449 |
| H | 4.65279992941176 | 0.18817843800090 | -4.73830887863059 |
| H | 5.35783227716437 | -0.52802057168130 | -3.25150805784755 |
| H | 5.75857024962713 | 1.15148758046289 | -3.70587919685753 |
| H | 4.92991464572188 | -1.98433763841472 | 3.28355905186210 |
| H | 5.90287339665566 | -0.52451578572404 | 2.91390271322293 |
| H | 5.61414540447296 | -1.74824277393684 | 1.63967093070221 |
| H | 2.91288585163262 | -0.57499423828692 | 4.04566346721431 |
| H | 2.25241261518829 | 0.70000471465619 | 2.96783861437393 |
| H | 3.84224277542872 | 0.92552971763303 | 3.76841105921060 |
| H | -1.37080932747181 | 2.54698561382312 | 1.68471482611857 |
| C | -4.28376837617131 | 0.82186338292094 | 1.35229197624888 |
| C | -4.97895115435899 | 2.03774861119925 | 1.21191748990203 |
| C | -5.15600817232387 | 2.66839684256972 | -0.02874041383284 |
| C | -4.63612799900336 | 2.02887024702870 | -1.16591217520740 |
| C | -3.93254239799949 | 0.81629155787090 | -1.08771524183792 |
| C | -4.20474989996113 | 0.18607624557142 | 2.73996023886174 |
| C | -3.49746359120357 | 0.14470939560264 | -2.38771674210493 |
| C | -5.88118508196123 | 4.00171230268341 | -0.13461314367664 |
| C | -7.11304882828502 | 3.91717625386344 | -1.05073843947908 |
| C | -4.92376928551541 | 5.11856630596883 | -0.58597297467545 |
| C | -4.72577115338908 | -0.43573975803795 | -3.11372079048382 |
| C | -2.68835656167637 | 1.07746055611346 | -3.30187697090122 |
| C | -3.86955590967851 | 1.17759165309389 | 3.86483047223604 |
| C | -5.52507996368655 | -0.53927988323807 | 3.06902629081418 |
| H | -5.40306265690147 | 2.51824072937089 | 2.10781177676741 |
| H | -4.78706703139305 | 2.48649274337789 | -2.15604176831453 |
| H | -3.39296971517497 | -0.56845728455062 | 2.71762452907996 |
| H | -2.84459233315864 | -0.71228033885073 | -2.13158598215950 |
| H | -6.23812596027362 | 4.25561629295552 | 0.88776296347196 |
| H | -7.81250469131815 | 3.12582426895550 | -0.71478028712153 |
| H | -6.82162969373423 | 3.68589308678597 | -2.09626552940348 |
| H | -7.66151084558552 | 4.88135804071894 | -1.06471021552264 |
| H | -4.53041086897411 | 4.91881080689603 | -1.60429011757447 |

| | | | |
|---|---|---|---|
| H | -4.05379093374756 | 5.20334674045094 | 0.09557289460393 |
| H | -5.44068244130826 | 6.10002057738253 | -0.61092986631537 |
| H | -4.42051496079664 | -0.96854609398499 | -4.03795375142708 |
| H | -5.43576590165920 | 0.36661404404756 | -3.40366167415704 |
| H | -5.27459781098113 | -1.15078434030491 | -2.46812914061151 |
| H | -1.81010090805723 | 1.49317848928395 | -2.77131229637150 |
| H | -3.30260224006688 | 1.92397974257892 | -3.67347704648722 |
| H | -2.32530117023948 | 0.52294223968733 | -4.19123850271363 |
| H | -2.93031381405834 | 1.72782008452019 | 3.66221862062645 |
| H | -3.73943028493855 | 0.63580343511573 | 4.82360310636931 |
| H | -4.67499093721527 | 1.92427511213092 | 4.02139099507508 |
| H | -5.45098548020668 | -1.07866571536668 | 4.03590553719867 |
| H | -5.80467313812388 | -1.27118734081425 | 2.28616845808810 |
| H | -6.36050935355381 | 0.18724396726713 | 3.14684850853130 |

224

DTPA Dimer

| | | | |
|---|---|---|---|
| C | -0.34173635123434 | 2.50042340084594 | -3.13841534587626 |
| C | 0.78007741113608 | 1.76837708076572 | -3.60175682778894 |
| C | -1.62082628346258 | 2.14064720494118 | -3.59127449911468 |
| C | -0.20040095831606 | 3.64426204797265 | -2.14253479937604 |
| C | 2.16184132424726 | 2.15706796197759 | -3.17665501949765 |
| C | 0.60238244866292 | 0.66604322982127 | -4.48048289289902 |
| C | -1.82668022600115 | 1.07276639047942 | -4.48158355198699 |
| H | -2.49655206772965 | 2.69548993479091 | -3.21733731435572 |
| C | 2.72539259707614 | 1.65467153268766 | -1.96316700375774 |
| C | 2.94577651017037 | 3.00356957336617 | -3.94833168556810 |
| C | -0.70188806434518 | 0.34231107825284 | -4.90171225089416 |
| C | 1.79777024690717 | -0.13224359507672 | -4.99900803093635 |
| O | 1.86102624585420 | 0.95768425936171 | -1.13505825890834 |
| C | 4.03666972061082 | 1.92263092490600 | -1.55170217662489 |
| H | 2.51691751382648 | 3.44877795513337 | -4.85898466946130 |
| C | 4.30324143580138 | 3.27838936288920 | -3.62210855103301 |
| H | -0.83676497106224 | -0.51284150140354 | -5.57725586678430 |
| P | 2.08054566767138 | -0.62148766232627 | -0.65128790953687 |
| C | 4.88148552507647 | 2.69176229246477 | -2.43782335484238 |
| C | 4.59536497653483 | 1.35149756325574 | -0.29500615674395 |
| C | 5.11681092475185 | 4.07888268152453 | -4.47715424974135 |
| O | 3.71713443329476 | -0.79996000606066 | -0.90207601752076 |
| C | 6.27988233430709 | 2.87463965433734 | -2.21653871683782 |
| C | 5.29027508654684 | 2.14590528957488 | 0.68651639524968 |
| C | 4.53630715366619 | -0.02869517061931 | -0.08238316167569 |
| H | 4.65328829420438 | 4.52919943336224 | -5.36874047215765 |
| C | 6.46348164278438 | 4.26760020450067 | -4.21026661381695 |
| C | 7.04954534176694 | 3.64184164009676 | -3.07943810957285 |
| H | 6.75553231763326 | 2.38970960893546 | -1.35428382168533 |
| C | 5.25192928817941 | 3.57096265179313 | 0.68325405306198 |
| C | 6.02106741803158 | 1.47766146828685 | 1.73577736448648 |
| C | 5.31059286264013 | -0.72470973757185 | 0.89861377398433 |
| H | 7.08201714194562 | 4.88145754257142 | -4.88211634340544 |
| H | 8.12658874524265 | 3.75918123143683 | -2.88639771257586 |

| | | | |
|---|---|---|---|
| H | 4.67312868179904 | 4.08402484116696 | -0.09711174615614 |
| C | 5.90920630127351 | 4.30458602441965 | 1.66059167506077 |
| C | 6.70210215011325 | 2.26267725562527 | 2.71236325366614 |
| C | 6.05658003494321 | 0.05265889130862 | 1.77626622002991 |
| H | 5.85163724982870 | 5.40336868559438 | 1.64856591717301 |
| C | 6.64844213200154 | 3.64782091560018 | 2.67946204726507 |
| H | 7.26025923403818 | 1.74303193246770 | 3.50635193347316 |
| H | 6.68706357778374 | -0.44304045486577 | 2.52974011721360 |
| H | 7.17072890686000 | 4.23958780458777 | 3.44617780988127 |
| C | -3.24311264495035 | 0.76603413428913 | -4.95237434477332 |
| C | -3.39791901983784 | -0.60121540589015 | -5.62162597149187 |
| C | -3.75999597401950 | 1.88556812495533 | -5.87493241965871 |
| C | 1.49389352385523 | -1.61762447100280 | -5.23932515914890 |
| C | 2.36593500930940 | 0.49975860232155 | -6.28430520346990 |
| C | -0.67006227223324 | 4.97863515778994 | -2.74565569482869 |
| C | -0.94647366465956 | 3.32296107695645 | -0.83994858908715 |
| S | 1.13060791855799 | -2.02171181522138 | -1.60614002967590 |
| S | 1.76813684813697 | -0.35900436335002 | 1.39804944588499 |
| H | 0.87679074239758 | 3.75014336622305 | -1.89778720203766 |
| H | 2.58750017523713 | -0.07920636595718 | -4.21868142960177 |
| C | 5.37125934405426 | -2.21916712914869 | 0.87113601121194 |
| H | -3.88125651029088 | 0.77116842537526 | -4.04011062935907 |
| H | -2.84072229574505 | -0.65178335303881 | -6.58017882770176 |
| H | -4.46393896408441 | -0.80003746516367 | -5.85070473141352 |
| H | -3.03153885158008 | -1.41991504304230 | -4.97118191361262 |
| H | -3.70896849731887 | 2.87564783050358 | -5.38085671565511 |
| H | -4.81654669083714 | 1.70453857068189 | -6.16222524442678 |
| H | -3.15374366149953 | 1.93653368836198 | -6.80337315545333 |
| H | 1.03210650736036 | -2.08573859856177 | -4.34915583511725 |
| H | 2.43182135864472 | -2.16501073555463 | -5.46332302549808 |
| H | 0.81702444869320 | -1.76552409037320 | -6.10629607995965 |
| H | 3.24517036873357 | -0.07212237832449 | -6.64620065455157 |
| H | 2.68432149333470 | 1.54715940908168 | -6.12460586427890 |
| H | 1.59977105208672 | 0.49631924267097 | -7.08737914957806 |
| H | -0.52186164141206 | 5.81005448698280 | -2.02573897050557 |
| H | -1.75013409620533 | 4.94394855554197 | -3.00043033107365 |
| H | -0.11460265715145 | 5.22282221391226 | -3.67378552745996 |
| H | -0.84676792974429 | 4.15339350896882 | -0.11241426259166 |
| H | -0.56442155911548 | 2.39694874157304 | -0.37194009254024 |
| H | -2.02651155696559 | 3.17448192071600 | -1.03106070752684 |
| H | 0.68467686111826 | -1.23591425072291 | 1.39573320966035 |
| C | 4.88382992955753 | -3.00400191038994 | 1.95084295931046 |
| C | 4.99268261475218 | -4.40563747791237 | 1.87371516813066 |
| C | 5.55343635413987 | -5.05788090811449 | 0.76703340350687 |
| C | 6.02132542313091 | -4.26134792517645 | -0.29154769444995 |
| C | 5.94372572729055 | -2.85999657100349 | -0.26729226601412 |
| C | 4.24272317264395 | -2.39136047559804 | 3.19146377514762 |
| C | 6.51593545128183 | -2.08636777803197 | -1.45366346194182 |
| C | 5.62687314121197 | -6.57645246289722 | 0.70455343522601 |
| C | 7.06902705708323 | -7.07647519062161 | 0.51996244286248 |

| | | | |
|---|---|---|---|
| C | 4.69710400079394 | -7.13203873849516 | -0.38833753518421 |
| C | 8.04155313775859 | -2.26530469814845 | -1.54504483263376 |
| C | 5.81770129247503 | -2.45791077420444 | -2.77299947327204 |
| C | 2.88489096533485 | -3.03400372054361 | 3.52232557854193 |
| C | 5.19621998093068 | -2.47217047297578 | 4.39695716765150 |
| H | 4.61339310915943 | -5.01726760792496 | 2.70795542133787 |
| H | 6.46575922277006 | -4.74845489855302 | -1.17398703804814 |
| H | 4.05216496621407 | -1.32223648458453 | 2.97067225237529 |
| H | 6.33609947338360 | -1.00662354497383 | -1.28321170915504 |
| H | 5.26031074582719 | -6.95617696251821 | 1.68351700976065 |
| H | 7.10838057122674 | -8.18511163373935 | 0.53686343286369 |
| H | 7.73471253192435 | -6.69462201903547 | 1.31969933997508 |
| H | 7.48836638221674 | -6.74450424446242 | -0.45253539861884 |
| H | 4.70967423458523 | -8.24160251112994 | -0.39456261825976 |
| H | 5.01405236746004 | -6.78619064210883 | -1.39421159273581 |
| H | 3.65179673854769 | -6.79744647396857 | -0.23530647975999 |
| H | 8.45810734488520 | -1.66175993138689 | -2.37784154747987 |
| H | 8.31556230778729 | -3.32515463488558 | -1.72796959484774 |
| H | 8.53943069378065 | -1.94819188822706 | -0.60654510101858 |
| H | 4.72186051880927 | -2.32371154555509 | -2.69153069020901 |
| H | 6.01580469568272 | -3.51279075930657 | -3.05584103965682 |
| H | 6.18626845467074 | -1.81746450271342 | -3.60070216175965 |
| H | 2.22491666951909 | -3.05678200857694 | 2.63371285509772 |
| H | 2.36505920593871 | -2.45835860559645 | 4.31564811761949 |
| H | 2.99778643568141 | -4.07489137093030 | 3.88947836502246 |
| H | 6.14752270065818 | -1.93723734469076 | 4.20364881743440 |
| H | 5.45086337304885 | -3.52694149348758 | 4.62944815752432 |
| H | 4.73326162651314 | -2.02893556593634 | 5.30200226446576 |
| H | -5.42275244494557 | -8.23316684896279 | 0.96445992100865 |
| H | 3.00096916640794 | -0.61370444816196 | 5.66137988582048 |
| H | -4.65872835434870 | -6.69353551911430 | 1.49060334770151 |
| H | 3.70318191616931 | 0.60993500192794 | 7.73092233292369 |
| H | 5.16954710865079 | 0.55491230033306 | 5.60610122144498 |
| C | -5.22675655868678 | -7.18050154078181 | 0.67320772032083 |
| H | -2.66597673468531 | -3.38969419887600 | -3.67620830486718 |
| H | -2.62675058647616 | -3.47135786062282 | -1.88616608035901 |
| H | -4.57315587588613 | -7.19420855444858 | -0.22383191994664 |
| C | -3.24386361335970 | -3.69389771251509 | -2.77870054615975 |
| H | 1.91331460261159 | 0.57823058372873 | 7.57715007868633 |
| C | 2.84672719047924 | 0.96111038134571 | 7.11865093929864 |
| C | 2.98666612969277 | 0.49930940463478 | 5.65534002999757 |
| C | 4.30905581618193 | 0.98118287774521 | 5.05196059434196 |
| H | -3.38503048786681 | -4.79316986822933 | -2.83321938101881 |
| H | 4.40567735356260 | 0.68466154819344 | 3.98966649034649 |
| H | 0.57968341260733 | -0.74638751919812 | 5.59022143405327 |
| H | -6.39742921356393 | -2.66600206578688 | -3.94192651848891 |
| H | -6.79267316169744 | -7.17103888189214 | -1.66986301863067 |
| C | -6.54133050853973 | -6.43337093242761 | 0.38647235687547 |
| H | -5.32274699836888 | -5.36835453725155 | -1.83802662434567 |
| H | -2.50759278005978 | -0.19900846919436 | 3.64503550821455 |

| | | | |
|---|---|---|---|
| C | 1.75765605325295 | 0.91243142271498 | 4.85663547419885 |
| H | -7.14810286930256 | -6.45165763240071 | 1.31841665409188 |
| C | 0.56991968358959 | 0.16266700877222 | 4.96926620666845 |
| H | -4.92070957504435 | -3.05194089763766 | -4.88654574307728 |
| H | 2.81667835608507 | 2.06928120336653 | 7.17419033905987 |
| S | -1.22721570850553 | -2.37221098648059 | 1.32735683541558 |
| C | -5.46390527555047 | -3.26532181311996 | -3.94429466329409 |
| C | -7.35522694082546 | -7.13242851113271 | -0.71392905547965 |
| H | 4.40603309979674 | 2.08519674837729 | 5.09978285722284 |
| C | -4.58831286101701 | -2.94846475509519 | -2.71993880261779 |
| H | -8.31031569604271 | -6.60451937979533 | -0.90790827808945 |
| H | -0.83607677062935 | -1.07027314650537 | -1.54593469372285 |
| C | -5.62651714044963 | -4.58987533914805 | -1.12059431883342 |
| H | -4.35632303290418 | -1.86467383376418 | -2.73536781164759 |
| C | -1.91431397836138 | -0.23751363255439 | 4.58254781092876 |
| C | -6.27369154481991 | -4.97023073692417 | 0.06532889625338 |
| S | -2.10341847313549 | -0.48374343157167 | -1.46419322458698 |
| H | -3.71753190075321 | -0.11018066099304 | 5.82445545865300 |
| C | 1.73694224198765 | 2.05487158283436 | 4.04618500742036 |
| C | -0.62562812011232 | 0.54754265098070 | 4.34148515775332 |
| H | 2.65696797415293 | 2.64227316873444 | 3.91320229434253 |
| H | -1.24912284063323 | -1.85783550785680 | 5.91862073101388 |
| H | -1.03894570347455 | -2.20769844415717 | 4.16448211111495 |
| C | -5.33168772639259 | -3.24849130493442 | -1.42219025823696 |
| P | -2.17316951465957 | -0.83877924282584 | 0.60259567742140 |
| C | -2.75848410760502 | 0.43049985475979 | 5.68576002202325 |
| C | -1.69375073970983 | -1.71897934491918 | 4.91125678456022 |
| H | -2.99493092147132 | 1.48457609204889 | 5.44418828031308 |
| H | -5.75875342900403 | -4.33508458270594 | -3.96104748298034 |
| H | -7.58991545339553 | -8.17801380109346 | -0.42684614732945 |
| H | 1.68456566782932 | 4.40418470838611 | 0.68839816858273 |
| C | 0.57001742591702 | 2.47309544518538 | 3.37761491342031 |
| C | -0.62349636670852 | 1.72973908414010 | 3.54502884022814 |
| C | -6.63170742147627 | -3.95585636171451 | 0.96604927147684 |
| O | -3.79336846525361 | -0.84313661822384 | 0.99936726650922 |
| O | -1.69239350969908 | 0.61532785003933 | 1.24550506350257 |
| H | -2.21228167898288 | 0.41625947606629 | 6.65177690135425 |
| C | -5.71019758516003 | -2.23790145812311 | -0.49603943858946 |
| H | -2.66541699491826 | -2.25288500044280 | 4.90697669929458 |
| H | -7.14044087045584 | -4.23800239652356 | 1.90233841223517 |
| C | 1.72528200317979 | 3.55896474059789 | 1.40662226130789 |
| H | 0.01238875907260 | 5.12827291154429 | 4.04075011687217 |
| C | -6.36245913127265 | -2.59889536386041 | 0.71824493132852 |
| C | -1.90811966448159 | 2.21865168749479 | 2.95254133529228 |
| C | 0.63557506508669 | 3.70379213920144 | 2.48126561494794 |
| C | -2.49784597107857 | 1.58841729411266 | 1.80932933241834 |
| C | -4.55733283553417 | -0.08140510209101 | 0.11551531578187 |
| H | 1.60568199403327 | 2.61677322122194 | 0.83871980112743 |
| C | 0.82439363675369 | 4.99276326916156 | 3.29801490663436 |
| C | -5.43113030494905 | -0.79395403685685 | -0.76044537216170 |

| | | | |
|---|---|---|---|
| H | 2.74031948289945 | 3.55505233129561 | 1.85288211974016 |
| H | 0.83299275175651 | 5.88162204222434 | 2.63355808981413 |
| C | -2.58172760983868 | 3.28299676791398 | 3.53350120731022 |
| C | -3.74401418680937 | 1.96987583074493 | 1.28603270756202 |
| H | -8.65447975385779 | -0.76739935828581 | 2.62085396691168 |
| H | -2.12764664741246 | 3.79131372899603 | 4.39800451664748 |
| H | -0.33858597530793 | 3.78202360658818 | 1.95885874770114 |
| C | -4.38942571852161 | 1.30565257200604 | 0.11614284445635 |
| C | -6.80765366920323 | -1.57296125935697 | 1.75884824056659 |
| H | 1.78573493564363 | 4.97883305786199 | 3.85276889746999 |
| C | -8.33953231358829 | -1.55040081289434 | 1.90057179081952 |
| H | -8.83008687575114 | -1.34217924622499 | 0.92843002676792 |
| C | -6.02304980788309 | -0.06394796854430 | -1.78318867023502 |
| H | -6.50879691673468 | -0.56694798732135 | 1.40319578835168 |
| C | -3.86959680409952 | 3.68986053949680 | 3.09203950423390 |
| C | -4.48962876035278 | 2.99257850560210 | 1.99369029588675 |
| H | -6.71089206969061 | -0.56969247252133 | -2.47782361444032 |
| C | -6.11592520525454 | -1.79951563960960 | 3.11540272368039 |
| C | -4.93851526074419 | 2.03173522469833 | -1.00327521305525 |
| H | -6.38519558118597 | -0.99349802080637 | 3.82890615950949 |
| C | -4.58040615474914 | 4.72880850158099 | 3.76175329036944 |
| C | -5.84389794603444 | 3.32279757749596 | 1.68223701361097 |
| C | -5.74284038989668 | 1.32199985788012 | -1.97080367509228 |
| H | -5.01455226754743 | -1.80903215979395 | 3.00128529174253 |
| H | -8.72728728288081 | -2.52206942268099 | 2.27077869767791 |
| H | -4.07854739059344 | 5.25656020453003 | 4.58768311615149 |
| H | -6.36656349399898 | 2.76528646534969 | 0.89477599667096 |
| C | -5.87868647329949 | 5.05254563170970 | 3.40209551648841 |
| C | -6.51842356953823 | 4.32462755135146 | 2.36610849683812 |
| C | -4.67593881300073 | 3.41579858580885 | -1.22481316320277 |
| H | -6.42378039022072 | -2.76423935927928 | 3.56928305403127 |
| H | -4.06324431639598 | 3.96398686868753 | -0.49688596307472 |
| C | -6.25337865721482 | 2.02947772501194 | -3.09937808345475 |
| H | -6.41961867115580 | 5.85208475760478 | 3.93004384503439 |
| H | -7.56363616240567 | 4.54878415843070 | 2.10461826139093 |
| H | -6.86780898616452 | 1.47885048840995 | -3.82836482965859 |
| C | -5.16741157309590 | 4.06727154604513 | -2.34763765282241 |
| C | -5.96851814004227 | 3.37259907233048 | -3.29145369523335 |
| H | -4.93242268619490 | 5.13065730349927 | -2.50577147698442 |
| H | -6.35604329218542 | 3.89899542680272 | -4.17635345502486 |

72

DSI Monomer

| | | | |
|---|---|---|---|
| C | 5.33459797437620 | -0.50065913279362 | 0.22180373730871 |
| C | 4.03295662986088 | -0.18332771069204 | -0.20185699864197 |
| C | 6.30715219653385 | 0.50583146820276 | 0.32641496795632 |
| C | 3.00155443078487 | -1.24174859795970 | -0.38530863510036 |
| C | 3.72020366556639 | 1.14711374148385 | -0.54048205620744 |
| C | 5.98997065203110 | 1.83517447470694 | 0.01067752730638 |
| C | 7.71795923407456 | 0.14456452745480 | 0.73091580619146 |
| C | 1.73104836129718 | -1.23295570704588 | 0.27090613213749 |

| | | | |
|---|---|---|---|
| C | 3.22670290995120 | -2.23828850813493 | -1.32911891634023 |
| C | 4.69464130161694 | 2.14785385838991 | -0.42651432322062 |
| S | 1.38704582321017 | -0.09327599033522 | 1.62877789374141 |
| C | 0.67636594650028 | -2.06787197027403 | -0.10924898262798 |
| H | 4.20136591874787 | -2.27448728486800 | -1.83952292366964 |
| C | 2.21246596475858 | -3.15206205172812 | -1.72195055866673 |
| C | 4.31106726712168 | 3.57486075622780 | -0.74939154957727 |
| N | 0.43251682360902 | 1.05977945542343 | 0.72888279033474 |
| C | 0.89487437557421 | -3.03555344414675 | -1.14891843702103 |
| C | -0.69650267387192 | -1.95708145098879 | 0.47958037302293 |
| C | 2.45169940982030 | -4.14002933027759 | -2.72182849137541 |
| S | -0.73320245833980 | 0.62312290401878 | -0.49324953120116 |
| C | -0.14131619769962 | -3.88398633806165 | -1.64164306646583 |
| C | -1.18838334682310 | -3.01812851968565 | 1.31177849770218 |
| C | -1.52573938128676 | -0.86121253932689 | 0.20015464604537 |
| H | 3.46267659082301 | -4.22332498446972 | -3.14886989470111 |
| C | 1.42932680916716 | -4.96629473546691 | -3.15969130957297 |
| C | 0.12181477987722 | -4.82807422461842 | -2.62211205566486 |
| H | -1.15724266862249 | -3.78315460592230 | -1.23698693134725 |
| C | -0.36528888166539 | -4.11043069299875 | 1.71316912263905 |
| C | -2.53967190523048 | -2.95126146424482 | 1.80185459491100 |
| C | -2.89569789144772 | -0.81654200170357 | 0.62061871610422 |
| H | 1.62452808185251 | -5.72242140309356 | -3.93474773885999 |
| H | -0.69022411881326 | -5.47269515973095 | -2.99000318745522 |
| H | 0.67706070923364 | -4.15026652158716 | 1.36845398407592 |
| C | -0.86266445732429 | -5.10048455811396 | 2.54650456748648 |
| C | -3.02558099049015 | -3.99153996661972 | 2.64676908899090 |
| C | -3.36331870992371 | -1.86154068832089 | 1.41347165245332 |
| H | -0.21163173555855 | -5.93160414151435 | 2.85578649705976 |
| C | -2.20398655346084 | -5.04632434407632 | 3.01150480080056 |
| H | -4.06310266881307 | -3.93601195716450 | 3.00996802823222 |
| H | -4.42034286310659 | -1.85993356570909 | 1.72110789835108 |
| H | -2.58613217080190 | -5.84109249758320 | 3.66944869619734 |
| H | 0.01274422260912 | 1.75582980159494 | 1.36664889424608 |
| C | -3.87083794087719 | 0.21214884047202 | 0.16689463594761 |
| C | -4.06086501470463 | 0.45718999303805 | -1.20969210940562 |
| C | -5.03380011103677 | 1.36499485343528 | -1.63879252811382 |
| C | -5.82859820098009 | 2.05298744195728 | -0.70527996282126 |
| C | -5.64625135915140 | 1.80909987441325 | 0.66023907687288 |
| C | -4.67640391923868 | 0.88954026965381 | 1.09673208026100 |
| C | -5.27785529154733 | 1.57668496161229 | -3.11634093887975 |
| C | -6.48607733390768 | 2.52153510028287 | 1.69603302321610 |
| O | 2.57663437390162 | 0.63824244510191 | 2.08604404811937 |
| O | -1.64397657821739 | 1.77957281660376 | -0.47147657291230 |
| O | 0.54524387439029 | -0.78664056031943 | 2.62409912896795 |
| O | 0.01147422782740 | 0.24641643892396 | -1.70469713329701 |
| H | 5.58622168422642 | -1.53521180583913 | 0.49556012026374 |
| H | 2.70690660532853 | 1.39561630638673 | -0.88973644266718 |
| H | 6.74732988681773 | 2.62450465386977 | 0.10455480795255 |
| H | -3.43957457134988 | -0.06434795321842 | -1.95170933552053 |

| | | | |
|---|---|---|---|
| H | -6.58084962700352 | 2.77887738118659 | -1.04151332286734 |
| H | -4.52883400166283 | 0.71725005176647 | 2.17306878217686 |
| F | -4.20542952892391 | 1.22826857000335 | -3.86128709220531 |
| F | -5.57300078064312 | 2.86843406904620 | -3.38983791892662 |
| F | -6.32654123967493 | 0.82951007745581 | -3.54648767411458 |
| F | -5.70966035253092 | 3.17894636081321 | 2.59059400699987 |
| F | -7.23708518484774 | 1.63866411359133 | 2.40225631776837 |
| F | -7.32810493760768 | 3.42146357701362 | 1.14292264790977 |
| F | 8.46841459831391 | -0.18415846544244 | -0.35187382163079 |
| F | 7.74191082767855 | -0.92113889546312 | 1.56633420513062 |
| F | 8.34251535795043 | 1.17104194985072 | 1.35135244635131 |
| F | 5.38723007873667 | 4.39458516444226 | -0.78591364484408 |
| F | 3.45311151188367 | 4.07134759685875 | 0.17385603152607 |
| F | 3.69077568673215 | 3.65618771735714 | -1.95047869213576 |

144

DSI Dimer

| | | | |
|---|---|---|---|
| C | -5.74289766192854 | 1.43610565559275 | -2.65499538163474 |
| C | -4.95133312881515 | 1.28954159073167 | -1.50067855969141 |
| C | -6.62604734717415 | 0.41679008800690 | -3.04002781683773 |
| C | -3.96536666085092 | 2.33529165339691 | -1.10640486975287 |
| C | -5.09394740680093 | 0.12479933561976 | -0.72316284641772 |
| C | -6.71244505070327 | -0.77333912823855 | -2.29944247318723 |
| C | -7.51963357329939 | 0.60323345053721 | -4.24560785978317 |
| C | -2.57550381280577 | 2.02685235329315 | -0.89677572521314 |
| C | -4.39347858436405 | 3.63381889373930 | -0.87827032956500 |
| C | -5.94029272765733 | -0.91143217537251 | -1.13775783901341 |
| S | -2.06339347862346 | 0.39848562371232 | -1.52532923842713 |
| C | -1.67086515722147 | 2.95728002065827 | -0.37198302508061 |
| H | -5.45781483084700 | 3.87888450058413 | -1.01535886712838 |
| C | -3.50143544788940 | 4.65157047517433 | -0.44874465088397 |
| C | -5.93602703988309 | -2.21483952213345 | -0.37299824351278 |
| N | -0.39193168123885 | 0.46402716253092 | -1.77190334578483 |
| C | -2.12684335656879 | 4.31595930353096 | -0.17873548068981 |
| C | -0.25812532390561 | 2.69445864947433 | 0.08046986847193 |
| C | -3.94708484706665 | 5.99213499617902 | -0.26070421568441 |
| S | 0.41508651395523 | 1.85379254919248 | -2.41837186921931 |
| C | -1.25137095826118 | 5.35861319932503 | 0.25138785402512 |
| C | 0.00845289682430 | 2.68412534469734 | 1.49255951220134 |
| C | 0.82285647990963 | 2.56292916041185 | -0.79891328986798 |
| H | -5.00270907239223 | 6.22806866489025 | -0.46442899795978 |
| C | -3.07111735603482 | 6.97852064861151 | 0.16262814284407 |
| C | -1.71158789031326 | 6.65688250524453 | 0.41229041514121 |
| H | -0.19505308075165 | 5.13023223638690 | 0.44473970532536 |
| C | -1.03126233107491 | 2.75724427246925 | 2.46551675169682 |
| C | 1.37203017516945 | 2.54298724019360 | 1.94800801304263 |
| C | 2.18628106312504 | 2.56507772877454 | -0.36778088828929 |
| H | -3.42500923664383 | 8.01105343531592 | 0.30074799714757 |
| H | -1.01444016639615 | 7.44427526195488 | 0.73531601396891 |
| H | -2.06990997587532 | 2.86924832329529 | 2.13200514577476 |
| C | -0.74521804571178 | 2.65591029313131 | 3.81722046047215 |

| | | | |
|---|---|---|---|
| C | 1.63198635212358 | 2.44540306500930 | 3.34614102042315 |
| C | 2.43147469173838 | 2.54227074891368 | 1.00175668778326 |
| H | -1.56888705236938 | 2.68475663255003 | 4.54441475173072 |
| C | 0.59316598982399 | 2.49339599366080 | 4.26160794078448 |
| H | 2.67194066357653 | 2.31668048350994 | 3.67932080489816 |
| H | 3.47027806868452 | 2.54550394612885 | 1.36239408686463 |
| H | 0.80343162451933 | 2.40347857665369 | 5.33781572957076 |
| H | 0.09828504563328 | 0.06101998248108 | -0.94137834832919 |
| C | 3.30203902106095 | 2.68570130896761 | -1.34031304490487 |
| C | 3.24561326733040 | 3.68083893432098 | -2.33864993690035 |
| C | 4.28351294917503 | 3.81753797213729 | -3.26697432597787 |
| C | 5.38738767040211 | 2.95069458602002 | -3.22971847408340 |
| C | 5.44881414686110 | 1.96489825713395 | -2.23632537625976 |
| C | 4.42310059117593 | 1.84030354875807 | -1.28443072862188 |
| C | 4.23800787212088 | 4.93656891408278 | -4.28305009399737 |
| C | 6.59452975248055 | 0.98106352306867 | -2.20318809767108 |
| O | -2.70493708752141 | 0.22744952072740 | -2.83649053986250 |
| O | 1.62947582330044 | 1.33190674882331 | -3.06352987592886 |
| O | -2.21478142474764 | -0.63084734310306 | -0.46254102348995 |
| O | -0.57611335456701 | 2.64745233265815 | -3.15830048748116 |
| H | -5.63740943083007 | 2.33331694397110 | -3.28073720729592 |
| H | -4.52182362343136 | 0.01301717371235 | 0.20211825646983 |
| H | -7.37722563613316 | -1.58410709228850 | -2.62725363787641 |
| H | 2.37244893387969 | 4.34738427651502 | -2.39635079615744 |
| H | 6.19103564347832 | 3.04102724716431 | -3.97217806866695 |
| H | 4.48126695879069 | 1.05811157587209 | -0.51505284705909 |
| F | 2.96869799452460 | 5.24058978945128 | -4.63660449012319 |
| F | 4.91832969788576 | 4.61905237925229 | -5.40878973593116 |
| F | 4.79561327978852 | 6.06905455746188 | -3.78467938200292 |
| F | 7.57962863473951 | 1.30290001487078 | -3.06558455900046 |
| F | 6.16735158010586 | -0.27286202470690 | -2.53047737458692 |
| F | 7.13965952295848 | 0.90018055489835 | -0.96550876552116 |
| F | -7.72208583030650 | -0.56359825210406 | -4.90172872303648 |
| F | -7.00344966969614 | 1.48938829204221 | -5.12687905501513 |
| F | -8.74042584232850 | 1.06834262610375 | -3.87744177277617 |
| F | -5.70679328887059 | -2.02496708663307 | 0.95192765537169 |
| F | -4.95790033655725 | -3.04569040157707 | -0.82320829383935 |
| F | -7.10686203878526 | -2.88087300141455 | -0.49481802649456 |
| C | 5.61616831672685 | -2.43186741813533 | -0.31964867280227 |
| C | 4.38290062077296 | -1.91132501142628 | 0.10278318474296 |
| C | 6.80788395828620 | -2.01738825770460 | 0.29624986084738 |
| C | 3.11084865409222 | -2.28886881912213 | -0.57688859147549 |
| C | 4.35735447192012 | -0.95152052534975 | 1.13501617710210 |
| C | 6.78180622629897 | -1.08278640237761 | 1.33810268471158 |
| C | 8.12893338032069 | -2.52412787014879 | -0.23537474412074 |
| C | 2.00573485634054 | -2.90169054099163 | 0.09318126479738 |
| C | 2.93011399059438 | -1.91916168696450 | -1.90604603753741 |
| C | 5.55081306064420 | -0.54689960921566 | 1.74720519541708 |
| S | 2.15288979480599 | -3.45309652336736 | 1.80057168924883 |
| C | 0.73360912350578 | -3.00596745102134 | -0.48357090286339 |

| | | | |
|---|---|---|---|
| H | 3.75827359693814 | -1.42510408994933 | -2.43670044237277 |
| C | 1.68503717353180 | -2.07806496349389 | -2.57323993455917 |
| C | 5.51372277598335 | 0.52777756486581 | 2.80458032700398 |
| N | 1.44708894664004 | -2.02282856377655 | 2.56621334216130 |
| C | 0.55007061111420 | -2.59716590983250 | -1.84945357168371 |
| C | -0.47937605944256 | -3.38418614613715 | 0.30678877254105 |
| C | 1.51375780170391 | -1.64942355064364 | -3.92059825943497 |
| S | 0.09049026615622 | -1.19525253111491 | 1.88010324909862 |
| C | -0.71987586102617 | -2.63406587452001 | -2.50107317873034 |
| C | -1.18770787831130 | -4.58782678255778 | -0.01530074608579 |
| C | -0.98493471243177 | -2.53485824225228 | 1.30289589430439 |
| H | 2.37987543460547 | -1.23255373619791 | -4.45429468431756 |
| C | 0.27268165735465 | -1.72145451645282 | -4.53101533124085 |
| C | -0.85010938567000 | -2.20839618958791 | -3.81299405136878 |
| H | -1.60796199280344 | -2.95900177819563 | -1.94537952959528 |
| C | -0.68142831208856 | -5.53601160976261 | -0.95173876649185 |
| C | -2.43868157881159 | -4.85786669810714 | 0.63883741579020 |
| C | -2.22476775266394 | -2.79393289088008 | 1.97119132374284 |
| H | 0.14752200234018 | -1.37061640724465 | -5.56602429720253 |
| H | -1.84150262666419 | -2.21301437251264 | -4.28730832221915 |
| H | 0.29210411496874 | -5.35118484564143 | -1.42644233973329 |
| C | -1.40191343684861 | -6.68081577815993 | -1.25398946064280 |
| C | -3.16580895817494 | -6.03357256073523 | 0.29240112052899 |
| C | -2.92395556337269 | -3.94145988440948 | 1.60641195410645 |
| H | -0.99736938997548 | -7.40639403886035 | -1.97520787038406 |
| C | -2.65926504519153 | -6.92605943898341 | -0.63854477849738 |
| H | -4.13340013345086 | -6.22019138590376 | 0.78224465483849 |
| H | -3.90707046776701 | -4.12482057820875 | 2.06448678381307 |
| H | -3.22572269653676 | -7.83284642308087 | -0.89819839686706 |
| H | 1.25307230978462 | -2.21673112581357 | 3.56288474782372 |
| C | -2.88077015468178 | -1.90525031624952 | 2.97400108252988 |
| C | -3.29250119817672 | -0.59895824685998 | 2.63491081319416 |
| C | -4.04128508270427 | 0.15756260899716 | 3.54176972526213 |
| C | -4.36439519733561 | -0.35489474832670 | 4.81201210322191 |
| C | -3.94075750358526 | -1.64175404148707 | 5.15620707324064 |
| C | -3.21473756490419 | -2.42012209202647 | 4.23646565984167 |
| C | -4.54680743754526 | 1.53427793256447 | 3.18792805658588 |
| C | -4.22872225506605 | -2.20679434465484 | 6.52830222319983 |
| O | 3.54254336465873 | -3.50426666531676 | 2.27462155375508 |
| O | 0.63259506148082 | -0.42471718172617 | 0.73378922125217 |
| O | 1.27137186235689 | -4.61429983991580 | 2.02465504946253 |
| O | -0.49021354530848 | -0.48666970838452 | 3.03114686715398 |
| H | 5.64732491041197 | -3.17470867952317 | -1.12914638636947 |
| H | 3.39507350576146 | -0.53360134209340 | 1.46490408606366 |
| H | 7.71541386788838 | -0.76656396838100 | 1.82127888957149 |
| H | -3.02381381506630 | -0.20005843633941 | 1.64763919826462 |
| H | -4.95357063378211 | 0.23956336724770 | 5.52369260684766 |
| H | -2.90511953535266 | -3.43996749096108 | 4.50789414614365 |
| F | -4.03425979603141 | 2.48577334574201 | 4.01529228671921 |
| F | -5.89173319768942 | 1.61405770051918 | 3.29282666145806 |

F -4.21917825361376 1.89644368772901 1.91841501730561
F -5.18518654095439 -1.50475877415130 7.17701618276771
F -3.11802493940377 -2.19277681370543 7.30611669630474
F -4.64170104733634 -3.49513069663460 6.45320329217831
F 8.50311171994747 -1.83520142988330 -1.34327720534016
F 8.05660527503768 -3.82941310625072 -0.58790305609163
F 9.12290992832395 -2.39470594219198 0.67250292802472
F 5.51209553184469 1.77524928341372 2.23980265461916
F 6.57799641509068 0.47682703735941 3.62968816577636
F 4.39244700533748 0.45459401845998 3.56550801057029
186
IDP Monomer
C 1.82755822200217 5.38657666798445 1.97778741665997
C 1.95413398896306 2.86405862219747 -2.85171717957664
C 2.20914292008962 4.03962155841402 2.09941681799098
C 0.45555801242526 5.68587734669454 2.02556348002779
C 2.93642092056987 1.83281684875312 -2.87166767783245
C 1.23151678882398 3.11383416015969 -4.02748607167681
C 1.68421660812497 3.65428686476113 -1.58490802401261
C 1.26697517409223 3.01315226157436 2.28811054256823
H 3.27812954450842 3.76970571150650 2.05444486853057
C -0.52611192556292 4.69108797385479 2.19776736576846
H 0.13271190366483 6.73673468985831 1.93431763351952
C 3.64404784858360 1.46408916824902 -1.61042991240396
C 3.21907381629382 1.12575411762839 -4.06746955975907
C 1.46662194970734 2.40227365710895 -5.21765827912445
H 0.44064680772762 3.87682784974984 -4.01874488131091
C -0.11310551530969 3.34056210486383 2.34097011112578
C 1.76353911943785 1.59541598694390 2.44765001703389
C -1.98523271919751 5.10191334029756 2.21921011600191
C 3.36017798234905 0.21682427857939 -0.97597921257450
C 4.58091361035420 2.28440690510794 -0.99520904436430
C 2.47610891320738 1.42857680823145 -5.22457127459509
C 4.30860595840530 0.07150078766802 -4.14858954250388
C -1.12794627215490 2.27564942078882 2.62144001925610
O 2.36076131581944 -0.58489318907941 -1.55114688782853
C 4.00677641843027 -0.24960752755125 0.16947357775019
H 4.83821699444552 3.24726452718455 -1.46153408672325
C 5.19497277757969 1.93275445496665 0.23993355556135
H 2.69277498983382 0.88269812259497 -6.15821460453484
C -1.98049392194559 1.76283699920343 1.59379070893026
C -1.30472453017788 1.78467452155695 3.90965149804448
P 0.93471257604503 -0.55848018011056 -0.75773617079726
C 4.91127599012154 0.65231131889254 0.84231861871923
C 3.71387495380704 -1.61137932979211 0.69306053559437
C 6.06367293475774 2.84183211008097 0.91391815183696
O -1.79926147528593 2.25435076776187 0.31321258042968
C -3.02489897911231 0.86205080734831 1.84489004376141
H -0.66973650063930 2.17390669341752 4.72001905758223
C -2.27331231886241 0.78650733312617 4.21306504735714

| | | | |
|---|---|---|---|
| O | 1.34901515567612 | -1.12858988129324 | 0.71675201143808 |
| N | 0.27094795722203 | 0.84337436276750 | -0.55840858803627 |
| O | 0.15469839476342 | -1.69827034433958 | -1.57020478212181 |
| C | 5.48269208789414 | 0.36323795270209 | 2.11670113691074 |
| C | 4.77014632222149 | -2.56401790717216 | 0.93406647236151 |
| C | 2.39888814153281 | -2.01058821293862 | 0.95156683140962 |
| H | 6.27750387019307 | 3.81157928609188 | 0.43805028969996 |
| C | 6.61157487338737 | 2.52461494600883 | 2.14709234489388 |
| P | -1.24923780305815 | 1.29981445507293 | -0.92317041783288 |
| C | -3.95466276816932 | 0.45617378406801 | 0.75481599908470 |
| C | -3.15227390871326 | 0.31258599124769 | 3.17210458263468 |
| C | -2.38523264449010 | 0.23714527833756 | 5.52483792704918 |
| H | -0.82695246565809 | -1.55255684748040 | -1.55696831308146 |
| C | 6.30799255451009 | 1.27814422253967 | 2.75500362317833 |
| H | 5.24519949861191 | -0.59175244897511 | 2.60405145669675 |
| C | 6.12266788275325 | -2.32731515386555 | 0.54745786351785 |
| C | 4.45054947761706 | -3.81738568699796 | 1.57188754536526 |
| C | 2.05391295812977 | -3.26044038486420 | 1.55566526743176 |
| H | 7.27102881520098 | 3.24001967718580 | 2.66091330627251 |
| O | -2.06864166199640 | -0.14468681928213 | -0.59325249123159 |
| O | -1.58179653009180 | 1.94565094796222 | -2.22643943895202 |
| C | -3.44344705417957 | -0.08840418367931 | -0.42432027607391 |
| C | -5.38590279954021 | 0.59088199628382 | 0.86707257669888 |
| C | -4.08825104056844 | -0.71502635211198 | 3.49730664153068 |
| H | -1.70455861745211 | 0.60737759279611 | 6.30703802170832 |
| C | -3.31682513870638 | -0.74918755531533 | 5.81050537501145 |
| H | 6.72379315656944 | 1.03694511084758 | 3.74477616712817 |
| H | 6.37250276579508 | -1.39562144857428 | 0.02197071495510 |
| C | 7.11587054499195 | -3.25851520566190 | 0.81491319341189 |
| C | 5.49843121543228 | -4.74464737312644 | 1.84922872786634 |
| C | 3.09641074097661 | -4.11570327355822 | 1.89123896844002 |
| C | 0.62442351032620 | -3.61307417028902 | 1.80315409882507 |
| C | -4.25243148458989 | -0.63781774505662 | -1.46364908524140 |
| C | -6.22539321940969 | 0.04053701264938 | -0.17093064980655 |
| C | -6.01107757206278 | 1.27680924494891 | 1.95028687349490 |
| C | -4.17107876898820 | -1.23047255617865 | 4.78326075656066 |
| H | -4.73992034925954 | -1.11188628163921 | 2.70765311843920 |
| H | -3.38905954361963 | -1.16624580264879 | 6.82615279784926 |
| H | 8.15056769715914 | -3.05592507181910 | 0.49984809330308 |
| C | 6.80741118290141 | -4.47171918487971 | 1.48462798515314 |
| H | 5.24175377493606 | -5.69217158436508 | 2.34805473742268 |
| H | 2.86755760591333 | -5.06341903445479 | 2.40200735622698 |
| C | 0.02884573538312 | -4.70027527136275 | 1.10361899150895 |
| C | -0.14459406939717 | -2.85910754088376 | 2.72926520329795 |
| C | -5.62964037166061 | -0.59007229631551 | -1.30223487006184 |
| C | -3.55048403283579 | -1.23142642710548 | -2.63989670575972 |
| C | -7.64188034013876 | 0.15468680827394 | -0.05149432625933 |
| C | -7.39226311644950 | 1.38318540735097 | 2.02798610030234 |
| H | -5.38065896637911 | 1.73394136593215 | 2.72548204240764 |
| H | -4.89609195058214 | -2.02863116289428 | 5.00283166082214 |

| | | | |
|---|---|---|---|
| H | 7.60515422554253 | -5.19873000704450 | 1.69871714780471 |
| C | -1.32762272776462 | -4.99286418532426 | 1.34000022211842 |
| C | 0.78883038231247 | -5.53376900157861 | 0.08620383073844 |
| C | -1.49982951388901 | -3.18062861260315 | 2.91377716081865 |
| C | 0.44896095132926 | -1.75585899224762 | 3.57997995046177 |
| H | -6.28119550073228 | -1.02601445811944 | -2.07477010123549 |
| C | -2.92422280935201 | -2.50115453157414 | -2.50430459588053 |
| C | -3.39378228477386 | -0.47939196084033 | -3.82978624630241 |
| H | -8.27069955020119 | -0.27703343476165 | -0.84571968856719 |
| C | -8.21817733524763 | 0.80718858989788 | 1.02726476019398 |
| H | -7.85026592314045 | 1.92433302007848 | 2.86967339578777 |
| C | -2.11455996660405 | -4.24157765930842 | 2.23002178863653 |
| H | -1.78743143587456 | -5.83556403544551 | 0.79727172939715 |
| H | -2.09292656311807 | -2.57775728065668 | 3.61814622634583 |
| C | -2.14069671062336 | -2.98849003514652 | -3.56477914407936 |
| C | -3.11555223667451 | -3.35341443439754 | -1.26635793445457 |
| C | -2.60862920377094 | -1.01705174864960 | -4.86781862865720 |
| C | -4.04194517876549 | 0.87551791261491 | -4.01238947362476 |
| H | -9.31267613889426 | 0.89247800963346 | 1.10249621771701 |
| C | -1.95496812022442 | -2.25328431956239 | -4.75004496593292 |
| H | -1.64281382579262 | -3.96596345224936 | -3.45120798329876 |
| H | -2.47498077219397 | -0.42624910807128 | -5.78759119366541 |
| C | -0.98822886586534 | -2.73234305814171 | -5.80802673814546 |
| C | 0.46541181535800 | -2.39292148522325 | -5.44153147658386 |
| C | -5.41471493633029 | 0.80055022778178 | -4.69490526376457 |
| C | -4.44247900936976 | -4.12479243869901 | -1.27409427181509 |
| C | 2.86664656205654 | 6.46968588773221 | 1.77927350116430 |
| C | 3.52093565006770 | 6.44636043316270 | 0.39033220845496 |
| C | -2.54503371088361 | 5.44428546047207 | 0.82905916921334 |
| C | 2.42834812575617 | 1.31930179948821 | 3.80177381572143 |
| C | 0.58213322132667 | 2.62931705279380 | -6.42191855560518 |
| C | -0.66346200555164 | 1.73114760175972 | -6.37154930010582 |
| C | 3.79756164715683 | -1.35101599152098 | -4.41850425351122 |
| C | 0.53791714604561 | 4.66101157701837 | -1.64423672769678 |
| C | -3.56998534852402 | -4.57111795313675 | 2.48097095128535 |
| C | -3.84108870672273 | -5.08312014797514 | 3.90231775683725 |
| C | 1.08978524843183 | -4.81610920665565 | -1.23666145176342 |
| C | 0.51013011381382 | -2.11307962858364 | 5.07122991030906 |
| H | 2.61548196317905 | 4.18004018378672 | -1.28698277471452 |
| H | 1.49761903012741 | 2.93669890810607 | -0.75965409217907 |
| H | 2.49756485191089 | 1.39458207151148 | 1.64312126998121 |
| H | 0.93600457287151 | 0.88268857667448 | 2.28138016290341 |
| H | -2.09118593052226 | 5.98746495102523 | 2.88291123214940 |
| H | -2.59749029192889 | 4.29958313422133 | 2.67783573505617 |
| H | 4.91117006484270 | 0.07802192062653 | -3.21829695415498 |
| H | 5.00972292650961 | 0.36339714282327 | -4.96154557175189 |
| H | 0.19201443670273 | -6.44622318260317 | -0.12187679760463 |
| H | 1.74406153857040 | -5.89277699726530 | 0.52094446128795 |
| H | -0.16123100144958 | -0.83764411697062 | 3.45061125119783 |
| H | 1.46576124115766 | -1.49963125884314 | 3.23081315523630 |

| | | | |
|---|---|---|---|
| H | -3.05389293598674 | -2.73298901005072 | -0.34789108434024 |
| H | -2.27415550004998 | -4.07194291721035 | -1.19324107252791 |
| H | -3.36341139424830 | 1.50919987400398 | -4.61855146392064 |
| H | -4.12333343011770 | 1.38506613476764 | -3.03280226199192 |
| H | -1.24659105599290 | -2.27055139092017 | -6.78417643575834 |
| H | -1.09074769153665 | -3.83082179355632 | -5.94027286185261 |
| H | 1.17283288503335 | -2.74503798205530 | -6.21976999349847 |
| H | 0.75466807931424 | -2.86048001777267 | -4.47856682153739 |
| H | 0.60364129140768 | -1.29932771211345 | -5.31814425499373 |
| H | -5.34398230776354 | 0.31136191736172 | -5.68828616379675 |
| H | -5.84098310930426 | 1.81352337163364 | -4.84315814860118 |
| H | -6.13821043459673 | 0.21587609646009 | -4.09148539231932 |
| H | -4.52002512943506 | -4.77053244036247 | -2.17212493271580 |
| H | -5.30762555393762 | -3.43180903238352 | -1.28211214501749 |
| H | -4.53267746978735 | -4.76869799312925 | -0.37644312770902 |
| H | 3.65675619437777 | 6.36037226043241 | 2.55397988400409 |
| H | 2.39781792387452 | 7.46096504023899 | 1.95226299897435 |
| H | 4.03012096467142 | 5.47801181950273 | 0.20681526454930 |
| H | 4.27718962240430 | 7.25123126183186 | 0.28831391008508 |
| H | 2.76630300234028 | 6.57972300400501 | -0.41074793410520 |
| H | -1.97345200514587 | 6.27144180329083 | 0.36041823068625 |
| H | -3.60522104567902 | 5.76275265043454 | 0.89817224590080 |
| H | -2.48863653450706 | 4.57115840962811 | 0.15140175645286 |
| H | 1.70802257587882 | 1.42822403931592 | 4.63857388077658 |
| H | 3.27058922517199 | 2.01600285558951 | 3.98727288327426 |
| H | 2.83564323834327 | 0.28849472950061 | 3.83691286009520 |
| H | 1.15100119773169 | 2.43479320913902 | -7.35597554172223 |
| H | 0.26941080940871 | 3.69429784318749 | -6.45541435809518 |
| H | -0.37571993934543 | 0.66185725297939 | -6.42875874979469 |
| H | -1.36114365315224 | 1.94527425688036 | -7.20723936149076 |
| H | -1.20429412398902 | 1.86813147897464 | -5.41277256591239 |
| H | 4.63890801565198 | -2.07239459707434 | -4.46105018687929 |
| H | 3.25706357796561 | -1.40921461561572 | -5.38427930542358 |
| H | 3.09815870793536 | -1.67900919778885 | -3.62553391922099 |
| H | -0.41651385021232 | 4.16782930085026 | -1.91608207710183 |
| H | 0.72943856655216 | 5.46829004920457 | -2.38190140441533 |
| H | 0.40228380550561 | 5.13429319571821 | -0.65295717209937 |
| H | -3.90947889972843 | -5.32587631322620 | 1.74141134810081 |
| H | -4.18258269065057 | -3.66072709265397 | 2.29880837438691 |
| H | -3.27510133965954 | -6.01471029109229 | 4.10639809594021 |
| H | -4.91890501808977 | -5.29612096371162 | 4.05477155048974 |
| H | -3.53250554326093 | -4.33568306997832 | 4.66113196610959 |
| H | 1.74455862915374 | -3.93561098084325 | -1.08723963110015 |
| H | 0.16368804413946 | -4.44801187934642 | -1.72027890885042 |
| H | 1.60204155199124 | -5.49856370614083 | -1.94477519996762 |
| H | 1.13306043326437 | -3.01504624490671 | 5.24147748945636 |
| H | -0.49968122797637 | -2.31638021840787 | 5.48081354215144 |
| H | 0.94787418442814 | -1.27846724091431 | 5.65631311742015 |

372

IDP Dimer

| | | | |
|---|---|---|---|
| C | 4.54378629646217 | 1.83638618186803 | 6.09422688584566 |
| C | 3.46668441398304 | 6.06321031507097 | -1.54579823988320 |
| C | 5.64395453537930 | 1.69592350089287 | 5.23023539482701 |
| C | 3.55626481554445 | 0.83833473803383 | 6.06881893515172 |
| C | 2.49622775592304 | 5.19953127299301 | -2.11894136530945 |
| C | 3.03159536179434 | 7.14998595196342 | -0.75730186274031 |
| C | 4.96044855993932 | 5.88762299959182 | -1.74074490367400 |
| C | 5.75755692785247 | 0.61932473197746 | 4.33077008648568 |
| H | 6.43103247484827 | 2.46839424628263 | 5.22842213351495 |
| C | 3.63784933739111 | -0.27011956555057 | 5.20721664602459 |
| H | 2.68286368267932 | 0.92321204291035 | 6.73606476260575 |
| C | 2.88498654080548 | 4.18681456762477 | -3.14821215497654 |
| C | 1.11417403204068 | 5.41425808587333 | -1.84414562788097 |
| C | 1.67372248691153 | 7.42530317903930 | -0.53698075146278 |
| H | 3.79059217813874 | 7.82482318039784 | -0.32733854422023 |
| C | 4.73425922426518 | -0.36982302379655 | 4.31137184769548 |
| C | 6.89231482536237 | 0.61940381352542 | 3.32837293645695 |
| C | 2.60530321461853 | -1.36723286521176 | 5.33902217447004 |
| C | 3.80072872979540 | 3.11997161872607 | -2.91569053959319 |
| C | 2.38683127124450 | 4.31315266889373 | -4.44423102686377 |
| C | 0.73366825863377 | 6.53196006557255 | -1.08517477496880 |
| C | 0.04970056725129 | 4.42314004872604 | -2.26460200104049 |
| C | 4.88572073807677 | -1.58000464152888 | 3.44560763823860 |
| O | 4.05722490452560 | 2.76216190433112 | -1.57795236353813 |
| C | 4.37880012570879 | 2.36265136421459 | -3.94521898167419 |
| H | 1.70814155206246 | 5.14856802009568 | -4.67150874521660 |
| C | 2.67312854729562 | 3.35706357277316 | -5.45590333431433 |
| H | -0.34219726617483 | 6.70229003204379 | -0.91478248660358 |
| C | 4.02049363650998 | -1.89661911686065 | 2.34493760176284 |
| C | 5.88867772744971 | -2.48726252435696 | 3.77088393309736 |
| P | 3.81493705533926 | 1.17172448174619 | -1.24878746180864 |
| C | 3.68781563217043 | 2.36463999028251 | -5.21148522640571 |
| C | 5.68257530528332 | 1.65159680916718 | -3.79893831539726 |
| C | 1.97554830412610 | 3.34921496152196 | -6.70003203973171 |
| O | 2.96928717622618 | -1.01398295436792 | 2.11488575226491 |
| C | 4.12528289051104 | -3.11060010852537 | 1.64151750089175 |
| H | 6.51989232815647 | -2.27799410519699 | 4.64803439638934 |
| C | 6.14970500859509 | -3.64442265957984 | 2.99308298660912 |
| O | 5.24476536134908 | 0.50338830705003 | -1.65159077824750 |
| N | 3.50296512484842 | 0.88933635577870 | 0.26679098336974 |
| O | 2.72070579146203 | 0.74119716586729 | -2.33840438256339 |
| C | 3.92537241911625 | 1.37484813381323 | -6.21074354606075 |
| C | 6.68802455949296 | 1.92778302357073 | -4.81852587028791 |
| C | 6.06492488921976 | 0.83006785546512 | -2.72735853955381 |
| H | 1.20854833231216 | 4.11877479283154 | -6.87690010208348 |
| C | 2.22652311233231 | 2.37150655400218 | -7.64980465325107 |
| P | 2.51776661782613 | -0.34255674781465 | 0.68099383721462 |
| C | 3.08556910664204 | -3.61240067778863 | 0.70420661198389 |
| C | 5.28901380563428 | -3.93792697095966 | 1.87941144558050 |
| C | 7.25462111572960 | -4.50176184844897 | 3.27561333371641 |

| | | | |
|---|---|---|---|
| H | 2.43372505626878 | -0.21359503679111 | -2.38271847335512 |
| C | 3.19998195279071 | 1.36889200047555 | -7.39358000189617 |
| H | 4.67581930726923 | 0.59464987535117 | -6.02006287119626 |
| C | 6.53859794566971 | 2.94103417739365 | -5.81372480342784 |
| C | 7.91626676770157 | 1.17684022472953 | -4.82617463902425 |
| C | 7.36362035123653 | 0.22551040494443 | -2.63258654680624 |
| H | 1.66542783490613 | 2.36192652461274 | -8.59616860644902 |
| O | 3.00015209140031 | -1.49912316859360 | -0.42429439461674 |
| O | 1.02714354757099 | -0.12908857511308 | 0.73532561521297 |
| C | 2.58039671285625 | -2.81618743455178 | -0.32398866679169 |
| C | 2.57611844516568 | -4.96378824185908 | 0.81362285345286 |
| C | 5.64089013695752 | -5.03356065989634 | 1.03409913270268 |
| H | 7.88215832517398 | -4.27844543529609 | 4.15208021027078 |
| C | 7.55077944546533 | -5.57577873215169 | 2.45135781771252 |
| H | 3.37861371855393 | 0.57802582191131 | -8.13757367235327 |
| H | 5.65957983958948 | 3.59620394835046 | -5.79393213929170 |
| C | 7.49402429889239 | 3.13168603705686 | -6.80282567988035 |
| C | 8.86477104015127 | 1.36840136312959 | -5.87361949885923 |
| C | 8.21157024192144 | 0.33235977687935 | -3.72440519317059 |
| C | 7.89236435081441 | -0.36324594424153 | -1.36426709600809 |
| C | 1.71385618160664 | -3.29531055816214 | -1.34853574698034 |
| C | 1.74439252132945 | -5.48491740832308 | -0.24322262175728 |
| C | 2.82829402223022 | -5.80302051695380 | 1.93917965541064 |
| C | 6.74845579659525 | -5.82418615311238 | 1.30637860057534 |
| H | 5.03347481630976 | -5.23938016232629 | 0.14306910460909 |
| H | 8.41323995299118 | -6.22266133773936 | 2.67043582854098 |
| H | 7.34723602851080 | 3.92898032065427 | -7.54686872448252 |
| C | 8.65521021049938 | 2.32004334740044 | -6.85812935721610 |
| H | 9.78358034058444 | 0.76167040285093 | -5.86335230739674 |
| H | 9.18452892354622 | -0.18147661924691 | -3.69073246327256 |
| C | 7.42549548235669 | -1.59490672220856 | -0.84365537051039 |
| C | 8.93455560994327 | 0.34456561599284 | -0.69551277010150 |
| C | 1.37390547076696 | -4.64032776597864 | -1.32727834554381 |
| C | 1.12915182881444 | -2.35804669878864 | -2.35111021671368 |
| C | 1.24316253565462 | -6.81699951949165 | -0.15208231480338 |
| C | 2.30562388420429 | -7.08624741682335 | 2.01134235961286 |
| H | 3.42847050337331 | -5.41629453375675 | 2.77263226455584 |
| H | 7.00549728267451 | -6.64968389177429 | 0.62589578013056 |
| H | 9.39622515473746 | 2.46831948466237 | -7.65780958373265 |
| C | 8.00150046383504 | -2.10256114317346 | 0.33550680734462 |
| C | 6.36533816041317 | -2.41060943120089 | -1.55134777293576 |
| C | 9.48063801241836 | -0.20826039528573 | 0.47608988398547 |
| C | 9.44122426991158 | 1.69962210724195 | -1.16024215183658 |
| H | 0.74208840150941 | -5.04330245425067 | -2.13274857197304 |
| C | 1.87840563223338 | -1.90798539588544 | -3.47005330366025 |
| C | -0.20662270311318 | -1.91639409923323 | -2.14767322023816 |
| H | 0.61016137971772 | -7.19449019608280 | -0.97024806718527 |
| C | 1.51503224846270 | -7.60670392958003 | 0.95396171743532 |
| H | 2.49943732134912 | -7.70107340925808 | 2.90304116008007 |
| C | 9.03222754243144 | -1.42768287366192 | 1.01069689646340 |

| | | | |
|---|---|---|---|
| H | 7.63706323945937 | -3.06271759171658 | 0.73034438932770 |
| H | 10.28266607621086 | 0.34200016260624 | 0.99564888054512 |
| C | 1.25948443652311 | -1.03910752003963 | -4.39419932136679 |
| C | 3.28558052103003 | -2.40702484258002 | -3.73035143818136 |
| C | -0.78126132115088 | -1.05217069744828 | -3.09911253206071 |
| C | -0.98641980476965 | -2.29206241746170 | -0.90212099352190 |
| H | 1.10912880714099 | -8.62717039420279 | 1.02085983468900 |
| C | -0.06827369968063 | -0.60937609137899 | -4.23066890878414 |
| H | 1.83982727722847 | -0.68181898304871 | -5.26127457329220 |
| H | -1.81561850054745 | -0.70288878736119 | -2.94430618752231 |
| C | -0.70869322727992 | 0.32616103838141 | -5.23283433921803 |
| C | -0.80233063046778 | 1.78002090949912 | -4.75559792750385 |
| C | -1.67117583145363 | -3.65854688409909 | -0.84471139692553 |
| C | 3.31848520257080 | -3.71306748162709 | -4.53611937683270 |
| C | 4.37196249294969 | 3.06995609113819 | 6.95060237696516 |
| C | 3.59218128688797 | 4.17413133136560 | 6.21806803056091 |
| C | 3.01896635392447 | -2.41582793026114 | 6.38193646375835 |
| C | 6.48176421931943 | 1.25752844313071 | 1.99565261142050 |
| C | 1.22609333716736 | 8.62255135201937 | 0.27589151513932 |
| C | 0.43597813806500 | 8.26656759632124 | 1.54423570735273 |
| C | 0.29371194882680 | 3.01676058841246 | -1.70881925554355 |
| C | 5.67516705530556 | 5.37645553427816 | -0.47946708230954 |
| C | 9.66749551530754 | -2.01635509454372 | 2.25040438746884 |
| C | 10.55010420885069 | -3.23624822829329 | 1.95242716372461 |
| C | 6.90759566985983 | -3.69992581923797 | -2.18162717974834 |
| C | 8.46274025083933 | 2.85789239747141 | -0.91546830501102 |
| H | 5.39493736026841 | 6.86860224907832 | -2.03092348845328 |
| H | 5.16461452886302 | 5.20326919736601 | -2.58818270363542 |
| H | 7.24515418650418 | -0.41083460300587 | 3.12936088057313 |
| H | 7.75555397301113 | 1.16875582827004 | 3.75927844216500 |
| H | 2.42710944652707 | -1.85806637575773 | 4.36442194628025 |
| H | 1.63730319464884 | -0.91516696077658 | 5.63440729562446 |
| H | -0.04053409936786 | 4.36795663955803 | -3.37009499190874 |
| H | -0.92514611399262 | 4.80420229580838 | -1.90407253497269 |
| H | 5.88200815741831 | -1.79871051369118 | -2.33424453688942 |
| H | 5.56914406589977 | -2.66045081911296 | -0.81935084177841 |
| H | 9.69421819262551 | 1.67024358357017 | -2.23897231529879 |
| H | 10.39359828212798 | 1.91027110908504 | -0.63097147431947 |
| H | 3.81388270732971 | -2.56113610140927 | -2.76886855256703 |
| H | 3.85286909876790 | -1.62132137879393 | -4.27478686375816 |
| H | -1.79260891629488 | -1.54797593499350 | -0.80718536552568 |
| H | -0.33630226923429 | -2.15743275329831 | -0.01386960779035 |
| H | -1.73169903656848 | -0.04111696609084 | -5.45363318158763 |
| H | -0.13777198799725 | 0.28358846851418 | -6.18484220079600 |
| H | -1.42997861012056 | 1.85730536963402 | -3.84606154618306 |
| H | -1.25574097026086 | 2.42274853092409 | -5.53772239519990 |
| H | 0.19988827599673 | 2.18640895156660 | -4.51802118562139 |
| H | -2.32605298146955 | -3.79144194333528 | -1.72050687696308 |
| H | -2.30231491815694 | -3.71063712517574 | 0.06280376256675 |
| H | -0.95729502668631 | -4.50219651751168 | -0.80613294071054 |

| | | | |
|---|---|---|---|
| H | 4.36109093569310 | -4.04676252755435 | -4.71558077753911 |
| H | 2.82190665844785 | -3.58796935787334 | -5.51965429623590 |
| H | 2.78974238317568 | -4.52329000755742 | -3.99421527475637 |
| H | 5.36801740734699 | 3.45559431017117 | 7.25551404643575 |
| H | 3.83697939699897 | 2.79909692174825 | 7.88558902986463 |
| H | 4.11598559657189 | 4.48543786427905 | 5.29186828643940 |
| H | 3.46094404913025 | 5.06936975312242 | 6.85988515970528 |
| H | 2.59021274182089 | 3.81216334814033 | 5.91153668157151 |
| H | 3.96667850641435 | -2.91461400946892 | 6.09236531595716 |
| H | 2.24323075995699 | -3.19956673751908 | 6.49304454318137 |
| H | 3.17812105476753 | -1.94549766912224 | 7.37348740207442 |
| H | 5.60502374629467 | 0.74769911970648 | 1.55342619643191 |
| H | 7.30701847344316 | 1.19814626806776 | 1.26205205158517 |
| H | 6.20272415111515 | 2.32368216771840 | 2.12502028795665 |
| H | 0.59876838780139 | 9.27899519372655 | -0.36726979830627 |
| H | 2.11798320437674 | 9.22410511878138 | 0.54928794425667 |
| H | 0.18483941109082 | 9.17887035617240 | 2.12213509897835 |
| H | 1.01888708547734 | 7.59364594793776 | 2.20552631696359 |
| H | -0.52116606602681 | 7.75681884829247 | 1.30901410973700 |
| H | 0.52031365007682 | 3.04902996859143 | -0.62709507818936 |
| H | 1.13584981367116 | 2.51480993420663 | -2.21729842838525 |
| H | -0.59301440537250 | 2.37283265435558 | -1.84343510798814 |
| H | 5.51671359379243 | 6.06457612192592 | 0.37621982457762 |
| H | 6.76741793013435 | 5.29277070120740 | -0.64816334507574 |
| H | 5.29414894988762 | 4.37925336632345 | -0.18768008024051 |
| H | 8.86856850088347 | -2.31331253545739 | 2.96193203498712 |
| H | 10.26622277411998 | -1.23385668068423 | 2.76188979968994 |
| H | 10.99447976902298 | -3.64813848368211 | 2.88174300972313 |
| H | 11.37822831611708 | -2.97196081842114 | 1.26387960053042 |
| H | 9.95958306721552 | -4.04337022853726 | 1.47379100960686 |
| H | 7.37765726317315 | -4.35496070178979 | -1.42070704642731 |
| H | 7.67200688263581 | -3.47565404236874 | -2.95341161269933 |
| H | 6.09107419694431 | -4.27547076691543 | -2.66495264900060 |
| H | 7.49980943338733 | 2.69930195885225 | -1.43981587887414 |
| H | 8.88796814291857 | 3.81476728894293 | -1.28062700026509 |
| H | 8.23435923357329 | 2.97468894315243 | 0.16231615850443 |
| C | -7.39293065794424 | 4.31573186961003 | 1.13624141410159 |
| C | -3.00136612500421 | 5.81931954875632 | 2.11072548215403 |
| C | -6.59075311275296 | 3.64713575505018 | 2.07332283744809 |
| C | -8.06643698438693 | 3.53886089258931 | 0.17949748923020 |
| C | -2.73940102325672 | 4.80588868927974 | 1.13683202118064 |
| C | -3.83174265926384 | 6.89840618901308 | 1.76663375767239 |
| C | -2.44875219627136 | 5.74062583698965 | 3.52257885855130 |
| C | -6.47941635950895 | 2.24494324111040 | 2.09791474236033 |
| H | -6.02138582440401 | 4.23695867902189 | 2.81019609011100 |
| C | -7.97549116803022 | 2.13642373517546 | 0.14687542989451 |
| H | -8.70026434887157 | 4.04431874845298 | -0.56877050104876 |
| C | -1.59269313581019 | 3.88733020228191 | 1.45029739578854 |
| C | -3.39695586566832 | 4.85217046170781 | -0.11451287208643 |
| C | -4.41108072646461 | 7.01504568630425 | 0.48908373912086 |

| | | | |
|---|---|---|---|
| H | -4.01621506730987 | 7.68551763178258 | 2.51701975698092 |
| C | -7.18630719602530 | 1.47716956217736 | 1.13247573295491 |
| C | -5.59107724747323 | 1.62116588236031 | 3.15340357877822 |
| C | -8.75653661612291 | 1.40196330542565 | -0.92789010185660 |
| C | -1.67024607376570 | 2.55640374203432 | 1.96082102556133 |
| C | -0.33268214375386 | 4.47868056936122 | 1.44822495517950 |
| C | -4.20843603633392 | 5.96551856979218 | -0.41661767805707 |
| C | -3.31009616584138 | 3.73731672487926 | -1.13077765339660 |
| C | -7.19180831623695 | -0.01456195401559 | 1.21205402551167 |
| O | -2.80723627052785 | 1.78144864925854 | 1.68577668858625 |
| C | -0.63628299202308 | 1.98359673229158 | 2.71156041638439 |
| H | -0.23352864024958 | 5.49711084042673 | 1.05343425708439 |
| C | 0.82878516007790 | 3.82385824544549 | 1.93136469247009 |
| H | -4.69032681669899 | 6.01088743482605 | -1.40737325713204 |
| C | -6.72383703626810 | -0.84122303475157 | 0.14187832725147 |
| C | -7.75635374994076 | -0.65536593049235 | 2.31117751475772 |
| P | -2.46809056584276 | 0.39617287495179 | 0.83473238793986 |
| C | 0.67037883432926 | 2.58234961481073 | 2.63832290265815 |
| C | -0.90699833377717 | 0.85255228292184 | 3.64287264117231 |
| C | 2.12882033500828 | 4.38425101845939 | 1.76084209792918 |
| O | -6.09087760203563 | -0.23908469581862 | -0.93264115905685 |
| C | -6.95131388187817 | -2.22120936136212 | 0.09976333828604 |
| H | -8.14838801717493 | -0.04459818378659 | 3.13769130564074 |
| C | -7.84906832977019 | -2.07200400991072 | 2.40486802522789 |
| O | -1.81438944150474 | -0.62040244114589 | 1.92913601366729 |
| N | -3.77300068963318 | -0.22788275668633 | 0.25986104186315 |
| O | -1.27715094228452 | 0.73780702516657 | -0.16334676829608 |
| C | 1.82997013620718 | 1.95451859512079 | 3.16919567081005 |
| C | -0.64070704990304 | 1.03377180800801 | 5.05490294879721 |
| C | -1.47028272014479 | -0.35874855186623 | 3.24052458819771 |
| H | 2.23006545340746 | 5.31711513858458 | 1.18623123309784 |
| C | 3.23830021077085 | 3.73799095538914 | 2.27990775362591 |
| P | -4.47127098975668 | -0.55187548319134 | -1.17039405333275 |
| C | -6.76016830527350 | -2.94663995446214 | -1.18253912034864 |
| C | -7.46048542185559 | -2.87639179935354 | 1.27455737583844 |
| C | -8.33551519030286 | -2.71494458237141 | 3.58199057116967 |
| H | -0.34932154446272 | 0.45203598570226 | 0.16524356537835 |
| C | 3.08240945152698 | 2.51813900361347 | 2.98595172050730 |
| H | 1.73550845238899 | 0.99442641321527 | 3.69094544939381 |
| C | -0.21280662510708 | 2.27880383934850 | 5.60774169497697 |
| C | -0.83992987481274 | -0.07112303653134 | 5.96059915726094 |
| C | -1.70670414082467 | -1.45408372218456 | 4.12847108649307 |
| H | 4.24457991715330 | 4.15629713550192 | 2.12685670466369 |
| O | -4.47628707314453 | -2.22480115876558 | -1.26473619137106 |
| O | -3.99283249976059 | 0.08916033946161 | -2.43814018382377 |
| C | -5.53014494700357 | -2.90799080176768 | -1.85381456791540 |
| C | -7.88401301066695 | -3.61616712702807 | -1.79066017403333 |
| C | -7.57932670629103 | -4.29429835850791 | 1.37594822580603 |
| H | -8.62323933952445 | -2.08896554828897 | 4.44104645474401 |
| C | -8.43348713764335 | -4.09648329983868 | 3.65174040680835 |

| | | | |
|---|---|---|---|
| H | 3.96358899840461 | 1.99484841256326 | 3.37067398541652 |
| H | -0.10170178592289 | 3.14818354036601 | 4.94674816866346 |
| C | 0.05636430457114 | 2.41125622231671 | 6.96310673732621 |
| C | -0.53729927639827 | 0.09166099128428 | 7.34480115140420 |
| C | -1.35876655075861 | -1.29923210235340 | 5.46124726567170 |
| C | -2.35640938721978 | -2.69854954418434 | 3.61626219853017 |
| C | -5.32470111528686 | -3.60465717190887 | -3.09306858828083 |
| C | -7.68135841892728 | -4.33743713155916 | -3.01959914105345 |
| C | -9.20814862395955 | -3.56175763880550 | -1.25770921947411 |
| C | -8.05147755677771 | -4.89020865323090 | 2.53698407814851 |
| H | -7.27364344066495 | -4.91491831304524 | 0.52264979684176 |
| H | -8.80307865016238 | -4.57948582154084 | 4.56875139772872 |
| H | 0.38051829379102 | 3.38576692462459 | 7.35846796557099 |
| C | -0.08638987458373 | 1.30515219082296 | 7.84095363663391 |
| H | -0.68173192592167 | -0.76951616536625 | 8.01571359394094 |
| H | -1.52331975781793 | -2.13579006110983 | 6.15738819668729 |
| C | -1.58182285579774 | -3.72080073408274 | 3.01341561984410 |
| C | -3.76285204180553 | -2.82893551964494 | 3.71207125558210 |
| C | -6.39609447772830 | -4.31706631589965 | -3.62250107629063 |
| C | -4.04378014747765 | -3.60309198098347 | -3.87084137007929 |
| C | -8.77638986440727 | -5.02136771635404 | -3.62543204348684 |
| C | -10.25761448161655 | -4.22417650307920 | -1.87785282871645 |
| H | -9.39738562663692 | -2.97489278400926 | -0.34949135491091 |
| H | -8.12258470635128 | -5.98681260810639 | 2.59661011863991 |
| H | 0.13993635010823 | 1.41799961916935 | 8.91187960269359 |
| C | -2.22809138231106 | -4.88430630105635 | 2.56225109629254 |
| C | -0.07865882086505 | -3.60479559470815 | 2.89330350935992 |
| C | -4.37089680419163 | -4.00471020308845 | 3.23568347483366 |
| C | -4.62678475003686 | -1.74827939311464 | 4.32389235477085 |
| H | -6.24921825353587 | -4.85806371316975 | -4.56988034536986 |
| C | -3.14302054159221 | -4.69423618492146 | -3.76060449350393 |
| C | -3.79818875860960 | -2.57347329162467 | -4.81577267175735 |
| H | -8.59603724945816 | -5.57583003616780 | -4.55973247114786 |
| C | -10.04203165806820 | -4.97525772175910 | -3.06347692640091 |
| H | -11.26903201381805 | -4.15925746077922 | -1.44882020600858 |
| C | -3.62196782477474 | -5.04728735414337 | 2.66723750723005 |
| H | -1.61961516565449 | -5.67827123652472 | 2.09620030898114 |
| H | -5.46637827475256 | -4.10087093639266 | 3.29763099059919 |
| C | -1.97773182114352 | -4.70182525713115 | -4.54934809297901 |
| C | -3.42391919887351 | -5.87135206027776 | -2.85050530399452 |
| C | -2.63819524718132 | -2.64569801084529 | -5.60704961110765 |
| C | -4.79356119990953 | -1.45891608812323 | -5.05734944078819 |
| H | -10.88176661429304 | -5.50208249069778 | -3.54131058829633 |
| C | -1.69907301213661 | -3.68247930541386 | -5.47321180511398 |
| H | -1.26359882158969 | -5.53574862866181 | -4.43532779223875 |
| H | -2.45630101695529 | -1.85279179926646 | -6.35052746066376 |
| C | -0.45926099322565 | -3.72776417829566 | -6.33754562479587 |
| C | -0.70050844099411 | -4.41513166399366 | -7.68918146598590 |
| C | -5.76712365743411 | -1.77876954245826 | -6.20050758290071 |
| C | -4.03337772131505 | -7.07489793990272 | -3.58262449397732 |

| | | | |
|---|---|---|---|
| C | -7.58546935340627 | 5.81289423101916 | 1.19837313995110 |
| C | -8.86830988865524 | 6.21226199006683 | 1.94251146308255 |
| C | -8.23031990408925 | 1.65026925343101 | -2.34971952635637 |
| C | -6.16452526539074 | 1.66122397614272 | 4.57567075670843 |
| C | -5.17288727250239 | 8.25910052883512 | 0.09229063791829 |
| C | -4.24641794243533 | 9.35500404367202 | -0.45735140289914 |
| C | -4.65781751215556 | 3.02356709545782 | -1.29984569287365 |
| C | -3.15903464759261 | 4.69267381746660 | 4.39319284057646 |
| C | -4.30548594255152 | -6.27454654944625 | 2.10631251541823 |
| C | -4.38197284227403 | -6.24602636861668 | 0.57424177481841 |
| C | 0.64565928623524 | -4.19975281579229 | 4.10775216530427 |
| C | -5.05733726429706 | -2.05630275727213 | 5.76388043074020 |
| H | -2.54636715186616 | 6.73936895108069 | 3.99674051256250 |
| H | -1.36324004534771 | 5.51400770082442 | 3.49948957693117 |
| H | -4.62966514403381 | 2.17014042557115 | 3.14555983853348 |
| H | -5.34361485288922 | 0.58116756453924 | 2.87037115735739 |
| H | -9.81702033299083 | 1.73285251576391 | -0.87057706522591 |
| H | -8.77106266157680 | 0.31389576617791 | -0.72293232600559 |
| H | -2.52243913626112 | 3.01505997660479 | -0.84184303636369 |
| H | -2.99789061852652 | 4.15573715819949 | -2.11238633490903 |
| H | 0.22088597765022 | -2.54445168535122 | 2.76237901776921 |
| H | 0.24923551758036 | -4.13419928029504 | 1.97527168488557 |
| H | -5.53008467112200 | -1.62475189352690 | 3.69283417450427 |
| H | -4.09289122617961 | -0.77803492055017 | 4.29975927659951 |
| H | -4.09975308400682 | -5.55883916703911 | -2.03253331997200 |
| H | -2.47493556168531 | -6.18518494727082 | -2.36552871620918 |
| H | -4.23991993002978 | -0.52372593894338 | -5.28138630314981 |
| H | -5.35748248820832 | -1.24243046951283 | -4.13136895374137 |
| H | 0.34909226489379 | -4.25479246680924 | -5.78799688734385 |
| H | -0.09121975985153 | -2.69328767354867 | -6.50286633234020 |
| H | -1.03991273161897 | -5.46182096933051 | -7.54891362792400 |
| H | 0.22136944818624 | -4.43384917778600 | -8.30622192794775 |
| H | -1.48796527568065 | -3.88960327167175 | -8.26727680934681 |
| H | -5.22677976437283 | -1.98055133500638 | -7.14845936446865 |
| H | -6.46568878564392 | -0.93551291680055 | -6.37660443985569 |
| H | -6.37460732220529 | -2.67658217375362 | -5.96663538878940 |
| H | -5.01720499604476 | -6.82031837206885 | -4.02458289217266 |
| H | -4.18615309387842 | -7.92638694782188 | -2.88811313349718 |
| H | -3.37719287189153 | -7.41861062061025 | -4.40839983914943 |
| H | -7.61051107307818 | 6.22781506480618 | 0.16806581654391 |
| H | -6.70642946177033 | 6.27017600904767 | 1.69504794448239 |
| H | -9.76661613309453 | 5.78320542136514 | 1.45306278975528 |
| H | -8.99076406089977 | 7.31457227861251 | 1.97483667043988 |
| H | -8.85285201538936 | 5.83825822552369 | 2.98664301131339 |
| H | -7.18403435981556 | 1.30464855453285 | -2.44782303938568 |
| H | -8.25859204551415 | 2.72872282746206 | -2.60825948457269 |
| H | -8.84507464327871 | 1.10808748727719 | -3.09693706400785 |
| H | -7.10072226917619 | 1.07341332671218 | 4.66289093561562 |
| H | -6.39823899761720 | 2.70032068046244 | 4.88621368396537 |
| H | -5.43804244680437 | 1.24728818798578 | 5.30470246333912 |

| | | | |
|---|---|---|---|
| H | -5.73030891599642 | 8.65079167872849 | 0.96995131186183 |
| H | -5.93527423513790 | 7.99887439592492 | -0.67180513722894 |
| H | -3.49008256229521 | 9.65087107892798 | 0.29842285288833 |
| H | -4.81549326448654 | 10.26228142419713 | -0.74605463372064 |
| H | -3.69653552370637 | 8.99521964725615 | -1.35088567287453 |
| H | -4.97899671974082 | 2.55120864552903 | -0.35103942641560 |
| H | -4.58754723595380 | 2.23611082303950 | -2.07208150564064 |
| H | -5.45776157986568 | 3.73418922544955 | -1.58877645434659 |
| H | -3.05252206158185 | 3.67555402751439 | 3.96716735451169 |
| H | -4.24415785776050 | 4.90719069503341 | 4.47560089440044 |
| H | -2.73431865080192 | 4.66800751142729 | 5.41728797538731 |
| H | -5.32811654204602 | -6.34753773031360 | 2.53083265899425 |
| H | -3.76122088102760 | -7.18781271703025 | 2.43144380307509 |
| H | -4.92937403571386 | -7.12343824991254 | 0.17178155551970 |
| H | -3.36872311573088 | -6.24350970286910 | 0.12589063336366 |
| H | -4.89237039046135 | -5.32733831643182 | 0.22014540597166 |
| H | 0.33682028727140 | -3.68818558859607 | 5.04170745072404 |
| H | 1.74486087378314 | -4.09964593667764 | 4.01445651952860 |
| H | 0.41124850727099 | -5.27757473679921 | 4.21978210265732 |
| H | -4.17917105366999 | -2.14462653201308 | 6.43603951032028 |
| H | -5.61590413723498 | -3.01320446018489 | 5.81598805135699 |
| H | -5.71331136215207 | -1.25568521610692 | 6.16303343924504 |

200

IDPi Monomer

| | | | |
|---|---|---|---|
| C | 0.24617620555735 | -4.96435276705528 | 2.11504956664619 |
| C | -1.76430531781136 | -0.73151356821914 | -3.79183372293826 |
| C | 0.17699047640170 | -4.72669121630491 | 0.73200278180534 |
| C | -0.84636930291061 | -4.57152267744914 | 2.90995232554413 |
| C | -0.41415062131279 | -0.46589318790298 | -4.11798055922414 |
| C | -2.37220680183110 | -1.90845901582228 | -4.27731538264983 |
| C | -2.58718746883217 | 0.19926165250162 | -2.93429413261253 |
| C | -0.93498432316944 | -4.08700467560480 | 0.13614789390769 |
| H | 1.00726575496821 | -5.07337561316429 | 0.09700767049930 |
| C | -1.95912951881632 | -3.90636801115133 | 2.37348527706382 |
| H | -0.80333763322375 | -4.75904365646773 | 3.99253548402781 |
| C | 0.26443886651500 | 0.77597804454203 | -3.63523904123426 |
| C | 0.31463768461780 | -1.38456272351493 | -4.92625061257451 |
| C | -1.68380809119986 | -2.81938236440363 | -5.09026728859791 |
| H | -3.41760221764653 | -2.10299180418414 | -3.99206113209434 |
| C | -1.99654952958126 | -3.65892271390602 | 0.97016471967038 |
| C | -0.94120494974185 | -3.87205028311528 | -1.36431743843322 |
| C | -3.08402036148718 | -3.41493967102814 | 3.26390520235500 |
| C | 1.30317603396534 | 0.71226548430127 | -2.66151371539177 |
| C | -0.01228846376746 | 2.03059173180682 | -4.16890608441407 |
| C | -0.33726702291367 | -2.53443212292340 | -5.39711401882063 |
| C | 1.74981798010147 | -1.12090403843253 | -5.33976634986417 |
| C | -3.13409115134814 | -2.90594560927813 | 0.36775706017959 |
| O | 1.54860948488871 | -0.53282186245671 | -2.05482912681613 |
| C | 2.11945947299884 | 1.78272073464784 | -2.30660064695232 |
| H | -0.79633489067878 | 2.11932390847154 | -4.93612932159395 |

| | | | |
|---|---|---|---|
| C | 0.68458931387484 | 3.20110850419293 | -3.75287879333943 |
| H | 0.23108669742185 | -3.24017634795811 | -6.02623757896594 |
| C | -3.28949670489249 | -1.51516103233599 | 0.64590100154245 |
| C | -4.03888863195672 | -3.48779299903384 | -0.51170599355940 |
| P | 1.20228917281315 | -0.63606521381364 | -0.46769787943833 |
| C | 1.76806322642017 | 3.08777942569168 | -2.80723766497950 |
| C | 3.34942279968214 | 1.56764572763475 | -1.49428370459838 |
| C | 0.34335634609141 | 4.48893040286143 | -4.26374023968181 |
| O | -2.37645927180687 | -0.94325363475578 | 1.52148900893004 |
| C | -4.32214530405061 | -0.73014633977375 | 0.12935457417421 |
| H | -3.95281603158294 | -4.56144770683241 | -0.73791973630214 |
| C | -5.03868543426909 | -2.72205734119473 | -1.17534564561983 |
| O | 2.09262180586716 | 0.50584989904449 | 0.27544422384729 |
| N | -0.31094714333677 | -0.55360900315204 | -0.10023195975182 |
| N | 1.81076840213373 | -2.18660777717298 | -0.15678197101378 |
| C | 2.43540284436704 | 4.27568999835466 | -2.38694900825540 |
| C | 4.63368609457201 | 1.92665387633119 | -2.04348199595108 |
| C | 3.31029815930010 | 1.01423182363812 | -0.21216519109628 |
| H | -0.48035908637542 | 4.56461206610931 | -4.99022655841980 |
| C | 1.02339000355893 | 5.62345511140163 | -3.84804370771479 |
| P | -1.27158922120410 | 0.15176782768375 | 0.99600945468880 |
| C | -4.49581003181941 | 0.67302154572036 | 0.58894113247649 |
| C | -5.18329614382485 | -1.31996390926598 | -0.86744335661725 |
| C | -5.87646745595924 | -3.30728754791969 | -2.17055528286364 |
| H | 1.22550226159872 | -2.82026773178806 | 0.43176935484252 |
| C | 2.07030549920519 | 5.51418923850654 | -2.89451885921620 |
| H | 3.23765344927758 | 4.20015693726749 | -1.63977468246636 |
| C | 4.80789464280297 | 2.32499224370913 | -3.40261369431603 |
| C | 5.79988153602147 | 1.83848353565067 | -1.20257696760878 |
| C | 4.43040489933240 | 1.01387410384735 | 0.67666584640880 |
| H | 0.74646603995462 | 6.61112516656498 | -4.24620984591951 |
| O | -2.18549410569267 | 1.16027275775766 | 0.08042986673594 |
| N | -0.58276370962961 | 0.88963873402031 | 2.22306981765893 |
| C | -3.43291184967756 | 1.58343223467647 | 0.54947238139410 |
| C | -5.76058442364459 | 1.12314185486272 | 1.11789639859553 |
| C | -6.13462162728680 | -0.56009474102310 | -1.61097537130529 |
| H | -5.76151239732565 | -4.38036451666270 | -2.38764132624280 |
| C | -6.79824511349266 | -2.54177243063272 | -2.86778360516523 |
| H | 2.58902659907759 | 6.42046126939390 | -2.54796590266752 |
| H | 3.93522581762525 | 2.36280008296224 | -4.06886487359964 |
| C | 6.06499335644886 | 2.64095847451993 | -3.89737986015681 |
| C | 7.07525917589218 | 2.18545428334476 | -1.73756703760729 |
| C | 5.64822629647708 | 1.43070531673525 | 0.15034925591855 |
| C | 4.31764375200726 | 0.74219803254313 | 2.14575934207132 |
| C | -3.56874386443749 | 2.96230963702339 | 0.88862311131857 |
| C | -5.92436765230470 | 2.51706321517559 | 1.44721842614632 |
| C | -6.85370425260407 | 0.23977848640720 | 1.36506429875745 |
| C | -6.91688941659117 | -1.15425266530498 | -2.59086582745079 |
| H | -6.23059021987066 | 0.51554505794498 | -1.41082490720423 |
| H | -7.42977712178313 | -3.00403461574927 | -3.64126242973112 |

| | | | |
|---|---|---|---|
| H | 6.17564390048049 | 2.93360279136621 | -4.95241095484255 |
| C | 7.20889031122105 | 2.58277558796797 | -3.05841921961160 |
| H | 7.95474604407274 | 2.12162291796008 | -1.07838511204872 |
| H | 6.52421461563652 | 1.45570968520959 | 0.81614553273051 |
| C | 4.79246144748630 | -0.46306431852744 | 2.72121469166559 |
| C | 3.84529137166138 | 1.79151037530358 | 2.98135650754943 |
| C | -4.82281936579944 | 3.40240529842727 | 1.29632278070283 |
| C | -2.41676608480129 | 3.90723192013018 | 0.81024949059074 |
| C | -7.18089854651501 | 2.97577732735295 | 1.94289947847224 |
| C | -8.05758812065986 | 0.71405359062383 | 1.86507400877515 |
| H | -6.73033659801396 | -0.83347726580945 | 1.16799684580426 |
| H | -7.63217619981175 | -0.54209763544176 | -3.16040213628848 |
| H | 8.19920180799065 | 2.84222388389519 | -3.46177570904294 |
| C | 4.72482723391168 | -0.62257633138149 | 4.11886127432833 |
| C | 5.42919512723600 | -1.55337830749442 | 1.88738519832260 |
| C | 3.78975215461821 | 1.58006229730051 | 4.36830904331349 |
| C | 3.43342701555592 | 3.14038400477458 | 2.42962373233944 |
| H | -4.95839988301309 | 4.46827836173430 | 1.53642269383214 |
| C | -1.93054179096691 | 4.33033556417476 | -0.45111373558366 |
| C | -1.83697663986680 | 4.39755605943773 | 2.01077788862514 |
| H | -7.29170875690740 | 4.04543476023150 | 2.17917398875497 |
| C | -8.23191690705760 | 2.09551132822258 | 2.14289493991399 |
| H | -8.88183473369831 | 0.01033051037079 | 2.05570524981578 |
| C | 4.21272204232813 | 0.37794550715887 | 4.96019059602057 |
| H | 5.07842964302722 | -1.56916563319805 | 4.56152884777160 |
| H | 3.40414310501601 | 2.38960196157109 | 5.00924652378426 |
| C | -0.86559363037059 | 5.24508807259059 | -0.49872251770171 |
| C | -2.57307854776067 | 3.87436704062993 | -1.74222008463323 |
| C | -0.79095506666272 | 5.33148745522613 | 1.91112689336742 |
| C | -2.32849906979987 | 3.89277498945155 | 3.35698483219597 |
| H | -9.19403103560883 | 2.46015923825385 | 2.53296191447784 |
| C | -0.28583778456341 | 5.76298237622053 | 0.67114088361682 |
| H | -0.47822478334563 | 5.54896525407508 | -1.48362603429541 |
| H | -0.32416052142715 | 5.71389142223745 | 2.83044267152900 |
| C | 0.87505011785360 | 6.73240687310884 | 0.59145750217492 |
| C | 0.56807062279797 | 8.01531531241676 | -0.19145960750020 |
| C | -1.53480631921655 | 4.33612450826698 | 4.58272818271869 |
| C | -3.77721476633437 | 4.73436229312610 | -2.15139518126388 |
| C | 1.49114414510979 | -5.54256441387148 | 2.74966946298690 |
| C | 2.34984502518864 | -4.46941335068694 | 3.44038010156941 |
| C | -3.03528840092622 | -3.85235219271443 | 4.72599039848014 |
| C | -1.12079706395216 | -5.16622775585406 | -2.16697999022575 |
| C | -2.33708759289770 | -4.06846446042530 | -5.65232895852280 |
| C | -3.67089043755937 | -4.47095606561711 | -5.02537588317685 |
| C | 1.87677854070812 | -0.12456934338735 | -6.50132415079394 |
| C | -3.65625937169351 | 0.97493011083033 | -3.71456288852145 |
| C | 4.05652694642753 | 0.15991125202509 | 6.44856843917590 |
| C | 2.65757319118581 | -0.35123718555864 | 6.83059241846306 |
| C | 6.95394141216464 | -1.62573189251768 | 2.05296348900425 |
| C | 1.93337630428555 | 3.27769920114159 | 2.14975727744704 |

| | | | |
|---|---|---|---|
| H | -1.92922680067027 | 0.90182312566316 | -2.39157606686427 |
| H | -3.08597112546400 | -0.40764143299955 | -2.15394123014291 |
| H | 0.01074386858390 | -3.39781320034201 | -1.66968140915556 |
| H | -1.72019989508194 | -3.14111301004204 | -1.65020697273115 |
| H | -4.05292919871882 | -3.70874425604035 | 2.80475014676519 |
| H | -3.06852569101114 | -2.30417817549258 | 3.23791443852256 |
| H | 2.21542951094119 | -2.08526115795886 | -5.63036917639941 |
| H | 2.33880409995075 | -0.75495128001641 | -4.47442148078915 |
| H | 5.19344563962858 | -1.39509411851397 | 0.81942359004696 |
| H | 4.98710311326034 | -2.53413591050375 | 2.16261167183489 |
| H | 4.00783550181079 | 3.36070546663345 | 1.50552225909485 |
| H | 3.73369142694099 | 3.91525910205569 | 3.16693690111501 |
| H | -2.89016266244659 | 2.81853633875052 | -1.65566351840173 |
| H | -1.80984977472859 | 3.89849697053416 | -2.54628267149073 |
| H | -2.35337216132727 | 2.78282460850318 | 3.32042629852558 |
| H | -3.39411309438305 | 4.18402019263691 | 3.48304412527514 |
| H | 1.73723628889994 | 6.21452881607461 | 0.11377099439087 |
| H | 1.20543940449012 | 6.98903390565211 | 1.61959736276649 |
| H | 0.27932823973034 | 7.78961778497090 | -1.23839007327580 |
| H | 1.44980686553500 | 8.68753376207660 | -0.22459393991191 |
| H | -0.27153004378213 | 8.57392561387523 | 0.26977171941790 |
| H | -1.55331127260730 | 5.43743022594083 | 4.71631125593661 |
| H | -0.47987743837686 | 4.00533174506627 | 4.52124184321254 |
| H | -1.96137141943804 | 3.87923791143077 | 5.49690340469662 |
| H | -4.21488659354982 | 4.37915929140578 | -3.10724382868746 |
| H | -3.48365829658314 | 5.79605290527027 | -2.28028265856809 |
| H | -4.57356346851611 | 4.69952993534879 | -1.38012902486614 |
| H | 2.09724349458924 | -6.05820574586520 | 1.97644895631815 |
| H | 1.19870293686031 | -6.31329495542611 | 3.49484901291120 |
| H | 3.25082887413149 | -4.91869708495385 | 3.90488904349087 |
| H | 1.78009182433123 | -3.94740535951059 | 4.23443601295536 |
| H | 2.69369125100993 | -3.70613404540175 | 2.71469851559295 |
| H | -2.14277018563838 | -3.43953573199296 | 5.23760594122333 |
| H | -3.01868712466146 | -4.95627129570159 | 4.83658606102609 |
| H | -3.92295573156102 | -3.47144075092225 | 5.26774638951626 |
| H | -2.11328321548330 | -5.62556406472376 | -1.98167575408019 |
| H | -0.35423844928009 | -5.92037652482502 | -1.89846192245325 |
| H | -1.03133675558403 | -4.96223612594675 | -3.25178805239813 |
| H | -1.61544028821744 | -4.91043967815944 | -5.56838529125589 |
| H | -2.47361505992557 | -3.92357219155511 | -6.74814505323997 |
| H | -3.57046873645219 | -4.62594151062250 | -3.93203699659327 |
| H | -4.04557917315842 | -5.41423072285004 | -5.46976406296374 |
| H | -4.45264688659192 | -3.69943849683106 | -5.17579983815553 |
| H | 1.33465978930007 | -0.48705056261060 | -7.39810645300791 |
| H | 2.93955540481443 | 0.02683109344284 | -6.77972633487296 |
| H | 1.45374534039602 | 0.86397136217335 | -6.23171748568374 |
| H | -3.19993733420823 | 1.66497211854245 | -4.45404574293273 |
| H | -4.28279813469064 | 1.57885757474455 | -3.02805570430126 |
| H | -4.33207681053737 | 0.28890749844884 | -4.26392026545225 |
| H | 4.25297963701177 | 1.11664994659813 | 6.97803328081124 |

| | | | |
|---|---|---|---|
| H | 4.82626233509547 | -0.55875063197612 | 6.80228298422461 |
| H | 2.46247263241735 | -1.34746191100199 | 6.38619008156771 |
| H | 1.86636172945633 | 0.33221026212773 | 6.46110590501401 |
| H | 2.55296589565918 | -0.44262904695301 | 7.93100390765594 |
| H | 7.24092748691187 | -1.79326586346466 | 3.11115497898112 |
| H | 7.43761043985514 | -0.68200286154127 | 1.72734405895885 |
| H | 7.38083410021612 | -2.45161040892931 | 1.44821387644748 |
| H | 1.33373490605380 | 3.07943949410379 | 3.05881384924912 |
| H | 1.58043511936448 | 2.55806758850326 | 1.38917648185813 |
| H | 1.69525175005403 | 4.29457967362050 | 1.78756969668561 |
| S | -0.58856557468477 | 0.45242975711892 | 3.78407934416348 |
| S | 3.25148597760604 | -2.87387901373278 | -0.72127340306956 |
| O | -1.89725031933224 | -0.02575927439223 | 4.27787026444021 |
| O | 0.17603192461225 | 1.44238552187440 | 4.56691672668768 |
| C | 0.50963578356038 | -1.10578597243526 | 3.74697615796496 |
| O | 3.65733042680596 | -3.89547764881879 | 0.25249770085985 |
| O | 4.11273838765453 | -1.79131811274858 | -1.21445493152561 |
| C | 2.78717826895968 | -3.86568702809444 | -2.28993984722812 |
| F | 1.80005035178178 | -0.78680921828284 | 3.75207268257404 |
| F | 0.26085639028582 | -1.81520916793479 | 2.61781956672413 |
| F | 0.23514670834488 | -1.88399520978310 | 4.80583693442356 |
| F | 1.93832848384621 | -3.18990425845375 | -3.06290349373536 |
| F | 2.24193711156825 | -5.03815421625735 | -1.94447341411437 |
| F | 3.92553546018606 | -4.08127244800330 | -2.94842451541433 |

400

IDPi Dimer

| | | | |
|---|---|---|---|
| C | 2.54191872345197 | 3.47624676702046 | 3.39077159627883 |
| C | 4.11643265492568 | 3.35478022523195 | -1.93486054052167 |
| C | 3.28009762874609 | 2.59590425250685 | 4.19587031103912 |
| C | 1.78822351725324 | 2.93211822611571 | 2.33356850113208 |
| C | 5.37286618102905 | 3.65224444155890 | -2.54222061972601 |
| C | 2.98324108498765 | 3.21036790076280 | -2.74958388905610 |
| C | 3.92443601645594 | 3.25490138076539 | -0.43183915370575 |
| C | 3.21696084462152 | 1.20035890338470 | 4.02333921345881 |
| H | 3.91652448154387 | 3.00087568554065 | 4.99875115121501 |
| C | 1.71972461972505 | 1.54741075513981 | 2.09870256169332 |
| H | 1.22791235896084 | 3.61586398860611 | 1.67960620150668 |
| C | 6.55729645724138 | 3.78419193152977 | -1.64402494146731 |
| C | 5.43585135383641 | 3.91288823609350 | -3.94200453447754 |
| C | 3.04484642609033 | 3.33298918600704 | -4.14559435919432 |
| H | 2.02450566673831 | 2.96421111785578 | -2.26933279784676 |
| C | 2.39829350253628 | 0.67069143133233 | 2.99085160885129 |
| C | 4.07299946476850 | 0.31534741989539 | 4.90950357111028 |
| C | 0.94502457303057 | 0.99038389680602 | 0.92510660763827 |
| C | 6.92535993685583 | 2.75328294573576 | -0.71456822606754 |
| C | 7.24346296591726 | 4.99012541649415 | -1.54210358810644 |
| C | 4.27283190207509 | 3.71100243220885 | -4.71041357841597 |
| C | 6.65009421938006 | 4.47990248023642 | -4.66710869128789 |
| C | 2.12999317811809 | -0.80302435971368 | 2.97349171333219 |
| O | 6.33425903271501 | 1.49667933236334 | -0.92840576758292 |

| | | | |
|---|---|---|---|
| C | 7.73401288139346 | 2.99132873999678 | 0.39989123936222 |
| H | 6.95926310214594 | 5.81596462611490 | -2.20927751702924 |
| C | 8.26371295509734 | 5.21519798523571 | -0.58124515793990 |
| H | 4.33265614578258 | 3.86788266207168 | -5.80011333574686 |
| C | 2.58905606914814 | -1.70013573266503 | 1.95556022955182 |
| C | 1.38974657449211 | -1.33442523050801 | 4.02303076887399 |
| P | 6.84896274455658 | 0.02341072680820 | -0.51950466726058 |
| C | 8.50552933432450 | 4.21447547707001 | 0.42439867817625 |
| C | 7.85828365824188 | 2.07252123676197 | 1.56742632740575 |
| C | 9.02059670588958 | 6.42384790778388 | -0.56394237879140 |
| O | 3.28186650222458 | -1.07108715577480 | 0.92812779336250 |
| C | 2.31559835130762 | -3.08003413608596 | 1.97178953342186 |
| H | 0.99416646947407 | -0.65130899972299 | 4.79006709392796 |
| C | 1.18449940184021 | -2.73122443106146 | 4.17129447933904 |
| O | 8.33203468681503 | 0.20856962456488 | 0.13469365606086 |
| N | 5.86321429830920 | -0.74216931205904 | 0.43931878872213 |
| N | 7.21062442838025 | -0.78360742890517 | -1.95210979210320 |
| C | 9.53488763256936 | 4.45570179784743 | 1.38238759570588 |
| C | 7.59809630890238 | 2.53618628186224 | 2.90886278774272 |
| C | 8.32737865265767 | 0.77206428051022 | 1.42487807931941 |
| H | 8.81107210615291 | 7.18401595394771 | -1.33229667300445 |
| C | 10.00583139403347 | 6.63278484705269 | 0.38791282999657 |
| P | 4.55744441092841 | -1.62130101969161 | 0.06980459477571 |
| C | 2.63531528770529 | -4.00874971362352 | 0.84673459474759 |
| C | 1.70162127344905 | -3.62347785739901 | 3.16668015168032 |
| C | 0.51589522082436 | -3.26272369497190 | 5.31454485384048 |
| H | 7.13168036038475 | -1.81666531080773 | -1.95571441993031 |
| C | 10.27088782621771 | 5.63213056780078 | 1.35904003647418 |
| H | 9.76140051456591 | 3.68863673074021 | 2.13504514974713 |
| C | 6.91590650407668 | 3.75865871525609 | 3.17380994831296 |
| C | 8.00236984075911 | 1.71078701665683 | 4.02190248645872 |
| C | 8.83590556637060 | -0.02168656125758 | 2.48957404042580 |
| H | 10.58950007353326 | 7.56551096634105 | 0.38649151576119 |
| O | 4.85368132619629 | -3.09836854687031 | 0.71515664570603 |
| N | 4.21839766451580 | -1.81223852961114 | -1.48389367541614 |
| C | 3.91627171545382 | -4.05793721428276 | 0.30056450557536 |
| C | 1.64877247020269 | -4.91586359165869 | 0.29867377270180 |
| C | 1.61389198690052 | -5.02618701791202 | 3.41771883985876 |
| H | 0.10616277970806 | -2.55863393002220 | 6.05574788484872 |
| C | 0.41084303100058 | -4.63139140357741 | 5.50397081366816 |
| H | 11.07026242296640 | 5.78704886528897 | 2.09919477286614 |
| H | 6.57623224364116 | 4.37357912904812 | 2.32950043360422 |
| C | 6.64337039805050 | 4.15061318674892 | 4.47627682582550 |
| C | 7.72185727130180 | 2.15423592250242 | 5.34769217590408 |
| C | 8.67584823499351 | 0.47864168487929 | 3.77620744905982 |
| C | 9.61824305677463 | -1.25864891575206 | 2.18277518409503 |
| C | 4.34832574578319 | -5.00742023700391 | -0.67253902498337 |
| C | 2.05534549780242 | -5.88127764038469 | -0.69369036767563 |
| C | 0.27911451576432 | -4.89652874118812 | 0.68568030102511 |
| C | 0.99278829823993 | -5.51558884040279 | 4.55778196718929 |

| | | | |
|---|---|---|---|
| H | 2.05994070151204 | -5.72784837681817 | 2.70083196718150 |
| H | -0.09760129493403 | -5.03323045425742 | 6.39316897158395 |
| H | 6.09339151845990 | 5.08627335604733 | 4.65741941885421 |
| C | 7.05452145088385 | 3.34867264677304 | 5.57334109816781 |
| H | 8.03370950145758 | 1.51870267681544 | 6.19079078051779 |
| H | 9.06552460322778 | -0.10098852540608 | 4.62694402127766 |
| C | 10.98370703389771 | -1.11872061102080 | 1.80811704738886 |
| C | 9.02118644067872 | -2.53957342026086 | 2.24928622440205 |
| C | 3.40769084902204 | -5.91746165739894 | -1.13154368167666 |
| C | 5.76507199616747 | -5.00341238042784 | -1.14049226283456 |
| C | 1.09387759137885 | -6.79340030993461 | -1.22117325117468 |
| C | -0.63314740000491 | -5.80318460429686 | 0.16384453627031 |
| H | -0.06261770833516 | -4.15474245014986 | 1.41475581323655 |
| H | 0.95802970074450 | -6.60133859857364 | 4.73194302762676 |
| H | 6.83519200082198 | 3.67276096987202 | 6.60178508182901 |
| C | 11.71855361094923 | -2.27398761339838 | 1.49235579813594 |
| C | 11.66305412481875 | 0.23498450444929 | 1.71825219437371 |
| C | 9.80767808869466 | -3.66694373030961 | 1.94512761253347 |
| C | 7.56896167932587 | -2.72793174825050 | 2.62468856569083 |
| H | 3.70960342447741 | -6.67925726395228 | -1.86608329359986 |
| C | 6.78748179518604 | -5.41431196004391 | -0.24888058543557 |
| C | 6.08362583675460 | -4.58566761336045 | -2.46494930350095 |
| H | 1.42535842788828 | -7.52281361724462 | -1.97648994489233 |
| C | -0.22469588458966 | -6.76438636830560 | -0.79642282232528 |
| H | -1.67824914981186 | -5.76334243907019 | 0.50016168884772 |
| C | 11.15018120401831 | -3.55977480877940 | 1.55111254278195 |
| H | 12.77543108042968 | -2.16499303584519 | 1.19542365694184 |
| H | 9.34436831526990 | -4.66414759348489 | 2.00726679762044 |
| C | 8.12502542345516 | -5.35851013714377 | -0.67955307923858 |
| C | 6.46976704044660 | -5.99214801649336 | 1.11610239582319 |
| C | 7.44142700258979 | -4.49708449237320 | -2.82797852330440 |
| C | 4.97679475480268 | -4.29402388063412 | -3.46188166587955 |
| H | -0.95708689291459 | -7.47661620140600 | -1.20551453373205 |
| C | 8.47648607361383 | -4.87857249082456 | -1.94941569528505 |
| H | 8.91935397414662 | -5.69574642655726 | 0.00382116697287 |
| H | 7.70257117624821 | -4.14299115917508 | -3.83627174737903 |
| C | 9.92719325503183 | -4.73385354931136 | -2.34761784296669 |
| C | 10.39534861943244 | -3.27488805485654 | -2.27729827473671 |
| C | 5.38392940988225 | -3.64303230271726 | -4.77983730417188 |
| C | 6.02246235582024 | -7.46018929571244 | 1.05370340390885 |
| C | 2.51619361530381 | 4.96303475465279 | 3.67301204953936 |
| C | 1.20561475477759 | 5.43443211164249 | 4.32058684320663 |
| C | 0.07068028054554 | 1.97821557474895 | 0.16432913019325 |
| C | 5.15220986266582 | -0.45263874028878 | 4.13579367713641 |
| C | 1.83144566925293 | 3.02704543213925 | -4.99398343608689 |
| C | 1.47682106262445 | 1.53246110339292 | -4.98969996406873 |
| C | 7.94504619090295 | 3.66484163550786 | -4.68173354463595 |
| C | 4.22351562491667 | 4.53831925379951 | 0.35382279604928 |
| C | 11.97986457318376 | -4.78759215045793 | 1.24729696367132 |
| C | 12.85260788648906 | -5.22584913550426 | 2.43278673440359 |

| | | | |
|---|---|---|---|
| C | 11.65847580269445 | 0.84539481579934 | 0.30808760159336 |
| C | 7.36057981213361 | -3.41651030810544 | 3.97863194216150 |
| H | 4.50849958183031 | 2.41275648718920 | -0.01446504904463 |
| H | 2.87331274823202 | 2.96547545503905 | -0.24869418566220 |
| H | 3.44201005125806 | -0.40735075265382 | 5.46908522535359 |
| H | 4.55715116209182 | 0.95796045432346 | 5.67314589376817 |
| H | 0.33125314383061 | 0.13505422811822 | 1.27813121948297 |
| H | 1.67437773760712 | 0.54481862064692 | 0.21761767366611 |
| H | 6.86950983039911 | 5.49114875993136 | -4.25981250356802 |
| H | 6.33945495742238 | 4.66464208664154 | -5.71610336612622 |
| H | 12.71446247972767 | 0.12018338269056 | 2.05693600074688 |
| H | 11.18649608217430 | 0.94314637324543 | 2.42660019444578 |
| H | 7.07548182859854 | -3.32409978572666 | 1.83098370747191 |
| H | 7.05095458511094 | -1.75135324551493 | 2.62011845212222 |
| H | 5.68591695851000 | -5.39011492033832 | 1.61923942888913 |
| H | 7.37147302654283 | -5.91071596096854 | 1.75830061216843 |
| H | 4.22039785096120 | -3.66094665318642 | -2.96198660912273 |
| H | 4.45377447955315 | -5.25279529538090 | -3.67450281008569 |
| H | 10.07024808283147 | -5.11651755858186 | -3.38101965927521 |
| H | 10.55540347478294 | -5.36553359075794 | -1.68523161115555 |
| H | 11.46134784243071 | -3.17303268897339 | -2.56691834394553 |
| H | 10.27898145123809 | -2.86442985281468 | -1.25303580555238 |
| H | 9.79790202537128 | -2.64651741515527 | -2.96467040153365 |
| H | 6.07070923359348 | -4.28094167196702 | -5.37369384488847 |
| H | 5.87930566614991 | -2.66796865968701 | -4.61817660024549 |
| H | 4.48439023701685 | -3.44954549790081 | -5.39524102102910 |
| H | 5.09597743438507 | -7.56770808348926 | 0.45481208684320 |
| H | 5.82046334520873 | -7.86001963218330 | 2.06819026850683 |
| H | 6.80142420692401 | -8.09345137691570 | 0.58288916833063 |
| H | 2.67294636538095 | 5.51926943466566 | 2.72442472493244 |
| H | 3.37209551469425 | 5.22487091459336 | 4.33063874914724 |
| H | 0.33363131554241 | 5.18941315356727 | 3.68073256910215 |
| H | 1.20867476082882 | 6.53157094524054 | 4.48386930555219 |
| H | 1.03943541689626 | 4.94133431245042 | 5.29914061434818 |
| H | 0.66065896995751 | 2.81409611119122 | -0.26036276419330 |
| H | -0.71594488917214 | 2.42473134910660 | 0.80364980472149 |
| H | -0.43487615668916 | 1.47745404443152 | -0.68069478114368 |
| H | 4.71329750037348 | -1.23989920352546 | 3.49253706450194 |
| H | 5.86169036287891 | -0.94996396248103 | 4.82700605923277 |
| H | 5.73135083074076 | 0.22449209482601 | 3.47925359893575 |
| H | 2.00694070579000 | 3.37537476094997 | -6.03343596023946 |
| H | 0.96007781529256 | 3.60599930686374 | -4.61372845309816 |
| H | 1.25359361113963 | 1.18088377654243 | -3.96394241755500 |
| H | 2.32214920628225 | 0.91574263921349 | -5.35354023709098 |
| H | 0.58754221544097 | 1.32941189617446 | -5.62071665865867 |
| H | 8.72418579423302 | 4.19362560927314 | -5.26785376477949 |
| H | 7.78156245936844 | 2.67200702027956 | -5.13831887240198 |
| H | 8.34389825771771 | 3.51032822809841 | -3.66262277339749 |
| H | 3.54006066210526 | 5.35576714720715 | 0.05196997383158 |
| H | 5.25870458049011 | 4.89969599193829 | 0.20398846822209 |

| | | | |
|---|---|---|---|
| H | 4.08205543711573 | 4.35604503630238 | 1.43551486866478 |
| H | 12.62595900025311 | -4.58386136544242 | 0.36637267818113 |
| H | 11.30838366754269 | -5.62202044496944 | 0.95350823105989 |
| H | 13.45417854322217 | -6.12325101804008 | 2.18262287674825 |
| H | 12.22871155498229 | -5.46586778038352 | 3.31760645961267 |
| H | 13.55204858144650 | -4.41858617943827 | 2.73135889576948 |
| H | 12.20040398094955 | 1.81288696352231 | 0.29479517072928 |
| H | 10.63119858154052 | 1.02980204125346 | -0.05698854868272 |
| H | 12.14667344315882 | 0.16743162040503 | -0.42138257011527 |
| H | 7.78129057133332 | -2.81210772570005 | 4.80884627331091 |
| H | 7.85234878385344 | -4.41042825380907 | 4.01235582317215 |
| H | 6.28068936733109 | -3.56775883932322 | 4.17916427266076 |
| S | 3.22509323129624 | -0.90387652544800 | -2.37929168939690 |
| S | 7.61332738822186 | -0.16427513075374 | -3.49078342875491 |
| O | 3.62425734261001 | -1.00009460630949 | -3.79380198163521 |
| O | 2.90934275961979 | 0.40666819149714 | -1.76375923752312 |
| C | 1.57745673979617 | -1.84813817416129 | -2.28691610261816 |
| O | 7.87487807048775 | -1.33737227771208 | -4.33474360510647 |
| O | 6.69715315016026 | 0.91857707835899 | -3.84072958828549 |
| C | 9.32291958623348 | 0.62325757043366 | -3.15761821349672 |
| F | 1.11582712942636 | -1.88446849946247 | -1.02173310472077 |
| F | 1.70496531259391 | -3.11166019400036 | -2.72945587511764 |
| F | 0.67418280844896 | -1.21481463703751 | -3.05326288706093 |
| F | 9.19193740715264 | 1.66444522778005 | -2.32466734480443 |
| F | 9.80660484415374 | 1.03681875505511 | -4.32843298798047 |
| F | 10.14864116278906 | -0.26950040912589 | -2.61577870478843 |
| H | -10.16547034750689 | -4.52658936685244 | -5.12734646085083 |
| H | -8.75495590500888 | -6.43735910195227 | -5.91597566221341 |
| H | -11.17977984816794 | -4.18177589780103 | -0.06155459891230 |
| H | -11.12691567474083 | 2.81027267138550 | 1.49947153497928 |
| H | -9.25353767222674 | -2.62337744279726 | -0.49349640499700 |
| C | -9.09316832563879 | -4.57065944832620 | -4.88164800012383 |
| H | -12.34158227507076 | -2.83588865005765 | -0.31166937131019 |
| H | -10.89895771824139 | 3.86217331586272 | 0.08257790880806 |
| C | -8.31271929063088 | -5.63108749913980 | -5.31182280296710 |
| C | -11.33712602371924 | -3.21735015453188 | -0.58640051713519 |
| C | -11.66427778391625 | 3.33509999703996 | 0.68559459111236 |
| H | -10.43872022182564 | -2.49383841081104 | -3.80266987078784 |
| H | -12.33180619840667 | 4.09594468564226 | 1.13924032261492 |
| C | -10.24201049159700 | -2.20626652651747 | -0.22053547555699 |
| H | -11.80821593953796 | -0.19363004416147 | 0.66223069439137 |
| C | -9.36385701811550 | -2.48973342871304 | -3.56663654297276 |
| C | -11.30661563588504 | 0.07511441344979 | -0.28240980576095 |
| C | -8.53735768311979 | -3.52206861385411 | -4.09012172868193 |
| H | -11.34606072574544 | -3.42136550470633 | -1.67600040415880 |
| C | -10.42895529740881 | -0.85208225878280 | -0.87018141773508 |
| C | -6.94244336193767 | -5.68651275537500 | -4.94700896190299 |
| C | -12.45233066951468 | 2.34264855126550 | -0.18110234707469 |
| H | -10.22782941498488 | -2.06978905747145 | 0.88009957034527 |
| H | -7.13923833486427 | 0.06348803689686 | -7.71757420485746 |

| | | | |
|---|---|---|---|
| H | -8.49744532233818 | 3.04999057367377 | -3.06583808505080 |
| C | -11.55401646153599 | 1.33221553663953 | -0.85700963513031 |
| H | -6.32841148424955 | -6.54571452111492 | -5.25629185934563 |
| C | -9.76894844276250 | -0.50244342284863 | -2.07706877720725 |
| C | -8.86525371746706 | -1.50198845173315 | -2.72755133307566 |
| H | -13.20317861085541 | 1.81314681056505 | 0.44287531344030 |
| C | -10.88513837109872 | 1.65362046302797 | -2.04872882029263 |
| C | -6.71392648684294 | 0.52790175540175 | -6.80460141425478 |
| C | -9.99550966665311 | 0.76217331621889 | -2.67282309126663 |
| C | -7.13052501625479 | -3.54318643869082 | -3.76931309888052 |
| H | -6.47938306985003 | -0.28265848363544 | -6.08448669067086 |
| C | -8.13656645336134 | 2.17881218989138 | -3.64421395027420 |
| H | -13.02320959891935 | 2.89982018095564 | -0.95472457912718 |
| C | -6.36667448074592 | -4.67100279018327 | -4.19717272292181 |
| H | -4.72781950551872 | 0.71902998254326 | -7.66267513198443 |
| H | -11.05845081645085 | 2.63842411849427 | -2.51383397855678 |
| H | -10.01792302629605 | 1.68543381992490 | -4.60696394619182 |
| H | -7.67328177362489 | 2.54536117455271 | -4.57999989478327 |
| C | -9.28046990466350 | 1.19874853323455 | -3.93228228525526 |
| C | -5.46335279750233 | 1.35873074095490 | -7.12975776934791 |
| H | -3.50280937849860 | -2.29635527836830 | -7.01526099774771 |
| H | -5.33082733307669 | -2.08638359085931 | -5.34338511726061 |
| H | -7.33755269118028 | 1.70881052458404 | -3.03913096345699 |
| H | -7.50200126497409 | 1.15318360697059 | -6.34157769471787 |
| H | -5.73758080884846 | 2.17204938985455 | -7.83508693543073 |
| C | -7.46108956297203 | -1.50663944961927 | -2.48145058205874 |
| C | -6.57191078139074 | -2.45556198054985 | -2.99405701739072 |
| C | -3.29954494422709 | -2.50317121721698 | -5.95366094311382 |
| C | -4.32349273073301 | -2.38779203014248 | -5.02394624083848 |
| H | -5.30793170250776 | -4.74224000251862 | -3.91924221431890 |
| O | -7.73759825652290 | -0.44086682738790 | 3.41566670783111 |
| H | -8.89562319953762 | 0.31525945651053 | -4.48130428876387 |
| C | -4.80170289856438 | 1.95276566217274 | -5.90486551261849 |
| O | -6.96146734971301 | -0.43875253862440 | -1.72916437291473 |
| F | -6.97968477301522 | -3.73532562805521 | 3.27636054899962 |
| H | -3.46341143654993 | 0.27069584847425 | -5.61918283989129 |
| C | -1.99117803198941 | -2.87117162854295 | -5.54317836834548 |
| H | -5.91011067461655 | 3.81708977907182 | -5.95666867217630 |
| C | -4.08421173898799 | -2.62430108142790 | -3.64012408921968 |
| C | -5.11890153586140 | -2.47811394948698 | -2.64660875822223 |
| H | -1.18588425615743 | -2.95633669372489 | -6.28794161034661 |
| C | -3.79457885925196 | 1.24469098353005 | -5.22635013819570 |
| C | -5.16375409021055 | 3.21637704171594 | -5.40977734341702 |
| F | -8.95486552220092 | -2.88436737017832 | 2.90836401725687 |
| S | -6.92918889363389 | -1.20989249550026 | 2.46263031746403 |
| C | -7.69861414507481 | -2.96359510919493 | 2.45931224056057 |
| O | -7.88460688479067 | 3.49671512994924 | -0.65077541746853 |
| F | -7.10798031734623 | 4.80043269285030 | 2.37235301279555 |
| O | -5.47601536570347 | -1.40043642569323 | 2.56098290485625 |
| F | -9.01601644962054 | 5.06449257738090 | 1.35842806459442 |

| | | | |
|---|---|---|---|
| C | -1.72710752221307 | -3.11793974413720 | -4.20406779717029 |
| C | -2.75385413745984 | -2.99822646890147 | -3.22227219063941 |
| P | -6.16431446737266 | -0.61799289604478 | -0.33038987235947 |
| C | -4.72439113891025 | -2.54859952369538 | -1.30739865872561 |
| O | -5.64073259358145 | -2.16135209403813 | -0.31077704539662 |
| N | -7.32654403396868 | -0.66168159748432 | 0.89804442096234 |
| C | -3.15801547004858 | 1.75567000886831 | -4.08220207356243 |
| C | -4.57002663715941 | 3.76564672265599 | -4.25678179803258 |
| H | -4.11023279205491 | 5.61785917101889 | -3.25514034792220 |
| C | -8.20889932414299 | 4.17530002918966 | 1.94734817604925 |
| H | -1.13833372601609 | 1.63772843102782 | -3.32487565362663 |
| N | -6.27794763800321 | 2.46833302457107 | 1.13919554816964 |
| S | -7.76000407176332 | 2.83121212033854 | 0.66199965851727 |
| H | -2.54042306373084 | 1.02845286637904 | -1.33502080656182 |
| F | -7.69333611312614 | -3.49092905176925 | 1.23509329013942 |
| H | -7.98205194386687 | 0.19165637442346 | 0.92743169576266 |
| C | -2.01595832743693 | 0.97228935319322 | -3.46800484943344 |
| C | -4.95162569516712 | 5.16771808021883 | -3.82030429957934 |
| H | -6.83781580465787 | -5.77099780326482 | 0.40363054711716 |
| C | -3.58311838685370 | 3.01051706735328 | -3.56982037057196 |
| H | -0.71628407509388 | -3.39984622928042 | -3.87563356308073 |
| H | -4.58348326824216 | -6.74553137905500 | -0.26122098510577 |
| C | -2.51174019539479 | -3.30871743944473 | -1.85197158720218 |
| N | -5.04540293642050 | 0.48496508605805 | -0.30142649213873 |
| H | -6.60587274109360 | -4.45969262696036 | -0.78377467496711 |
| C | -3.48373587877753 | -3.11036709617217 | -0.87785122166398 |
| C | -2.35144394697330 | 0.29489694805604 | -2.13952076720112 |
| C | -6.32349781022784 | -5.50022093471163 | -0.53962807574119 |
| P | -5.00342804179823 | 1.96901568042736 | 0.31263204228800 |
| H | -5.07386617704788 | 5.78993799567472 | -4.73306886856151 |
| F | -8.84535106074346 | 3.62635574444358 | 2.98926155124659 |
| C | -4.80422560765310 | -5.66700954716625 | -0.41384957094681 |
| H | -6.43664788003543 | 6.30629801382862 | -2.68777918245162 |
| O | -4.61805925064618 | 3.01026996738660 | -0.89618937593205 |
| O | -8.68699807156630 | 1.68573024810858 | 0.95508599579535 |
| C | -6.23247686811651 | 5.25561880879898 | -2.97915225629743 |
| H | -1.69339741376565 | 0.19897283990972 | -4.19536622935117 |
| H | -1.51878211074179 | -0.35651280616264 | -1.81177519582177 |
| H | -1.54737259851211 | -3.75424774285959 | -1.56331869408904 |
| H | -4.32866523102345 | -5.39886243774765 | -1.37907899174568 |
| H | -3.26232062097030 | -0.32458994547841 | -2.22690769003490 |
| C | -2.86917559909083 | 3.63125122833145 | -2.41157059119247 |
| H | -4.52258325643350 | -1.72107928796662 | 7.39817831598618 |
| H | -7.11006149144515 | 4.88792675460019 | -3.54588680275006 |
| H | -6.71811922228522 | -6.14935681027111 | -1.34714893758071 |
| C | -3.38642937519359 | -3.70442974333383 | 0.49236926444542 |
| C | -3.39627923771014 | 3.65422181706062 | -1.08041131994784 |
| C | -4.11483584389981 | -4.91039294578773 | 0.71289594336256 |
| H | -0.88570786528353 | -1.96027916441670 | 2.07326796168437 |
| O | -3.71621612749484 | 2.02698731881079 | 1.32383366547095 |

| | | | |
|---|---|---|---|
| H | -3.17073381457442 | -2.36188583580107 | 6.43735925726545 |
| H | 0.42305013206265 | 2.41995812949971 | 4.70453543216846 |
| C | -4.18048681800127 | -1.90571310503580 | 6.35554354034498 |
| H | -6.17130806500274 | 4.64923857655140 | -2.05637112308557 |
| H | -1.25816532265575 | 4.28560538267993 | -3.66811949804318 |
| C | -1.66313127065801 | 4.27858169694589 | -2.64447886950742 |
| H | -0.82467514008696 | 0.60544319471090 | 3.50938761357113 |
| C | -4.06967809741853 | -0.58446382649139 | 5.62606334128760 |
| H | -1.97176572385137 | -0.80748804836683 | 5.13491970940392 |
| C | -2.86747297243132 | -0.17056020420386 | 5.03906154026438 |
| C | -1.66531781863261 | -1.98260724931287 | 1.28238791027659 |
| C | -2.54805967102715 | -3.18719280824301 | 1.51111731757654 |
| H | -6.78776973060412 | 2.06621087291951 | 3.56904253589747 |
| C | -0.59895243755487 | 2.14233708570240 | 5.02662289133686 |
| C | -5.19076077963232 | 0.25501362663436 | 5.49547096978454 |
| H | -6.14120656901729 | -0.05362033119652 | 5.95408343867588 |
| H | -1.11195045567141 | 3.05889471148848 | 5.38409815654474 |
| C | -2.76010522656268 | 1.04662018546136 | 4.33901566971477 |
| C | -1.38590081985803 | 1.48680035066026 | 3.88295321479215 |
| C | -3.61798897895792 | 3.22347305162265 | 2.04942279120047 |
| C | -4.08234768326443 | -5.49512211059715 | 1.99148974685330 |
| H | -6.15959820406403 | -2.46349330574198 | 5.59707191989362 |
| C | -2.77334494233994 | 4.34210344125679 | -0.03069912316800 |
| H | -1.12389510783018 | -2.08771381271353 | 0.31947963599160 |
| C | -5.14805670027788 | 1.46017675022563 | 4.77608438708226 |
| C | -5.13844425811022 | -2.88856182443900 | 5.67143940115620 |
| C | -3.91219216706604 | 1.85885732453895 | 4.18971473948337 |
| C | -6.40504828630725 | 2.28624045564373 | 4.58743804263625 |
| H | -1.80423402438778 | 0.19869134346466 | 1.13933645999745 |
| C | -2.45621079039501 | -0.68036814304923 | 1.28381898343424 |
| H | -4.64730838372331 | -6.42791945810294 | 2.15812756148337 |
| H | -3.19290299123966 | -0.67183355577441 | 0.46384789173993 |
| C | -3.33175028186183 | 4.39861508729686 | 1.34993100910409 |
| C | -0.93978444398267 | 4.93251666553056 | -1.61360517852339 |
| H | -6.13860947971519 | 3.36080332975613 | 4.55937728993240 |
| H | -4.80356028763327 | -3.11193276095643 | 4.64058979001831 |
| C | -3.79699090001789 | 3.16191949702674 | 3.46360088115865 |
| H | -0.51662330096507 | 1.45375293629638 | 5.89305978487371 |
| C | -2.53388954394953 | -3.82225975531315 | 2.76664662247305 |
| C | -1.48552643097708 | 4.95600050154990 | -0.28098370931932 |
| H | -1.44964131167465 | 2.18979410219190 | 3.02990014282809 |
| C | -3.30891445892088 | -4.96210073900912 | 3.03771795504175 |
| H | -5.20985767527950 | -3.84178139672357 | 6.23361926432770 |
| H | 0.71215374730012 | 5.52585996564161 | -2.90522274544294 |
| C | 0.31561746499115 | 5.55447126340553 | -1.87898438519361 |
| C | -7.52490884338142 | 2.05642355945113 | 5.60009753650725 |
| H | -7.17375933450117 | 2.19736860906902 | 6.64353998269547 |
| C | -3.48695282834116 | 5.64713448082980 | 2.05192157363121 |
| H | -3.01709983046397 | -0.54797450376945 | 2.22632684938320 |
| H | -3.29730588755054 | 6.93067529816783 | 0.29945029871518 |

H -1.87769045685633 -3.41847990835833 3.55467243598639
H -7.95083609082969 1.03791665614772 5.51253129484012
C -3.80389423210962 4.37303881326612 4.14836265524592
C -3.44225248578354 6.90848881348425 1.38871675700691
C -0.69493761843094 5.55593015708524 0.74550516880913
C -3.23516801348649 -5.65942936843549 4.37795594264942
H -2.40633890857321 -7.46622731706351 5.31374049561867
C -3.71963800007493 5.62388799540922 3.47465603394833
H -4.26454936120150 -5.84469710920220 4.75352265554785
H -8.35277969282040 2.76904777892296 5.41816256487904
C 1.03105389210321 6.17092300353502 -0.86616416030168
H -3.91138864915198 4.36209916397183 5.24404078884949
C -2.46399480630416 -6.98555130644705 4.31553643357876
H -1.05304094481673 5.53839577124608 1.78229079432709
H -2.94979694233829 -7.70280980380664 3.62301501188215
C 0.52484921747003 6.15267203950935 0.45903203198546
C -3.59628260863030 8.09100078646213 2.09712903768648
H -2.75187984426297 -4.98251894145170 5.11255529048983
C -3.85078164129788 6.85900747978192 4.17605713374064
H 1.99584648178968 6.65113216088989 -1.08538968847180
H -3.57373661469213 9.05299997425868 1.56331891261031
C -3.78877939965769 8.06977819608134 3.50424379590854
H -1.43070074331616 -6.82057080211637 3.95036261680033
H -4.01773568074074 6.82893319760173 5.26394539293804
H 1.10600453383812 6.60973172732187 1.27378606947818
H -3.90138305806670 9.01509751602165 4.05616684718387
233
X-TBS (xTB)
P -0.66901539166255 -0.04435863068277 -1.29576850442843
N -1.13487422275255 -1.04259435796603 -2.45249252184694
S -0.91496876166070 -0.65362802518258 -3.95829416151606
O 0.42710963701308 -0.30752083207831 -4.33411127313939
O -1.96267640203079 0.12884506269674 -4.55615990129256
C -1.17170650565818 -2.37516402037546 -4.69353348504435
F -0.40059436984061 -3.29645117677720 -4.10973974749045
F -2.43574009025005 -2.79005330454325 -4.57467368328492
F -0.87710081363281 -2.38954093042984 -6.00202027089528
N -0.14380304739480 -0.90903463425118 -0.05408061775529
P 0.70831182157828 -0.35164738243157 1.15131613887654
N 1.02538923451504 -1.56409846145650 2.19508726648320
S -0.18275193628142 -2.18331087107953 2.89960733261013
O -1.13342886207040 -1.35042935832655 3.54116136801508
O -0.83216314565812 -3.34344441725654 2.21769255414360
Si -0.66029541747152 -4.41057202487463 0.85504288769246
C 1.17176005046643 -4.33505975195807 0.40549039456650
H 1.81637019888472 -4.55917219078099 1.25210375402298
H 1.39870661424710 -3.32104677461101 0.08301191865970
H 1.43429891426851 -5.01112073715166 -0.40307318693808
C -1.83776808207931 -3.80293608676485 -0.49316065064678
H -2.88398822980388 -3.86330933300783 -0.20677252905271

| | | | |
|---|---|---|---|
| H | -1.62009520406607 | -2.76973768809061 | -0.75876456263884 |
| H | -1.71359722436324 | -4.39724384012413 | -1.39375835983026 |
| C | -1.20155255931664 | -6.10075212028344 | 1.64405680335051 |
| C | -0.08793353980069 | -6.69616728254322 | 2.51941686422864 |
| H | 0.15413099422866 | -6.03272738899778 | 3.34521132408054 |
| H | 0.81240820986596 | -6.87045310939776 | 1.93625236956237 |
| H | -0.41597821011348 | -7.64782382715267 | 2.93506058122294 |
| C | -2.46504992810479 | -5.94791348785992 | 2.50295630831330 |
| H | -2.29378932702399 | -5.24437657506779 | 3.31464252279609 |
| H | -3.30145681286370 | -5.59438087797542 | 1.90471172059819 |
| H | -2.73978458516249 | -6.91041784725598 | 2.93234176301559 |
| C | -1.50248249925795 | -7.09122657309550 | 0.51137768747068 |
| H | -2.32332685880879 | -6.73499368518417 | -0.10411728636967 |
| H | -0.63151287868787 | -7.22316128309129 | -0.12442632282947 |
| H | -1.77847782782289 | -8.05971496864285 | 0.92386767368505 |
| C | 0.70748537328432 | -3.09865487986673 | 4.28535506067435 |
| F | -0.13615201851811 | -3.82860252957181 | 5.01692246836398 |
| F | 1.30602564156549 | -2.22976179964854 | 5.09845151282706 |
| F | 1.63870020385104 | -3.91867141473162 | 3.80745290036847 |
| O | 2.09690643444300 | 0.25390092708980 | 0.70424129769626 |
| C | 2.94293431939683 | 0.50332462234382 | 1.80368758994322 |
| C | 2.68804784691542 | 1.61439020055108 | 2.57354272010848 |
| C | 3.45411116324539 | 1.83016416341621 | 3.75967951170141 |
| C | 3.14871180665633 | 2.83333309876857 | 4.70521627223732 |
| C | 3.93586311356663 | 3.02114296518769 | 5.80816117486313 |
| C | 5.06570415644576 | 2.21807649734761 | 6.02612227185394 |
| C | 5.35561262848243 | 1.20203952371950 | 5.16027022919034 |
| C | 4.54617600304614 | 0.96346320782739 | 4.02759604086117 |
| C | 4.77534084777628 | -0.14154542324416 | 3.18863393856670 |
| C | 3.96073040705692 | -0.42684650245647 | 2.11476830100356 |
| C | 4.11562067103355 | -1.69162237148578 | 1.38370042282288 |
| C | 4.37029416124726 | -2.84391997733932 | 2.13362810301623 |
| C | 4.49752448008458 | -4.08132793388304 | 1.53393499680099 |
| C | 4.33776069430628 | -4.17120386064562 | 0.16032569221872 |
| C | 4.08222433627828 | -3.02284852617593 | -0.60366912913532 |
| C | 3.99463839501883 | -1.78354538324797 | -0.00518637098148 |
| H | 3.83075059012417 | -0.89409332768473 | -0.59478072193536 |
| C | 3.91642185550893 | -3.38398980438207 | -2.05918863119489 |
| C | 4.00276160725177 | -4.89217553857301 | -2.01588327422578 |
| C | 3.83151160426328 | -5.81567784446416 | -3.02677288965020 |
| C | 3.98487518356400 | -7.16643058852618 | -2.73913793254393 |
| C | 4.30092223202047 | -7.59273842785576 | -1.45820348645633 |
| C | 4.45420922597487 | -6.67646689538231 | -0.43043183387378 |
| C | 4.30011050570477 | -5.32923162032791 | -0.71468132859215 |
| H | 4.68574202290779 | -7.00637239600989 | 0.57146139657825 |
| H | 4.41911418466109 | -8.64825674686636 | -1.26020312620654 |
| H | 3.85408992468452 | -7.89505216915995 | -3.52636924568640 |
| H | 3.56926291317531 | -5.50875507850889 | -4.02693841757464 |
| C | 2.62552396635500 | -2.85055234474297 | -2.70866354636713 |
| H | 1.79160057064668 | -3.53605735695922 | -2.56364203714470 |

| | | | |
|---|---|---|---|
| H | 2.34698447790783 | -1.89908968356976 | -2.25229018250477 |
| C | 2.93826730149148 | -2.64647697420047 | -4.20058059169504 |
| H | 2.57681257858980 | -1.67028660849405 | -4.52245852406281 |
| H | 2.42291760474672 | -3.39078564517863 | -4.80839858874449 |
| C | 4.46387849066420 | -2.77786354626432 | -4.35513745207967 |
| H | 4.88313835165509 | -1.97027067095968 | -4.95623440852152 |
| H | 4.72042595753043 | -3.71322831218275 | -4.85379427132467 |
| C | 5.02745174591634 | -2.77417765235388 | -2.93457188242481 |
| H | 5.96009020635073 | -3.33415227916880 | -2.85446925400080 |
| H | 5.20844880261255 | -1.75626397749865 | -2.59375198803375 |
| H | 4.68931507988801 | -4.96105896575954 | 2.12991790321904 |
| H | 4.43562954046053 | -2.76863140049673 | 3.20874431996417 |
| H | 5.60365645831160 | -0.79593761402589 | 3.41566136271119 |
| H | 6.20088227011961 | 0.55223964173806 | 5.33354651962945 |
| H | 5.68703059994435 | 2.39556159665671 | 6.89118640581787 |
| H | 3.68607287223518 | 3.79163466895018 | 6.52224027762964 |
| H | 2.27504115207709 | 3.44957460350447 | 4.56050026341976 |
| C | 1.61694732177333 | 2.56540676981127 | 2.20080879161287 |
| C | 0.31768475281990 | 2.13935684209129 | 2.01072553572563 |
| C | -0.75920603527740 | 3.05535955488797 | 1.87816671261164 |
| C | -0.46144813752722 | 4.39992360499417 | 1.87266156085205 |
| C | 0.85471882867296 | 4.88143192329389 | 1.96276622206233 |
| C | 1.13209957194736 | 6.26561730614237 | 1.95384565053390 |
| C | 2.41699754102890 | 6.72154387968310 | 2.03931342027396 |
| C | 3.47794377066739 | 5.80834004323023 | 2.12823500945384 |
| C | 3.23629779414521 | 4.46235766771491 | 2.17117939370417 |
| C | 1.91871626316007 | 3.95792038782744 | 2.11325525657820 |
| H | 4.06140229126193 | 3.77254374917560 | 2.26070773564543 |
| H | 4.49214229331924 | 6.17764954856853 | 2.17418708613319 |
| H | 2.62493809210843 | 7.78108186705967 | 2.03396277100791 |
| H | 0.30426401841845 | 6.95425281521895 | 1.87355851284309 |
| H | -1.26372831769920 | 5.12130975749437 | 1.82641808898228 |
| C | -2.17953236846800 | 2.66312181587379 | 1.89823983817258 |
| C | -2.64857310772505 | 1.69743398052564 | 2.79045419342118 |
| C | -4.00696495865525 | 1.50375808228306 | 2.98218119445618 |
| C | -4.91027963942878 | 2.27503968023515 | 2.26820918990300 |
| C | -4.44863748088221 | 3.19903412043235 | 1.31799137004835 |
| C | -3.09921969362173 | 3.39515057060587 | 1.13674414611622 |
| H | -2.74229904558356 | 4.12003003554386 | 0.42104027936491 |
| C | -5.60401792882673 | 3.90412093519826 | 0.64714043459933 |
| C | -6.78571135291685 | 3.28599102051294 | 1.35675849531776 |
| C | -8.12890139430427 | 3.55294270528876 | 1.19260138113207 |
| C | -9.05021684412748 | 2.88385354548729 | 1.98778342138035 |
| C | -8.63057152074222 | 1.97493673949194 | 2.94662232776277 |
| C | -7.28334496896478 | 1.70233012709742 | 3.11928597820133 |
| C | -6.36234827933830 | 2.34749489254241 | 2.30980411591150 |
| H | -6.96099861404759 | 1.00941104423522 | 3.88107020004352 |
| H | -9.36101642243949 | 1.47741804149254 | 3.56807207355338 |
| H | -10.10510054589829 | 3.08318794372077 | 1.86557882593248 |
| H | -8.46830798289827 | 4.27410927703415 | 0.46368360818879 |

| | | | |
|---|---|---|---|
| C | -5.63149537840506 | 3.66524746287116 | -0.87881059622592 |
| C | -6.14835406627033 | 4.96475911967962 | -1.48827102129961 |
| H | -5.86207563174311 | 5.06357437575779 | -2.53578982984756 |
| H | -7.23611202724265 | 5.00971912041691 | -1.42448572509333 |
| C | -5.52159746419767 | 6.03779268431891 | -0.60249005984524 |
| H | -6.04608418143994 | 6.99081841658219 | -0.66538315162620 |
| H | -4.48647392125808 | 6.19836336137660 | -0.90238062373634 |
| C | -5.57422475794885 | 5.44706094746791 | 0.81026799969289 |
| H | -6.47898458239872 | 5.77323405999436 | 1.32494433894843 |
| H | -4.72024281158953 | 5.75951477239802 | 1.41123182815463 |
| H | -6.24304305556337 | 2.80157648093483 | -1.13881675066366 |
| H | -4.61646909987202 | 3.47365379840018 | -1.22699000158556 |
| H | -4.34763827467490 | 0.77421794588937 | 3.70069912532674 |
| H | -1.94938462298405 | 1.11006661447788 | 3.36572884980755 |
| O | 0.00543010489190 | 0.77647029979315 | 2.03729191212133 |
| O | 0.39640556340422 | 0.99101515400943 | -1.87986388554304 |
| C | 0.43558455155863 | 2.37466712123736 | -1.70965736960440 |
| C | -0.68742301792175 | 3.16719778596998 | -1.84243619807867 |
| C | -0.58895031848920 | 4.56513313416397 | -1.55960441014809 |
| C | -1.71194719653429 | 5.42111137218265 | -1.54146642628245 |
| C | -1.57677186157526 | 6.76063086650009 | -1.29583375596091 |
| C | -0.31144565757655 | 7.32173460718549 | -1.07194971337141 |
| C | 0.79411836501117 | 6.51952760114922 | -1.06339416243173 |
| C | 0.68028540489310 | 5.12975443125158 | -1.28083829784100 |
| C | 1.81238698656516 | 4.29861364285716 | -1.29024250969631 |
| C | 1.72154681618496 | 2.94226647830511 | -1.49570306557353 |
| C | 2.96971675464040 | 2.17952686462393 | -1.66223312063789 |
| C | 3.11680172815571 | 1.31035523855047 | -2.74448176996546 |
| C | 4.34785855492824 | 0.75361595315840 | -3.04538175688465 |
| C | 5.44124593970228 | 1.05221063466797 | -2.24803820625907 |
| C | 5.29607388156216 | 1.90453885040855 | -1.14278107270027 |
| C | 4.07394627278706 | 2.46643788446500 | -0.85241158577420 |
| H | 3.95911162776168 | 3.13498947436089 | -0.01238243453832 |
| C | 6.62307470584804 | 2.12082315084841 | -0.45389439659402 |
| C | 7.53243352862491 | 1.23360635819905 | -1.26995923120185 |
| C | 8.87981504041563 | 0.99361543118966 | -1.10154887056646 |
| C | 9.52920830139406 | 0.15583252604835 | -1.99899947767622 |
| C | 8.84089566551790 | -0.42199744192402 | -3.05484299496265 |
| C | 7.48687979577937 | -0.18613708609862 | -3.23181126303846 |
| C | 6.83246040979260 | 0.63672220576365 | -2.32903929587966 |
| H | 6.95770167277181 | -0.63353276050134 | -4.05998862946614 |
| H | 9.36678735103739 | -1.06243286746547 | -3.74828885531200 |
| H | 10.58415640261554 | -0.04332933318698 | -1.87662983710138 |
| H | 9.42750621487082 | 1.44646293232479 | -0.28758878954619 |
| C | 6.59288683722470 | 1.76426131836257 | 1.04950042340117 |
| H | 7.01019100012229 | 0.77451532598653 | 1.23196557964943 |
| H | 5.55806434500125 | 1.76007857117060 | 1.39193229282327 |
| C | 7.36740561997735 | 2.87233928689407 | 1.76043503867161 |
| H | 8.43937867631513 | 2.67372452937659 | 1.71980430521988 |
| H | 7.07427892641906 | 2.96841713982024 | 2.80619472380492 |

| | | | |
|---|---|---|---|
| C | 7.03747183235056 | 4.11149181393876 | 0.93456887373912 |
| H | 7.73769476032210 | 4.92938651751198 | 1.10221487445001 |
| H | 6.03391535131389 | 4.46215258323350 | 1.17600912189014 |
| C | 7.08173616753141 | 3.60397705531815 | -0.50639890500377 |
| H | 8.10275704283443 | 3.65144544057861 | -0.88812645192411 |
| H | 6.44785713991958 | 4.18943378097037 | -1.17220934394790 |
| H | 4.44599778107225 | 0.11804432403203 | -3.91140111270192 |
| H | 2.27006944971106 | 1.08760227412187 | -3.37623044575115 |
| H | 2.78373991484340 | 4.75524489061893 | -1.16939048313343 |
| H | 1.77656172548148 | 6.93239826067299 | -0.88870293824605 |
| H | -0.22081082186678 | 8.38500582168599 | -0.90711148872225 |
| H | -2.44738810350594 | 7.40036483013193 | -1.28514174335857 |
| H | -2.68972134365992 | 5.00837597559942 | -1.73644976877188 |
| C | -1.96314023752552 | 2.62460817799265 | -2.35775483508651 |
| C | -2.55084261197542 | 1.52425309034626 | -1.77918236976015 |
| C | -3.76189439749630 | 0.97261294244473 | -2.24621440885166 |
| C | -4.41496395067476 | 1.62051103675673 | -3.26678081388787 |
| C | -3.85116928520196 | 2.73358848413633 | -3.91413211234180 |
| C | -4.49872079399109 | 3.34394311924113 | -5.01002259615449 |
| C | -3.89385756569986 | 4.34756393514963 | -5.71211497780231 |
| C | -2.60115457240390 | 4.76308615592964 | -5.35996124304933 |
| C | -1.96070423160201 | 4.21144610917792 | -4.28449848664915 |
| C | -2.58161831989692 | 3.21444340727517 | -3.50130211360629 |
| H | -0.95812348451577 | 4.52956313862120 | -4.04264773664109 |
| H | -2.10723328128935 | 5.52052907308265 | -5.95080662335476 |
| H | -4.39157612424406 | 4.80698263045697 | -6.55340579834627 |
| H | -5.47674819047161 | 2.98328009838781 | -5.29473746163788 |
| H | -5.36154324422222 | 1.23707974583751 | -3.61930034744233 |
| C | -4.26587723107429 | -0.31286118230570 | -1.73989154522679 |
| C | -4.55863754194281 | -1.29050829253417 | -2.69251440125769 |
| C | -5.01114302121259 | -2.54000160872316 | -2.31713211068113 |
| C | -5.18537936627165 | -2.80175305164716 | -0.96778188994708 |
| C | -4.90808164827852 | -1.82187764382720 | -0.00259800559948 |
| C | -4.44241655579087 | -0.57952751818680 | -0.38157812144448 |
| H | -4.22102538045121 | 0.18593731628365 | 0.34854015570023 |
| C | -5.16489068881915 | -2.35975554026183 | 1.38507013467874 |
| C | -5.59488237194682 | -3.78101660494527 | 1.09560240011237 |
| C | -5.94097047128508 | -4.79853197955233 | 1.96212395160255 |
| C | -6.29648317831991 | -6.03797018771208 | 1.44367087154792 |
| C | -6.30701847065443 | -6.26399452576125 | 0.07624816266326 |
| C | -5.96127046064064 | -5.25192589376631 | -0.80426642431018 |
| C | -5.60801183992337 | -4.01499058221576 | -0.28939826752570 |
| H | -5.96460203643728 | -5.42237090276882 | -1.87072675630380 |
| H | -6.58532736075374 | -7.23604932470682 | -0.30427298500039 |
| H | -6.56610566583562 | -6.83741469552036 | 2.11845338088439 |
| H | -5.93415339099647 | -4.65571079402936 | 3.03048394139598 |
| C | -3.93467732393516 | -2.27140342938834 | 2.30386604689451 |
| H | -3.23120857667276 | -3.07935890979381 | 2.12153284870893 |
| H | -3.41155914111957 | -1.33353224196601 | 2.11268366015078 |
| C | -4.47401582411760 | -2.29339382342612 | 3.73845888473198 |

| | | | |
|---|---|---|---|
| H | -3.89197674818314 | -1.61898236207451 | 4.36610432163765 |
| H | -4.37897762974019 | -3.29147159689323 | 4.16703103312881 |
| C | -5.95439157823133 | -1.87832278359556 | 3.64812400551122 |
| H | -6.16840950775165 | -1.01627522466904 | 4.27925978609628 |
| C | -6.22209190176085 | -1.56435768216819 | 2.17194241034210 |
| H | -7.23582510678406 | -1.82862290105473 | 1.86972645492649 |
| H | -6.07442477327357 | -0.50301026194886 | 1.97254028262831 |
| H | -6.60374117828254 | -2.68683116426420 | 3.98453198397456 |
| H | -5.21182139802509 | -3.29419521023902 | -3.06301860237127 |
| H | -4.38393956414340 | -1.06714756033437 | -3.73433689136446 |
| O | -1.86600538997520 | 0.86774397868084 | -0.74284341480022 |

233

X-TBS ($r^2$SCAN-3c)

| | | | |
|---|---|---|---|
| P | -0.63280173788842 | 0.01501184465619 | -1.48919388920071 |
| N | -0.92160039504701 | -1.20804529721096 | -2.46115294723842 |
| S | -0.86680510172859 | -1.21015871468288 | -4.06547148898191 |
| O | 0.47791282788490 | -0.98249640207424 | -4.62432863266853 |
| O | -2.00554080992147 | -0.55050280602506 | -4.72760763538495 |
| C | -1.18165604034068 | -3.06115377743551 | -4.30751174643113 |
| F | -0.26638065172877 | -3.80186050994607 | -3.66320761745367 |
| F | -2.39985631812386 | -3.40705560208951 | -3.86846012017917 |
| F | -1.10510986054244 | -3.33248919164498 | -5.61846028162486 |
| N | -0.03614004824656 | -0.50493637561464 | -0.12500354587388 |
| P | 0.70963918593617 | -0.32169699140593 | 1.21572343621994 |
| N | 0.85059980762796 | -1.71491067070142 | 2.05457392402441 |
| S | -0.13348215283706 | -2.39949929161787 | 3.01747890085832 |
| O | -1.11861013957153 | -1.65670506759865 | 3.79811881215119 |
| O | -0.79168547099076 | -3.67477238487085 | 2.38887030881255 |
| Si | -0.75106164544980 | -4.47611516651214 | 0.80974083576236 |
| C | 0.94470975526459 | -4.28912496540544 | 0.08179408705382 |
| H | 1.74117523062100 | -4.47674478959362 | 0.80676730211026 |
| H | 1.08788104172934 | -3.28671504252699 | -0.33122731286155 |
| H | 1.06424173123403 | -5.00613499633358 | -0.73951514955128 |
| C | -2.07452944189914 | -3.68694121060955 | -0.21766569568762 |
| H | -3.07545807047793 | -3.95969527074442 | 0.12575149517877 |
| H | -1.99216635955371 | -2.59622652024650 | -0.21624441365757 |
| H | -1.97155732978834 | -4.01521604027395 | -1.25753510826347 |
| C | -1.18144895903944 | -6.24008262041928 | 1.32585969033684 |
| C | -0.05811371498001 | -6.84142830570963 | 2.18364172939639 |
| H | 0.06840915489761 | -6.29333796607658 | 3.12354075327367 |
| H | 0.90197170150261 | -6.84231950996952 | 1.65494805402484 |
| H | -0.30109004128145 | -7.88201600465320 | 2.43645082087311 |
| C | -2.50279016231320 | -6.26172306169542 | 2.11199945181014 |
| H | -2.43590762098996 | -5.68011312900879 | 3.03776936278330 |
| H | -3.33796270195299 | -5.86661979853086 | 1.52329918532944 |
| H | -2.75287981712465 | -7.29614727294878 | 2.38330435985098 |
| C | -1.34689549862700 | -7.06785357266808 | 0.03748843254679 |
| H | -2.15356152307072 | -6.67852937682740 | -0.59403850770923 |
| H | -0.42689766589246 | -7.09466756943991 | -0.55774315549001 |
| H | -1.60165377377760 | -8.10343113795204 | 0.29856689775942 |

| | | | |
|---|---|---|---|
| C | 1.02383462182058 | -3.26382699225522 | 4.24508631382596 |
| F | 0.28171154997489 | -3.89202891858409 | 5.15717712122195 |
| F | 1.78133106625025 | -2.34257811888108 | 4.83850043247589 |
| F | 1.79624912918536 | -4.14657999510694 | 3.61321042405825 |
| O | 2.21343692272285 | 0.16398918108040 | 0.93723210610744 |
| C | 3.01960230879445 | 0.34486030194741 | 2.07261361677462 |
| C | 2.79392003439715 | 1.46788863610293 | 2.84017708634241 |
| C | 3.53948665295421 | 1.61824270236779 | 4.05272317323692 |
| C | 3.28449498830826 | 2.64422881659296 | 4.99452568165931 |
| C | 4.03984779241954 | 2.75465854726053 | 6.13810635005517 |
| C | 5.08910367548124 | 1.84855324879239 | 6.39822936661684 |
| C | 5.33841920740580 | 0.82059805095771 | 5.52334662407950 |
| C | 4.56284938956388 | 0.66406454016727 | 4.34729108327985 |
| C | 4.77287958968848 | -0.42768346475464 | 3.48109635698101 |
| C | 4.00064976830909 | -0.63729568570754 | 2.35572053100909 |
| C | 4.17931460596060 | -1.84860999111466 | 1.53224621900369 |
| C | 4.37984796374656 | -3.07406278820082 | 2.19024128386367 |
| C | 4.49396086950690 | -4.26472856909288 | 1.48609604998836 |
| C | 4.39747445865015 | -4.23106137697438 | 0.09805548842013 |
| C | 4.23638203677049 | -3.00837070153374 | -0.57644514693682 |
| C | 4.13801837511176 | -1.82032587436772 | 0.12691267694241 |
| H | 4.02222194432671 | -0.87546106073730 | -0.39611700903086 |
| C | 4.17399928762183 | -3.22734602063422 | -2.07207401862134 |
| C | 4.18008924350338 | -4.74528949589707 | -2.16004022686733 |
| C | 4.02550356373349 | -5.57822857985383 | -3.26099086518424 |
| C | 4.06804572404549 | -6.96346702504965 | -3.08024329407338 |
| C | 4.26031635757760 | -7.51517242987668 | -1.81318822595252 |
| C | 4.40056608659650 | -6.68983299806776 | -0.70020096422267 |
| C | 4.35554057805913 | -5.30935862651093 | -0.88046955365448 |
| H | 4.53307967230817 | -7.11750703062283 | 0.28947146674407 |
| H | 4.29011648734786 | -8.59375306107458 | -1.69411124458846 |
| H | 3.94504376013261 | -7.61790853554182 | -3.93762180086883 |
| H | 3.85733444770533 | -5.17570995508968 | -4.25355786704482 |
| C | 2.98167783779942 | -2.55913084984137 | -2.79613778750836 |
| H | 2.06007008629699 | -3.13269003697817 | -2.67885539864389 |
| H | 2.81152258384520 | -1.57139370877587 | -2.35266137776900 |
| C | 3.41639389364004 | -2.40787187108363 | -4.27082188866649 |
| H | 3.09297542265238 | -1.44120628455464 | -4.66212119001961 |
| H | 2.94031615603092 | -3.16492446871578 | -4.90121348162268 |
| C | 4.95938386936204 | -2.55869490807433 | -4.28548002501113 |
| H | 5.45160513574200 | -1.74488894555136 | -4.82678131634012 |
| H | 5.25553345177741 | -3.48694084807371 | -4.78417930760880 |
| C | 5.38250101807762 | -2.59335755600247 | -2.81183135815388 |
| H | 6.31305174367692 | -3.14022577079588 | -2.63126836132853 |
| H | 5.50946407418593 | -1.57566915104166 | -2.42627539986740 |
| H | 4.61612208351306 | -5.20409245358498 | 2.01735164881032 |
| H | 4.39525851938329 | -3.09221045327233 | 3.27503222395917 |
| H | 5.56930296852407 | -1.12786250006077 | 3.71414095551346 |
| H | 6.12536191538545 | 0.09936606665329 | 5.72440047245960 |
| H | 5.68421626124484 | 1.95632327055989 | 7.29922227894029 |

| | | | |
|---|---|---|---|
| H | 3.82265598003505 | 3.54414603607865 | 6.85034978782687 |
| H | 2.47725893958914 | 3.34343505156780 | 4.81198144488700 |
| C | 1.77920561097220 | 2.46878065885199 | 2.43522927677554 |
| C | 0.46167633885308 | 2.10153170739141 | 2.21999806936441 |
| C | -0.58993814112203 | 3.03286100996054 | 2.01886710997319 |
| C | -0.23449408873672 | 4.36531070497673 | 1.92703208072550 |
| C | 1.10078183887635 | 4.79994265012240 | 2.03419203776934 |
| C | 1.43259976878655 | 6.17287604141873 | 1.92740430985407 |
| C | 2.73714701620212 | 6.59116606183045 | 2.01415020655908 |
| C | 3.76465501163984 | 5.64605695688961 | 2.21297282297765 |
| C | 3.47018993533435 | 4.31043895288269 | 2.35499083544298 |
| C | 2.13311277246770 | 3.84740208882078 | 2.28796778655408 |
| H | 4.27331057155256 | 3.60180623089206 | 2.51868457265377 |
| H | 4.79811954514260 | 5.97508868529491 | 2.25934362000307 |
| H | 2.98160709272888 | 7.64472592515323 | 1.92414335610810 |
| H | 0.63102982404407 | 6.88486503348414 | 1.75791489088833 |
| H | -1.01464199640715 | 5.11062444079336 | 1.80252795422630 |
| C | -2.02810213378369 | 2.68430085031462 | 2.04548291083382 |
| C | -2.55238561345398 | 1.88139792759715 | 3.07237688517594 |
| C | -3.92549256661048 | 1.79023886016009 | 3.27858339585804 |
| C | -4.78759775791329 | 2.49417109084483 | 2.43984139658128 |
| C | -4.27444455680593 | 3.23862137543004 | 1.36458332687807 |
| C | -2.90899183524025 | 3.33612699578425 | 1.16700427877289 |
| H | -2.50420464517242 | 3.93204533169133 | 0.35345130752329 |
| C | -5.39082095111423 | 3.88275327486178 | 0.57130376343558 |
| C | -6.61223870055724 | 3.48178737034062 | 1.37894562536080 |
| C | -7.94771800680665 | 3.81514356975335 | 1.19510750428756 |
| C | -8.90289400110330 | 3.32428052424903 | 2.08897924891443 |
| C | -8.52734353289328 | 2.51696589451345 | 3.16296755410546 |
| C | -7.18994805387810 | 2.18340679026574 | 3.35987295753563 |
| C | -6.23789901094265 | 2.66056942603707 | 2.46203284040265 |
| H | -6.89652788962126 | 1.57108963305349 | 4.20637444281146 |
| H | -9.28181685900189 | 2.15268789178442 | 3.85309805954164 |
| H | -9.94838109612733 | 3.58113725118496 | 1.94970869180172 |
| H | -8.26127184962737 | 4.45448535429190 | 0.37702609655400 |
| C | -5.45052712456421 | 3.38514434657213 | -0.89316934599672 |
| C | -6.26652619146973 | 4.43933539606985 | -1.65432616293641 |
| H | -5.93333748668301 | 4.51471019657598 | -2.69424471158029 |
| H | -7.32431970497683 | 4.16050926569515 | -1.68013285591858 |
| C | -6.06973957850148 | 5.76660928467626 | -0.87346983671242 |
| H | -7.03500865265793 | 6.19886538082294 | -0.59325182153518 |
| H | -5.55430786813503 | 6.51922765218264 | -1.47818298114452 |
| C | -5.23926981699703 | 5.41161831020285 | 0.37870074555712 |
| H | -5.54236548815220 | 5.96592796440643 | 1.27172403518763 |
| H | -4.18004030574080 | 5.62369989715213 | 0.19943707895049 |
| H | -5.85846497070552 | 2.37347014365819 | -0.96793232844584 |
| H | -4.42532230774224 | 3.35313259123815 | -1.27468611718890 |
| H | -4.30986225477548 | 1.20466692746574 | 4.10834380465586 |
| H | -1.87732130818946 | 1.37351042096814 | 3.75273681657637 |
| O | 0.11726123635506 | 0.74544804226304 | 2.27848401230145 |

| | | | |
|---|---|---|---|
| O | 0.26671534690411 | 1.08684323400200 | -2.30859201303609 |
| C | 0.27556805044629 | 2.45308852710730 | -2.04240606246974 |
| C | -0.89164902627236 | 3.18468144584087 | -2.18209067920626 |
| C | -0.86380164119273 | 4.57441058863363 | -1.83810745838458 |
| C | -2.02593869256679 | 5.38451642286962 | -1.81472646753708 |
| C | -1.95395939830456 | 6.71832762614602 | -1.48774841773210 |
| C | -0.71426173181073 | 7.31791694756860 | -1.18446073219955 |
| C | 0.42837852979618 | 6.55682885150531 | -1.18162084909856 |
| C | 0.38200638356193 | 5.17291838938899 | -1.47904190179222 |
| C | 1.55138477883058 | 4.38723510666072 | -1.45965812473930 |
| C | 1.53557135483910 | 3.03493322532453 | -1.73878822805292 |
| C | 2.81852637829634 | 2.30504021142861 | -1.84354525604295 |
| C | 3.09384288601736 | 1.49532651956801 | -2.95901994100186 |
| C | 4.37454523267656 | 1.00533722174952 | -3.18884757456864 |
| C | 5.39239233423101 | 1.30997190936383 | -2.28783679451197 |
| C | 5.11524358387127 | 2.07271629704941 | -1.14191526572427 |
| C | 3.84290181017453 | 2.56793828810716 | -0.91866836719166 |
| H | 3.62857153568696 | 3.17526645703464 | -0.04315677452334 |
| C | 6.36208196246782 | 2.25951159956752 | -0.30348344191007 |
| C | 7.41208929929966 | 1.56560035405354 | -1.15432741200797 |
| C | 8.77766491391962 | 1.42626834503046 | -0.94521904506780 |
| C | 9.54218732879367 | 0.72029997615220 | -1.87819972587320 |
| C | 8.94952959968399 | 0.16048334549092 | -3.01033789941339 |
| C | 7.58142481764843 | 0.29758392498504 | -3.23080278628907 |
| C | 6.81794271431113 | 0.99893939650347 | -2.30044096481621 |
| H | 7.12106193819596 | -0.13194520818205 | -4.11538862824838 |
| H | 9.55980824355149 | -0.38238599496384 | -3.72526392139507 |
| H | 10.61047975225415 | 0.60919546548609 | -1.72055358836385 |
| H | 9.26210642015592 | 1.85643242193120 | -0.07530807195183 |
| C | 6.24061110941478 | 1.63289990828863 | 1.10731994923742 |
| H | 6.33143462400988 | 0.54387065267164 | 1.07900829617345 |
| H | 5.24263080486680 | 1.86852015267926 | 1.48806398584302 |
| C | 7.31418155512243 | 2.32169021773471 | 1.96499034524042 |
| H | 8.22848048231302 | 1.72121806663412 | 1.99625465326882 |
| H | 6.97415890601842 | 2.43019355588143 | 3.00001775787556 |
| C | 7.58176341704188 | 3.69363942318649 | 1.28831585230491 |
| H | 8.63652541473762 | 3.79080776887412 | 1.01442950665670 |
| H | 7.35382711772099 | 4.52931941289294 | 1.95694810969673 |
| C | 6.68405210184323 | 3.73461170624698 | 0.03524126189539 |
| H | 7.13962046573586 | 4.25900361769273 | -0.80975737076651 |
| H | 5.73911544832600 | 4.23796042293605 | 0.26911367869147 |
| H | 4.57404566202472 | 0.41747759036093 | -4.07952572805553 |
| H | 2.31142073283203 | 1.28860390721878 | -3.68056423927131 |
| H | 2.50071329236207 | 4.87362701953929 | -1.25479144028789 |
| H | 1.38939147285052 | 6.99881344069693 | -0.93739055575394 |
| H | -0.66845171988555 | 8.37651534832950 | -0.94946082133272 |
| H | -2.86048635023536 | 7.31541974227546 | -1.46983649041633 |
| H | -2.98576714862840 | 4.94526387075410 | -2.06087422237978 |
| C | -2.12775292580872 | 2.56066267251710 | -2.70924253814352 |
| C | -2.65713744064367 | 1.43323558662803 | -2.11391272568140 |

| | | | |
|---|---|---|---|
| C | -3.86227239264689 | 0.81207992709508 | -2.51605986513012 |
| C | -4.53361047968747 | 1.38429351180990 | -3.57544450792674 |
| C | -4.02289621404631 | 2.49555259139367 | -4.27526902788069 |
| C | -4.70570822794071 | 3.02520555123156 | -5.39844078533888 |
| C | -4.17310706001229 | 4.06419749583083 | -6.12035711105247 |
| C | -2.92267152530852 | 4.60455113592475 | -5.75499718864452 |
| C | -2.24585642023523 | 4.12806847765454 | -4.65713427860550 |
| C | -2.78538620737471 | 3.08386518572735 | -3.86743695448022 |
| H | -1.28076482369731 | 4.54645889005845 | -4.39729899792009 |
| H | -2.48553941921341 | 5.39892119037649 | -6.35148954144021 |
| H | -4.69955982573951 | 4.45653330457660 | -6.98440794488002 |
| H | -5.65210188355288 | 2.57675319110793 | -5.68657596219957 |
| H | -5.47889166584773 | 0.95591180672532 | -3.89549326072276 |
| C | -4.38319606835401 | -0.41364316436917 | -1.87813217655307 |
| C | -4.79204617491932 | -1.46656843693458 | -2.71260659147373 |
| C | -5.30490573697822 | -2.64460861615447 | -2.18758970956768 |
| C | -5.41491836229419 | -2.76828209146342 | -0.80605800933852 |
| C | -5.02178654236036 | -1.71826029320029 | 0.04168472515546 |
| C | -4.50310461641073 | -0.54577391275618 | -0.48404405205021 |
| H | -4.19698256529912 | 0.26986366953873 | 0.16634887287489 |
| C | -5.22793639972299 | -2.09797878474470 | 1.49431440598118 |
| C | -5.73094350923162 | -3.52865666509723 | 1.37258217916072 |
| C | -6.03844942978019 | -4.45773326246336 | 2.35923220624433 |
| C | -6.46522953131919 | -5.73545118353594 | 1.98747630671449 |
| C | -6.58050734443488 | -6.08770723795677 | 0.64259857588849 |
| C | -6.26767386894840 | -5.16766217566280 | -0.35512081021165 |
| C | -5.84590723980210 | -3.89257724837342 | 0.01569758297662 |
| H | -6.34621114758894 | -5.44299463646416 | -1.40273826837858 |
| H | -6.90995193454698 | -7.08638480651569 | 0.37324739294580 |
| H | -6.70290107101329 | -6.46420144429095 | 2.75630135642329 |
| H | -5.94098437975263 | -4.21701113635073 | 3.41170297386240 |
| C | -3.96510460666892 | -1.97598098738157 | 2.37948080546526 |
| H | -3.28336149907010 | -2.81454185461024 | 2.23228158630810 |
| H | -3.43279239792195 | -1.05973972904166 | 2.10003494379046 |
| C | -4.47766744722433 | -1.87421353893782 | 3.82997622870349 |
| H | -3.87327639863847 | -1.16401022751564 | 4.40023023867366 |
| H | -4.38925009783136 | -2.83654369914929 | 4.34357493537910 |
| C | -5.96149080624074 | -1.43292424214875 | 3.73078831105946 |
| H | -6.16908958159010 | -0.54748581410048 | 4.33743738896220 |
| C | -6.21531893016885 | -1.16503770597826 | 2.23976240839995 |
| H | -7.25312025027519 | -1.32727379274607 | 1.93343483882299 |
| H | -5.94840040866932 | -0.13181325849572 | 1.99093786018067 |
| H | -6.62722457193813 | -2.22116704800721 | 4.09513669030306 |
| H | -5.58898124864974 | -3.45830481978465 | -2.84823317107888 |
| H | -4.65776145995638 | -1.36400384414612 | -3.78417138650158 |
| O | -1.94365287108165 | 0.86276908332859 | -1.05359971046123 |

283

I-1 ($r^2$SCAN-3c/xTB)

| | | | |
|---|---|---|---|
| P | -1.75459043117740 | 0.56544727074455 | -1.32381271061364 |
| N | -1.26564923026848 | 1.65597657223881 | -0.26861530797369 |

| | | | |
|---|---|---|---|
| P | -0.22639496028113 | 1.32642576857430 | 0.88844068774492 |
| N | 0.20578623935117 | -0.20810206607504 | 1.15265511918861 |
| S | -1.02435711550075 | -1.11916283204366 | 1.43343325076939 |
| O | -0.73224169913832 | -2.51261399382924 | 1.21924807106169 |
| O | -2.32080258948168 | -0.62202613803716 | 1.09360206701759 |
| C | -1.07304958641021 | -1.10049042803976 | 3.37691987423823 |
| F | -2.28041915533665 | -0.83185367165834 | 3.87780477255687 |
| F | -0.70806188082024 | -2.29119386977513 | 3.89142966181094 |
| F | -0.22128687655212 | -0.20687778733386 | 3.90757051806259 |
| O | 1.09766367362064 | 2.16824220196204 | 0.67814278261407 |
| C | 1.73935526654523 | 2.91324456010214 | 1.66639813571228 |
| C | 1.03312023100561 | 3.88538742130150 | 2.34437226957237 |
| C | 1.64516799752154 | 4.61974085500055 | 3.39978818951929 |
| C | 0.92558333953355 | 5.48312750956200 | 4.25401129651113 |
| C | 1.56562179535782 | 6.18474099225934 | 5.23899272629610 |
| C | 2.95383546967160 | 6.06951196099291 | 5.41257553016985 |
| C | 3.67311846225142 | 5.22246076583077 | 4.61775602393611 |
| C | 3.03556845110880 | 4.46089929034204 | 3.61375018010791 |
| C | 3.74289578923967 | 3.54458668440098 | 2.81900174933758 |
| C | 3.13423940410264 | 2.72633353628626 | 1.88885364894366 |
| C | 3.99289776998010 | 1.81210934701433 | 1.11696187830797 |
| C | 5.18525900992299 | 1.34413342634757 | 1.69460903773349 |
| C | 6.11907778686772 | 0.70888030546273 | 0.91090515837041 |
| C | 5.87431104721298 | 0.48546881607299 | -0.45276289105132 |
| C | 4.65264385603178 | 0.83380942700512 | -0.99945332119581 |
| C | 3.72752071556076 | 1.50217682350187 | -0.21746602816765 |
| H | 2.79751531938712 | 1.79690052137197 | -0.66643975743063 |
| H | 4.41586805001261 | 0.62140740116162 | -2.02843363385501 |
| C | 7.05863469030233 | -0.11813722724966 | -1.03886029185547 |
| C | 7.34395802652639 | -0.47877828350000 | -2.34689087910428 |
| C | 8.57364168449313 | -1.05510658148043 | -2.62374489514676 |
| C | 9.50308690851800 | -1.26159655201616 | -1.61520334053031 |
| C | 9.23231242725674 | -0.87469286928763 | -0.30887993570115 |
| C | 8.01186881535607 | -0.29792785489991 | -0.02427342089663 |
| C | 7.50334642723807 | 0.23912079900007 | 1.29233181575272 |
| C | 7.50183109592819 | -0.80250624317520 | 2.43677328913507 |
| C | 8.62206945684158 | -0.37855007267781 | 3.38535699077441 |
| C | 8.57557399106438 | 1.14303660479390 | 3.30565072960935 |
| C | 8.38788022256121 | 1.40399789851870 | 1.81269948485047 |
| H | 7.92461118288167 | 2.36892623581550 | 1.60939154376411 |
| H | 9.35376573953905 | 1.37958929488381 | 1.30575935693401 |
| H | 9.47463486699235 | 1.61777197509940 | 3.69682528969980 |
| H | 7.71351793795390 | 1.51365136103528 | 3.86280901436495 |
| H | 8.46798562354250 | -0.75399504127486 | 4.39591725462388 |
| H | 9.58573988675794 | -0.74135061497780 | 3.02452909472976 |
| H | 7.63194373166043 | -1.81517705388904 | 2.05517921156284 |
| H | 6.54568386434577 | -0.74837694511374 | 2.96028659982700 |
| H | 9.96990595423870 | -1.02351786185696 | 0.46593910535860 |
| H | 10.45380814497067 | -1.71675866106048 | -1.85075211569177 |
| H | 8.81299160476358 | -1.34331900873435 | -3.63721904697296 |

| | | | |
|---|---|---|---|
| H | 6.62590994267895 | -0.30291897199393 | -3.13379132768955 |
| H | 5.37245589083429 | 1.48683889417049 | 2.74969251248360 |
| H | 4.81165044680472 | 3.48906576117865 | 2.95751810140182 |
| H | 4.73917067596315 | 5.10916183542015 | 4.75071626120430 |
| H | 3.44249873226194 | 6.64513067238053 | 6.18461010737468 |
| H | 1.00145945162135 | 6.83411274809806 | 5.89191382632042 |
| H | -0.14339264974468 | 5.57365270312129 | 4.13336123053744 |
| C | -0.36388271444528 | 4.19891780816385 | 1.97935826701961 |
| C | -1.31652322346587 | 3.20972965733377 | 2.02467696803589 |
| C | -2.68125256924542 | 3.46860746523587 | 1.76168147012434 |
| C | -3.03498734583600 | 4.74602922335450 | 1.39185812954979 |
| C | -2.08869785605279 | 5.77901868167192 | 1.26896608037545 |
| C | -2.46654407020346 | 7.07617114099860 | 0.85908398347603 |
| C | -1.53509291571430 | 8.06628651110557 | 0.72042343694410 |
| C | -0.18276997096400 | 7.79722286939228 | 0.98116619795839 |
| C | 0.21149068008773 | 6.55720403331334 | 1.40341936181869 |
| C | -0.72866340216536 | 5.51728422775984 | 1.57816695039410 |
| H | 1.25616563658539 | 6.36155094128623 | 1.59012446218684 |
| H | 0.55029502108909 | 8.57856723054627 | 0.84553045779838 |
| H | -1.82928195658650 | 9.05616654291685 | 0.40386223143457 |
| H | -3.51046948054538 | 7.26317707693121 | 0.65379907966421 |
| H | -4.07365926801697 | 4.97212818958810 | 1.19664619208549 |
| C | -3.74090808285891 | 2.47791871519915 | 1.99309881475332 |
| C | -4.81063243169590 | 2.36333153642958 | 1.10158620643156 |
| C | -5.90678541460661 | 1.61142723603784 | 1.46125304326738 |
| C | -5.94766726688225 | 0.96720770057349 | 2.70663753882700 |
| C | -4.86237689839096 | 1.02347303369707 | 3.56530035232621 |
| C | -3.76509692604297 | 1.78084509292197 | 3.20008123391493 |
| H | -2.92616790511350 | 1.87011126011215 | 3.87485023680638 |
| H | -4.87456496013428 | 0.50547432391711 | 4.51269529684693 |
| C | -7.25118023646812 | 0.34637050077910 | 2.86201636638510 |
| C | -7.79093562076082 | -0.35603305065430 | 3.92712263562252 |
| C | -9.08932536363421 | -0.82869125281370 | 3.82298148639415 |
| C | -9.83067734330512 | -0.60151956202997 | 2.67418080147012 |
| C | -9.29459179666642 | 0.10887561992706 | 1.60700270697929 |
| C | -8.00420093104521 | 0.58772990882941 | 1.70117302189727 |
| C | -7.19615880404642 | 1.38830114878125 | 0.70507936826269 |
| C | -7.88492107351755 | 2.69361857254834 | 0.26547886689693 |
| C | -8.74061138943802 | 2.33745492055108 | -0.95715436626111 |
| C | -8.17255709357423 | 1.02128117973482 | -1.51935437219477 |
| C | -6.98247145847619 | 0.65080117817640 | -0.62857618758375 |
| H | -6.90031448009467 | -0.42698863071945 | -0.48778456076487 |
| H | -6.05027169617276 | 1.00220741984946 | -1.06788576093329 |
| H | -7.85877733546464 | 1.12399427474812 | -2.55931805061453 |
| H | -8.92672865422110 | 0.23452303211009 | -1.48754922307937 |
| H | -9.78409819958855 | 2.21187455412437 | -0.66807170292569 |
| H | -8.70434465341451 | 3.13445715648970 | -1.69951655506569 |
| H | -8.47892747169706 | 3.12022219132891 | 1.07404453503226 |
| H | -7.11725805174066 | 3.41738743965151 | -0.01109239079583 |
| H | -9.89333554410380 | 0.27808448680241 | 0.72564345723189 |

| | | | |
|---|---|---|---|
| H | -10.84134221973746 | -0.97823836618025 | 2.60851952492410 |
| H | -9.52909889322017 | -1.37825457215776 | 4.64316683303096 |
| H | -7.20945011406081 | -0.53074085532130 | 4.82064748578060 |
| H | -4.76218881386509 | 2.87231258954695 | 0.14848862352154 |
| O | -0.87526458721550 | 1.89199007835676 | 2.26123210800046 |
| N | -1.09604439495522 | -0.90512084767741 | -1.42458579817966 |
| S | 0.41807735592393 | -0.90428194467812 | -1.76143885925624 |
| O | 1.13622373693128 | -2.07825041746789 | -1.33308145281038 |
| O | 1.10481744752529 | 0.34971205914959 | -1.65142799070771 |
| C | 0.34253556344227 | -1.29271326894535 | -3.64517662234339 |
| F | -0.04788173596963 | -0.28230942553179 | -4.43038698464427 |
| F | 1.51368106980314 | -1.72683532182342 | -4.14258150222804 |
| F | -0.53226301362630 | -2.29162835646693 | -3.83481278246345 |
| O | -1.55404525130779 | 1.28807303411183 | -2.77207762588221 |
| C | -2.14971874538032 | 2.56198578206639 | -2.72487576706808 |
| C | -3.52555384824701 | 2.63627639171286 | -2.81216876687451 |
| C | -4.18940195764670 | 3.86791385523381 | -2.56800016439781 |
| C | -5.59542821173974 | 3.97408006397527 | -2.48818430212544 |
| C | -6.19192191783465 | 5.17648918268437 | -2.22399735893926 |
| C | -5.41687084718978 | 6.33251038507800 | -2.03940134728580 |
| C | -4.05350166198726 | 6.25848168599400 | -2.09395206281027 |
| C | -3.40556350137800 | 5.02709872474297 | -2.33449136227300 |
| C | -2.00429912659916 | 4.92010207296527 | -2.34324762704130 |
| C | -1.35883734197061 | 3.71392647796611 | -2.50684215927299 |
| C | 0.11124443463556 | 3.68274556977449 | -2.45584264384815 |
| C | 0.86814090240747 | 3.02898760620286 | -3.42989682324762 |
| C | 2.24261936020948 | 3.12335744707847 | -3.38620724098955 |
| C | 2.87655696961974 | 3.82278936142965 | -2.34781745727214 |
| C | 2.13085171943880 | 4.46438611948190 | -1.37287637957888 |
| C | 0.75279984626006 | 4.40338632849843 | -1.44858001094397 |
| H | 0.15250868639720 | 4.89664091197565 | -0.70116221151484 |
| H | 2.61323379213650 | 4.99855095480660 | -0.56614841519949 |
| C | 4.31464033490429 | 3.68497681465086 | -2.50302239495713 |
| C | 5.36010399278189 | 4.09318180837575 | -1.69089542853048 |
| C | 6.65357597377694 | 3.75066348406937 | -2.05137223831233 |
| C | 6.89954332070304 | 3.02666637292116 | -3.20774801067842 |
| C | 5.85376776251546 | 2.62360769021060 | -4.02837571317286 |
| C | 4.56191475615236 | 2.94538405653659 | -3.66805525630541 |
| C | 3.26944700491199 | 2.57015881977792 | -4.35023613408789 |
| C | 3.13259533365469 | 3.20827568818799 | -5.75253548190643 |
| C | 2.30677655253680 | 2.20976897286332 | -6.55752128890200 |
| H | 2.41802171966645 | 2.35211621113176 | -7.63267819657535 |
| H | 1.25011066137361 | 2.30264133084482 | -6.30633343468194 |
| C | 2.84074905041458 | 0.85918692688985 | -6.08812550710174 |
| C | 3.12085862446329 | 1.04962717843390 | -4.59224428589882 |
| H | 4.02579098935864 | 0.51641420329086 | -4.29888988930110 |
| H | 2.30575393256716 | 0.67480606908961 | -3.97496656239484 |
| H | 2.13835930844663 | 0.04706041688111 | -6.27093106221235 |
| H | 3.76917303592977 | 0.63056024440247 | -6.61593494854741 |
| H | 2.67185081270528 | 4.19463631600839 | -5.70073106034390 |

| | | | |
|---|---|---|---|
| H | 4.12349944498426 | 3.31441664421692 | -6.19915086972033 |
| H | 6.05498760804480 | 2.05446272539550 | -4.92583723808641 |
| H | 7.91483542746780 | 2.76442230039336 | -3.46799898112529 |
| H | 7.48167395724431 | 4.04578168032192 | -1.42313155746998 |
| H | 5.16901634356800 | 4.64979942927886 | -0.78481052163193 |
| H | 0.36473877834889 | 2.46731197051054 | -4.20256049567252 |
| H | -1.41938864130720 | 5.81708196449691 | -2.19618173931456 |
| H | -3.44341968602705 | 7.13772347606755 | -1.94659873082443 |
| H | -5.90803283142350 | 7.27568969658461 | -1.84970816353672 |
| H | -7.26803675278622 | 5.24582862014647 | -2.15826837092852 |
| H | -6.19628233768357 | 3.08912145906717 | -2.62990674979875 |
| C | -4.25259223127849 | 1.40285941756041 | -3.17504773580014 |
| C | -4.05195535452174 | 0.26847124045531 | -2.41577988685534 |
| C | -4.57552289787045 | -0.98293303569708 | -2.80765867148946 |
| C | -5.40403895096360 | -1.02437245015885 | -3.90605715946906 |
| C | -5.68541550226923 | 0.11678746644054 | -4.67798515161966 |
| C | -6.53306171797626 | 0.05465545130084 | -5.80572395712613 |
| C | -6.72438301389965 | 1.15081876533938 | -6.59835952069014 |
| C | -6.05412941244774 | 2.34950572767096 | -6.30889679687564 |
| C | -5.23799045464174 | 2.44544182676156 | -5.21537106462909 |
| C | -5.05751468090087 | 1.34614884532387 | -4.34704786515803 |
| H | -4.71905127967171 | 3.36947243306116 | -5.01238466769020 |
| H | -6.18404114041642 | 3.20079647535213 | -6.96068382991468 |
| H | -7.37455118762729 | 1.09819218046987 | -7.45897344985501 |
| H | -7.01994623229529 | -0.88307106784737 | -6.03183747236982 |
| H | -5.83726103217055 | -1.96968972932314 | -4.20128945678882 |
| C | -4.23253160973406 | -2.25599056785997 | -2.14766083021942 |
| C | -4.49503484589334 | -2.50507539604401 | -0.80115737556413 |
| C | -4.32227514010174 | -3.78452508000702 | -0.31365355354975 |
| C | -3.84010991448316 | -4.80227770120820 | -1.15191886924305 |
| C | -3.50669583680940 | -4.53995261009102 | -2.47061944890391 |
| C | -3.72425908589633 | -3.26841228437135 | -2.96312635268269 |
| H | -3.49325068470616 | -3.04526894816800 | -3.99502862052022 |
| H | -3.11044039341042 | -5.31462450086733 | -3.11107980729250 |
| C | -3.81904763897997 | -6.05238679767942 | -0.41199071704234 |
| C | -3.44877580691038 | -7.33157669679070 | -0.79732599639437 |
| C | -3.57457856853913 | -8.36387575949684 | 0.11866868587725 |
| C | -4.05691849830685 | -8.12138307126428 | 1.39600170658529 |
| C | -4.42153166941283 | -6.83947223044156 | 1.78636208885012 |
| C | -4.30183256030111 | -5.80539120770204 | 0.88067700789154 |
| C | -4.65303283996101 | -4.34898551863863 | 1.04947115257529 |
| C | -3.89553297801253 | -3.66373134612760 | 2.19892506362519 |
| C | -4.75745098947597 | -2.46159100799517 | 2.59840588022759 |
| C | -6.19335809353050 | -2.78613283530207 | 2.14744097217587 |
| C | -6.12974130070631 | -4.14176461762965 | 1.43647687268205 |
| H | -6.43058601260579 | -4.93942499986196 | 2.11772421021545 |
| H | -6.77999246716395 | -4.18316449909105 | 0.56227011506280 |
| H | -6.56592911753455 | -2.01595547999775 | 1.47363388111699 |
| H | -6.87879360347393 | -2.82772609175718 | 2.99391127842161 |
| H | -4.38381439794852 | -1.56441261457109 | 2.10682605458429 |

| | | | |
|---|---|---|---|
| H | -4.70401729058780 | -2.28520850985068 | 3.67274214963487 |
| H | -3.80356934090596 | -4.36456699620528 | 3.03220236727059 |
| H | -2.89489227831124 | -3.35948120129505 | 1.89276975895632 |
| H | -4.79546446913967 | -6.66436206906544 | 2.78530757523790 |
| H | -4.15200313054049 | -8.93932383656607 | 2.09491619530853 |
| H | -3.29990261433360 | -9.36941851014029 | -0.16585940873088 |
| H | -3.07868313562573 | -7.52297333582123 | -1.79436219701523 |
| H | -4.84659216826254 | -1.70205166718186 | -0.17519599370357 |
| O | -3.32542723122314 | 0.38190096072758 | -1.22960686817325 |
| H | 2.56892717389304 | -2.17752913733688 | 5.20012799593246 |
| C | 3.62527171943900 | -2.05105547666119 | 4.93091849720433 |
| C | 4.08579040763416 | -0.66473466879898 | 5.30844990936315 |
| C | 3.19721049564215 | 0.41555878582443 | 5.31751903675710 |
| C | 3.63790510906557 | 1.69563721723301 | 5.64351274459853 |
| C | 4.97428874415814 | 1.91546896944169 | 5.96788012843109 |
| C | 5.86843134149848 | 0.84773022539870 | 5.96460700537860 |
| C | 5.42493048630873 | -0.43000740342967 | 5.63486095564613 |
| H | 6.11845410580461 | -1.26672105300093 | 5.64688162219243 |
| H | 6.90724222943113 | 1.00517470283286 | 6.23698330878063 |
| H | 5.30934678854298 | 2.91113617438209 | 6.23817094575754 |
| H | 2.92748832420017 | 2.51650247667900 | 5.65036534427028 |
| H | 2.15124995999691 | 0.25328185612653 | 5.06366583591794 |
| C | 3.77939912779109 | -2.34617765931587 | 3.42717576718714 |
| C | 2.90455106590012 | -1.43863020543120 | 2.56665251984457 |
| C | 2.99130665729971 | -1.72614906135232 | 1.05655671653497 |
| C | 2.33644510634848 | -3.00908631933874 | 0.71337534959241 |
| N | 3.01122536767682 | -4.04273668534826 | 0.34163634109683 |
| O | 4.34431414872500 | -4.01597472665611 | 0.27736618010535 |
| Si | 5.21879227964545 | -4.64863644348984 | -1.11045987808630 |
| C | 5.19610679980812 | -6.52031303327509 | -1.06410514659588 |
| H | 5.99229727310197 | -6.92933387539419 | -1.69742748325128 |
| H | 4.23866021042212 | -6.92567873733681 | -1.40272676410241 |
| H | 5.36846172797001 | -6.87047878270130 | -0.04010768737381 |
| C | 6.90051135498392 | -4.00274576913804 | -0.60705778920227 |
| H | 7.13385447981430 | -4.33993514813906 | 0.40899966344732 |
| H | 7.68825171595720 | -4.37145421838374 | -1.27204088582879 |
| H | 6.93208151065327 | -2.90732232991538 | -0.61891291332291 |
| C | 4.56763654078288 | -3.89926044545984 | -2.70879717954039 |
| C | 5.66797863862549 | -4.05679721670403 | -3.77909789183437 |
| H | 6.57885803038185 | -3.51777672194733 | -3.49856551253381 |
| H | 5.91978842981440 | -5.10857478914841 | -3.95622496105281 |
| H | 5.30600052305823 | -3.63633481098329 | -4.72590849198395 |
| C | 4.28733583233490 | -2.40728654268837 | -2.47858115722598 |
| H | 3.40623482353405 | -2.24761382718774 | -1.84453591982524 |
| H | 5.13893655198691 | -1.88847701526643 | -2.02819759157054 |
| H | 4.05371423071468 | -1.92326174313367 | -3.43589224270069 |
| C | 3.27967278028037 | -4.57747818877630 | -3.19643269696314 |
| H | 2.45199897367694 | -4.40598983989631 | -2.49871500114707 |
| H | 3.40666424880162 | -5.65460960054757 | -3.35053479264959 |
| H | 2.96877120382954 | -4.12670916796952 | -4.14798220919695 |

| | | | |
|---|---|---|---|
| O | 2.55010623191834 | -5.26655406933808 | 0.01994226142163 |
| Si | 0.93969363481847 | -5.96261173149616 | 0.11344674556412 |
| C | 1.33957262835342 | -7.50970602096050 | -0.87840646158237 |
| H | 0.47728206524705 | -8.18725238449712 | -0.87170563380368 |
| H | 1.54458070516074 | -7.24199154739682 | -1.92182971806243 |
| H | 2.20060895776177 | -8.05029640940644 | -0.47371274930271 |
| C | -0.32244089524654 | -4.92347476198598 | -0.72918048212826 |
| H | 0.10117926847392 | -4.42472872118593 | -1.60380205130044 |
| H | -1.16578274146765 | -5.54207820414785 | -1.04478295748676 |
| H | -0.69580579753880 | -4.12766123614982 | -0.08089587288692 |
| C | 0.57867721223095 | -6.37644002592018 | 1.92692923107973 |
| C | 0.70084752132286 | -5.11624332743850 | 2.79993981349022 |
| H | 0.35047154431454 | -5.32753823112480 | 3.81853121579201 |
| H | 1.73953081166505 | -4.77651572371876 | 2.87586822031635 |
| H | 0.09868765856100 | -4.28093274736051 | 2.42012877068467 |
| C | 1.52501720110855 | -7.46649068371558 | 2.46171312968565 |
| H | 1.31355606410346 | -7.64148233662952 | 3.52365072809451 |
| H | 1.38919367082973 | -8.41069079984864 | 1.92740247150746 |
| H | 2.57326722892871 | -7.16378786129443 | 2.37112831336983 |
| C | -0.87861008391988 | -6.87266998593603 | 1.98810808426414 |
| H | -1.04661394813780 | -7.75574054032121 | 1.36186352014739 |
| H | -1.57458581812693 | -6.09034853547953 | 1.66729790960192 |
| H | -1.13344949368771 | -7.13928931131678 | 3.02158788025389 |
| H | 1.26204315614693 | -3.11618342709847 | 0.81048772097987 |
| H | 4.02603097134394 | -1.70771291496944 | 0.70820705252916 |
| H | 2.41784209624623 | -0.95630258262160 | 0.52787224586473 |
| H | 1.85220139713757 | -1.50837367151698 | 2.86909372378848 |
| H | 3.18475422327100 | -0.38932388854171 | 2.69692791360138 |
| H | 3.51322482524315 | -3.39504425878132 | 3.24452724881997 |
| H | 4.82802068216947 | -2.22955474069960 | 3.12571012168118 |
| H | 4.19689914498206 | -2.79477396661492 | 5.49766986038331 |

283

I-1 ($r^2$SCAN-3c)

| | | | |
|---|---|---|---|
| P | -1.66247994885110 | 0.58038883230242 | -1.36163322580973 |
| N | -1.29092286175654 | 1.45912970084822 | -0.11020171695189 |
| P | -0.39319805944095 | 1.36493699159705 | 1.17666643932985 |
| N | 0.09712800161055 | -0.04811702541633 | 1.78208078309077 |
| S | -0.97482406708110 | -1.19249911422746 | 2.10763814510632 |
| O | -0.38244628933191 | -2.52630357805066 | 1.88885187420757 |
| O | -2.34415399454291 | -0.94183682920254 | 1.62381375373970 |
| C | -1.13266635005611 | -1.13242317782992 | 4.00008384784950 |
| F | -1.99516853768352 | -2.08290437374453 | 4.39421066774743 |
| F | 0.05460144681323 | -1.36691736080853 | 4.58587117565709 |
| F | -1.58489350751258 | 0.05609385980439 | 4.41474198229917 |
| O | 0.93170208355609 | 2.23220803528978 | 0.85619071030312 |
| C | 1.62121236806400 | 2.99047025724346 | 1.79390963164862 |
| C | 0.95382058611112 | 4.02192849838504 | 2.43722699020554 |
| C | 1.62347810470468 | 4.72820352119451 | 3.48324609582642 |
| C | 0.96461363172417 | 5.64858048371605 | 4.33386602502712 |
| C | 1.65348969737521 | 6.30696243130493 | 5.32555881668468 |

| | | | |
|---|---|---|---|
| C | 3.03531857579212 | 6.08811287640775 | 5.50937373812853 |
| C | 3.69832944294582 | 5.18951816887344 | 4.71017098495587 |
| C | 3.01010693082939 | 4.47672223289218 | 3.69678110450633 |
| C | 3.66990065130334 | 3.52914521594357 | 2.89389011876457 |
| C | 3.01688445792786 | 2.73844153903130 | 1.96226074290824 |
| C | 3.82498332653346 | 1.80339082240403 | 1.14162382075576 |
| C | 5.05089439722610 | 1.32999568375790 | 1.66208719271368 |
| C | 5.96166460377740 | 0.70817949723172 | 0.83114148860988 |
| C | 5.65474384894583 | 0.47824250477621 | -0.52248942222504 |
| C | 4.40614815079500 | 0.83130748440092 | -1.01863965348637 |
| C | 3.50823059128504 | 1.50080339497134 | -0.19388678942123 |
| H | 2.57054822634691 | 1.82597866282975 | -0.61808319214418 |
| H | 4.13311919407112 | 0.63137312207497 | -2.04918206400698 |
| C | 6.82309412328336 | -0.11375167805732 | -1.16611845420772 |
| C | 7.05114840996729 | -0.48172590197341 | -2.49178683412271 |
| C | 8.28543331288057 | -1.02942491610010 | -2.83380092904740 |
| C | 9.28078142644009 | -1.19835646326012 | -1.86854583870514 |
| C | 9.06236062028312 | -0.80430908896282 | -0.54623003791911 |
| C | 7.83463424040924 | -0.26024356313602 | -0.19931528540296 |
| C | 7.38422258702789 | 0.28057802750719 | 1.14300292317900 |
| C | 7.48754345035043 | -0.73677655523545 | 2.32449985328877 |
| C | 8.63854858931196 | -0.24331725117197 | 3.20818018081793 |
| C | 8.51036743961775 | 1.27723798496997 | 3.10149621386278 |
| C | 8.27786258645635 | 1.48841393419946 | 1.60329003684183 |
| H | 7.81104590050879 | 2.44585922836822 | 1.35658196387372 |
| H | 9.23671884546433 | 1.43934665253312 | 1.07609468648397 |
| H | 9.38668874096128 | 1.81925485593041 | 3.47113036946860 |
| H | 7.63735400079220 | 1.61303973053411 | 3.67533652169997 |
| H | 8.56602974340451 | -0.61668029704415 | 4.23475736095494 |
| H | 9.60591616856714 | -0.56327299586720 | 2.80035568537182 |
| H | 7.61161958006086 | -1.76304379770506 | 1.96626906511821 |
| H | 6.55884797829077 | -0.69171280340190 | 2.90201400354053 |
| H | 9.84903589100268 | -0.92125367698679 | 0.19472915623061 |
| H | 10.23603011996043 | -1.62986983148523 | -2.15131886040253 |
| H | 8.47824206293134 | -1.32339605301604 | -3.86107527983435 |
| H | 6.28337104838537 | -0.33677058460096 | -3.24569143470890 |
| H | 5.28735900910765 | 1.47683057004275 | 2.71288094959747 |
| H | 4.74210171765387 | 3.42824963210009 | 3.01833102919279 |
| H | 4.75859737927515 | 4.99823210788183 | 4.84991822319784 |
| H | 3.56706818497305 | 6.62230561121005 | 6.29014526427710 |
| H | 1.12810591801187 | 6.99820958011047 | 5.97674625071106 |
| H | -0.09801628210840 | 5.82133793966902 | 4.20656408824145 |
| C | -0.42348925532420 | 4.41574210816121 | 2.05531108431123 |
| C | -1.44597076843921 | 3.49486243344917 | 2.09075874755495 |
| C | -2.80550458946281 | 3.79936208651066 | 1.83385065381626 |
| C | -3.09056090548281 | 5.08805376931549 | 1.43528057405736 |
| C | -2.08283876801879 | 6.06738841100016 | 1.29824128174962 |
| C | -2.39584879649151 | 7.37562645742422 | 0.85492936069357 |
| C | -1.41278424011098 | 8.32091399273142 | 0.69337391208044 |
| C | -0.07010608563607 | 7.99218795377553 | 0.97186955060265 |

| | | | |
|---|---|---|---|
| C | 0.26360803914139 | 6.73941093862216 | 1.43083039430837 |
| C | -0.72752773478629 | 5.74628316626102 | 1.62289963481497 |
| H | 1.30070102194631 | 6.49822642336283 | 1.63311564911029 |
| H | 0.70805211171981 | 8.73357617543858 | 0.82111990648794 |
| H | -1.66398203010854 | 9.31786145096047 | 0.34562350846701 |
| H | -3.43330136546333 | 7.61018935475140 | 0.63580097633087 |
| H | -4.12191353789725 | 5.36735269980154 | 1.23863609971544 |
| C | -3.88715110372838 | 2.82272416556480 | 2.06922914488079 |
| C | -4.92956538727252 | 2.67447994737437 | 1.14290113767421 |
| C | -6.01152158523222 | 1.87092133671940 | 1.46199505104846 |
| C | -6.07147849286364 | 1.21200749269082 | 2.70046543105153 |
| C | -5.02342740263390 | 1.32386846901707 | 3.61135964357953 |
| C | -3.93789196216678 | 2.12587436561404 | 3.28741017164983 |
| H | -3.13004766258561 | 2.24699786880313 | 4.00005347981938 |
| H | -5.05335127139670 | 0.80827259436941 | 4.56664438187638 |
| C | -7.34063071018257 | 0.49873743799984 | 2.78275842482305 |
| C | -7.88858492415794 | -0.24712160885928 | 3.82312260586990 |
| C | -9.15119305332394 | -0.80925169963954 | 3.65301061019183 |
| C | -9.84983913755699 | -0.62786937127330 | 2.45912336164774 |
| C | -9.30262440093899 | 0.12618298402737 | 1.41684437666453 |
| C | -8.04595178570212 | 0.69449330773249 | 1.57803295273791 |
| C | -7.23942861380627 | 1.56748515000334 | 0.63051355568155 |
| C | -8.00141163989602 | 2.82446171043660 | 0.14247140946298 |
| C | -8.81137666744215 | 2.37530240505861 | -1.09103525343824 |
| C | -8.12317791060759 | 1.08460691997710 | -1.61222766679319 |
| C | -6.89890852937429 | 0.87189652061763 | -0.70988572751715 |
| H | -6.63684668337437 | -0.18131625457857 | -0.57767172118426 |
| H | -6.02540538280714 | 1.37743672299174 | -1.13235123538970 |
| H | -7.82640115147428 | 1.16552941751871 | -2.66317791819505 |
| H | -8.80416540582915 | 0.23059885596164 | -1.54473976388284 |
| H | -9.85342536725998 | 2.17680656799995 | -0.82294550127248 |
| H | -8.82781909031916 | 3.16202743005867 | -1.85157560656997 |
| H | -8.61940319618188 | 3.25887993562736 | 0.93372873194666 |
| H | -7.25720425123161 | 3.57324427261699 | -0.14951122463281 |
| H | -9.86964506412037 | 0.26074317618439 | 0.50203283437789 |
| H | -10.83276967318047 | -1.07284079905362 | 2.33886422646424 |
| H | -9.59484516408586 | -1.39407664006770 | 4.45293981243539 |
| H | -7.34185434804185 | -0.38854392715975 | 4.75083498269427 |
| H | -4.87543817899914 | 3.19074635453243 | 0.18701575778591 |
| O | -1.10665415631753 | 2.17354007022812 | 2.38925882202849 |
| N | -1.05411803136172 | -0.88428158525919 | -1.60910758269306 |
| S | 0.43208258585497 | -1.19997801497065 | -2.06938323485084 |
| O | 0.89900753307579 | -2.46953326171926 | -1.48207421717710 |
| O | 1.36296576853847 | -0.05333911277994 | -2.07584682976648 |
| C | 0.19489808484583 | -1.65379029965665 | -3.89847066803175 |
| F | -0.47256900792220 | -0.69303243271744 | -4.55156714772678 |
| F | 1.39714190151786 | -1.81281856611429 | -4.48063155373407 |
| F | -0.48713577032355 | -2.80284583157070 | -4.01042209008785 |
| O | -1.44891606004824 | 1.48136183284413 | -2.70697925126551 |
| C | -2.11495212953900 | 2.71072635112488 | -2.67464033570670 |

| | | | |
|---|---|---|---|
| C | -3.48811091439429 | 2.70247571224940 | -2.83686076307355 |
| C | -4.20401144750759 | 3.91947881395495 | -2.62557626062775 |
| C | -5.61800964794244 | 3.99082513431235 | -2.64001183252610 |
| C | -6.26688932678947 | 5.18060030889118 | -2.40606946257898 |
| C | -5.53571787889140 | 6.36074845083203 | -2.15633143506457 |
| C | -4.16304635998308 | 6.32282939655453 | -2.12736418153212 |
| C | -3.46410242378813 | 5.10937012304426 | -2.34007270504739 |
| C | -2.05502815069114 | 5.06276585705232 | -2.29265299047313 |
| C | -1.35297723242801 | 3.88469510673191 | -2.45123079813185 |
| C | 0.12412854020284 | 3.88620224085433 | -2.40908762675019 |
| C | 0.88791873729865 | 3.24019607998577 | -3.39717111967657 |
| C | 2.26925112007943 | 3.35400202432960 | -3.37470275722786 |
| C | 2.90428390932130 | 4.05580385277312 | -2.32970143948217 |
| C | 2.15605628144021 | 4.69793379818335 | -1.34828364794235 |
| C | 0.77126328194494 | 4.62118522256727 | -1.40747156500855 |
| H | 0.17266090399001 | 5.10600114136971 | -0.64598857163699 |
| H | 2.63884090546004 | 5.23510623644585 | -0.53654176795404 |
| C | 4.34657746133920 | 3.90186480621421 | -2.47030563749958 |
| C | 5.38997930918447 | 4.31816511752790 | -1.64703195272269 |
| C | 6.69175189519848 | 3.95200271368700 | -1.98124417664919 |
| C | 6.94474347116446 | 3.19053584662946 | -3.12404732507850 |
| C | 5.89777145545878 | 2.78264988423917 | -3.95365909013776 |
| C | 4.59844192646544 | 3.13160310635220 | -3.61854021346012 |
| C | 3.30898883890492 | 2.80969677311490 | -4.34412312755271 |
| C | 3.24170176495686 | 3.52576540073248 | -5.72271270950090 |
| C | 2.11304025004100 | 2.81484969343311 | -6.47305270567921 |
| H | 2.23078429441711 | 2.89833391040305 | -7.55752925415631 |
| H | 1.15029444653005 | 3.27055922045425 | -6.22068487272948 |
| C | 2.16402018636067 | 1.34617556104817 | -5.98071829012404 |
| C | 3.10401383417890 | 1.33114507103727 | -4.74900905133132 |
| H | 4.07834680983900 | 0.91127999284738 | -5.02317157616866 |
| H | 2.70406344499840 | 0.73912189764544 | -3.92322027976884 |
| H | 1.16509063856358 | 0.98655824729471 | -5.72087675292994 |
| H | 2.54475410425376 | 0.67811190508917 | -6.75901326355408 |
| H | 3.09574329980835 | 4.60525418396450 | -5.61879516174420 |
| H | 4.19935697773787 | 3.35967937792117 | -6.23139419070823 |
| H | 6.10604730006941 | 2.18631263710724 | -4.83820157432452 |
| H | 7.96383950481652 | 2.90271833934909 | -3.36326846355066 |
| H | 7.51762832280384 | 4.25310143232820 | -1.34399352823736 |
| H | 5.19434890105961 | 4.89785810267199 | -0.74938349474971 |
| H | 0.38392166930735 | 2.66672590720585 | -4.16697294562658 |
| H | -1.50893998216973 | 5.98739203788384 | -2.12846023719470 |
| H | -3.58656776623887 | 7.22223612363267 | -1.93260975780318 |
| H | -6.06107959677445 | 7.29493521657750 | -1.98545783838203 |
| H | -7.35198422474496 | 5.21139136804441 | -2.41214425971058 |
| H | -6.19093826965230 | 3.09085989122640 | -2.83434124987388 |
| C | -4.15201630624354 | 1.44231868641115 | -3.24690511874169 |
| C | -3.97215791505793 | 0.28537382535618 | -2.50810652947316 |
| C | -4.48782732309414 | -0.97521640955787 | -2.89432930187836 |
| C | -5.26564582938417 | -1.01085888063355 | -4.03562423235528 |

| | | | |
|---|---|---|---|
| C | -5.49198605861503 | 0.12813476196686 | -4.83366921799074 |
| C | -6.26402317614719 | 0.04948602401229 | -6.01947454923115 |
| C | -6.40861158400156 | 1.14204875449880 | -6.83791544019043 |
| C | -5.76647248882045 | 2.35450702587375 | -6.50958779509730 |
| C | -5.02483017567444 | 2.46729256960312 | -5.35741017382335 |
| C | -4.89105088706886 | 1.37204517644122 | -4.46809062390465 |
| H | -4.53066660948421 | 3.40339856677948 | -5.12682971883746 |
| H | -5.85312222247619 | 3.20592935800735 | -7.17715441601969 |
| H | -6.99789209590018 | 1.07124371601552 | -7.74645699401528 |
| H | -6.72668574144792 | -0.89978169258849 | -6.27417011978333 |
| H | -5.70240978751708 | -1.95704698933011 | -4.34168580634080 |
| C | -4.19859640075093 | -2.25726840158466 | -2.20598482037529 |
| C | -4.53235407032084 | -2.50170915703772 | -0.86490175694281 |
| C | -4.40808879726610 | -3.78894310115863 | -0.35550605604814 |
| C | -3.87718491847405 | -4.81703575921455 | -1.16070988863289 |
| C | -3.48665752911007 | -4.56775571058311 | -2.47200710365342 |
| C | -3.67139704539753 | -3.29424676968360 | -2.99140184624036 |
| H | -3.39174425804335 | -3.08214895133415 | -4.01824933965645 |
| H | -3.05922994070918 | -5.35354089761450 | -3.08805928385744 |
| C | -3.85095495979417 | -6.05564340805252 | -0.39196038274549 |
| C | -3.41064253247694 | -7.33423272130143 | -0.73313508052209 |
| C | -3.50078382751142 | -8.34888529195990 | 0.21712649987162 |
| C | -4.02537081515113 | -8.08963079627164 | 1.48540676443985 |
| C | -4.47152179235539 | -6.81017991962456 | 1.82284957627445 |
| C | -4.38099612232577 | -5.79413495061272 | 0.88214958734354 |
| C | -4.83075252064856 | -4.35478125711053 | 0.99484368745429 |
| C | -4.28408333867792 | -3.59403561823743 | 2.22228930546383 |
| C | -5.25964957120317 | -2.42145278829501 | 2.45381700238973 |
| C | -6.57746221153892 | -2.80709192889299 | 1.73130700123618 |
| C | -6.36320610981558 | -4.23652541975938 | 1.21724233595224 |
| H | -6.64401777260543 | -4.96372763684059 | 1.98861801491567 |
| H | -6.93097368268695 | -4.47079027472004 | 0.31142471117656 |
| H | -6.77134451018475 | -2.12563505384836 | 0.89760864479591 |
| H | -7.44809357467037 | -2.74208956995877 | 2.38866052111882 |
| H | -4.84613880643908 | -1.49271977806515 | 2.05269642854745 |
| H | -5.41843306576194 | -2.25176014665250 | 3.52336852681811 |
| H | -4.30288449732015 | -4.27874820078852 | 3.07794563732890 |
| H | -3.25289028188524 | -3.26923102188872 | 2.07269587494467 |
| H | -4.88330823220665 | -6.61994381113152 | 2.81037377053721 |
| H | -4.08587709371536 | -8.89085444091630 | 2.21558320866512 |
| H | -3.16238614749731 | -9.35069980021193 | -0.02946700093243 |
| H | -3.00532567766761 | -7.53930558886935 | -1.72015200429059 |
| H | -4.90969253389696 | -1.68919093349262 | -0.25572374146147 |
| O | -3.26677326131834 | 0.37841780634703 | -1.30810098026096 |
| H | 2.88543554217816 | -2.48953860818586 | 5.19434076798422 |
| C | 3.91427329056615 | -2.30539754269420 | 4.86069356357648 |
| C | 4.33420913278100 | -0.91296950220210 | 5.25648096633759 |
| C | 3.41335228509551 | 0.13949743275837 | 5.29934557424569 |
| C | 3.81701850409653 | 1.42981101277766 | 5.63046761661098 |
| C | 5.15194009815759 | 1.69034586899876 | 5.93047034607553 |

| | | | |
|---|---|---|---|
| C | 6.07790439056177 | 0.64987780741287 | 5.90101856115216 |
| C | 5.66990199434661 | -0.63844868794500 | 5.56525080432376 |
| H | 6.39640639055021 | -1.44748399167184 | 5.55019928632400 |
| H | 7.11830492105653 | 0.83944937775913 | 6.14837699089916 |
| H | 5.46520181484447 | 2.69515724170437 | 6.19718732902181 |
| H | 3.08732424835716 | 2.23316317715004 | 5.65610006090132 |
| H | 2.36856118379869 | -0.05476677439835 | 5.07387149299925 |
| C | 3.98961125601845 | -2.54958190147030 | 3.34202127279154 |
| C | 2.98575869267141 | -1.70248169819644 | 2.56928632255438 |
| C | 2.98795120438357 | -1.97135898438783 | 1.05039041031971 |
| C | 2.41924932129297 | -3.30185693417443 | 0.75636313919860 |
| N | 3.12699350335403 | -4.32240433474847 | 0.41645680483632 |
| O | 4.46350704080543 | -4.24999305890365 | 0.32962168698142 |
| Si | 5.33515991154042 | -4.84519535597532 | -1.08077906206714 |
| C | 5.39777685003155 | -6.70821679617965 | -1.02912846959813 |
| H | 6.14847498021497 | -7.07050421013285 | -1.74070926149905 |
| H | 4.43655638682400 | -7.16160953178223 | -1.28223200750801 |
| H | 5.68977986674820 | -7.05064867845506 | -0.03074551346598 |
| C | 6.98764984332435 | -4.09451293510439 | -0.66713630186819 |
| H | 7.28659231244878 | -4.37231118018319 | 0.34938206540120 |
| H | 7.75747443376935 | -4.45868538317676 | -1.35522520535027 |
| H | 6.96663314546726 | -3.00156673656313 | -0.73215350982056 |
| C | 4.56692511345940 | -4.14181603374015 | -2.65943513048541 |
| C | 5.65012618523276 | -4.27366378779009 | -3.75094037254796 |
| H | 6.53823229211789 | -3.67633919871198 | -3.51678120270230 |
| H | 5.96254042922788 | -5.31409592416621 | -3.90354412760869 |
| H | 5.24586951732000 | -3.90869233693040 | -4.70424045974067 |
| C | 4.23155567593917 | -2.65604493139456 | -2.46080282512655 |
| H | 3.36987407865712 | -2.51569038580889 | -1.80367987731644 |
| H | 5.07804171642096 | -2.10128235654286 | -2.04745398299093 |
| H | 3.97597094697010 | -2.19881834471990 | -3.42640319004495 |
| C | 3.30915664419798 | -4.90059762125372 | -3.11029375055880 |
| H | 2.49356098481471 | -4.80691415253320 | -2.38699577290846 |
| H | 3.50967138396930 | -5.96581641047420 | -3.27010818005623 |
| H | 2.95206773723842 | -4.48101272114452 | -4.06015004585461 |
| O | 2.68028946319513 | -5.56181331825519 | 0.12881557282716 |
| Si | 1.09273928252797 | -6.28726855055703 | 0.37653992983985 |
| C | 1.36761302777069 | -7.78707847948052 | -0.69550352420515 |
| H | 0.50323939589787 | -8.45699620467569 | -0.62866758352292 |
| H | 1.47620206346803 | -7.48244009191207 | -1.74175377789499 |
| H | 2.25940305866075 | -8.34814638716473 | -0.40191481071654 |
| C | -0.27231637148330 | -5.23289635600330 | -0.30365630660270 |
| H | -0.00481625257488 | -4.80790626479268 | -1.27561505316663 |
| H | -1.13003239151325 | -5.90069012092305 | -0.45205994173893 |
| H | -0.60576505339935 | -4.41638744870461 | 0.34260800755336 |
| C | 0.91498264093145 | -6.70997775643569 | 2.20933283897202 |
| C | 0.95374789407459 | -5.45681635642483 | 3.09796109599332 |
| H | 0.77591653609870 | -5.74515994535549 | 4.14279096898220 |
| H | 1.93066791940151 | -4.96225197844320 | 3.06109359909331 |
| H | 0.18768508092126 | -4.72744012385538 | 2.81939859363419 |

| | | | |
|---|---|---|---|
| C | 2.02773881248442 | -7.67748754262850 | 2.64076946484982 |
| H | 1.91374408110807 | -7.91971329873621 | 3.70566149718507 |
| H | 1.99060606585090 | -8.61805475779841 | 2.08103478497373 |
| H | 3.02201618114202 | -7.23691574527723 | 2.50344732972545 |
| C | -0.45816782203959 | -7.39095894066941 | 2.36877971400390 |
| H | -0.55067571400621 | -8.28971300565192 | 1.74815255159518 |
| H | -1.28127479993353 | -6.71636955587193 | 2.10884187496609 |
| H | -0.59754507559435 | -7.69567499275901 | 3.41468484166927 |
| H | 1.35298569133870 | -3.46867465330193 | 0.86180325155337 |
| H | 3.99478886850217 | -1.86111385598945 | 0.64103619353920 |
| H | 2.32922945000817 | -1.24038236645076 | 0.56873858548977 |
| H | 1.97841840730435 | -1.87447295317416 | 2.96032042519440 |
| H | 3.19958609683442 | -0.63705520393453 | 2.69920012740012 |
| H | 3.80144591496151 | -3.61537856773068 | 3.15774250855243 |
| H | 5.00621339714123 | -2.34452079994379 | 2.98349398979127 |
| H | 4.55601174824434 | -3.03318344809578 | 5.36954529596146 |

516

I-2 (r$^2$SCAN-3c/xTB)

| | | | |
|---|---|---|---|
| P | -6.99924739249037 | 0.98737668744637 | 1.55585511758646 |
| O | -7.31646033450730 | 2.52681603694143 | 1.37170809911686 |
| O | -8.12780757871074 | 0.47166229976900 | 2.61145852311500 |
| C | -8.08185482722958 | 3.20639071399858 | 2.31899200675925 |
| C | -7.49129173149300 | 4.25930415483398 | 3.05817780039621 |
| C | -8.30320555305326 | 5.01566602652454 | 3.87330183001592 |
| C | -9.66513560669321 | 4.72419621079863 | 4.05819930884116 |
| C | -10.47358371167874 | 5.49703960293140 | 4.92046530984448 |
| C | -11.77331234648967 | 5.14995223798768 | 5.15767935309674 |
| C | -12.30849812607081 | 3.99836113428260 | 4.56035892512375 |
| C | -11.55538466704740 | 3.24071794029060 | 3.70583893206698 |
| C | -10.22429519998916 | 3.59995782249041 | 3.39796430425718 |
| C | -9.41130794736054 | 2.87317270651362 | 2.48047716948120 |
| C | -9.98043063756581 | 1.70435455621368 | 1.77700913261068 |
| C | -11.14447914720907 | 1.80560265506372 | 0.96747039757391 |
| C | -11.71795048336336 | 3.04305269535604 | 0.60099461821597 |
| C | -12.82666833533987 | 3.09159148101686 | -0.19924803668884 |
| C | -13.42893546111452 | 1.90844220021786 | -0.65456816926894 |
| C | -12.89126187168982 | 0.69588950685473 | -0.32522509024790 |
| C | -11.72590774308680 | 0.61230899355538 | 0.46752752161127 |
| C | -11.14569499694269 | -0.62617640645047 | 0.79404191155248 |
| C | -9.98053774125609 | -0.72808320665680 | 1.52267993929930 |
| C | -9.38729466139691 | 0.47708101062301 | 1.98028405540445 |
| H | -7.87911016440487 | 5.85529157749172 | 4.40527231863143 |
| H | -10.03599944633715 | 6.36275689771408 | 5.39615496183986 |
| H | -12.38814626920190 | 5.74352915967545 | 5.81754829169301 |
| H | -13.32434388997448 | 3.70784796885467 | 4.78347647618801 |
| H | -11.97800739971519 | 2.34976173759001 | 3.26825469625826 |
| H | -11.26795612033054 | 3.95738921180228 | 0.95564467294751 |
| H | -13.25006463902963 | 4.04489604524006 | -0.48031735986992 |
| H | -14.31753568425673 | 1.96643999548792 | -1.26590881081304 |
| H | -13.34259303253600 | -0.22229763269691 | -0.67144737335286 |

| | | | |
|---|---|---|---|
| H | -11.64325561452640 | -1.52380870150163 | 0.45634324987197 |
| N | -7.34394033742530 | 0.21241244655849 | 0.21007206700266 |
| P | -6.33850606593140 | -0.67274268804699 | -0.64002647140426 |
| O | -6.54303943791193 | -0.22499816887298 | -2.18122689380357 |
| O | -6.83531319846770 | -2.17983758843988 | -0.58227846212454 |
| C | -7.90374926505985 | -0.29658961673535 | -2.51793302356888 |
| C | -8.70318106123227 | 0.87013726975358 | -2.54691621761665 |
| C | -10.05330310914846 | 0.71310684174463 | -2.76259105733150 |
| C | -10.63752745188847 | -0.55280830968269 | -2.95571326737437 |
| C | -12.02457242749197 | -0.69913109693131 | -3.17488820397504 |
| C | -12.58041839618884 | -1.93464719722276 | -3.35698648759651 |
| C | -11.77073572732707 | -3.08052658468166 | -3.33327423118735 |
| C | -10.41953371448096 | -2.97033739396005 | -3.14853288854868 |
| C | -9.81411769572177 | -1.70927725177117 | -2.96464667053188 |
| C | -8.41762246161849 | -1.54816488663081 | -2.75256554586121 |
| C | -7.49053061342330 | -2.70238409679819 | -2.79850796270245 |
| C | -7.34394198793835 | -3.42540389281296 | -4.01250220314325 |
| C | -7.93550640409927 | -3.02007039265649 | -5.22734872926490 |
| C | -7.71142138098896 | -3.72384396699069 | -6.37898690925542 |
| C | -6.89721220464461 | -4.86668868160653 | -6.37095990484370 |
| C | -6.31290998807078 | -5.28334990268270 | -5.20842514738543 |
| C | -6.51294822432962 | -4.57062840646225 | -4.00680484133852 |
| C | -5.92546473807324 | -4.97995610731985 | -2.79886614491758 |
| C | -5.99914673808243 | -4.25588629251915 | -1.62440166838996 |
| C | -6.75041910450383 | -3.04017097297372 | -1.68346414628982 |
| H | -10.68996099412998 | 1.58554121395191 | -2.80281688689857 |
| H | -12.63605953934861 | 0.19090372049307 | -3.19556581171337 |
| H | -13.64277803752601 | -2.03812248430005 | -3.52195393483488 |
| H | -12.22048587531543 | -4.05302311754193 | -3.47009454806990 |
| H | -9.79898732090760 | -3.85285922341148 | -3.14476619947629 |
| H | -8.55007206628900 | -2.13247870541842 | -5.24550449777763 |
| H | -8.15762078762944 | -3.39704103984079 | -7.30651010360394 |
| H | -6.73202237758901 | -5.40791335665991 | -7.29045689611922 |
| H | -5.67473406973823 | -6.15481177154512 | -5.19405041412071 |
| H | -5.39785335742729 | -5.91967889231033 | -2.82426838098588 |
| N | -5.58509050792635 | 0.87479952335456 | 2.34879847300274 |
| N | -4.73707834173605 | -0.60536207204649 | -0.40794783371835 |
| S | -5.23755684162483 | -0.58257897642065 | 2.74945856633905 |
| O | -5.93832200697512 | -1.61378422856677 | 2.04375950534367 |
| O | -3.83630780601338 | -0.81069350508655 | 3.03104513824968 |
| S | -4.19967472268883 | 0.85383942481306 | -0.52542505105739 |
| O | -5.15855148984091 | 1.90967806023379 | -0.36632638883338 |
| O | -2.91139175633912 | 1.00433090967853 | 0.10042517204100 |
| C | -3.60865008881359 | 1.04606566734803 | -2.35059492824147 |
| C | -5.83443074617622 | -0.74349947719474 | 4.57538622445050 |
| F | -2.45783309910584 | 0.37459476179923 | -2.51263900586044 |
| F | -4.41154265973169 | 0.64664495326510 | -3.33807761277994 |
| F | -3.33575793444436 | 2.33645862725003 | -2.58555414029446 |
| F | -7.05900449903558 | -0.32915956957940 | 4.90489793875189 |
| F | -4.99105112679787 | -0.04831467698126 | 5.35590829254060 |

| | | | |
|---|---|---|---|
| F | -5.73822275123436 | -2.02707217608914 | 4.95724486690463 |
| C | -6.97078945576024 | -7.89655859998834 | 3.53548403239475 |
| C | -7.32388075721512 | -8.55931905299832 | 2.37039554741875 |
| C | -8.08057028954073 | -7.92609720987567 | 1.39244249709058 |
| C | -8.45583803224209 | -6.61398326646375 | 1.58718151672940 |
| C | -8.08279914661802 | -5.93700129034945 | 2.75657674330808 |
| C | -7.35301736292482 | -6.58078176988866 | 3.74237214284095 |
| H | -6.38416410456576 | -8.40833295458981 | 4.28490324396853 |
| H | -7.00990684113225 | -9.58257662588601 | 2.22310882645995 |
| H | -8.35776748212034 | -8.45586750490522 | 0.49221555435481 |
| H | -7.07470966288328 | -6.06370862367239 | 4.64912252919210 |
| C | -9.26712418378279 | -5.71496572370646 | 0.68646211073870 |
| C | -9.13653176744539 | -4.38250130352191 | 1.39198963663731 |
| C | -9.56836414342137 | -3.13631268210044 | 0.99565502201738 |
| C | -9.44734447655024 | -2.05217698698241 | 1.87516542807911 |
| C | -8.90776618196686 | -2.26101410693445 | 3.14421063637260 |
| C | -8.42691964138471 | -3.49896208302830 | 3.52971673364834 |
| C | -8.53236685555435 | -4.55983563748658 | 2.64774066251442 |
| H | -9.98449351948175 | -2.98284860866231 | 0.00921485954051 |
| H | -8.88128556836103 | -1.43941079221575 | 3.84160341361316 |
| H | -7.98821514494000 | -3.63068001551054 | 4.50867889193905 |
| C | -8.82340976847979 | -5.73736011091171 | -0.78967636666774 |
| H | -8.72504104658931 | -4.71359406676562 | -1.14785796297135 |
| H | -7.85672064102119 | -6.22596282163458 | -0.90376585779836 |
| C | -10.75903774941715 | -6.16135086742189 | 0.65921304841569 |
| H | -11.36766039912583 | -5.55593662035991 | 1.33029768905222 |
| H | -10.82977204697192 | -7.20154690025670 | 0.98243425312139 |
| C | -9.93471139008195 | -6.45563126237733 | -1.55558256845902 |
| H | -9.80409330661448 | -7.53700834136535 | -1.48734511764016 |
| H | -9.95561845713461 | -6.18102175876854 | -2.60995700676358 |
| C | -11.19169962236382 | -6.04150949757079 | -0.79976087250845 |
| H | -12.05265008562923 | -6.66938923805107 | -1.02696543034895 |
| H | -11.44147935834730 | -5.00561293288292 | -1.03484558611224 |
| C | -6.99043746500010 | 2.50847448333868 | -3.23971417174341 |
| C | -8.11825749589615 | 2.21660035716795 | -2.47309845589166 |
| C | -8.75253832526510 | 3.23658675482558 | -1.75946822771104 |
| C | -8.27692351877840 | 4.52565586795776 | -1.86018334880039 |
| C | -7.15605947417661 | 4.81007634627978 | -2.65360042873864 |
| C | -6.49523953932240 | 3.79614908167983 | -3.32745193888843 |
| H | -6.52084509135687 | 1.71676508661644 | -3.80232893304174 |
| H | -9.60935865168950 | 2.99943075249641 | -1.14439825169063 |
| H | -5.62330377873385 | 4.00324194364365 | -3.93169254666448 |
| C | -8.83350954743484 | 5.79383792200277 | -1.25476965537628 |
| C | -7.91295973718460 | 6.84385444297351 | -1.83220634575263 |
| C | -7.93200542778458 | 8.21411476127661 | -1.67407419314356 |
| C | -6.97311945087866 | 8.98170021546483 | -2.32402399336718 |
| C | -6.01177523299581 | 8.38938458404501 | -3.12751033211496 |
| C | -5.98323857535163 | 7.01357305698036 | -3.29413343487532 |
| C | -6.93117852307262 | 6.24517891092011 | -2.63825140221254 |
| H | -8.67783746475415 | 8.69757481009265 | -1.06266906659750 |

| | | | |
|---|---|---|---|
| H | -6.98137732773221 | 10.05566663156933 | -2.20616652201937 |
| H | -5.28174221232268 | 9.00699237464869 | -3.63147316095118 |
| H | -5.23967186190047 | 6.54841730706657 | -3.92527162600553 |
| C | -10.31879583118018 | 6.02411659213978 | -1.59330047167978 |
| H | -10.43293198287382 | 6.49845534831583 | -2.56819515786377 |
| H | -10.82219633547458 | 5.05708298172663 | -1.62625903217885 |
| C | -8.82898032335316 | 5.77871558774963 | 0.28259512090929 |
| H | -7.85225891676983 | 6.04294040111442 | 0.68745969293245 |
| H | -9.06347849045903 | 4.76891038483534 | 0.61248000173082 |
| C | -10.89493082807494 | 6.88357546587968 | -0.45956647495411 |
| H | -11.89396312729515 | 6.54264920110790 | -0.18828047193769 |
| H | -10.98126889938444 | 7.92328942398846 | -0.77503439804917 |
| C | -9.92082934357039 | 6.75688847095553 | 0.72611689424159 |
| H | -10.42057328081243 | 6.39539955696713 | 1.62591977655627 |
| H | -9.48287303560487 | 7.72530996882520 | 0.96824229848112 |
| C | -3.02916108989392 | -7.47736478869968 | 5.14676115128206 |
| C | -2.31530892610564 | -8.49960095825374 | 4.53873280372846 |
| C | -2.29928916284642 | -8.63273642548107 | 3.15621500650831 |
| C | -3.00433741062262 | -7.72716829217149 | 2.39146588201821 |
| C | -3.72688984025176 | -6.69388666420438 | 3.00705606627159 |
| C | -3.74213367244188 | -6.56341268661320 | 4.38644581274611 |
| H | -3.03276749257115 | -7.39655539021934 | 6.22447113800284 |
| H | -1.76842327946758 | -9.20242004734119 | 5.14974455072879 |
| H | -1.74621172997882 | -9.43814831317876 | 2.69516319633139 |
| H | -4.30503865560589 | -5.76882701093283 | 4.85560532111927 |
| C | -3.16600062571251 | -7.67645249939254 | 0.89043812758833 |
| C | -4.04591048022879 | -6.45884057718063 | 0.72509682486573 |
| C | -4.52019474750012 | -5.89748960120700 | -0.43749136920363 |
| C | -5.40280035108202 | -4.80263066303848 | -0.38982289004796 |
| C | -5.75592145181071 | -4.31017950433314 | 0.86859246867828 |
| C | -5.22642664570601 | -4.82375269329903 | 2.03860360825249 |
| C | -4.37866405892649 | -5.91249748714460 | 1.97333077783862 |
| H | -4.19969637307682 | -6.31897338878454 | -1.37826664862174 |
| H | -6.44989466668631 | -3.50011264152937 | 0.95948321465696 |
| H | -5.49640510269175 | -4.36864211828885 | 2.97946540237211 |
| C | -1.81877820112156 | -7.58498558097141 | 0.13261033371196 |
| H | -1.60869164431869 | -6.56662019413708 | -0.19267177187370 |
| H | -1.01079675053367 | -7.89472499658971 | 0.79628781392026 |
| C | -3.85020342297031 | -8.95736446912315 | 0.33999010356271 |
| H | -4.67504593905811 | -8.67172484131556 | -0.31443935112068 |
| H | -4.25951346995847 | -9.56206978246016 | 1.14832798947921 |
| C | -1.93154691256663 | -8.56406024543291 | -1.03555852289603 |
| H | -0.95618255434825 | -8.89870110499272 | -1.38634678978952 |
| H | -2.45528314722135 | -8.09833209159920 | -1.87212521593468 |
| C | -2.77521029582113 | -9.69499223086719 | -0.45750790985081 |
| H | -2.16526555124622 | -10.30815383913355 | 0.20748489713952 |
| H | -3.20257697434672 | -10.33972119622132 | -1.22438996759279 |
| C | -5.43047059831616 | 4.75591271560538 | 4.27627378067790 |
| C | -6.05296370232357 | 4.57908859838559 | 3.03696934515295 |
| C | -5.33374680044206 | 4.80032440895357 | 1.86145974009359 |

| | | | |
|---|---|---|---|
| C | -4.03316936298709 | 5.25097506166509 | 1.95377201855711 |
| C | -3.42956913710174 | 5.45610102765512 | 3.20398707120576 |
| C | -4.11999624110332 | 5.18208648076245 | 4.37329381391335 |
| H | -5.98896024128299 | 4.54219635716553 | 5.17674712286005 |
| H | -5.79655605894834 | 4.62415475386470 | 0.90178595463348 |
| H | -3.65334000096096 | 5.30812859850392 | 5.33969181897766 |
| C | -3.08985784165044 | 5.64426340394268 | 0.84493142595449 |
| C | -1.83518788880862 | 5.98900314146264 | 1.61339413265614 |
| C | -0.58903193695229 | 6.35031867896341 | 1.14567666174855 |
| C | 0.41390963531205 | 6.66075895933007 | 2.05553272197371 |
| C | 0.16718371481958 | 6.63299927225735 | 3.41880554977512 |
| C | -1.07908432393149 | 6.26494136094485 | 3.90194366808531 |
| C | -2.07008346975137 | 5.92463960102426 | 2.99525300644243 |
| H | -0.37695954146452 | 6.39442023715080 | 0.08943005026230 |
| H | 1.39405475392538 | 6.93088880417730 | 1.68937997138956 |
| H | 0.95349007864035 | 6.89516472416116 | 4.11136617972357 |
| H | -1.27233007521554 | 6.24553776043705 | 4.96488683008904 |
| C | -2.87422657882315 | 4.56598846235851 | -0.24011727338411 |
| H | -1.84510853485343 | 4.20489468611945 | -0.20122144113548 |
| H | -3.52195549902041 | 3.70670136586434 | -0.07361889675278 |
| C | -3.61355589038911 | 6.88066620349842 | 0.07339646608468 |
| H | -3.32357775405755 | 7.81255925165433 | 0.56006447084073 |
| H | -4.70326097435616 | 6.84043098613888 | 0.02131458329763 |
| C | -3.17524307554919 | 5.23438084992487 | -1.58903371562816 |
| H | -4.20027570374312 | 5.00712834438999 | -1.88142702940438 |
| H | -2.51282477097016 | 4.87726498446874 | -2.37688827762116 |
| C | -3.03486232256454 | 6.73198749984371 | -1.32746480184439 |
| H | -3.57290715127538 | 7.33669754325307 | -2.05589756311351 |
| H | -1.98601094035945 | 7.02970368782688 | -1.33793381063613 |
| H | -1.15045547341196 | -3.90688008851775 | -2.61431301894243 |
| H | -4.48612248204029 | -2.14929274472720 | -3.00367213564846 |
| H | -5.33163907780964 | -1.35767556127587 | -5.14981604003880 |
| C | -3.91606550265280 | -2.36250919173567 | -3.90402633419835 |
| C | -4.39746247157836 | -1.90966828277175 | -5.12781776672577 |
| C | -2.20057627140396 | -3.60658728162446 | -2.50774507079471 |
| C | -2.70004820614044 | -3.04747418182945 | -3.82007834676554 |
| C | -3.67561753771646 | -2.13814594583973 | -6.29607501967090 |
| H | -4.05306485335370 | -1.77708287459017 | -7.24666391973621 |
| C | -1.97669359781970 | -3.25499481989720 | -4.99820116022528 |
| C | -2.45904795753663 | -2.81084854278992 | -6.22654484672728 |
| H | -2.76928353848903 | -4.51889586896674 | -2.28306427220849 |
| H | -3.36227456279504 | -2.30131931479178 | -1.20410323097070 |
| C | -2.33754472550276 | -2.67567544004673 | -1.29720961571850 |
| H | -1.71095606332466 | -1.78324155223660 | -1.41937353409227 |
| H | -1.02032394369570 | -3.76700117712465 | -4.94487773543189 |
| H | -1.87735376504610 | -2.98285774286568 | -7.12605918983118 |
| C | -1.99147359662803 | -3.38722200728516 | 0.00866133349570 |
| H | -2.45832471302734 | -4.37817838362413 | 0.01254388424695 |
| H | -2.45385363388632 | -3.34394296396325 | 2.12272237499776 |
| H | -0.91058443059103 | -3.52547438327348 | 0.11629067104632 |

| | | | |
|---|---|---|---|
| H | 2.29119004801114 | -3.90179812958523 | 3.01350545126278 |
| C | -2.55761605353147 | -2.68519550334435 | 1.25784400623068 |
| H | 0.75342272856624 | -6.67338532753134 | 5.04155219832251 |
| H | -3.62384407681140 | -2.48843016129021 | 1.11191626073491 |
| H | 2.56010372084449 | -4.41066456163728 | 4.67512329796534 |
| C | 2.04832154605761 | -4.67996869944467 | 3.74727467214572 |
| H | -0.80231549778180 | -5.88442434838959 | 5.34515773110169 |
| C | 0.26700961847681 | -5.70359111096978 | 5.20111382765239 |
| H | -2.04915085686937 | -2.90210892828613 | 5.96857432815208 |
| H | 2.44401305111722 | -5.64071054664697 | 3.39517376720006 |
| C | -1.94577462118164 | -1.37483418618371 | 1.59086202894335 |
| C | 0.52752060492522 | -4.80092241026806 | 3.97522981880456 |
| H | -2.42418905922898 | -4.07010314885437 | 4.68382894151283 |
| C | -1.96028121946000 | -3.10245074814529 | 4.89767206945308 |
| H | 0.67364802504674 | -5.26817222130181 | 6.11989023995778 |
| Si | -0.19214284948799 | -3.06436306031767 | 4.29595322674033 |
| H | -2.29363677613993 | -0.43138081807101 | 1.18084372136941 |
| O | -0.22775665953714 | -2.41346320001501 | 2.67880594452250 |
| N | -0.91842225760335 | -1.31105490945980 | 2.36696632152201 |
| H | -1.21969827062228 | -5.45118267910638 | 2.79815940800912 |
| C | -0.12474963691901 | -5.43912546228563 | 2.73467839548092 |
| H | -2.55285861136374 | -2.34507415783550 | 4.36883169915942 |
| H | 2.69962548712180 | 2.08083682797775 | 4.40409801884052 |
| H | 0.21248970012978 | -6.47904416653117 | 2.64333234076900 |
| H | 1.71874916482961 | 0.89430547475283 | 5.28500043346126 |
| O | -0.28842530501769 | -0.17424153841139 | 2.72659154200326 |
| H | 0.15791437165110 | -4.90353592198177 | 1.82392289027901 |
| C | 1.85091529275023 | 1.39043509341920 | 4.31933236052127 |
| H | -2.23162420262357 | 1.31885190278354 | 5.74638040265215 |
| C | 1.02347935498788 | -2.05809651101112 | 5.29324398843513 |
| C | -1.71576163629234 | 0.47841862794252 | 5.26759937743853 |
| Si | -0.96814842846755 | 1.13120549008997 | 3.67730532426564 |
| H | -1.06123547914919 | 0.00202174766720 | 5.99881062418416 |
| H | 2.10997834649960 | 0.63256144753703 | 3.57230027084051 |
| H | 1.88670627898268 | -1.87954849675708 | 4.63805244580104 |
| H | 1.35829752656169 | -2.58610896561730 | 6.19184478187787 |
| H | 0.62184216750069 | -1.08729114026474 | 5.58433437675973 |
| C | 0.60561783977209 | 2.19304740685845 | 3.91025965204151 |
| H | 0.15723605358251 | 2.77627832106123 | 5.97651127112355 |
| C | 0.30850713645067 | 3.24562989663640 | 4.99969457131512 |
| H | -2.50838363009137 | -0.21380875981090 | 4.96419263490978 |
| H | 1.15848575509784 | 3.93489247646440 | 5.06910048392113 |
| C | -2.28398508057856 | 2.09372375786839 | 2.80802692415323 |
| C | 0.89497853611988 | 2.90038124230077 | 2.57268121768976 |
| H | 1.73283877878776 | 3.59874920928628 | 2.69852749756073 |
| H | -2.32016030592920 | 3.12220949371124 | 3.17586345115828 |
| H | -3.26106432123359 | 1.63261094674450 | 2.96619957940878 |
| H | -0.57926453653562 | 3.83602286656549 | 4.75391599472751 |
| H | 0.03193493467312 | 3.47182539222226 | 2.21438713588295 |
| H | -2.13118638661074 | 2.10635552425411 | 1.72828832409304 |

| | | | |
|---|---|---|---|
| H | 1.17489603479061 | 2.16414157101497 | 1.80929692108508 |
| H | 4.20088115951558 | -5.92196881071885 | 0.00334012342414 |
| O | 6.72577195723014 | -0.76151922145491 | 1.04793030527767 |
| H | 4.12759970183372 | -4.18970705148797 | 1.75955561938754 |
| C | 5.06632701823687 | -5.30719726297437 | 0.20001887502713 |
| C | 7.58685878336339 | -7.75236096150336 | -3.75991790221868 |
| C | 7.29960274567735 | -3.66917019339629 | 0.69313141126703 |
| C | 6.22964984005246 | -5.45098810398319 | -0.53861766526970 |
| C | 7.34352962308230 | -4.63372570857547 | -0.29336257069007 |
| H | 11.11113063357994 | -6.02697382221849 | -2.16824676202146 |
| C | 5.76043247103039 | -7.30704755159559 | -2.26063275342237 |
| C | 6.29730598345152 | -8.01362821679220 | -3.32414137614335 |
| H | 9.93177176079621 | -4.74695931674663 | 0.29143338917103 |
| C | 6.53843604841309 | -6.33896268500605 | -1.64606136746239 |
| H | 10.27300160506339 | -4.43045631146534 | -3.61667821539305 |
| H | 4.75619107672936 | -7.50438100381252 | -1.91626541751596 |
| C | 5.02994919709920 | -4.34628256777336 | 1.19056990096639 |
| H | 10.89553038748928 | -3.13675862414426 | -2.60967690914223 |
| H | 7.99244811853301 | -8.31125544249734 | -4.59054923201570 |
| H | 5.70823966886664 | -8.77276932016935 | -3.81771173949935 |
| H | 9.76024725284783 | -6.41357709765244 | -0.26516754621435 |
| H | 11.79077196281448 | -4.76606893371816 | -1.16195302556987 |
| H | 8.14906277463690 | -3.03288819763891 | 0.89602613548209 |
| C | 8.45816033157512 | -4.95914753797416 | -1.25910036162244 |
| C | 7.84360872857304 | -6.06624919004168 | -2.08714396016584 |
| C | 10.87016650974259 | -5.10818746099727 | -1.63334189428973 |
| C | 9.77961045217561 | -5.37247446122319 | -0.58806987656570 |
| H | 9.36315876917353 | -6.59406640639120 | -3.51412302586157 |
| H | 8.19517776387083 | -3.55511270147338 | -2.92245414452134 |
| C | 8.36697775977007 | -6.77914300401857 | -3.14738930870824 |
| C | 8.88260485269226 | -3.74312547529172 | -2.10157657462450 |
| H | 8.88736339721677 | -2.85421980363507 | -1.46929315831308 |
| C | 10.30009615909543 | -4.04917411643767 | -2.59561890580400 |
| P | 5.50713245379816 | -0.23186612134649 | 0.15120005899682 |
| N | 4.29092954496450 | -1.26655983117179 | 0.17364657941678 |
| S | 3.00856762783579 | -0.99443823920389 | 1.02712244911819 |
| O | 2.48139882377585 | 0.33695499314524 | 0.98590153716619 |
| O | 2.98426204306009 | -1.65584126058790 | 2.30432857564733 |
| C | 1.80235362625064 | -2.00371980791356 | -0.02711204076319 |
| F | 2.14883812679585 | -2.03410929258480 | -1.31746016959292 |
| F | 1.70531455442789 | -3.27910189701775 | 0.36974005924620 |
| F | 0.56920153814515 | -1.48193489728158 | 0.02839025604733 |
| N | 6.05113278562798 | -0.07528251580733 | -1.34553161602589 |
| P | 7.02928977731457 | 1.06581262388033 | -1.82866054831968 |
| N | 7.36659528974640 | 0.86196915633819 | -3.40994825129215 |
| S | 8.14487572915233 | -0.40022434109405 | -3.78139931953513 |
| O | 9.33294526149446 | -0.73361298025980 | -3.08504699222491 |
| O | 7.33273146204914 | -1.61309984247827 | -4.10308101644582 |
| Si | 5.69186049081529 | -2.14468689650393 | -4.32221324300833 |
| C | 4.71344851863991 | -0.58447412162712 | -4.73354118543457 |

| | | | |
|---|---|---|---|
| H | 5.12353979949397 | -0.04642783537115 | -5.58453254543372 |
| H | 4.76135746180301 | 0.07961154918808 | -3.87318644529680 |
| H | 3.66665512001564 | -0.78890139988086 | -4.93863937824129 |
| C | 5.16755352600691 | -3.01496030452125 | -2.72958351410261 |
| H | 5.73432635432906 | -3.92023415271910 | -2.53303625852851 |
| H | 5.27915222357074 | -2.35210261901917 | -1.87325071780681 |
| H | 4.11856940720268 | -3.29009811277683 | -2.78270350038946 |
| C | 5.90610420440165 | -3.36187138973906 | -5.82093317052383 |
| C | 6.07689513069228 | -2.60197159748392 | -7.14561857682877 |
| H | 6.96841595452220 | -1.98132127734123 | -7.12517404496684 |
| H | 5.21560108848933 | -1.96940963447486 | -7.34354405854737 |
| H | 6.17689186252496 | -3.31095560806407 | -7.96610594577209 |
| C | 7.11738912110871 | -4.28705455607782 | -5.63221507883576 |
| H | 8.03270537848555 | -3.70603776986319 | -5.54674224314519 |
| H | 7.00606041358067 | -4.89688952937002 | -4.73890526760657 |
| H | 7.21027047526829 | -4.95430134788107 | -6.48783565435434 |
| C | 4.64475858861389 | -4.22991156480117 | -5.92518070859520 |
| H | 4.51463172764783 | -4.82874620843130 | -5.02864049138848 |
| H | 3.76178791126394 | -3.60982310809545 | -6.05214546265161 |
| H | 4.72174165442559 | -4.90077496010196 | -6.77846376368430 |
| C | 8.68636638728028 | 0.07738868414367 | -5.52058737406909 |
| F | 9.51019123044011 | 1.12284323056787 | -5.47516882627817 |
| F | 7.64499423605007 | 0.40977579965633 | -6.27719508844585 |
| F | 9.32686076569818 | -0.92084154546165 | -6.13132141445063 |
| O | 6.42921794659350 | 2.51672504771513 | -1.66213446704565 |
| C | 7.19849022544125 | 3.48331605845880 | -2.34180272903371 |
| C | 8.38048281150151 | 3.88708393295053 | -1.76543198741820 |
| C | 9.23947031913297 | 4.77076809426906 | -2.48774342506175 |
| C | 10.54917046010757 | 5.08951723181966 | -2.06712224418103 |
| C | 11.32027454432697 | 5.96753618864495 | -2.77836951577381 |
| C | 10.82634886314371 | 6.57137769555773 | -3.94470696903437 |
| C | 9.58488851505063 | 6.24069871131617 | -4.40901321975357 |
| C | 8.77540395096517 | 5.31495172859832 | -3.71428825416601 |
| C | 7.54358431669800 | 4.88637645001613 | -4.24053679721616 |
| C | 6.77193718448063 | 3.93409669749818 | -3.61217776270511 |
| C | 5.59669026578916 | 3.36550726238902 | -4.28639292192531 |
| C | 5.69892476497508 | 3.08392912552624 | -5.65214876394254 |
| C | 4.65358395925499 | 2.51259998889767 | -6.35239374094862 |
| C | 3.49210355945214 | 2.19130276613388 | -5.66738244090712 |
| C | 3.37736538325454 | 2.47331101866369 | -4.29826794038754 |
| C | 4.40834550498133 | 3.07761390452694 | -3.61121086264862 |
| H | 4.30557754860369 | 3.32388453830132 | -2.56520657180595 |
| C | 2.03705379406205 | 2.02247822635599 | -3.77739071641241 |
| C | 1.44456667154879 | 1.34164929758860 | -4.98764270928747 |
| C | 0.26421081697932 | 0.63755436633311 | -5.11147623244007 |
| C | -0.07826054256435 | 0.11164153713568 | -6.35153511847260 |
| C | 0.74876254336965 | 0.28306761893657 | -7.45134995366079 |
| C | 1.94646980371264 | 0.97067309727921 | -7.33366261670095 |
| C | 2.29146845426650 | 1.49445998109912 | -6.09805930613639 |
| H | 2.59745304380976 | 1.09218243575047 | -8.18657211979880 |

| | | | |
|---|---|---|---|
| H | 0.45907579945372 | -0.13070047631739 | -8.40630427711806 |
| H | -1.00273252156897 | -0.43936158296277 | -6.45029408092584 |
| H | -0.38915325077760 | 0.47752721035677 | -4.26822850076674 |
| C | 2.09597075058820 | 1.12703225241990 | -2.52754709981073 |
| H | 2.22479147011727 | 0.07934940161458 | -2.79737069615366 |
| H | 2.94763056010928 | 1.41182034659927 | -1.90662038933838 |
| C | 0.78367553516064 | 1.36600121556000 | -1.76164168739516 |
| H | 0.98748060852053 | 1.49139589936362 | -0.69905078820080 |
| H | 0.10941341591785 | 0.51424784549584 | -1.85165822386920 |
| C | 0.13741609180314 | 2.62012521283672 | -2.37469223472757 |
| H | -0.17449340162464 | 3.32949656652696 | -1.60775884581534 |
| H | -0.75667968820686 | 2.35178200724290 | -2.93490296919736 |
| C | 1.18328992200391 | 3.21678002961464 | -3.31512193817958 |
| H | 0.73280553907790 | 3.74363333987977 | -4.15759553555895 |
| H | 1.83283643901938 | 3.90971346976286 | -2.78349611503372 |
| H | 4.75422368887901 | 2.29907363179669 | -7.40596566651761 |
| H | 6.62758296146167 | 3.28793407520275 | -6.16344762699211 |
| H | 7.21404556721827 | 5.30065437290553 | -5.18164419535973 |
| H | 9.20473873478948 | 6.66878971296266 | -5.32487959106197 |
| H | 11.44163636234256 | 7.27836708546958 | -4.48071118391300 |
| H | 12.32071141232117 | 6.19771608276744 | -2.44387236354031 |
| H | 10.94885888651168 | 4.62253125213043 | -1.18058606761675 |
| C | 8.77685516541761 | 3.39459366294004 | -0.42669158373468 |
| C | 8.85798208778219 | 2.04286529112419 | -0.15826572424162 |
| C | 9.43722925998535 | 1.54946135239561 | 1.03979754012514 |
| C | 9.85878635733418 | 2.46632020355058 | 1.97622217986738 |
| C | 9.71424304780920 | 3.85102751528510 | 1.79169687774658 |
| C | 10.16222046378536 | 4.76960073840622 | 2.76565858581294 |
| C | 10.01966844572713 | 6.11473067506665 | 2.57597932841617 |
| C | 9.41323689755570 | 6.59393420808372 | 1.40554864075582 |
| C | 8.99521603654440 | 5.72795013823266 | 0.43202415279918 |
| C | 9.15339542229562 | 4.33252451397077 | 0.58241378409285 |
| H | 8.54670489332496 | 6.11358764541663 | -0.47054006503909 |
| H | 9.28822392818487 | 7.65862408207110 | 1.27241549993526 |
| H | 10.36672905575891 | 6.81446025905150 | 3.32140113276098 |
| H | 10.61803585260316 | 4.38177926081859 | 3.66430301704521 |
| H | 10.34865357959139 | 2.12067846590545 | 2.87438820244516 |
| C | 9.75615634286176 | 0.13012840482374 | 1.27468935688348 |
| C | 10.34043319858980 | -0.64923423197751 | 0.27528456252053 |
| C | 10.88220866584396 | -1.89035018866297 | 0.56738432434483 |
| C | 10.82591743332516 | -2.36746719742900 | 1.86754801424386 |
| C | 10.16800028652030 | -1.62594021444884 | 2.86104641420690 |
| C | 9.64464705411836 | -0.38737165190196 | 2.57077207436062 |
| H | 9.16292926865548 | 0.19877825450027 | 3.33804548946913 |
| C | 10.18550357216425 | -2.36083476756458 | 4.18097084016437 |
| C | 11.02195946459888 | -3.57255912273357 | 3.83864593343315 |
| C | 11.45728438760016 | -4.58884273944792 | 4.66357569429313 |
| C | 12.24400135392155 | -5.60087940675289 | 4.12899453479458 |
| C | 12.60459172258995 | -5.58716271809042 | 2.79079246064222 |
| C | 12.18120109099607 | -4.56513122640792 | 1.95714680000575 |

| | | | |
|---|---|---|---|
| C | 11.37878217843094 | -3.56458244293856 | 2.48124555194254 |
| H | 12.48618719297338 | -4.54454575475192 | 0.92238058088627 |
| H | 13.22913346084250 | -6.37557283473138 | 2.39643604103784 |
| H | 12.58841669330670 | -6.40175817211694 | 4.76721098235463 |
| H | 11.20654155512598 | -4.60161042462821 | 5.71361466361028 |
| C | 8.76081780427641 | -2.74725272955085 | 4.63387880718040 |
| C | 8.82315212104055 | -2.79391285183817 | 6.15618366641210 |
| H | 7.83395048884649 | -2.69444672301606 | 6.60379441111508 |
| H | 9.25480179321549 | -3.73773798733547 | 6.48905666271713 |
| C | 9.74289849491547 | -1.62630812363992 | 6.51060360468185 |
| H | 10.22596262752383 | -1.76194964972851 | 7.47773439964055 |
| H | 9.15946731898803 | -0.70675913003637 | 6.55350909128695 |
| C | 10.76699824430854 | -1.55193505682141 | 5.36730756602755 |
| H | 11.72052482140728 | -1.98559917510963 | 5.67030831617105 |
| H | 10.95383977239976 | -0.51973831952197 | 5.07134009743081 |
| H | 8.43755755550025 | -3.68875647723663 | 4.19065961473155 |
| H | 8.06672149030466 | -1.96885367684681 | 4.31689721625267 |
| H | 11.36535742440030 | -2.45990270532501 | -0.21111231564089 |
| H | 10.40473752015048 | -0.27087159482406 | -0.73359952054733 |
| O | 8.45275033822652 | 1.09947103348536 | -1.10765171736960 |
| O | 5.07643084382119 | 1.14475970273744 | 0.82890466505759 |
| C | 5.73144170414166 | 1.84957805091047 | 1.83780607478367 |
| C | 6.25465687216328 | 1.22739875889583 | 2.95411707741352 |
| C | 7.01704411894867 | 1.99894148101313 | 3.88523707128521 |
| C | 7.70998529793758 | 1.41787570187255 | 4.97034945344397 |
| C | 8.42701187773583 | 2.18788267212941 | 5.84550233583192 |
| C | 8.47077282595144 | 3.58174141126499 | 5.69952746523400 |
| C | 7.82052994784333 | 4.17562726178016 | 4.65505006341265 |
| C | 7.10637606146051 | 3.40281252177655 | 3.71447745897786 |
| C | 6.43123002386388 | 4.00365601421647 | 2.63850334477434 |
| C | 5.75034574833257 | 3.26632300405258 | 1.69896471679595 |
| C | 4.92724479939549 | 3.97844206742753 | 0.70665860280786 |
| C | 3.61897952497013 | 3.55554973952207 | 0.46939357739951 |
| C | 2.74412254024230 | 4.32085434101087 | -0.28143939628070 |
| C | 3.19031687018943 | 5.51032763218516 | -0.83345543036991 |
| C | 4.51302846209892 | 5.93116485934558 | -0.62469672839600 |
| C | 5.37376303518599 | 5.17911728035533 | 0.14298117341894 |
| H | 6.38651611813676 | 5.50959938547759 | 0.31845858825546 |
| C | 4.76037742348259 | 7.26686163613115 | -1.28611001996504 |
| C | 3.41989784020832 | 7.53835868009894 | -1.92686018941071 |
| C | 3.03288807154340 | 8.62031313556430 | -2.68947063681479 |
| C | 1.73187471571476 | 8.66978003719143 | -3.17296921951461 |
| C | 0.83433949943805 | 7.64923727332197 | -2.89893923510292 |
| C | 1.21611032125054 | 6.55676873012593 | -2.13654279808189 |
| C | 2.51229083557844 | 6.50602244740255 | -1.64792456764637 |
| H | 0.51505777341871 | 5.75931625865079 | -1.93869638478195 |
| H | -0.17357106587310 | 7.70425010181072 | -3.28532188145717 |
| H | 1.41504050723827 | 9.51210615294312 | -3.77061144974249 |
| H | 3.72478126144745 | 9.41962746280072 | -2.91177994079963 |
| C | 5.92375744412470 | 7.22536132706948 | -2.30457625047585 |

| | | | |
|---|---|---|---|
| H | 5.55478784027450 | 7.12473697822517 | -3.32478542349377 |
| H | 6.55314698498332 | 6.36097256653521 | -2.09068522851608 |
| C | 6.71610708509576 | 8.51295753416021 | -2.08753939521158 |
| H | 6.25224481772050 | 9.33934687274855 | -2.62787383994743 |
| H | 7.74862059586448 | 8.41779219561570 | -2.42409629357245 |
| C | 6.60050092474446 | 8.74454933186985 | -0.58430262765293 |
| H | 6.83335177017678 | 9.76926565690024 | -0.29656158657016 |
| H | 7.27643836266793 | 8.07461430506459 | -0.05324155753523 |
| C | 5.14988776521568 | 8.37652578903456 | -0.27295784356404 |
| H | 4.50329192860289 | 9.24262828841847 | -0.42179757410424 |
| H | 5.02084680684218 | 8.03517038096412 | 0.75402610339019 |
| H | 1.72396077716866 | 3.99345253418455 | -0.40132963818049 |
| H | 3.27001289133479 | 2.63058437949381 | 0.89687857986953 |
| H | 6.42734918440739 | 5.08182797025348 | 2.57561918555230 |
| H | 7.84516030764597 | 5.24719818858065 | 4.52463239132865 |
| H | 9.01930356057692 | 4.17616619979669 | 6.41467112684119 |
| H | 8.95857645701550 | 1.72491484936105 | 6.66397402351566 |
| H | 7.66608082370274 | 0.34874336409352 | 5.10924190115174 |
| C | 5.98112441018823 | -0.19853455323180 | 3.24361499856735 |
| C | 6.26064500450527 | -1.16899241472165 | 2.31014646255253 |
| C | 5.98728122145642 | -2.53630199006990 | 2.52139808123408 |
| C | 5.49489817137335 | -2.91282136921293 | 3.74780493734186 |
| C | 5.18451367179113 | -1.96649494412254 | 4.73998687252646 |
| C | 4.63471622531566 | -2.36549141184202 | 5.97761263811242 |
| C | 4.24217536214202 | -1.44050113959113 | 6.90308856991682 |
| C | 4.36544840679008 | -0.07324910296436 | 6.61659038493239 |
| C | 4.92045302668982 | 0.34408246695640 | 5.43815022142902 |
| C | 5.38150914409560 | -0.58471902824643 | 4.48060387824876 |
| H | 4.99847167213767 | 1.39990584503606 | 5.23326083770518 |
| H | 4.01667178308163 | 0.65450031599321 | 7.33379435291962 |
| H | 3.82208810326219 | -1.75174992754448 | 7.84787283557145 |
| H | 4.52246471389543 | -3.42243814212547 | 6.17099471091028 |
| H | 5.30160803459168 | -3.95683744076013 | 3.94447237767242 |
| C | 6.13213087458099 | -3.52796903095569 | 1.44450975429986 |

516

I-2 ($r^2$SCAN-3c)

| | | | |
|---|---|---|---|
| P | -7.15891455301539 | 0.71493015572830 | 1.44623828459811 |
| O | -7.47782001087818 | 2.30371417693213 | 1.39098102468959 |
| O | -8.39873096851447 | 0.09917892477832 | 2.29638428595311 |
| C | -8.30642184139250 | 2.94546401725982 | 2.29970957819809 |
| C | -7.75056901514977 | 4.02547959800716 | 3.03727384723227 |
| C | -8.60975174173629 | 4.72664851005181 | 3.86161746320442 |
| C | -9.95580366765746 | 4.35786099637457 | 4.04508672563425 |
| C | -10.79102539740187 | 5.06571819675369 | 4.94471481796488 |
| C | -12.08230423020724 | 4.65928816713249 | 5.17263499639020 |
| C | -12.58076929722747 | 3.51304751421121 | 4.51928035498901 |
| C | -11.80138503186003 | 2.81925643201763 | 3.62353345640148 |
| C | -10.47867661347501 | 3.23640986605845 | 3.33282549359903 |
| C | -9.63794939483799 | 2.57015504975701 | 2.38383204143620 |
| C | -10.20055386509544 | 1.45717464023806 | 1.57669851957311 |

| | | | |
|---|---|---|---|
| C | -11.35954697068845 | 1.62217270558178 | 0.75709614451534 |
| C | -11.93400287959241 | 2.88621778407733 | 0.48363849111095 |
| C | -13.03929298949660 | 2.99930307491426 | -0.32726400099685 |
| C | -13.63561318974264 | 1.85192565634607 | -0.89004905875225 |
| C | -13.09884010929644 | 0.61128647178726 | -0.64681786380146 |
| C | -11.94315969734134 | 0.46239502829835 | 0.15810995395231 |
| C | -11.39596255647055 | -0.81324364613042 | 0.41474906921303 |
| C | -10.25202943546683 | -0.98854101380847 | 1.16976808195319 |
| C | -9.64270787632488 | 0.19827341523415 | 1.66693623665921 |
| H | -8.23421291775785 | 5.59318359545720 | 4.39696926151412 |
| H | -10.38037410944115 | 5.92764208214805 | 5.46301167559376 |
| H | -12.71334134989032 | 5.20289362422320 | 5.86829517073403 |
| H | -13.58853970930359 | 3.16977155191015 | 4.73055609978158 |
| H | -12.19949884358338 | 1.93603051942444 | 3.13903169885314 |
| H | -11.49074801087622 | 3.77398488937895 | 0.92033760772698 |
| H | -13.45773039747577 | 3.97983432612824 | -0.53227426355980 |
| H | -14.51755818490726 | 1.95334873749596 | -1.51462899404264 |
| H | -13.54372629873285 | -0.27958382599201 | -1.07902863643449 |
| H | -11.91729185245401 | -1.68270340385526 | 0.02436356360957 |
| N | -7.23128255840724 | 0.29339083253879 | -0.06560749607172 |
| P | -6.44732943022706 | -0.63399290664863 | -1.06394176280011 |
| O | -6.89939461379067 | -0.27508950693546 | -2.57698571635883 |
| O | -7.04240203550914 | -2.12054526066613 | -0.83323720619504 |
| C | -8.25375645022688 | -0.42728858419874 | -2.86317957269091 |
| C | -9.04838396581709 | 0.73878069700153 | -2.98462887076669 |
| C | -10.39369873075648 | 0.55616273854513 | -3.22560812110503 |
| C | -10.96150299448662 | -0.72972851482385 | -3.34781740033703 |
| C | -12.34521165715651 | -0.89249026647843 | -3.60225693282588 |
| C | -12.90314502159369 | -2.14319333318278 | -3.70603815874224 |
| C | -12.09331312523607 | -3.28670918643437 | -3.55132553046294 |
| C | -10.74263767945131 | -3.16214160727278 | -3.32289314950805 |
| C | -10.13089737826904 | -1.88937098741521 | -3.23680668902218 |
| C | -8.72801431005883 | -1.71176943593191 | -3.01862430997880 |
| C | -7.79592054586944 | -2.86665578787853 | -2.95272091258415 |
| C | -7.68252948216164 | -3.76768959073789 | -4.05283476474760 |
| C | -8.28134758229945 | -3.52706374208788 | -5.31264517481921 |
| C | -8.12839557786189 | -4.42287858345503 | -6.34478468514881 |
| C | -7.37786994440012 | -5.60480381079720 | -6.16723724469804 |
| C | -6.77288364250808 | -5.85819869474519 | -4.96106864360365 |
| C | -6.89456007489637 | -4.94484393147902 | -3.88359805355784 |
| C | -6.27602804365324 | -5.19470334553545 | -2.64501756842444 |
| C | -6.29167306431908 | -4.29738952717921 | -1.58868987319344 |
| C | -7.01865572481135 | -3.09107947747936 | -1.82653724461213 |
| H | -11.03396054163601 | 1.42544411145716 | -3.34647384553314 |
| H | -12.95788891326504 | -0.00188991193463 | -3.70678604049446 |
| H | -13.96498290711110 | -2.25475088826937 | -3.90099061199609 |
| H | -12.53838635852634 | -4.27475513991532 | -3.61626228188247 |
| H | -10.13033932982557 | -4.04854480724253 | -3.21517597706594 |
| H | -8.85299929685402 | -2.61821220082083 | -5.46248912713547 |
| H | -8.58379213278009 | -4.21585832202117 | -7.30795401915189 |

| | | | |
|---|---|---|---|
| H | -7.27129306261153 | -6.30451012022501 | -6.98986412308672 |
| H | -6.17815393308392 | -6.75568785574554 | -4.81623468341092 |
| H | -5.78940394495491 | -6.15560541797745 | -2.52979358501289 |
| N | -5.79391757190765 | 0.46254591778206 | 2.25082618991863 |
| N | -4.83987855032041 | -0.73400029442939 | -1.06214981483706 |
| S | -5.52637157071324 | -0.40206388641828 | 3.55598082639013 |
| O | -6.45572005748842 | -1.51259229625109 | 3.82191183519028 |
| O | -4.07992187723540 | -0.68114065712277 | 3.66590154450618 |
| S | -3.97431954319174 | 0.58800678730414 | -1.34188001463759 |
| O | -4.76014678122951 | 1.83472027190835 | -1.39392820048995 |
| O | -2.72344433173623 | 0.56717738850470 | -0.56149483434826 |
| C | -3.36417044089766 | 0.36591531766735 | -3.13255150878783 |
| C | -5.82642114748219 | 0.81922695282503 | 4.97474375844382 |
| F | -2.38496021703222 | -0.54220529959636 | -3.19756186533616 |
| F | -4.35979824486932 | -0.00990515849334 | -3.94394594120338 |
| F | -2.88336724414359 | 1.54598426747078 | -3.56713909265647 |
| F | -7.07425503561983 | 1.31135731169439 | 4.91997112089936 |
| F | -4.95809250485827 | 1.83726491104209 | 4.90509162848857 |
| F | -5.66117107401058 | 0.19492286090048 | 6.15192470904788 |
| C | -7.45766243687022 | -8.26841115033974 | 3.24201150889559 |
| C | -7.85991073200183 | -8.94553645183358 | 2.08831458819808 |
| C | -8.60642234804424 | -8.29404130265226 | 1.10303449651065 |
| C | -8.93303100400846 | -6.95846499436199 | 1.28195339846527 |
| C | -8.53315451588437 | -6.28014161062982 | 2.44500420109807 |
| C | -7.79497372621241 | -6.93052219722916 | 3.43156608335155 |
| H | -6.86654314996163 | -8.78565937906283 | 3.99179232695219 |
| H | -7.58365059597365 | -9.98689349226582 | 1.95347064062439 |
| H | -8.91202158877477 | -8.82663341276439 | 0.20581835760463 |
| H | -7.46589435049812 | -6.40106296652327 | 4.32094183717772 |
| C | -9.70630675620745 | -6.03946116911053 | 0.35845194852692 |
| C | -9.55880334488692 | -4.70247681590539 | 1.07118837021004 |
| C | -9.93992134838825 | -3.43487909831488 | 0.66195301389853 |
| C | -9.76820069206626 | -2.33808067338811 | 1.53237069925478 |
| C | -9.22606041814336 | -2.56224083863756 | 2.80910638496751 |
| C | -8.80775986357552 | -3.82378704241683 | 3.20645882372456 |
| C | -8.95636294452423 | -4.89077298129871 | 2.33006990653271 |
| H | -10.35097688021686 | -3.27235863806258 | -0.33179931789763 |
| H | -9.12102469640584 | -1.73828264387072 | 3.50081453597784 |
| H | -8.35983789697535 | -3.96038270316870 | 4.18597062312403 |
| C | -9.22335516818391 | -6.05864338377746 | -1.12519628605243 |
| H | -9.01982100574266 | -5.03291186666230 | -1.44554166133329 |
| H | -8.29012288619695 | -6.61660406593358 | -1.23023169026399 |
| C | -11.21655706081666 | -6.46569486358456 | 0.27046333942381 |
| H | -11.82588988958720 | -5.94457539415842 | 1.01363245857800 |
| H | -11.28915327439066 | -7.54125905056117 | 0.46742777510903 |
| C | -10.37801657924120 | -6.64768919705953 | -1.94813088136334 |
| H | -10.33067638142733 | -7.74418005127642 | -1.94187798099022 |
| H | -10.35783826766085 | -6.32478985497973 | -2.99461280616032 |
| C | -11.61793378520408 | -6.18918695030893 | -1.17807000030886 |
| H | -12.53554287363767 | -6.71021471544126 | -1.47020104177342 |

| | | | |
|---|---|---|---|
| H | -11.77538620670596 | -5.11403630768843 | -1.32956259818064 |
| C | -7.35763113530597 | 2.42617319933241 | -3.71976529384995 |
| C | -8.47039469604718 | 2.09681944782807 | -2.92953949339028 |
| C | -9.09650282010772 | 3.09214819619748 | -2.16506623692857 |
| C | -8.62363360679933 | 4.39214585737334 | -2.22354811044467 |
| C | -7.53563082468041 | 4.71911716810055 | -3.04842520021845 |
| C | -6.88959669096820 | 3.73172647505196 | -3.78941916497287 |
| H | -6.87333855656667 | 1.65199801295331 | -4.30453382441990 |
| H | -9.93814469270989 | 2.82724748694964 | -1.52874884855317 |
| H | -6.03778298344689 | 3.97038789120375 | -4.41930742647821 |
| C | -9.13530119460018 | 5.61545213117424 | -1.49324904460364 |
| C | -8.22969062899288 | 6.70422265798628 | -2.04291687417050 |
| C | -8.20639340507011 | 8.06513492613868 | -1.77015959560062 |
| C | -7.26198858534437 | 8.87320989420123 | -2.41058561012255 |
| C | -6.35324001906910 | 8.32784063143974 | -3.31711663914147 |
| C | -6.36504746889069 | 6.96233373950777 | -3.59319560588265 |
| C | -7.29832530200773 | 6.15530469441553 | -2.94833007873030 |
| H | -8.90520192140624 | 8.51219963067636 | -1.07125510987759 |
| H | -7.23903049451768 | 9.93829120822786 | -2.20171221073670 |
| H | -5.62983285659934 | 8.97183756445500 | -3.80776846854358 |
| H | -5.65582644064923 | 6.53425456462685 | -4.29558934080181 |
| C | -10.64566412741569 | 5.88156033831915 | -1.70494340058635 |
| H | -10.85025622548024 | 6.32729324600667 | -2.68299516487134 |
| H | -11.16195684602118 | 4.91610930237960 | -1.66345972363194 |
| C | -9.04869594333697 | 5.48667761738062 | 0.04758253261806 |
| H | -8.03135711539525 | 5.65370027994980 | 0.41187406319260 |
| H | -9.32913097660005 | 4.46434139602143 | 0.31502846911203 |
| C | -11.08497030054405 | 6.76502067426238 | -0.52346423273394 |
| H | -12.11136505038044 | 6.53467593684577 | -0.22131406471573 |
| H | -11.07193259865149 | 7.82204388797316 | -0.80623152395873 |
| C | -10.06703597453103 | 6.49140087015630 | 0.61659501518762 |
| H | -10.55039347952794 | 6.08867235676400 | 1.51262798650201 |
| H | -9.56901788879961 | 7.41715416676445 | 0.92087457469556 |
| C | -3.46963222190332 | -6.92144627020709 | 5.53567749166483 |
| C | -2.77151184789295 | -8.02167385329298 | 5.03197191413229 |
| C | -2.73351884116216 | -8.27404904438939 | 3.65818447708721 |
| C | -3.39942528233073 | -7.41528583501189 | 2.79722269016117 |
| C | -4.10973257890924 | -6.31373618381562 | 3.30716716246426 |
| C | -4.14812245459653 | -6.05881852806974 | 4.67669626760892 |
| H | -3.48505998869727 | -6.73952797480070 | 6.60596917119136 |
| H | -2.25071252294831 | -8.68515302426247 | 5.71553419082325 |
| H | -2.18424852359516 | -9.13106322922882 | 3.27655250491982 |
| H | -4.69452205720621 | -5.20442165335523 | 5.06643095228125 |
| C | -3.50704276604264 | -7.48035122482620 | 1.28541415541198 |
| C | -4.37196541868221 | -6.26462964480815 | 0.99753910066184 |
| C | -4.81755052202776 | -5.78806941384594 | -0.21953248687013 |
| C | -5.68959975313280 | -4.67642469162978 | -0.28378363231322 |
| C | -6.04304810366953 | -4.05377230798070 | 0.93068282700574 |
| C | -5.55141922135001 | -4.49727045989028 | 2.15181154568380 |
| C | -4.72374055029625 | -5.60966217212071 | 2.19048470941442 |

| | | | |
|---|---|---|---|
| H | -4.49203600122930 | -6.28433045798848 | -1.12724461647629 |
| H | -6.74042373852901 | -3.23127907837257 | 0.94301048105209 |
| H | -5.85074231278746 | -3.98055241994708 | 3.05847195672100 |
| C | -2.11084209697917 | -7.45861319441382 | 0.57860892092942 |
| H | -1.83524383525770 | -6.45311166129668 | 0.25299689434642 |
| H | -1.34998898694417 | -7.79116897138745 | 1.29313558177776 |
| C | -4.17133229814820 | -8.80915556858985 | 0.77121824253450 |
| H | -4.96231830652957 | -8.55265353518229 | 0.05886746557630 |
| H | -4.63617859758756 | -9.36522626732624 | 1.58931970828661 |
| C | -2.21062276728864 | -8.47206958042393 | -0.56441033955658 |
| H | -1.22969953461353 | -8.80997039233351 | -0.91282084961039 |
| H | -2.73795593474574 | -8.03365121505618 | -1.42157206976519 |
| C | -3.05941958960319 | -9.58421852898173 | 0.05404081809641 |
| H | -2.45581735970148 | -10.14641645493832 | 0.77806189874424 |
| H | -3.45218624450246 | -10.29640867765728 | -0.67823190945894 |
| C | -5.71450363617861 | 4.77270589671575 | 4.21220202843975 |
| C | -6.32953596214957 | 4.44446867147096 | 2.98956444024160 |
| C | -5.61188588029417 | 4.63024314309976 | 1.79448877320073 |
| C | -4.33885770267665 | 5.17909562542104 | 1.84396961950869 |
| C | -3.74842947855132 | 5.51876026210947 | 3.07471109849029 |
| C | -4.43201518501309 | 5.29957519122871 | 4.26712548465697 |
| H | -6.25433016547867 | 4.58667794003836 | 5.13496038104806 |
| H | -6.06123961961870 | 4.37088148115506 | 0.84072681211471 |
| H | -3.97775448497006 | 5.53260266753961 | 5.22584990046312 |
| C | -3.41409328503741 | 5.52747890925756 | 0.69314746208421 |
| C | -2.18958992418711 | 6.03219821665312 | 1.43249048465815 |
| C | -0.97347825431966 | 6.46022002092059 | 0.92125025760298 |
| C | 0.01149330691065 | 6.92625204181537 | 1.79467302204666 |
| C | -0.22164908684627 | 6.96374373064926 | 3.17039015644824 |
| C | -1.43640747329113 | 6.52581017858393 | 3.69420220763909 |
| C | -2.41498989545236 | 6.05450738028169 | 2.82070165112212 |
| H | -0.78158045705083 | 6.43169554666098 | -0.14583090128173 |
| H | 0.96713220486210 | 7.25744553814826 | 1.39781311214580 |
| H | 0.55318805105394 | 7.32807251574545 | 3.83773596624468 |
| H | -1.61189475970180 | 6.54768991450030 | 4.76602181423455 |
| C | -3.10126785922335 | 4.34931355493885 | -0.28916155502364 |
| H | -2.02887586092515 | 4.13068447268654 | -0.25895413130294 |
| H | -3.62459090740462 | 3.43966303011811 | 0.00395868168818 |
| C | -4.01151472520697 | 6.63980588207173 | -0.23114926647795 |
| H | -3.80875755299038 | 7.64252408676687 | 0.15641237365777 |
| H | -5.09995575415478 | 6.50902770506410 | -0.28382767785598 |
| C | -3.51821715677495 | 4.83020171407334 | -1.69055969812285 |
| H | -4.55987464073328 | 4.54642278547482 | -1.88299879184501 |
| H | -2.90547335582488 | 4.38673648144022 | -2.48138649984941 |
| C | -3.41279160336971 | 6.35387122833375 | -1.60797241575850 |
| H | -3.95110679821290 | 6.87015885124790 | -2.40680428852822 |
| H | -2.36378957992853 | 6.67251970251167 | -1.64982185953078 |
| H | -0.52645855881432 | -4.19810847521753 | -1.57877471425155 |
| H | -3.85599168612535 | -4.05380786723987 | -3.28295410367098 |
| H | -4.16747357025236 | -3.36683351866847 | -5.63280237993030 |

| | | | |
|---|---|---|---|
| C | -3.00253556782578 | -3.70566568498979 | -3.86067496846900 |
| C | -3.17932180374017 | -3.31544897220386 | -5.18365954983432 |
| C | -1.58533420409736 | -4.02257814148288 | -1.80729045859279 |
| C | -1.74279906943666 | -3.64522437915316 | -3.25533837939837 |
| C | -2.09229457304344 | -2.85830680467001 | -5.92788439977596 |
| H | -2.22725299961075 | -2.55225007258324 | -6.96087407928513 |
| C | -0.66149577051643 | -3.18804535327557 | -4.01119772768288 |
| C | -0.83249163752777 | -2.79823278313451 | -5.33792801314398 |
| H | -2.11641970754770 | -4.96636721188879 | -1.62096674262756 |
| H | -3.18761731696054 | -2.74145420998873 | -1.12442959058178 |
| C | -2.14787038559618 | -2.95713982732265 | -0.85526774255170 |
| H | -1.59412743425707 | -2.01988867732676 | -0.98092719165001 |
| H | 0.32333280611883 | -3.13240004643910 | -3.55367120314877 |
| H | 0.01944041452185 | -2.43995888152055 | -5.90854704088479 |
| C | -2.08849416375407 | -3.43902072509803 | 0.58856245121088 |
| H | -2.64238547567497 | -4.37961130489855 | 0.66558347847363 |
| H | -2.85510546874293 | -3.03647768656647 | 2.56257300149424 |
| H | -1.05520445751892 | -3.64860045422588 | 0.87914755749858 |
| H | 1.88141973631753 | -4.02191886098820 | 2.69108922176943 |
| C | -2.74236771830446 | -2.49494077980777 | 1.61581950332147 |
| H | 1.15639117691749 | -6.26911755451564 | 5.61678801559455 |
| H | -3.74069049526030 | -2.21653107949350 | 1.27033560017332 |
| H | 2.68457520683943 | -3.94231681040845 | 4.26244223349440 |
| C | 1.95683729263877 | -4.50746792946570 | 3.66962808567760 |
| H | -0.20760677959392 | -5.34943548580583 | 6.26760426626531 |
| C | 0.75193540709818 | -5.25617579229508 | 5.74523666472162 |
| H | -1.64705333554772 | -2.71075177349205 | 6.58796570071529 |
| H | 2.35746324338225 | -5.51989953684283 | 3.51988408230752 |
| C | -2.01427246227593 | -1.23573620739704 | 1.89373235191164 |
| C | 0.59014469001517 | -4.59050290519234 | 4.36585976610080 |
| H | -2.27175282975013 | -3.81946613933806 | 5.34966861192918 |
| C | -1.75944128445708 | -2.86429443658257 | 5.50950308637290 |
| H | 1.44469236760032 | -4.70391235570847 | 6.39097594088750 |
| Si | -0.09678608183496 | -2.85654377970892 | 4.66052535697761 |
| H | -2.33265890350228 | -0.27843415707984 | 1.49751375963507 |
| O | -0.36445254572000 | -2.35491432943782 | 3.00722187620775 |
| N | -0.96132131481647 | -1.21114290361848 | 2.63745240168599 |
| H | -1.36479027041977 | -5.51560654271761 | 3.96239942095112 |
| C | -0.36936300311682 | -5.43260782999634 | 3.51355749755493 |
| H | -2.41384779197885 | -2.07466260063358 | 5.12542077564148 |
| H | 2.68383714960591 | 2.43035590702052 | 4.21794912151683 |
| H | 0.02689454719603 | -6.45211034534261 | 3.41437514470037 |
| H | 1.77979534113561 | 1.19732851555869 | 5.10979573529250 |
| O | -0.26266519392536 | -0.09987273097955 | 2.96628166387048 |
| H | -0.48290683272286 | -5.02036516813670 | 2.50700411900237 |
| C | 1.85224324312858 | 1.71458898696659 | 4.14693888882192 |
| H | -2.16086972350239 | 1.82515840926804 | 5.74820796212376 |
| C | 1.16428911824952 | -1.73285162044095 | 5.43976944341039 |
| C | -1.66643445640206 | 0.92403244947849 | 5.36563805777858 |
| Si | -0.96163331054724 | 1.34901210152851 | 3.69202277853395 |

| | | | |
|---|---|---|---|
| H | -0.90810100883989 | 0.62476201284054 | 6.09402093805662 |
| H | 2.10763760904450 | 0.97766125400781 | 3.38006742497331 |
| H | 1.97571970950237 | -1.51986786178865 | 4.73621896382386 |
| H | 1.59753723826572 | -2.21135389148750 | 6.32491966011796 |
| H | 0.72660067654595 | -0.78418728354558 | 5.75572822805622 |
| C | 0.56135852799271 | 2.47129967363656 | 3.79911569239197 |
| H | 0.21508748642420 | 3.03921709238821 | 5.89281959489246 |
| C | 0.26607206617127 | 3.50492904681313 | 4.90305395009978 |
| H | -2.43254065417640 | 0.14833481316938 | 5.29409234777331 |
| H | 1.06835114242445 | 4.25449209170544 | 4.92017867792695 |
| C | -2.26658953669097 | 2.06933379528657 | 2.58130082562427 |
| C | 0.75404276384118 | 3.20870009374768 | 2.46538960944554 |
| H | 1.60137871936244 | 3.90088582440313 | 2.55178652683986 |
| H | -2.47913748594157 | 3.08982745545310 | 2.92556330456330 |
| H | -3.20774966886916 | 1.51435689608062 | 2.61948485618066 |
| H | -0.67265500888921 | 4.04250211016501 | 4.72423726087275 |
| H | -0.12348552156928 | 3.80252072983853 | 2.18718623218413 |
| H | -1.93701231156591 | 2.13598847089300 | 1.54020675994157 |
| H | 0.97553746348346 | 2.50725652050638 | 1.65414882745664 |
| H | 4.27812017461837 | -6.11930338339578 | 0.10700379908462 |
| O | 6.77325846626884 | -0.84336628914774 | 1.20052759237773 |
| H | 4.21286789806255 | -4.43417108007631 | 1.91480347297108 |
| C | 5.12906729122693 | -5.46513313680622 | 0.27231981075006 |
| C | 7.45051674904036 | -7.49387790045670 | -4.06544539582648 |
| C | 7.30015427346112 | -3.71161547651868 | 0.68071806722131 |
| C | 6.25091945410404 | -5.51996396063339 | -0.54857940447044 |
| C | 7.33729653424123 | -4.65361059850436 | -0.33520953601467 |
| H | 10.92015124670889 | -6.04118322708328 | -2.45183279211252 |
| C | 5.73130778907564 | -7.27174656547551 | -2.37855021559876 |
| C | 6.20634639145709 | -7.85733997652949 | -3.54954269998767 |
| H | 9.97722539577754 | -4.71655920864794 | 0.11952446758306 |
| C | 6.52382902448226 | -6.32550857632691 | -1.73236135201354 |
| H | 10.21365135297812 | -4.24144798043621 | -3.75820007014817 |
| H | 4.75658385333340 | -7.54171666558084 | -1.98295893935429 |
| C | 5.09717286929668 | -4.52608369687537 | 1.29365719475559 |
| H | 10.91290185839715 | -3.08185090317360 | -2.64849593780511 |
| H | 7.80431955360869 | -7.94908059784367 | -4.98535768427446 |
| H | 5.60214745913428 | -8.59458965487451 | -4.06900767336476 |
| H | 9.72346822510568 | -6.39531614003533 | -0.38085491273835 |
| H | 11.79674115997286 | -4.92319876756469 | -1.42512416789866 |
| H | 8.13578723175556 | -3.03582279806965 | 0.84377494030974 |
| C | 8.42464867531992 | -4.89849602552759 | -1.36173287862561 |
| C | 7.78517447764875 | -5.95973046614796 | -2.24375239323058 |
| C | 10.81008273369288 | -5.13733445972102 | -1.84479638186480 |
| C | 9.76673041184202 | -5.36262820727882 | -0.73990668835701 |
| H | 9.19992462289024 | -6.26634672539834 | -3.84889631574353 |
| H | 8.15310765975555 | -3.37118733141442 | -2.92095575414732 |
| C | 8.24467454678224 | -6.54351421097188 | -3.41790949574286 |
| C | 8.86846425271909 | -3.63985638906910 | -2.14309602710885 |
| H | 8.92812954485243 | -2.80143850575530 | -1.43992175627522 |

| | | | |
|---|---|---|---|
| C | 10.26828557734807 | -3.96294627412473 | -2.70136194975000 |
| P | 5.65833329595971 | -0.09249373570688 | 0.29801103369145 |
| N | 4.36344728159866 | -0.96564659548849 | -0.00176688509547 |
| S | 2.97691552935085 | -0.89945018919938 | 0.79891856914345 |
| O | 2.26959851142088 | 0.38835777725663 | 0.67988558929711 |
| O | 2.98718748434283 | -1.55322713787012 | 2.12116493932043 |
| C | 2.00528827933763 | -2.07908794734440 | -0.31381375640515 |
| F | 2.00307152288288 | -1.66891291994557 | -1.59041969874255 |
| F | 2.50213333200815 | -3.32270542960488 | -0.26346502708667 |
| F | 0.73967471186301 | -2.10534623934993 | 0.12846048271445 |
| N | 6.41856789456278 | 0.24489286133934 | -1.04183233443097 |
| P | 7.35492852443418 | 1.17109543936269 | -1.85491774123821 |
| N | 7.37673598193262 | 0.80595615357548 | -3.44612984554449 |
| S | 8.09310447995763 | -0.35333828705924 | -4.16362402248214 |
| O | 9.37781629205036 | -0.87726709462867 | -3.70710650500256 |
| O | 7.13673840523906 | -1.53920472120585 | -4.53551129320509 |
| Si | 5.42730017335948 | -1.94099932817129 | -4.28980063672642 |
| C | 4.46164612710591 | -0.36396643440870 | -4.20986012947993 |
| H | 4.76017195883411 | 0.34286149353003 | -4.98822477200659 |
| H | 4.57325788471632 | 0.12789290301513 | -3.24020972234494 |
| H | 3.39682653183955 | -0.58342613484847 | -4.35269911216025 |
| C | 5.34846976983792 | -2.93919047886095 | -2.73073528742184 |
| H | 5.76354252623194 | -3.94225565048180 | -2.86012092379042 |
| H | 5.87930723989086 | -2.44554484979502 | -1.91217628239710 |
| H | 4.30331188390212 | -3.03993263843733 | -2.41903046216893 |
| C | 5.05871602560985 | -2.96884526194100 | -5.82911590605717 |
| C | 5.16897836519461 | -2.11171003382165 | -7.09876959774552 |
| H | 6.19510903122073 | -1.76670307022590 | -7.26361738264526 |
| H | 4.51328118158643 | -1.23413698396963 | -7.05800145562867 |
| H | 4.87506349895190 | -2.70670974199459 | -7.97366410338517 |
| C | 6.01781420979613 | -4.16603083469734 | -5.92758804362058 |
| H | 7.06179873557803 | -3.84480874869627 | -6.01157440193635 |
| H | 5.93606290127413 | -4.83059584316465 | -5.06044491365328 |
| H | 5.77738521577143 | -4.75916795083184 | -6.82006050661039 |
| C | 3.61324116991349 | -3.48556371270543 | -5.68465122868567 |
| H | 3.48660362745370 | -4.10078363325617 | -4.78652265333403 |
| H | 2.88590748881856 | -2.66627351119451 | -5.64597226022690 |
| H | 3.36001133003313 | -4.11021017134736 | -6.55130649410152 |
| C | 8.35406241531855 | 0.35761451561488 | -5.90153068105901 |
| F | 9.08960924444465 | 1.46365664345940 | -5.80572511762885 |
| F | 7.18420799811781 | 0.64012266019364 | -6.47138108978519 |
| F | 9.00025491506586 | -0.55174072858369 | -6.63172458231430 |
| O | 6.85468279384759 | 2.69181751102653 | -1.75580061566184 |
| C | 7.62392191006557 | 3.64473875395406 | -2.44531795936422 |
| C | 8.84875933430974 | 3.99488854443179 | -1.91109485900496 |
| C | 9.68326579665186 | 4.87855159824749 | -2.66859450186379 |
| C | 11.02519443724619 | 5.16299492009145 | -2.31699233172859 |
| C | 11.77976772596655 | 6.03707896823246 | -3.06326162598366 |
| C | 11.23278660699025 | 6.67406951400888 | -4.19670007571054 |
| C | 9.94943618958902 | 6.38662429753587 | -4.58972810151923 |

| | | | |
|---|---|---|---|
| C | 9.15489915536173 | 5.46812411885705 | -3.85876792638594 |
| C | 7.87129570547715 | 5.10141897971266 | -4.30999274148545 |
| C | 7.09880398553020 | 4.16134808141025 | -3.65652128236514 |
| C | 5.84103765145711 | 3.67774377405663 | -4.26374943305939 |
| C | 5.85434179369681 | 3.39797725615672 | -5.64108277042231 |
| C | 4.73822068688219 | 2.88733105206669 | -6.28933020331828 |
| C | 3.58690191673155 | 2.64666506280374 | -5.54641353586268 |
| C | 3.54513064977134 | 2.95622184491485 | -4.17452090697099 |
| C | 4.65861583936529 | 3.47148430573232 | -3.53109035228935 |
| H | 4.62526525356350 | 3.70805946302950 | -2.47163151377823 |
| C | 2.17937659651460 | 2.63869473592023 | -3.60333154079913 |
| C | 1.50871065950616 | 1.94640045836114 | -4.77574480165825 |
| C | 0.28317807069012 | 1.29842836938524 | -4.84320349809826 |
| C | -0.12499937648209 | 0.73605230745655 | -6.05521862138218 |
| C | 0.68646900893951 | 0.81195902461706 | -7.18810006370456 |
| C | 1.92630296040431 | 1.44518191283148 | -7.12626257401575 |
| C | 2.33065676995616 | 2.00844049870813 | -5.91790529681230 |
| H | 2.56676811861942 | 1.48896330039425 | -8.00260840132282 |
| H | 0.35313026449719 | 0.36607705733274 | -8.12021518269666 |
| H | -1.08098792569975 | 0.22413430601703 | -6.10868521456708 |
| H | -0.35486929514627 | 1.20739131354377 | -3.97283906848001 |
| C | 2.14487426612299 | 1.83358409957517 | -2.28524008282488 |
| H | 2.29804756925358 | 0.76636347014351 | -2.45293884241199 |
| H | 2.95784594972721 | 2.18246008079227 | -1.63830365047009 |
| C | 0.77797954708572 | 2.14914686299476 | -1.63719008973842 |
| H | 0.88689656891447 | 2.26061142724878 | -0.55675530728540 |
| H | 0.06871633636281 | 1.32903645473993 | -1.78706520306982 |
| C | 0.27139254969076 | 3.45139511127351 | -2.30709848788924 |
| H | -0.01302093096381 | 4.21079338451110 | -1.57058642474207 |
| H | -0.61907029416422 | 3.25718893755126 | -2.91287618500989 |
| C | 1.42606226178064 | 3.93305489970262 | -3.19343931785254 |
| H | 1.10504342926465 | 4.51997784904234 | -4.05966712261893 |
| H | 2.12795245677611 | 4.54331733687389 | -2.61602427233644 |
| H | 4.78409267570064 | 2.64694392528201 | -7.34752722986986 |
| H | 6.77636571620828 | 3.53849662627354 | -6.19586977095833 |
| H | 7.48895476613352 | 5.56084648350438 | -5.21647590518568 |
| H | 9.52660063062021 | 6.84354941882701 | -5.47980878038461 |
| H | 11.83610762678425 | 7.37261837804703 | -4.76725041834309 |
| H | 12.80865616371459 | 6.23442477832349 | -2.78007307684786 |
| H | 11.46280041757792 | 4.67696517719895 | -1.45323883829768 |
| C | 9.30152816214778 | 3.44004025517975 | -0.61230935249926 |
| C | 9.35316901689321 | 2.07146459473591 | -0.40245905524701 |
| C | 9.90734678352304 | 1.47200284059623 | 0.75763733961822 |
| C | 10.35368542684579 | 2.32613396321991 | 1.74779782382548 |
| C | 10.28283021154116 | 3.72547666670958 | 1.62779139477380 |
| C | 10.78551702176420 | 4.56507554490113 | 2.65146105483577 |
| C | 10.72425361518990 | 5.93053753628822 | 2.53376705011517 |
| C | 10.13602678676576 | 6.50673607258923 | 1.38984916679789 |
| C | 9.64973757872072 | 5.71659827335316 | 0.37419518191262 |
| C | 9.73531930563620 | 4.30515753054591 | 0.44384717795114 |

| | | | |
|---|---|---|---|
| H | 9.20594431923934 | 6.17939181051023 | -0.49832279381948 |
| H | 10.07091623627200 | 7.58686465720443 | 1.30736152147733 |
| H | 11.11677604093501 | 6.56782531623064 | 3.31961711061800 |
| H | 11.21330059581172 | 4.10131264945156 | 3.53470072440797 |
| H | 10.81970947213139 | 1.90650947403275 | 2.63429466707320 |
| C | 10.16910371931884 | 0.02553660401027 | 0.92647708061210 |
| C | 10.82062100295729 | -0.71635733717596 | -0.07202532515991 |
| C | 11.35250725450692 | -1.97238311027328 | 0.20534728757246 |
| C | 11.21781592021267 | -2.50136695568910 | 1.48751090396596 |
| C | 10.49815191018223 | -1.79964484287742 | 2.46898953496168 |
| C | 9.97668920502400 | -0.54979286492001 | 2.19379859064487 |
| H | 9.44612598418316 | 0.01322917842352 | 2.95689502010661 |
| C | 10.46590417334703 | -2.56747713762217 | 3.77246727344215 |
| C | 11.31613357766696 | -3.78025303473992 | 3.43368509148257 |
| C | 11.70519905765594 | -4.84641608565634 | 4.23424433689257 |
| C | 12.51112838641071 | -5.85037061538422 | 3.69096534987190 |
| C | 12.93408333430556 | -5.78606913505486 | 2.36318922720763 |
| C | 12.55389368871528 | -4.71898594753519 | 1.55373240072775 |
| C | 11.73979144303875 | -3.72404963308494 | 2.08987209758630 |
| H | 12.89723552338991 | -4.66102106630610 | 0.52581957529877 |
| H | 13.56815078019321 | -6.56997211979053 | 1.96105964313669 |
| H | 12.81720161964710 | -6.68596171443482 | 4.31264478471587 |
| H | 11.39998981987512 | -4.91167209221381 | 5.27278642562043 |
| C | 9.03502025474732 | -2.93948772857631 | 4.22935999368467 |
| C | 9.14527396177695 | -3.24361469663907 | 5.73155822628032 |
| H | 8.22611588289719 | -2.95967420057336 | 6.25342969761261 |
| H | 9.28571375296631 | -4.31584207762476 | 5.89794348926032 |
| C | 10.37801313395147 | -2.44455773849518 | 6.23449284164531 |
| H | 11.10680425996566 | -3.11132688510355 | 6.70509692348022 |
| H | 10.10111130467093 | -1.70158738636550 | 6.98867562114479 |
| C | 10.98173216669217 | -1.76095080491779 | 4.99036349160012 |
| H | 12.07411803476336 | -1.70661302032794 | 5.01086750071985 |
| H | 10.59906695342032 | -0.73787132794316 | 4.90192341642968 |
| H | 8.61838401569976 | -3.76312546771256 | 3.64302509967382 |
| H | 8.39564289654428 | -2.06598818923092 | 4.06879589645123 |
| H | 11.89809662165675 | -2.51069535839503 | -0.56368254036781 |
| H | 10.96971897305715 | -0.27988600941922 | -1.05370165609667 |
| O | 8.91155710815532 | 1.21739002353552 | -1.42082301475532 |
| O | 5.18544503293063 | 1.16204086122902 | 1.20940274316174 |
| C | 5.98240289761025 | 1.80600738538886 | 2.15016306959320 |
| C | 6.51865504226497 | 1.08041619087730 | 3.20089371582258 |
| C | 7.41923999391890 | 1.74613278363023 | 4.09185815463471 |
| C | 8.15142832132722 | 1.06191200369604 | 5.09267657693594 |
| C | 9.00973330095224 | 1.73874761755190 | 5.92760003252800 |
| C | 9.16359262783743 | 3.13621363542986 | 5.81884317994240 |
| C | 8.47980999590138 | 3.82573142851487 | 4.84785496311517 |
| C | 7.61723427299745 | 3.15260381695980 | 3.94876615553059 |
| C | 6.92941705348978 | 3.85242040069403 | 2.93708215668161 |
| C | 6.10835835017299 | 3.21901851384319 | 2.02347516890217 |
| C | 5.31018552192213 | 4.02895386726559 | 1.07395272650413 |

| | | | |
|---|---|---|---|
| C | 3.95597922424801 | 3.73436052962090 | 0.84120907432617 |
| C | 3.14685883481994 | 4.59581707653288 | 0.10839415650225 |
| C | 3.69275552699639 | 5.76618753934936 | -0.41050237246736 |
| C | 5.05205483150506 | 6.06656575649654 | -0.20237732998736 |
| C | 5.85599059001808 | 5.20570976022849 | 0.52444279769665 |
| H | 6.90762475495677 | 5.43089357594105 | 0.68167591057409 |
| C | 5.40391006853772 | 7.40835976945671 | -0.82273087454653 |
| C | 4.09242708402842 | 7.79052518972122 | -1.47594188616492 |
| C | 3.79762905678532 | 8.91638316351213 | -2.22974332152937 |
| C | 2.49539411354092 | 9.09396973945271 | -2.70211357240116 |
| C | 1.49963629687319 | 8.15918315121360 | -2.41057770242116 |
| C | 1.79272772203775 | 7.02652038314364 | -1.65605513650049 |
| C | 3.09511642133161 | 6.84117615489530 | -1.19659083455645 |
| H | 1.02025043132729 | 6.29717206615403 | -1.43818747213278 |
| H | 0.49008271579536 | 8.31486469014807 | -2.77791286832517 |
| H | 2.25286401174011 | 9.96889171317065 | -3.29710492917843 |
| H | 4.56418874485878 | 9.65439601648859 | -2.45213748130637 |
| C | 6.62612323512990 | 7.34615359377495 | -1.79640821065055 |
| H | 6.30917433352223 | 7.38492967126525 | -2.84180990622298 |
| H | 7.14176251021997 | 6.39176740933189 | -1.65171690476835 |
| C | 7.55283620958225 | 8.50182602585412 | -1.39533560978895 |
| H | 7.23395143705788 | 9.43422655992676 | -1.87784853104887 |
| H | 8.59463346930935 | 8.31926886218132 | -1.68189460135855 |
| C | 7.33814438502046 | 8.61008845035067 | 0.11623393249233 |
| H | 7.71809055864741 | 9.54202885466433 | 0.54726772291741 |
| H | 7.83261681968364 | 7.77682163459716 | 0.62660938369870 |
| C | 5.82175773402973 | 8.47443156722905 | 0.25104159650720 |
| H | 5.34756241299292 | 9.42890769811297 | -0.00097943424888 |
| H | 5.48745522200899 | 8.18906650094732 | 1.25215968903465 |
| H | 2.09598698044526 | 4.35778230092711 | -0.03233175478446 |
| H | 3.51584321465836 | 2.84004084614708 | 1.26517080979905 |
| H | 7.02811911900909 | 4.93331247829056 | 2.90079716765572 |
| H | 8.60251788135280 | 4.89895924150313 | 4.73947717428165 |
| H | 9.82887569998740 | 3.66106613322601 | 6.49702690176466 |
| H | 9.56927477592690 | 1.19195532887774 | 6.68031679643803 |
| H | 8.03729813218539 | -0.01160625293739 | 5.19440632641730 |
| C | 6.14890449584811 | -0.33658035696676 | 3.43398201048495 |
| C | 6.31636059016528 | -1.28424165694087 | 2.44633225275168 |
| C | 6.03540861499793 | -2.66216947693287 | 2.60563069788801 |
| C | 5.58350824962357 | -3.06437242292015 | 3.84456167567589 |
| C | 5.33690166619992 | -2.14834854868735 | 4.88686026547308 |
| C | 4.82551218069546 | -2.58363221655866 | 6.13474032806458 |
| C | 4.51449704630538 | -1.68095539495303 | 7.12128646104308 |
| C | 4.68261650577192 | -0.30028443093860 | 6.88876576491803 |
| C | 5.20413573746887 | 0.15236749321508 | 5.69939067064164 |
| C | 5.57821193807455 | -0.75433637030426 | 4.67852798518658 |
| H | 5.32241349704934 | 1.21681008675809 | 5.53355367441558 |
| H | 4.39407955441712 | 0.41298241859864 | 7.65426273705996 |
| H | 4.11663822572343 | -2.02393836978076 | 8.07107334618727 |
| H | 4.66911363021156 | -3.64807923152029 | 6.28693777596403 |

H 5.39544792844309 -4.11926880252743 4.02192022928193
C 6.16424249883669 -3.63785969735793 1.50482962748236
315
RC-1 (r2SCAN/3c/xTB)
P 1.71608061208169 1.37002400766993 1.47169159698413
O 3.29220205404283 1.45622211139427 1.35726859759835
O 1.37083052619290 2.33170824373935 2.74221015599721
C 4.04501956688337 1.85303563806953 2.46170841712788
C 4.90932590683601 0.92123124543444 3.08262215846550
C 5.74955384642560 1.37070728473896 4.07492106727376
C 5.72271549352486 2.69886973520299 4.53462203255856
C 6.58227114473725 3.13882527367029 5.56490683825622
C 6.48378980867486 4.40654118013757 6.06444840600876
C 5.50118607673294 5.27753506425118 5.56950596872307
C 4.66583633420259 4.88578405826948 4.55951100424427
C 4.77474596763898 3.60029375413755 3.98411346942591
C 3.95901606765659 3.15767974354019 2.90265512949819
C 2.94325126214132 4.06528646446667 2.32872626223477
C 3.28055261895804 5.33374401637731 1.78429195751064
C 4.61395576311889 5.73773815219705 1.55414614810940
C 4.89008685301098 6.96121286711313 1.00758347727755
C 3.85088251524406 7.84779939095535 0.68484747790319
C 2.54980309447674 7.48037793626114 0.88524634965883
C 2.22930140581647 6.21005721948874 1.41148785460623
C 0.89595216214584 5.80520650093408 1.59954951524637
C 0.56108088565828 4.55141895284740 2.06368894167217
C 1.62372571276610 3.66996875429273 2.39218622493797
H 6.44251747343543 0.67828962143472 4.53161255257456
H 7.31275624623809 2.44534682088349 5.95587732483082
H 7.14379350876908 4.73885748713522 6.85179461586128
H 5.40368526937599 6.26495094884903 5.99589383996092
H 3.90607614830845 5.56288469922255 4.20156467513458
H 5.41822655739066 5.06528408751175 1.80975819191099
H 5.91379352831581 7.25576686099681 0.82741425737121
H 4.08953074234971 8.81910759838869 0.27683333445585
H 1.74088550071817 8.15195883775330 0.63752040844251
H 0.11430437015698 6.51485178044807 1.36967882361234
N 1.05313820035445 2.11267730648089 0.23049620467309
P 0.02363223129143 1.46794475665466 -0.79160626028084
O 0.55518318393759 1.88054977946959 -2.26344533682598
O -1.37420180977788 2.20261542613206 -0.63958248806242
C 0.75322026185419 3.26970094462035 -2.30612913270828
C 2.04817811843277 3.82109450771970 -2.16417056005286
C 2.15650090916562 5.19156216224956 -2.09348857962030
C 1.03332409192311 6.03624801453902 -2.15561832150303
C 1.16183581711290 7.44014941676732 -2.07946673093129
C 0.06159746650909 8.24938416944978 -2.13222979657204
C -1.21718947960909 7.68754760671490 -2.26657645645158
C -1.37228717116325 6.33193693643772 -2.36844103084625
C -0.25603214200362 5.46949552711284 -2.32984775051222

| | | | |
|---|---|---|---|
| C | -0.36857302135301 | 4.05421809267795 | -2.42152541079316 |
| C | -1.67964864829858 | 3.40775544785879 | -2.65628311053609 |
| C | -2.38471330006558 | 3.69514428733543 | -3.85583523997951 |
| C | -1.83688135024904 | 4.45128243755314 | -4.91306908315642 |
| C | -2.55263365054655 | 4.66493931731219 | -6.05908289110036 |
| C | -3.84607958901411 | 4.14025314710632 | -6.20246650985009 |
| C | -4.39824695546238 | 3.39728782040836 | -5.19794730556991 |
| C | -3.68095250586882 | 3.15066822992165 | -4.00790913950226 |
| C | -4.23190261963763 | 2.40259654087794 | -2.95619697278761 |
| C | -3.54363809424840 | 2.05837162430389 | -1.80817969832665 |
| C | -2.19532317059825 | 2.52993674066937 | -1.72426303171166 |
| H | 3.13440480989603 | 5.64247573253910 | -2.00149613198273 |
| H | 2.15237261810067 | 7.85869037172966 | -1.97816503665125 |
| H | 0.16713351096263 | 9.32266514768639 | -2.07193951520250 |
| H | -2.08181952113121 | 8.33440090826563 | -2.29724079855204 |
| H | -2.35737243385308 | 5.90789960839642 | -2.48353399924135 |
| H | -0.83576586285608 | 4.84399326742958 | -4.81896639524724 |
| H | -2.11999651558888 | 5.23772337585873 | -6.86608498073267 |
| H | -4.39487167255843 | 4.32135273878370 | -7.11450896595125 |
| H | -5.38697614513022 | 2.97556281661036 | -5.30389967141130 |
| H | -5.25964071312541 | 2.10538542642681 | -3.08318993647128 |
| N | 1.33245981787735 | -0.13060901168390 | 1.95398318813388 |
| N | -0.20905023151314 | -0.13235622912775 | -0.84798739561999 |
| S | -0.18320677581549 | -0.31195435395241 | 2.21148793852650 |
| O | -1.04122160937732 | 0.73391941795414 | 1.74031864544055 |
| O | -0.67025255630336 | -1.66567723371191 | 2.08372284182227 |
| S | 1.11796568857381 | -0.93761674551855 | -0.92239413521595 |
| O | 2.33530989159786 | -0.23488548878210 | -0.64163224717469 |
| O | 0.96069606549836 | -2.28247642084273 | -0.43161573722493 |
| C | 1.28708291452395 | -1.33719114514173 | -2.79592463495055 |
| C | -0.31392903449035 | -0.19084819362981 | 4.12887910541117 |
| F | 0.25825760395130 | -2.09199622141410 | -3.20329069846122 |
| F | 1.33291927258099 | -0.28415562074301 | -3.61536990697474 |
| F | 2.39737010230349 | -2.04654401222655 | -3.03662888101386 |
| F | 0.21020014228581 | 0.88865449161909 | 4.71300134240576 |
| F | 0.29853994472011 | -1.24748204947859 | 4.68796298615612 |
| F | -1.60218837489047 | -0.25054900585354 | 4.51010921448833 |
| C | -7.13170235827345 | 2.72733659948355 | 3.51082567040903 |
| C | -7.67414151404853 | 3.44249743858120 | 2.45475470881865 |
| C | -6.86672277895669 | 4.21346274523197 | 1.62811670534913 |
| C | -5.50831309713267 | 4.23973019038948 | 1.86140413194594 |
| C | -4.95568182605711 | 3.49806098911594 | 2.91475696307971 |
| C | -5.76749610828266 | 2.75184750454384 | 3.75296536666146 |
| H | -7.77885869148390 | 2.14001054593182 | 4.14636675706564 |
| H | -8.73933986523989 | 3.40564691064046 | 2.27748320080556 |
| H | -7.30182013858117 | 4.77637966719257 | 0.81463064198899 |
| H | -5.34511216414249 | 2.19332944014575 | 4.57552773423027 |
| C | -4.43497581884115 | 5.00584357810709 | 1.12769946208290 |
| C | -3.17879961851286 | 4.46063657441880 | 1.77221526703463 |
| C | -1.86004134296061 | 4.71258998024848 | 1.46547554140940 |

| | | | |
|---|---|---|---|
| C | -0.84951150877121 | 4.20519035239546 | 2.29294466360621 |
| C | -1.20070424849055 | 3.45481051952351 | 3.41521911522263 |
| C | -2.52034368375815 | 3.15399495060736 | 3.69845439224622 |
| C | -3.51194080355817 | 3.65580742705553 | 2.87397065921804 |
| H | -1.59735569886719 | 5.28914975597467 | 0.58897412667838 |
| H | -0.42475492821621 | 3.11799306319944 | 4.08325619681611 |
| H | -2.76586746643846 | 2.55031379724507 | 4.56103403304237 |
| C | -4.50768853466851 | 4.87925589062209 | -0.40701189809621 |
| H | -3.51760978688716 | 4.63904799214385 | -0.79169152775104 |
| H | -5.18546973020873 | 4.08052452956508 | -0.70505107250826 |
| C | -4.56130920043104 | 6.53266978548470 | 1.40656439832311 |
| H | -3.86280585884734 | 6.85520055846966 | 2.17824777019483 |
| H | -5.57311916054099 | 6.75380783636977 | 1.75141462327015 |
| C | -4.95371335908477 | 6.24670311812477 | -0.92543157755959 |
| H | -6.04023483259899 | 6.33596220219585 | -0.87351093788508 |
| H | -4.64683441669102 | 6.41854931309643 | -1.95696697336629 |
| C | -4.31139355677835 | 7.21269794008755 | 0.06315886439819 |
| H | -4.74034271266000 | 8.21351694090200 | 0.02210627444400 |
| H | -3.23971907504327 | 7.28277571162658 | -0.12946687957987 |
| C | 3.40371901538237 | 2.03503805157486 | -3.20590609378210 |
| C | 3.26851812342168 | 3.00327988106464 | -2.21180846582887 |
| C | 4.35053638925430 | 3.29415858794911 | -1.37658773671454 |
| C | 5.55357621009087 | 2.65645750367183 | -1.58459360947315 |
| C | 5.68855085791488 | 1.71297384574575 | -2.61348651836225 |
| C | 4.60406625026959 | 1.37950915863055 | -3.40872404890705 |
| H | 2.56437106246710 | 1.82370235235358 | -3.84966538934536 |
| H | 4.22869497664015 | 4.02076398852635 | -0.58552967212408 |
| H | 4.69531814520956 | 0.64080287412431 | -4.19213874402065 |
| C | 6.87210609605967 | 2.85979674163713 | -0.87400055729778 |
| C | 7.78702609274530 | 1.93951929142615 | -1.64855971319763 |
| C | 9.13700819963749 | 1.70387693609626 | -1.49050098208905 |
| C | 9.77059788416534 | 0.80904195852839 | -2.34429495298053 |
| C | 9.06571979493316 | 0.16052687624847 | -3.34612754017408 |
| C | 7.70839636077277 | 0.38683274146895 | -3.51215757731088 |
| C | 7.07254529407161 | 1.27192964164922 | -2.65657676494095 |
| H | 9.70705449169515 | 2.20354786350681 | -0.72282946882393 |
| H | 10.82809262458793 | 0.61998541392835 | -2.22799962895274 |
| H | 9.58017998611401 | -0.52569391939750 | -4.00399298312994 |
| H | 7.15642901705239 | -0.11662341353505 | -4.29248583009544 |
| C | 7.33566681253156 | 4.32900531626985 | -0.87304379922780 |
| H | 7.84340990673529 | 4.58478218813095 | -1.80327559491242 |
| H | 6.45852733431116 | 4.97030171336267 | -0.77796013544840 |
| C | 6.80949250716907 | 2.51822280032301 | 0.62412810991038 |
| H | 6.91786093097204 | 1.44809462074314 | 0.80010817970508 |
| H | 5.83458222133167 | 2.82028122680244 | 1.00113089412296 |
| C | 8.24763030008415 | 4.49405809028691 | 0.34961156691585 |
| H | 8.07629159524684 | 5.45665425326604 | 0.83134019259554 |
| H | 9.29519980068996 | 4.46324022897596 | 0.04998008641186 |
| C | 7.92218488873163 | 3.32658415236908 | 1.29938750165013 |
| H | 7.60109420645562 | 3.67860189851220 | 2.28088794810620 |

| | | | |
|---|---|---|---|
| H | 8.80213216803919 | 2.70196785352106 | 1.45438011801863 |
| C | -7.73674646028254 | -1.23290513090226 | 4.25131341850493 |
| C | -8.88156655636520 | -1.50724977103798 | 3.51782331547449 |
| C | -8.93874972683427 | -1.22895880874433 | 2.15833813353244 |
| C | -7.83443248581462 | -0.67685145312615 | 1.54402689471428 |
| C | -6.67422598575240 | -0.40353053491354 | 2.28497396434361 |
| C | -6.62150498157663 | -0.67893253763089 | 3.64220362659757 |
| H | -7.71645029595563 | -1.44916813842898 | 5.30999253300553 |
| H | -9.74011416816723 | -1.93718350652524 | 4.01236541891658 |
| H | -9.83959306662248 | -1.43578248193139 | 1.59914426111999 |
| H | -5.73251003770141 | -0.45550205347482 | 4.21439921539295 |
| C | -7.66836531671566 | -0.24818640128473 | 0.10539725258891 |
| C | -6.25161045104519 | 0.27811516346154 | 0.11211185458608 |
| C | -5.53964889674280 | 0.81772900609214 | -0.93257277598932 |
| C | -4.25478001718266 | 1.35109283417260 | -0.72194746022745 |
| C | -3.72937578174334 | 1.26772213486842 | 0.57028760276136 |
| C | -4.41311965403133 | 0.66520336715974 | 1.61191112927886 |
| C | -5.69197167231808 | 0.18826748411195 | 1.39486038931059 |
| H | -6.00181769970078 | 0.83106935778717 | -1.90710967153446 |
| H | -2.76349165087502 | 1.67318938844764 | 0.79229321590548 |
| H | -3.93771813586242 | 0.59978145128069 | 2.57929258045757 |
| C | -7.90508372317826 | -1.39317464637897 | -0.90998146522813 |
| H | -6.96518043574444 | -1.80651724316489 | -1.27502487689053 |
| H | -8.45858066481540 | -2.19687068565715 | -0.42270882463549 |
| C | -8.69000151285861 | 0.85346825660350 | -0.28722763066478 |
| H | -8.16109763430386 | 1.68371881015702 | -0.75724044482252 |
| H | -9.20500709499946 | 1.24106387337792 | 0.59078785999025 |
| C | -8.75346928653470 | -0.78868174394250 | -2.02785002733961 |
| H | -9.31931148839934 | -1.54555128879609 | -2.56958438565359 |
| H | -8.12022522299272 | -0.25804793910370 | -2.74076084476891 |
| C | -9.64501067148076 | 0.20144528423099 | -1.28648868405877 |
| H | -10.43529644584575 | -0.33550557671209 | -0.75959699189225 |
| H | -10.10880696565807 | 0.93222083876977 | -1.94787065129989 |
| C | 4.79105504503504 | -1.41084024631444 | 3.82054267590424 |
| C | 4.91987207316639 | -0.51555085569110 | 2.75584367876938 |
| C | 5.13001454784604 | -0.99609116083357 | 1.46388046584988 |
| C | 5.25368955395964 | -2.35627217743506 | 1.27477211502975 |
| C | 5.12925420148591 | -3.24745600458683 | 2.35017475676216 |
| C | 4.87832954296695 | -2.77688564347039 | 3.62877951357543 |
| H | 4.60279478999285 | -1.02067137866659 | 4.81115482273858 |
| H | 5.19563639135000 | -0.30847255442450 | 0.63420165249447 |
| H | 4.76306409644541 | -3.45658934340150 | 4.46112456701509 |
| C | 5.55510031678429 | -3.10321252231226 | 0.00058177216242 |
| C | 5.47667494167675 | -4.53784788460247 | 0.46053674535004 |
| C | 5.59165371988503 | -5.69766157645647 | -0.27753049563867 |
| C | 5.50449110161043 | -6.92218737565202 | 0.37401488999525 |
| C | 5.32226406369504 | -6.98490886655454 | 1.74715896711675 |
| C | 5.20607255927694 | -5.82471821744959 | 2.49754106498699 |
| C | 5.27055032346052 | -4.60333626200752 | 1.84679818724255 |
| H | 5.74464311908396 | -5.66271076568062 | -1.34670082622559 |

| | | | |
|---|---|---|---|
| H | 5.58578244108552 | -7.83672037446727 | -0.19552253890066 |
| H | 5.26770527619054 | -7.94716454165590 | 2.23491380015973 |
| H | 5.06624313369002 | -5.87253060787332 | 3.56786129192155 |
| C | 4.58989185492101 | -2.77100977576824 | -1.15832749715301 |
| H | 4.12634303988015 | -3.68489267690264 | -1.53036164135202 |
| H | 3.79337018494509 | -2.10948209935028 | -0.82360344210175 |
| C | 6.96926567308253 | -2.76450965441715 | -0.54050812023525 |
| H | 7.71414833778917 | -3.48018223075181 | -0.19104275336488 |
| H | 7.26049263189403 | -1.77058094031436 | -0.19617060808308 |
| C | 5.44426429838334 | -2.12827591326061 | -2.25327976533747 |
| H | 5.49911053092911 | -1.05215623531008 | -2.09114109633552 |
| H | 5.03221795607195 | -2.30677952890490 | -3.24535231361452 |
| C | 6.82218417036153 | -2.75194436664440 | -2.05791308317004 |
| H | 7.61646810188549 | -2.18338880730643 | -2.53969681220173 |
| H | 6.83468430138838 | -3.76998889543846 | -2.45034449013047 |
| H | -4.00115583897788 | -2.23505483885341 | -3.85868795744719 |
| H | -1.31482605278019 | 0.14465180555973 | -3.27362880840076 |
| H | -0.29907843932954 | 1.51539868696223 | -5.00432057467854 |
| C | -1.82613346226422 | 0.24127566961335 | -4.22735541749999 |
| C | -1.24812138871779 | 1.03246213312785 | -5.21883660545963 |
| C | -3.69751904413291 | -1.28266082160402 | -3.40416639338324 |
| C | -3.03361116321865 | -0.42040157728418 | -4.45449253478416 |
| C | -1.87072131167091 | 1.18206961193115 | -6.45247200713655 |
| H | -1.41414310219778 | 1.79604044777469 | -7.22128906046980 |
| C | -3.65023370660034 | -0.26114647326285 | -5.70214692389625 |
| C | -3.08032372547651 | 0.53229911349145 | -6.69033509384253 |
| H | -4.62209461067211 | -0.78464949727686 | -3.08328202925792 |
| H | -2.51057896908351 | -0.65074083731877 | -1.69253346070193 |
| C | -2.85886300522262 | -1.57662317109151 | -2.16365866427718 |
| H | -1.94923112744051 | -2.12538629918462 | -2.43236921132002 |
| H | -4.58832505908515 | -0.77504699502964 | -5.89549897316003 |
| H | -3.57384241065812 | 0.63779570327785 | -7.65069610683636 |
| C | -3.64061393670064 | -2.36437955412274 | -1.12354623896647 |
| H | -4.60272347575371 | -1.86799574729729 | -0.94459072743281 |
| H | -3.70273120800057 | -2.64419941624192 | 1.01494019679043 |
| H | -3.84390372048697 | -3.38065075753780 | -1.47892524154348 |
| H | -5.23792294730649 | -6.64111722417064 | 0.92461696568504 |
| C | -2.95637621407241 | -2.40713682398484 | 0.26029028312901 |
| H | -7.79159088320334 | -4.83686809454664 | 3.02443565459150 |
| H | -2.55416236059324 | -1.41353543718643 | 0.49326540349394 |
| H | -5.80063719535268 | -7.14281480727850 | 2.53007015336669 |
| C | -5.94579222944147 | -6.41351574678108 | 1.72808751907904 |
| H | -6.68058001430769 | -3.68910730989505 | 3.77727270165764 |
| C | -6.76569171927945 | -4.71097999433256 | 3.39173535808805 |
| H | -3.59899235254810 | -3.01460586181656 | 4.64877562823745 |
| H | -6.96073398642832 | -6.54547061469259 | 1.33340455519472 |
| C | -1.80535777809090 | -3.33096593253796 | 0.40865232500358 |
| C | -5.77636866438331 | -4.96668304093531 | 2.23456392886991 |
| H | -4.41818914292561 | -2.28007574270891 | 3.25811602124092 |
| C | -3.65553971373090 | -3.00375091333539 | 3.55679971112666 |

| | | | |
|---|---|---|---|
| H | -6.62036662759100 | -5.41072247550065 | 4.22037160570834 |
| Si | -4.00289227882067 | -4.70182816757726 | 2.88614052609806 |
| H | -0.80864611468794 | -3.09067675207254 | 0.04405849275954 |
| O | -3.09676954281042 | -4.97221215784961 | 1.41019345341805 |
| N | -1.90933807022204 | -4.44235282500886 | 1.06471152977278 |
| H | -6.00445122553065 | -2.94763904676264 | 1.38275155561543 |
| C | -6.10364093394928 | -3.99812690161408 | 1.08461716878752 |
| H | -2.69540109508851 | -2.62612025539136 | 3.18660434064466 |
| H | -0.38615043810773 | -8.88388023533802 | 3.37580687260624 |
| H | -7.14361254782978 | -4.14995524063016 | 0.77001620163035 |
| H | -0.82822275704824 | -7.38042218942016 | 4.20208816865079 |
| O | -0.90773998933211 | -5.34340406588272 | 1.16866211425372 |
| H | -5.45971813913886 | -4.17088159428708 | 0.21701268192793 |
| C | -0.57559650435169 | -7.81317187333113 | 3.23000979481943 |
| H | 0.63996532638640 | -4.10864382893886 | 4.48847698060010 |
| C | -3.60596688633433 | -6.06356448747791 | 4.11341772552361 |
| C | -0.19226015470518 | -4.30365962577688 | 3.80593625329601 |
| Si | 0.40362187164059 | -5.26853764501612 | 2.33546177631012 |
| H | -1.01978107451410 | -4.72805019816498 | 4.37316273295033 |
| H | -1.43908081797113 | -7.70397256668678 | 2.56697165992747 |
| H | -3.56538506125117 | -7.04402335636020 | 3.62916630100988 |
| H | -4.39102254852462 | -6.09591709204987 | 4.87925592699609 |
| H | -2.65647434558317 | -5.90221886238451 | 4.62941011570562 |
| C | 0.67138272879170 | -7.14495028129218 | 2.62749159798511 |
| H | 1.63775394080142 | -6.81064171338224 | 4.57303230410903 |
| C | 1.85339422029551 | -7.28765922148641 | 3.61060805372553 |
| H | -0.48263432770606 | -3.32595596766025 | 3.39951156983352 |
| H | 2.04914639892139 | -8.35136320497787 | 3.79599371518359 |
| C | 1.89682174764030 | -4.43952020449346 | 1.63367194290382 |
| C | 1.03148885655934 | -7.85744548804392 | 1.30958825721666 |
| H | 1.22260473047864 | -8.91987894550971 | 1.50822637581701 |
| H | 2.48886970304653 | -4.01708694694782 | 2.45002476698938 |
| H | 1.57198102830274 | -3.59578273858098 | 1.01411383997099 |
| H | 2.76467129410680 | -6.84087534872499 | 3.19997037540218 |
| H | 1.93236517811531 | -7.43346096632469 | 0.85334808114399 |
| H | 2.53522279537723 | -5.08580570988017 | 1.02959967635744 |
| H | 0.21427779060391 | -7.79110943902870 | 0.58334671693560 |
| H | 1.20152752472092 | -6.15762736379919 | -1.03743689159732 |
| H | -0.77276738647752 | -7.98616126406418 | -2.52878071559389 |
| C | 1.77324836442288 | -5.56518667889221 | -1.75341097794688 |
| H | 2.83029226049819 | -5.82515905113060 | -1.63116519092959 |
| H | 1.64847882360746 | -4.51181329182648 | -1.47020595560173 |
| C | 0.19643897920936 | -8.41325874053757 | -2.80223901058194 |
| H | 0.03787866302805 | -9.45767446582171 | -3.10326221762320 |
| H | 0.83010163117725 | -8.41636523928403 | -1.90822973065805 |
| H | 2.66850730646659 | -7.87169501623449 | -5.17866450850685 |
| H | -2.90132974918725 | -3.46301375187452 | -5.78757117752214 |
| Si | 1.20062123501487 | -5.80046392793113 | -3.51092476591776 |
| C | 2.19832897849675 | -8.31481329271609 | -4.29544284791989 |
| C | 0.85308389547678 | -7.63600849501242 | -3.95902753125367 |

C -1.97055271845631 -3.79016429261419 -5.32268814703393
C -1.75572895155881 -5.43143976934384 -2.08028578084817
C -1.41417722904503 -4.91803406897331 -3.27809770133246
O -2.37576791149921 -4.39424560727916 -4.07684665902924
O -0.19097548180830 -4.88152042270706 -3.81528902650042
H 2.90611503740760 -8.24521865141164 -3.46067030277388
H -1.32019909168558 -2.92771403368456 -5.14694518846672
H -2.79261049245952 -5.43609478408733 -1.77539187642479
H -1.00522994062825 -5.82099204664555 -1.41012208400415
H 2.02983909310770 -9.38017513101658 -4.49912461703062
H -1.45995576252234 -4.51732564257707 -5.96115173824598
C -0.06670389827536 -7.69966603846436 -5.19299771602899
C 2.42666114378891 -5.04783115473347 -4.70811954682851
H 0.34875256752822 -7.13932281618858 -6.03806453015057
H -0.19444703687889 -8.74299390489847 -5.50976955823545
H 3.41874224082997 -5.49867333684378 -4.60641655335351
H 2.10466384209807 -5.14613808332692 -5.74982143351367
H -1.05657604098603 -7.28917172372884 -4.96859047033973
H 2.52046746576754 -3.98137699244426 -4.47734948081392
315
TS-1 (r²SCAN-3c/xTB)
P 1.71133964862267 1.38185068283354 1.39780944460362
O 3.29058440199413 1.42511574735097 1.28378731339662
O 1.39508894663631 2.30854132289808 2.70337499189689
C 4.06081634864076 1.77761276945356 2.39041532247337
C 4.92063176624914 0.81601918051665 2.97343083361807
C 5.77781381497535 1.22720211308024 3.96846378088789
C 5.77109089144603 2.53995242355561 4.47062679289955
C 6.64403688397585 2.93653429944855 5.50729117722939
C 6.56530938553774 4.18902732006095 6.04712031064471
C 5.59080379519571 5.08763836545967 5.58673709124711
C 4.74357020850910 4.73849998372625 4.57101946592593
C 4.83100207253571 3.46988867340276 3.95587840194513
C 4.00110681452253 3.07098476668680 2.86838019331021
C 3.00184311325242 4.01643805806577 2.32681007679380
C 3.36583500470349 5.29119348682624 1.81514226052357
C 4.70705785679167 5.66920884329510 1.58681502515354
C 5.00945455380178 6.90026205240630 1.07204016228984
C 3.99009210603924 7.82021013759651 0.78110337560959
C 2.68176076292293 7.47872643631126 0.98054518345272
C 2.33377890239486 6.20250438892202 1.47386938126025
C 0.99206910270182 5.82484206360083 1.66034096744623
C 0.63052129645296 4.56810584020290 2.09448052322916
C 1.67375565999260 3.65163830911224 2.39069466024205
H 6.47042020631596 0.51490333548138 4.39394825843927
H 7.36808689438104 2.22178912152808 5.87104952330879
H 7.23481040240556 4.48796238546553 6.83980820500557
H 5.50904128860794 6.06235198159389 6.04455079464586
H 3.99017396639758 5.43617331978078 4.23999830117710
H 5.49641132365786 4.97080926349529 1.81835662913052

| | | | |
|---|---|---|---|
| H | 6.03888287241672 | 7.17489691510224 | 0.89286605312072 |
| H | 4.24960536879568 | 8.79634094305928 | 0.39806308685635 |
| H | 1.88774032610495 | 8.17626242121473 | 0.75749468727240 |
| H | 0.22542317037162 | 6.55779728530032 | 1.45370321492639 |
| N | 1.06975936950564 | 2.17789939800730 | 0.17749053343590 |
| P | 0.01104264097038 | 1.57582710418644 | -0.84229155268931 |
| O | 0.53426847896901 | 2.01925376379343 | -2.31014932219458 |
| O | -1.37454515742414 | 2.32977571691968 | -0.66581465374727 |
| C | 0.75827707107491 | 3.40444898992504 | -2.31487974444967 |
| C | 2.06574110373677 | 3.92506504435095 | -2.17097371666929 |
| C | 2.20363459226950 | 5.29011510258924 | -2.06046270771263 |
| C | 1.09741101955329 | 6.15884500520489 | -2.08384424801801 |
| C | 1.25527008814523 | 7.55665347577675 | -1.96364346487285 |
| C | 0.17121888770131 | 8.38906309949892 | -1.97591823403591 |
| C | -1.12012937667176 | 7.85773467842035 | -2.11422129905676 |
| C | -1.30350471100923 | 6.50983370688423 | -2.26087974263539 |
| C | -0.20498267143076 | 5.62407947629599 | -2.26217280451942 |
| C | -0.34826298663921 | 4.21509925152511 | -2.39644472294390 |
| C | -1.67223592964312 | 3.60000901458034 | -2.64267056116230 |
| C | -2.38076921700278 | 3.93898961159591 | -3.82691678528492 |
| C | -1.82985868549306 | 4.72642630199658 | -4.85962853164614 |
| C | -2.54947828654684 | 4.99143658055413 | -5.99248576829518 |
| C | -3.85084829834146 | 4.48975907504084 | -6.14656297310493 |
| C | -4.40622689536725 | 3.71711823429752 | -5.16652510279384 |
| C | -3.68432821201126 | 3.41578122187566 | -3.99189027889285 |
| C | -4.23423529338453 | 2.62525377691023 | -2.97127217639449 |
| C | -3.53985897473458 | 2.22521213370063 | -1.84568300125770 |
| C | -2.19198951707518 | 2.69302356718038 | -1.74111316610617 |
| H | 3.19160795870191 | 5.71794046475921 | -1.96521415820544 |
| H | 2.25523901098204 | 7.95175530555984 | -1.86056669792890 |
| H | 0.29931003896063 | 9.45739683630261 | -1.88084107562895 |
| H | -1.97185727207734 | 8.52225302330356 | -2.11241479905669 |
| H | -2.29792997127670 | 6.10985226688674 | -2.38090073833671 |
| H | -0.82322836938690 | 5.10291826108404 | -4.75800030105791 |
| H | -2.11388416471355 | 5.58748268930255 | -6.78091778087830 |
| H | -4.40313758697636 | 4.71243193828030 | -7.04728208717046 |
| H | -5.40094545162533 | 3.31198803604737 | -5.28126423087645 |
| H | -5.26465049324877 | 2.33839826884706 | -3.10240550988183 |
| N | 1.28157071645573 | -0.12330997465901 | 1.81525264736620 |
| N | -0.25907033954937 | -0.01683702738976 | -0.93232705887480 |
| S | -0.24237480991742 | -0.25264471559919 | 2.06688253478639 |
| O | -1.04308732944725 | 0.87875360298857 | 1.70466753568911 |
| O | -0.80526749273144 | -1.55827925902781 | 1.83622761987420 |
| S | 1.04475773259432 | -0.85716992651897 | -1.03607446063328 |
| O | 2.28362015099992 | -0.18969167440599 | -0.75879765865379 |
| O | 0.86613554917044 | -2.21509536613269 | -0.59463088072879 |
| C | 1.20270724338449 | -1.19098787343257 | -2.92844128090793 |
| C | -0.30451719041049 | -0.27333856085480 | 3.99403560467315 |
| F | 0.20375022560191 | -1.97144338145739 | -3.36818849899539 |
| F | 1.21228682984173 | -0.11277783591406 | -3.71469725263779 |

| | | | |
|---|---|---|---|
| F | 2.33614306954061 | -1.85390962817031 | -3.19718767544424 |
| F | -0.01447137742464 | 0.86779401756413 | 4.62325183049445 |
| F | 0.56612391804771 | -1.18360039575553 | 4.46118024985042 |
| F | -1.52287165965558 | -0.64574565348901 | 4.43088187205451 |
| C | -7.05198997359767 | 2.62737419726767 | 3.40687645572716 |
| C | -7.59384788960962 | 3.37506096036000 | 2.37328191534459 |
| C | -6.79247771143154 | 4.20490953456968 | 1.59924846050573 |
| C | -5.43978624770722 | 4.25542956033093 | 1.85974126707216 |
| C | -4.88751141580576 | 3.48502028373108 | 2.89232698925014 |
| C | -5.69435375292937 | 2.68028871972562 | 3.67986856328001 |
| H | -7.69388854599791 | 1.99109106294177 | 3.99940544875463 |
| H | -8.65353281997129 | 3.31529700758878 | 2.17076585593423 |
| H | -7.22726770143407 | 4.79099246026188 | 0.80205353999975 |
| H | -5.27252678771595 | 2.09562483785426 | 4.48459751885026 |
| C | -4.37121979304839 | 5.07035086642059 | 1.17336608866598 |
| C | -3.11284672776422 | 4.51310193876842 | 1.80405452297446 |
| C | -1.79355750987487 | 4.77561677246155 | 1.50777196676105 |
| C | -0.78556469930047 | 4.24543048725536 | 2.32387766246816 |
| C | -1.13951075558211 | 3.48184525848590 | 3.43548788399465 |
| C | -2.45842341555754 | 3.16567619527377 | 3.70365281110216 |
| C | -3.44685481488354 | 3.67264768121857 | 2.87915123674186 |
| H | -1.52792942777332 | 5.37300277520654 | 0.64606917668522 |
| H | -0.36535042057325 | 3.13907244255215 | 4.10188139710236 |
| H | -2.70457045659924 | 2.54318712301408 | 4.55265556062268 |
| C | -4.43267246769869 | 5.02084584446406 | -0.36568464132709 |
| H | -3.43951186629335 | 4.80228932551484 | -0.75446300224677 |
| H | -5.10577176475769 | 4.23623120133612 | -0.70810700741053 |
| C | -4.51838359300781 | 6.57990897111667 | 1.52773283270286 |
| H | -3.83603319524784 | 6.86991409118884 | 2.32631739907189 |
| H | -5.53820738503534 | 6.77334641549959 | 1.86572285574423 |
| C | -4.88030072300683 | 6.41083849686725 | -0.81923928318122 |
| H | -5.96781815173034 | 6.49217289387920 | -0.77468988486332 |
| H | -4.56371543569755 | 6.63658585720188 | -1.83735924563429 |
| C | -4.25428644108689 | 7.32851560888645 | 0.22421698069675 |
| H | -4.68696434815992 | 8.32857370127604 | 0.22811021554153 |
| H | -3.18051429247344 | 7.41184395472190 | 0.04903089663737 |
| C | 3.37274065408536 | 2.14144059312748 | -3.27813778794391 |
| C | 3.26736931270707 | 3.08280072707555 | -2.25489861335297 |
| C | 4.36210054372011 | 3.32401177719327 | -1.42048647503841 |
| C | 5.54868573690170 | 2.66588900601386 | -1.65733432887082 |
| C | 5.65486554407787 | 1.75213565318503 | -2.71600862245476 |
| C | 4.55680117127948 | 1.46662342480664 | -3.51133242325389 |
| H | 2.52301439983784 | 1.96617859519137 | -3.91931425805335 |
| H | 4.26244420139305 | 4.02853384156671 | -0.60660892350564 |
| H | 4.62533126501937 | 0.74981918028666 | -4.31712826521636 |
| C | 6.87599157045282 | 2.81597870666700 | -0.94998090719887 |
| C | 7.76447615148526 | 1.90060637181229 | -1.76024275096032 |
| C | 9.10977900392037 | 1.62982365032951 | -1.61976342921985 |
| C | 9.71719208097278 | 0.74852367442673 | -2.50602774933074 |
| C | 8.99123996773080 | 0.14890048854226 | -3.52308015811692 |

| | | | |
|---|---|---|---|
| C | 7.63836972669831 | 0.41099974176765 | -3.67194124377055 |
| C | 7.02826244915593 | 1.28201404232702 | -2.78378804696165 |
| H | 9.69600293327278 | 2.09139409093725 | -0.84051068093227 |
| H | 10.77078658873422 | 0.53195650932460 | -2.40337342044128 |
| H | 9.48579445256463 | -0.52704229190297 | -4.20636668269002 |
| H | 7.07011266316562 | -0.05404344917436 | -4.46434040939131 |
| C | 7.37396694234155 | 4.27308349244064 | -0.90371174548538 |
| H | 7.88223667096719 | 4.54768053188646 | -1.82820856243870 |
| H | 6.51288575214118 | 4.93124440756862 | -0.78165373667190 |
| C | 6.81380755324577 | 2.42692009153565 | 0.53639637798996 |
| H | 6.89723022497807 | 1.34930999792149 | 0.67653518697681 |
| H | 5.84870735947419 | 2.73977668450329 | 0.92930982103718 |
| C | 8.29657643282646 | 4.37629121934438 | 0.31768820736572 |
| H | 8.15061901664465 | 5.32611044079513 | 0.83206114793946 |
| H | 9.34134960131360 | 4.33111555134171 | 0.01044301913046 |
| C | 7.94934871792184 | 3.18587334647064 | 1.23046861187996 |
| H | 7.64264775322706 | 3.51258859622034 | 2.22518093518385 |
| H | 8.81503556653198 | 2.53581551542221 | 1.35871868958159 |
| C | -7.64366936056933 | -1.56255143231416 | 3.97817981816581 |
| C | -8.74974430468056 | -1.88903407612381 | 3.20735918128335 |
| C | -8.80665382901078 | -1.54428900655854 | 1.86300929246253 |
| C | -7.73690665442892 | -0.88121371033211 | 1.29874478851149 |
| C | -6.61064721895335 | -0.56592483537442 | 2.07443807841750 |
| C | -6.56384923718053 | -0.89586453735735 | 3.41980621516651 |
| H | -7.62566358987852 | -1.82692191116017 | 5.02596689023651 |
| H | -9.57981896393460 | -2.41003618139123 | 3.66128420829369 |
| H | -9.68111568083901 | -1.78849881730598 | 1.27781250249300 |
| H | -5.70621965054857 | -0.62873319164935 | 4.02010782708033 |
| C | -7.58357852068597 | -0.35514793242704 | -0.10912251390081 |
| C | -6.19699541731356 | 0.24526885790070 | -0.05481180353831 |
| C | -5.49867794101320 | 0.87178748062199 | -1.05963438580344 |
| C | -4.24278439160271 | 1.45130738899992 | -0.80156874092980 |
| C | -3.73347657995370 | 1.33716876266833 | 0.49437208794703 |
| C | -4.40356153660975 | 0.65561107471868 | 1.49477843919341 |
| C | -5.65228404972416 | 0.12514431774716 | 1.23193435999301 |
| H | -5.94415883567846 | 0.91277117056156 | -2.04176226528879 |
| H | -2.79112121598768 | 1.77785350262877 | 0.74889454793661 |
| H | -3.94159182444746 | 0.56590984547054 | 2.46671835398973 |
| C | -7.76648247675473 | -1.43903945223442 | -1.19972246717863 |
| H | -6.81060939989297 | -1.75105999310109 | -1.61914983051991 |
| H | -8.24053497143773 | -2.31722297212779 | -0.75939750270130 |
| C | -8.65766539285638 | 0.72039295890051 | -0.42841190182589 |
| H | -8.17398136182896 | 1.58940561283134 | -0.87685325039991 |
| H | -9.16478578455220 | 1.05069120671797 | 0.47734710824741 |
| C | -8.68754581801477 | -0.82366155273792 | -2.25337567436886 |
| H | -9.22705716665245 | -1.58108290602874 | -2.82056158513456 |
| H | -8.10983009253931 | -0.21712552374172 | -2.95301553893888 |
| C | -9.60897745907343 | 0.07086229099598 | -1.43166792865828 |
| H | -10.34997972440731 | -0.53774149704294 | -0.91123115091973 |
| H | -10.13428910238055 | 0.80870829030693 | -2.03687502610286 |

| | | | |
|---|---|---|---|
| C | 4.83913540164720 | -1.53809550147287 | 3.64662440278580 |
| C | 4.92175599895180 | -0.61080042566272 | 2.60416336762620 |
| C | 5.08647103404278 | -1.05465323923964 | 1.29232927576596 |
| C | 5.21036018640019 | -2.40846021716078 | 1.06082135286585 |
| C | 5.13493420734535 | -3.33084555582376 | 2.11420973567204 |
| C | 4.93074432820214 | -2.89753545616027 | 3.41405663244964 |
| H | 4.68198735557101 | -1.17890554992117 | 4.65419496542118 |
| H | 5.11613076501290 | -0.34459385257227 | 0.47973322092435 |
| H | 4.85131341179070 | -3.60020798124003 | 4.23128054238511 |
| C | 5.47306149049651 | -3.11702905529605 | -0.24351665804702 |
| C | 5.40021057589331 | -4.56501045725286 | 0.17383585245990 |
| C | 5.47459232416729 | -5.70226714401790 | -0.60364291505540 |
| C | 5.41347790526599 | -6.94621780423850 | 0.01361831386327 |
| C | 5.30311286647016 | -7.04972593994679 | 1.39182348854362 |
| C | 5.22842885676213 | -5.91263232653188 | 2.18145417471724 |
| C | 5.25746557115698 | -4.67145295341899 | 1.56603892737148 |
| H | 5.57488388206814 | -5.63542269078714 | -1.67747040160653 |
| H | 5.46076474062737 | -7.84283186942246 | -0.58739398849209 |
| H | 5.27217346617066 | -8.02539115253791 | 1.85381539942828 |
| H | 5.15285693025269 | -5.99529021605858 | 3.25564066032063 |
| C | 4.49386998433001 | -2.74432899782824 | -1.37684004554459 |
| H | 3.99403987004096 | -3.64009234588010 | -1.74637915750767 |
| H | 3.72550771451961 | -2.06298742586162 | -1.01688666387058 |
| C | 6.88168809554179 | -2.77388154758367 | -0.79695986752246 |
| H | 7.63142711455687 | -3.49021715217755 | -0.45941137314999 |
| H | 7.17452741319533 | -1.78103747709067 | -0.45071847988510 |
| C | 5.34178430417776 | -2.11320543953770 | -2.48483150995750 |
| H | 5.41226290840401 | -1.03807306303398 | -2.32336054247874 |
| H | 4.91406816814058 | -2.28524822185912 | -3.47127898002179 |
| C | 6.71557863575788 | -2.75252640418826 | -2.31163852755395 |
| H | 7.50896699300740 | -2.18905516156288 | -2.80066324928592 |
| H | 6.71391023220758 | -3.76864112863406 | -2.70898355569130 |
| H | -4.04404669085042 | -2.11357097652737 | -3.96446632341998 |
| H | -1.39578123861986 | 0.29430861500623 | -3.21159466191770 |
| H | -0.37798431585870 | 1.75780370600267 | -4.86749007909289 |
| C | -1.88263844267383 | 0.41041398312855 | -4.17633533063433 |
| C | -1.30596276797305 | 1.25521642248820 | -5.12347269873968 |
| C | -3.72740483838781 | -1.19234976972386 | -3.45705760399373 |
| C | -3.06094802290410 | -0.28037091393941 | -4.46044827217453 |
| C | -1.90304795785122 | 1.43214049095063 | -6.36623034184173 |
| H | -1.44800047717733 | 2.09014113322226 | -7.09870230768295 |
| C | -3.65077830095564 | -0.09575341516423 | -5.71776702067330 |
| C | -3.08397450622425 | 0.75364304960750 | -6.66022359023242 |
| H | -4.65045592201346 | -0.70961916651677 | -3.11030723130564 |
| H | -2.59601079812482 | -0.68155742344296 | -1.66041115572774 |
| C | -2.90705816076929 | -1.56923068865787 | -2.22457558574235 |
| H | -1.97094662587448 | -2.06112116197986 | -2.51107238621095 |
| H | -4.56747908473952 | -0.62999139489594 | -5.95362004665375 |
| H | -3.55672553446386 | 0.88027693475501 | -7.62844035317109 |
| C | -3.71071607966271 | -2.47021913516329 | -1.29885485484140 |

| | | | |
|---|---|---|---|
| H | -4.65486192652181 | -1.96367510967102 | -1.06099056859463 |
| H | -3.83436384382704 | -3.10910639717247 | 0.73540243245363 |
| H | -3.95618570907886 | -3.41328263125348 | -1.79899233813056 |
| H | -5.08263454851290 | -7.01528782856054 | 0.67524739837286 |
| C | -3.06434525349031 | -2.74478539389066 | 0.05982069499564 |
| H | -7.86038256049348 | -5.42048444818041 | 2.65206692318141 |
| H | -2.68487202022154 | -1.80032573586108 | 0.48014725238273 |
| H | -5.68374941384001 | -7.57032961286422 | 2.24864270470875 |
| C | -5.84354983473815 | -6.84646770129759 | 1.44429782510522 |
| H | -6.87402623023014 | -4.19962467180680 | 3.46555375669105 |
| C | -6.86616694879711 | -5.22209652753941 | 3.07140293303688 |
| H | -3.86901732871085 | -3.25388018090016 | 4.45427299101591 |
| H | -6.82632474053163 | -7.04951773341696 | 1.00057040935234 |
| C | -1.87566773949870 | -3.64809870197886 | 0.17289780070686 |
| C | -5.80196770497306 | -5.39714627893224 | 1.96716509808773 |
| H | -4.76534431679936 | -2.64662091057561 | 3.05072680288490 |
| C | -3.92628658791616 | -3.27457392737706 | 3.36251183267503 |
| H | -6.71166656584756 | -5.91569784641541 | 3.90365904805855 |
| Si | -4.08859487297885 | -5.01281907380337 | 2.71922027748488 |
| H | -0.88637177510320 | -3.29374976300496 | -0.10607322210324 |
| O | -3.04742243260468 | -5.26179508397580 | 1.34983423445384 |
| N | -1.89443064465549 | -4.57117166175380 | 1.12364423048574 |
| H | -6.12633050034216 | -3.38941283251876 | 1.11471488159816 |
| C | -6.14056613091176 | -4.44240993783431 | 0.80739340788785 |
| H | -3.01401776303318 | -2.80271016383485 | 2.98325281039297 |
| H | 0.09839881552971 | -8.57391620675654 | 3.98180097248443 |
| H | -7.15204937837107 | -4.66230635172047 | 0.44244122684803 |
| H | -0.63481834467572 | -7.08835578579174 | 4.60725533619515 |
| O | -0.80407346377916 | -5.37134263407388 | 1.33588401235141 |
| H | -5.44775165983182 | -4.56389302597195 | -0.03146473106750 |
| C | -0.24968132249931 | -7.57100820927977 | 3.70439291246941 |
| H | 0.23817489080864 | -3.75340706563612 | 4.68396104016053 |
| C | -3.67681516591902 | -6.31374872328543 | 4.00691903554496 |
| C | -0.48917133663896 | -4.04858411939267 | 3.92188252939522 |
| Si | 0.36497572304755 | -5.00403234280506 | 2.57594275414907 |
| H | -1.33914807451612 | -4.52628122410891 | 4.40946325561431 |
| H | -1.07306108227583 | -7.67777811618358 | 2.99190792591873 |
| H | -3.60418578332368 | -7.30913311110978 | 3.55724696384709 |
| H | -4.45241877461789 | -6.34239194276471 | 4.78122903525788 |
| H | -2.72532453387595 | -6.10570636847686 | 4.50291919109196 |
| C | 0.91259822475419 | -6.76888460611500 | 3.09632630546054 |
| H | 1.64627089312874 | -6.07413506086504 | 5.04742022611703 |
| C | 2.01924590913615 | -6.59527726790071 | 4.15862773514850 |
| H | -0.82010277753706 | -3.12356619725687 | 3.43514822376453 |
| H | 2.38897822913131 | -7.58005006435285 | 4.47161146223891 |
| C | 1.77209390592475 | -4.00269197250301 | 1.91724596916056 |
| C | 1.48616185567192 | -7.55311266564512 | 1.90142295508413 |
| H | 1.85043705703397 | -8.53001578255033 | 2.24481220150603 |
| H | 2.25320127764883 | -3.45754085120588 | 2.73435914028601 |
| H | 1.38064102143727 | -3.24385528163153 | 1.23105071637707 |

| | | | |
|---|---|---|---|
| H | 2.86246877129937 | -6.02344017484205 | 3.75878769978027 |
| H | 2.32486318215481 | -7.02829560314705 | 1.43136116659971 |
| H | 2.52666307572187 | -4.58471889377955 | 1.38574927135644 |
| H | 0.71970321189545 | -7.72829457311878 | 1.13936374564802 |
| H | 1.06527231695040 | -6.02285706579813 | -0.55610962084446 |
| H | -0.99962902392285 | -7.90992328437015 | -2.06147609262934 |
| C | 1.66024389569943 | -5.49090044636989 | -1.29970938558638 |
| H | 2.70851784063533 | -5.77434904378270 | -1.15737873272155 |
| H | 1.56381238806427 | -4.41882540525314 | -1.08190444988092 |
| C | -0.02058409939228 | -8.34519703325060 | -2.28182519038968 |
| H | -0.17235257937736 | -9.39948017103335 | -2.54849974831415 |
| H | 0.57770318641657 | -8.31515305426435 | -1.36464491187573 |
| H | 2.52505636584082 | -7.92980007282344 | -4.60396474440266 |
| H | -2.99102513400941 | -3.69152429787936 | -5.53512654627463 |
| Si | 1.08789254961426 | -5.78301506811984 | -3.04066991661756 |
| C | 2.02380808648924 | -8.33451746420506 | -3.71981239293319 |
| C | 0.68379838669637 | -7.61681400963822 | -3.44205406707833 |
| C | -2.05916506453828 | -3.92937812105802 | -5.02259598748653 |
| C | -1.91646292663502 | -5.31002788521581 | -1.64125226190744 |
| C | -1.53629041433303 | -4.86926534557446 | -2.87974315427469 |
| O | -2.47535364881378 | -4.41807141769430 | -3.72346700990653 |
| O | -0.30744968519107 | -4.84459161552978 | -3.36095657854268 |
| H | 2.71021904918595 | -8.25240488861250 | -2.86841998331694 |
| H | -1.44202556104759 | -3.03328288790270 | -4.91612586364609 |
| H | -2.96747554569956 | -5.46799375842206 | -1.44132783401088 |
| H | -1.18576750631125 | -5.81410531996119 | -1.02657570583837 |
| H | 1.83663931497888 | -9.40164754982216 | -3.89398561783480 |
| H | -1.50768102558362 | -4.70317008984605 | -5.56295932922329 |
| C | -0.20189924492233 | -7.69900608947420 | -4.69946363488813 |
| C | 2.30305434841873 | -5.09111989129819 | -4.27916715363227 |
| H | 0.24949322747745 | -7.17644719215225 | -5.54982537160668 |
| H | -0.34483591289034 | -8.74871603954760 | -4.98613860726096 |
| H | 3.29131264618609 | -5.54744997616554 | -4.16338200255431 |
| H | 1.97461104111945 | -5.23234605015540 | -5.31373031878552 |
| H | -1.18988254714061 | -7.26301082050477 | -4.51753712042056 |
| H | 2.40948029983753 | -4.01688077704303 | -4.09722067452702 |

315

P-1 ($r^2$SCAN-3c/xTB)

| | | | |
|---|---|---|---|
| P | 1.77583433828117 | 1.44130032774772 | 1.30600332481756 |
| O | 3.35485925492128 | 1.49279670705219 | 1.16299086891499 |
| O | 1.49121728121413 | 2.34497503124149 | 2.63520376622805 |
| C | 4.13841028074910 | 1.81535360967120 | 2.26803728597934 |
| C | 4.98295016097335 | 0.83028806657835 | 2.83230187753640 |
| C | 5.85576271006777 | 1.21009881935910 | 3.82547472076236 |
| C | 5.88141543611248 | 2.51773577972630 | 4.34090063665425 |
| C | 6.77299395341082 | 2.88478024486205 | 5.37249872931681 |
| C | 6.72327337274154 | 4.13095187893564 | 5.93010364113443 |
| C | 5.75944558099260 | 5.05263168171955 | 5.49369796404563 |
| C | 4.89413375340163 | 4.73204779479557 | 4.48379035871317 |
| C | 4.95185307880192 | 3.47110265417421 | 3.84969410951145 |

| | | | |
|---|---|---|---|
| C | 4.10087874942695 | 3.10118477667905 | 2.76830301682208 |
| C | 3.09427555448360 | 4.05879401631317 | 2.26402711848818 |
| C | 3.44739327448054 | 5.34403081991463 | 1.77250515299608 |
| C | 4.78348140050869 | 5.72436229755968 | 1.51933057332537 |
| C | 5.07634546768192 | 6.96681490474629 | 1.02704504789534 |
| C | 4.05238210139247 | 7.89581463100260 | 0.78527890166265 |
| C | 2.74839027838657 | 7.55194329068611 | 1.00827582556790 |
| C | 2.40910858124726 | 6.26446425229362 | 1.47783509417144 |
| C | 1.07131029238892 | 5.88302866489667 | 1.68475668487100 |
| C | 0.71853731576212 | 4.61533303380102 | 2.09371591830496 |
| C | 1.76719927509498 | 3.69181609168219 | 2.34576555908905 |
| H | 6.53246490830766 | 0.47652073652641 | 4.24067562570608 |
| H | 7.48779328116479 | 2.15211097185622 | 5.71870903842012 |
| H | 7.40754054527800 | 4.40717033976998 | 6.71849149946602 |
| H | 5.70023489398767 | 6.02220473721615 | 5.96576197440667 |
| H | 4.14922413710284 | 5.44746125790549 | 4.17200233413206 |
| H | 5.57614544485973 | 5.01829205882549 | 1.71389207425114 |
| H | 6.10167112332220 | 7.24370833465145 | 0.82845273193534 |
| H | 4.30471154265593 | 8.88075868703550 | 0.42026267142187 |
| H | 1.95121830562621 | 8.25651747008325 | 0.82127614960564 |
| H | 0.30117728719979 | 6.62147272879292 | 1.51327652085506 |
| N | 1.11108528303225 | 2.25530661536001 | 0.10855420748448 |
| P | 0.04330034268720 | 1.64853132907136 | -0.90057041712424 |
| O | 0.54843028011894 | 2.11360057699182 | -2.37192495521693 |
| O | -1.35036840983334 | 2.38436068084626 | -0.70788119674037 |
| C | 0.78657522361044 | 3.49561823733049 | -2.35029662166539 |
| C | 2.10121079117185 | 4.00081860719311 | -2.21491881422378 |
| C | 2.25430517332806 | 5.36241274026428 | -2.08151384831999 |
| C | 1.15683011934213 | 6.24198930388535 | -2.06448432077916 |
| C | 1.33046998864852 | 7.63500136657357 | -1.91490203109133 |
| C | 0.25407775616913 | 8.47666825893044 | -1.88076746732497 |
| C | -1.04495035474571 | 7.95976429538148 | -2.00046273868781 |
| C | -1.24369634083028 | 6.61772667268852 | -2.17744120690970 |
| C | -0.15361940050900 | 5.72302552253883 | -2.22718456630669 |
| C | -0.31255846868290 | 4.31915360712798 | -2.39408691670053 |
| C | -1.64771856508202 | 3.72903214938127 | -2.63767511686333 |
| C | -2.36670502029643 | 4.12011225892324 | -3.79974738933323 |
| C | -1.81220993974186 | 4.92244495261670 | -4.81906181166019 |
| C | -2.54397359794480 | 5.24198305972115 | -5.92995153278044 |
| C | -3.86218936153843 | 4.78293251729175 | -6.07403180923967 |
| C | -4.42111624556407 | 3.99570862194691 | -5.10763636086735 |
| C | -3.68628867179548 | 3.63692145824427 | -3.95737832812075 |
| C | -4.23707036142148 | 2.82689997735901 | -2.95297533258004 |
| C | -3.53175125886508 | 2.36924765587449 | -1.85652924250933 |
| C | -2.17096132059675 | 2.80223876363156 | -1.75830427987368 |
| H | 3.24771573604269 | 5.77959398699281 | -1.99580204573984 |
| H | 2.33598367755570 | 8.01897169955395 | -1.82417730274182 |
| H | 0.39414415996227 | 9.54125484219308 | -1.76265473624649 |
| H | -1.89056992334372 | 8.63091810543904 | -1.95956194907883 |
| H | -2.24445929356080 | 6.22961683874009 | -2.28293144031665 |

| | | | |
|---|---|---|---|
| H | -0.79345468471415 | 5.26729756238536 | -4.72537080967514 |
| H | -2.10540126838088 | 5.84886976875294 | -6.70852851590818 |
| H | -4.42441766931440 | 5.04990931456097 | -6.95652419120963 |
| H | -5.42916833219343 | 3.62251579865478 | -5.21525603946345 |
| H | -5.27717871021029 | 2.57119482654267 | -3.07239587993278 |
| N | 1.35335133143397 | -0.07212797213960 | 1.68703165143822 |
| N | -0.20551420212515 | 0.05342768508554 | -1.01544876116597 |
| S | -0.17991158779037 | -0.22496749088062 | 1.89346113821874 |
| O | -0.96428859074484 | 0.94236303828206 | 1.60613235664362 |
| O | -0.73629291128300 | -1.50320340559526 | 1.56121570494700 |
| S | 1.10658849828122 | -0.77238296722128 | -1.12112211890906 |
| O | 2.34468684865331 | -0.08539748574107 | -0.89462638058306 |
| O | 0.97284487240169 | -2.14088009903833 | -0.70444241968424 |
| C | 1.20339878942839 | -1.10740116523378 | -3.02819674462731 |
| C | -0.27283633124676 | -0.34119542109601 | 3.81702272558228 |
| F | 0.20998416762380 | -1.94091243323870 | -3.40537603615339 |
| F | 1.12873162102753 | -0.04782403140302 | -3.83417183475811 |
| F | 2.34008918844235 | -1.74162565378862 | -3.35895258877090 |
| F | 0.07770837848026 | 0.76855783619602 | 4.47782547516428 |
| F | 0.53412795796697 | -1.31031901550041 | 4.27769711532133 |
| F | -1.51867159717171 | -0.64536617321381 | 4.22811303128256 |
| C | -6.92854360370537 | 2.54967617754349 | 3.41359855177088 |
| C | -7.48829628932646 | 3.32848558774600 | 2.41297808255299 |
| C | -6.70406566362202 | 4.19900353434414 | 1.66642530062260 |
| C | -5.35042710770805 | 4.25847950323457 | 1.91969012967863 |
| C | -4.78017763172289 | 3.45809401680015 | 2.91927720886207 |
| C | -5.57002200836207 | 2.61217912075515 | 3.68051633432353 |
| H | -7.55679074134009 | 1.88022009785868 | 3.98392436333840 |
| H | -8.54826155541696 | 3.26001955954396 | 2.21437359039128 |
| H | -7.15267710210349 | 4.80803657680461 | 0.89419192061870 |
| H | -5.13456069657856 | 2.00112770190771 | 4.45811194509331 |
| C | -4.29762382395195 | 5.11119180142443 | 1.25536721227695 |
| C | -3.02745712433768 | 4.54464123861600 | 1.85314064160984 |
| C | -1.71402687771092 | 4.82864796619214 | 1.55167950926015 |
| C | -0.69211299309380 | 4.27966935531253 | 2.33755022748530 |
| C | -1.02716484680294 | 3.47605254957910 | 3.42694647337578 |
| C | -2.34033937813756 | 3.13864753437316 | 3.69772688826101 |
| C | -3.34209709197325 | 3.66358531141031 | 2.90113052534584 |
| H | -1.46287419099115 | 5.45607963595096 | 0.70700523774895 |
| H | -0.24359599521160 | 3.11234057332592 | 4.07138620828947 |
| H | -2.57153519759471 | 2.48308917706967 | 4.52582391439258 |
| C | -4.37160959382773 | 5.12075017538272 | -0.28392012309944 |
| H | -3.38380204790920 | 4.90763087424583 | -0.68887654867022 |
| H | -5.05445843066563 | 4.35540340355905 | -0.64949659813776 |
| C | -4.45560543553122 | 6.60423664678903 | 1.66874265248179 |
| H | -3.78097496905350 | 6.86602552002091 | 2.48353139949535 |
| H | -5.47921067936815 | 6.77918965335693 | 2.00569106952037 |
| C | -4.81234618261440 | 6.52990523754149 | -0.68287657652697 |
| H | -5.89973019163249 | 6.61370289492367 | -0.63760771095075 |
| H | -4.49249332551751 | 6.79477407843459 | -1.69051991524084 |

| | | | |
|---|---|---|---|
| C | -4.18625656840173 | 7.40406584977069 | 0.39727080532911 |
| H | -4.61647801691647 | 8.40436715701339 | 0.43976500654486 |
| H | -3.11182527721387 | 7.49154177419474 | 0.22811126114727 |
| C | 3.37928137336828 | 2.20617052864094 | -3.34584656149624 |
| C | 3.29441211302460 | 3.14996075776818 | -2.32283338004556 |
| C | 4.40376054563868 | 3.38660320109434 | -1.50626079467938 |
| C | 5.58479298132327 | 2.72712530673593 | -1.76393886283223 |
| C | 5.67149850934285 | 1.81480586636150 | -2.82549655140965 |
| C | 4.55831570723660 | 1.52993131514601 | -3.59992491894758 |
| H | 2.51730245702741 | 2.03023457316495 | -3.97059497619057 |
| H | 4.31942612457942 | 4.08920792645281 | -0.68896171933047 |
| H | 4.61140411703008 | 0.81189209858746 | -4.40582110957022 |
| C | 6.92377463023084 | 2.87480440684486 | -1.07857722986904 |
| C | 7.79852000843836 | 1.96387722952870 | -1.90849584089251 |
| C | 9.14741351347861 | 1.69787486188552 | -1.79607966861008 |
| C | 9.74006295804197 | 0.82081882158635 | -2.69650660964905 |
| C | 8.99561879727587 | 0.22060910788261 | -3.69975112778946 |
| C | 7.63925260496578 | 0.47906946663047 | -3.82129152645319 |
| C | 7.04402616839630 | 1.34644147536417 | -2.91956293857986 |
| H | 9.74773262700422 | 2.16121804050682 | -1.02862743780966 |
| H | 10.79637330801728 | 0.60798989004438 | -2.61565662188263 |
| H | 9.47876019795123 | -0.45193117983163 | -4.39448594819978 |
| H | 7.05685694725928 | 0.01400538805843 | -4.60327488620228 |
| C | 7.42215233166034 | 4.33179298171867 | -1.03215517357837 |
| H | 7.91951813298660 | 4.61015069380576 | -1.96157977188919 |
| H | 6.56259291419971 | 4.98960679051602 | -0.89768193415522 |
| C | 6.88515687397769 | 2.47750421846081 | 0.40660042948115 |
| H | 6.96835025547082 | 1.39898767266838 | 0.54015245807088 |
| H | 5.92747652724508 | 2.79042108507897 | 0.81730459555328 |
| C | 8.35932721620651 | 4.43160437203680 | 0.17883729193715 |
| H | 8.21138125256199 | 5.37549493425431 | 0.70351850395715 |
| H | 9.40081169834644 | 4.39839409289425 | -0.14124776861133 |
| C | 8.03340545242210 | 3.23055915170727 | 1.08556649117632 |
| H | 7.74700389963820 | 3.54544384466630 | 2.09013438910166 |
| H | 8.90280852736277 | 2.58077087371229 | 1.18811435864529 |
| C | -7.54542317073702 | -1.78287403019868 | 3.78275624468711 |
| C | -8.64939518776587 | -2.08722055718421 | 3.00043561250781 |
| C | -8.72743573518464 | -1.65555552024338 | 1.68203840776622 |
| C | -7.68109697330278 | -0.92705426414899 | 1.15620254289732 |
| C | -6.55611754044123 | -0.63408036922986 | 1.94233546337127 |
| C | -6.48777838121041 | -1.05271584266650 | 3.26145370903671 |
| H | -7.50854547970071 | -2.12062090031044 | 4.80875215150465 |
| H | -9.46112704230590 | -2.66088531901801 | 3.42381253115501 |
| H | -9.59959295724083 | -1.88734504599994 | 1.08787876566584 |
| H | -5.62506763437381 | -0.81565656197036 | 3.86677643060908 |
| C | -7.55117065596212 | -0.31227402848689 | -0.21787092080408 |
| C | -6.17626641582087 | 0.31237370153096 | -0.13655138763567 |
| C | -5.49216164014149 | 0.99866636610288 | -1.11130535771899 |
| C | -4.23289325296281 | 1.56336453849280 | -0.83619009539592 |
| C | -3.71313103661862 | 1.38902530229316 | 0.44870886169150 |

| | | | |
|---|---|---|---|
| C | -4.37367441425669 | 0.65607391393644 | 1.41888065595153 |
| C | -5.61871146110665 | 0.12686909061838 | 1.13706210733666 |
| H | -5.94660970971256 | 1.09147285457676 | -2.08612415933647 |
| H | -2.76831598557358 | 1.81766042059422 | 0.71568084720871 |
| H | -3.90386580866801 | 0.51751373991720 | 2.38120004768266 |
| C | -7.71769441930326 | -1.33451858829175 | -1.36918828320015 |
| H | -6.75800897722961 | -1.59873290060977 | -1.81167177426792 |
| H | -8.16785296198830 | -2.24823274031065 | -0.97803015353653 |
| C | -8.65067354797892 | 0.75535492711804 | -0.46739932304509 |
| H | -8.18945262045028 | 1.65944411422470 | -0.86788744555778 |
| H | -9.15836038327610 | 1.02126986652347 | 0.45911466985750 |
| C | -8.66073305204226 | -0.68014065713198 | -2.37907782172044 |
| H | -9.18840509564496 | -1.41495975129214 | -2.98601868003224 |
| H | -8.10237610540857 | -0.02018762895386 | -3.04547193815612 |
| C | -9.59537172909791 | 0.14398404197377 | -1.50051220758228 |
| H | -10.31818671118506 | -0.51059234980020 | -1.01112683084177 |
| H | -10.14230657806632 | 0.90342511533966 | -2.05822895881445 |
| C | 4.79365259703614 | -1.52354430539959 | 3.48175640429840 |
| C | 4.93816017576365 | -0.59249039489770 | 2.45007115261872 |
| C | 5.11646279398754 | -1.03025259384818 | 1.13859060926642 |
| C | 5.19478601314092 | -2.38570849853465 | 0.89777018871573 |
| C | 5.05125004293120 | -3.31292787114457 | 1.93947479137436 |
| C | 4.83216147233671 | -2.88335337772852 | 3.23787928978874 |
| H | 4.63071907227157 | -1.16564644387733 | 4.48908763999249 |
| H | 5.19409235574811 | -0.31386977149814 | 0.33456560333960 |
| H | 4.70448978516750 | -3.58901661419945 | 4.04629495294895 |
| C | 5.47024295813809 | -3.09159631600566 | -0.40571797344679 |
| C | 5.36409881900176 | -4.54016695340356 | 0.00147051398807 |
| C | 5.46123345260114 | -5.67319667429569 | -0.77988992474752 |
| C | 5.35148712254674 | -6.92062386756701 | -0.17626548156471 |
| C | 5.16305555963472 | -7.03053997545366 | 1.19286407725817 |
| C | 5.06445431816901 | -5.89755366466092 | 1.98627210786201 |
| C | 5.15337397321913 | -4.65253219659589 | 1.38440259079073 |
| H | 5.61900031207058 | -5.59961348726747 | -1.84624684049376 |
| H | 5.42002008965293 | -7.81399125653793 | -0.77977768790839 |
| H | 5.08991335505662 | -8.00829045168068 | 1.64554958426140 |
| H | 4.92126730879981 | -5.98669608695403 | 3.05272291922210 |
| C | 4.50207429148923 | -2.70173700286695 | -1.54405153823886 |
| H | 4.02366198477265 | -3.59710015262604 | -1.94308507733609 |
| H | 3.71865664302048 | -2.04100111206181 | -1.17821366293628 |
| C | 6.88660698336012 | -2.76250530166528 | -0.94660612739824 |
| H | 7.61874183155156 | -3.50664146630527 | -0.63136460900650 |
| H | 7.20192760552453 | -1.78935741849336 | -0.56645299121335 |
| C | 5.36012191147918 | -2.03222695800382 | -2.61990580267692 |
| H | 5.43515212840382 | -0.96516118300576 | -2.41445371977493 |
| H | 4.93837973335262 | -2.16138442460662 | -3.61548288015852 |
| C | 6.72796408278427 | -2.68770264838396 | -2.46108623331294 |
| H | 7.52847023781227 | -2.11439244854881 | -2.92640561680331 |
| H | 6.72026072185427 | -3.68975541840396 | -2.89301461257485 |
| H | -4.11142248338958 | -2.01378772760992 | -3.89935247587997 |

| | | | |
|---|---|---|---|
| H | -1.43229160235421 | 0.34931970003267 | -3.15497580476819 |
| H | -0.45370693118612 | 1.85405548908782 | -4.80261766790352 |
| C | -1.94711461782561 | 0.49903830441496 | -4.10091471215176 |
| C | -1.39398207225713 | 1.36698105062679 | -5.04203165135995 |
| C | -3.78736407828550 | -1.10504251689130 | -3.37211811947750 |
| C | -3.14174282849673 | -0.16986828911477 | -4.36684567036049 |
| C | -2.03037631452394 | 1.58751556922908 | -6.25801534072330 |
| H | -1.59398697855589 | 2.26568152322083 | -6.98344315693790 |
| C | -3.77205567720461 | 0.05912525193986 | -5.59755771484142 |
| C | -3.22814841017944 | 0.93078562188389 | -6.53272094276673 |
| H | -4.71097053984789 | -0.63732398197169 | -3.00703076174626 |
| H | -2.66531425545902 | -0.64439716147859 | -1.55105980614219 |
| C | -2.96812742223440 | -1.51344983483540 | -2.14783014861786 |
| H | -2.01393458540829 | -1.97099093877399 | -2.44080991014826 |
| H | -4.70455322913347 | -0.45467114210032 | -5.81639408837780 |
| H | -3.73278432558067 | 1.09432410899584 | -7.47903564026251 |
| C | -3.78423433609550 | -2.44987166802727 | -1.26107574370434 |
| H | -4.67101468039906 | -1.89618879460359 | -0.92707633607338 |
| H | -3.89265713718612 | -3.35909908618850 | 0.64348711363223 |
| H | -4.15636124472232 | -3.30252326053494 | -1.84475713299561 |
| H | -4.98208751008666 | -7.50792037718298 | 0.39282634144858 |
| C | -3.10776699230889 | -2.93379466611845 | 0.01582220587466 |
| H | -8.00442678188368 | -6.04413044410956 | 2.09475666148775 |
| H | -2.68248689439229 | -2.08628387174401 | 0.56663230550164 |
| H | -5.72581889606980 | -8.07919127828545 | 1.89586853009526 |
| C | -5.82934297919944 | -7.36567345882493 | 1.07252670874939 |
| H | -7.15155332580922 | -4.73792405532744 | 2.93542406813506 |
| C | -7.06094008595522 | -5.77285869416673 | 2.58605771619186 |
| H | -4.63687803724014 | -3.80939877730279 | 4.30655131517270 |
| H | -6.74999341633918 | -7.61206961010910 | 0.52747676147892 |
| C | -1.97149905893467 | -3.97236533848149 | -0.04464453377521 |
| C | -5.89302679787950 | -5.91615298486608 | 1.58791025465428 |
| H | -5.11443238164021 | -3.12662827605010 | 2.74421888177978 |
| C | -4.37189936476582 | -3.75311000818792 | 3.24612130717159 |
| H | -6.93935216048777 | -6.42393643419766 | 3.45786403652098 |
| Si | -4.26457743222823 | -5.45309462031971 | 2.48691004902329 |
| H | -1.00481688646866 | -3.45884482508205 | -0.11576768166050 |
| O | -3.08152480722762 | -5.55421635621582 | 1.25669109549178 |
| N | -1.99571781677993 | -4.62872122121866 | 1.28761536500365 |
| H | -6.23004900637309 | -3.92471575009294 | 0.70949930441690 |
| C | -6.16137039563413 | -4.97492480690144 | 0.39933771653371 |
| H | -3.40953654391445 | -3.24187133563524 | 3.16311345083295 |
| H | 0.15596447776182 | -8.55821698281014 | 4.20570476272382 |
| H | -7.11582806972448 | -5.24193253781894 | -0.07390841845648 |
| H | -0.70468713261176 | -7.11469235799184 | 4.76902362797586 |
| O | -0.83511109015789 | -5.44903617561205 | 1.43288870008892 |
| H | -5.37630832760467 | -5.04740217150635 | -0.36002924677916 |
| C | -0.24577827600154 | -7.58533729845301 | 3.89383623455084 |
| H | -0.15071260927202 | -3.89195704927851 | 4.90730094722515 |
| C | -3.88045283341465 | -6.76380999032476 | 3.78030789216708 |

| | | | |
|---|---|---|---|
| C | -0.79563368830314 | -4.14868486874009 | 4.06083219305213 |
| Si | 0.19463649465990 | -5.00788108681902 | 2.73091826304274 |
| H | -1.66876484125840 | -4.69167959144704 | 4.42674625912646 |
| H | -1.02996844784166 | -7.75668732087550 | 3.15041329791135 |
| H | -3.64701714232848 | -7.72399499122352 | 3.30946983407541 |
| H | -4.72548753722241 | -6.91016497273540 | 4.46249784401322 |
| H | -3.01419206396787 | -6.47525770834570 | 4.38330801579050 |
| C | 0.87964527737720 | -6.71042241288865 | 3.31629188579776 |
| H | 1.49932267625475 | -5.94484744509976 | 5.28049696356394 |
| C | 1.93468112819897 | -6.44896550201010 | 4.41030848557606 |
| H | -1.12874122614873 | -3.20623754030724 | 3.61234181783867 |
| H | 2.36530782966247 | -7.40040732113767 | 4.75003080488145 |
| C | 1.56946009367389 | -3.87808567930230 | 2.19915488924004 |
| C | 1.54883827133528 | -7.46914133292499 | 2.15531869824013 |
| H | 1.97906109517275 | -8.41012333092212 | 2.52371486649187 |
| H | 1.95658844672398 | -3.32714145776694 | 3.06140163595055 |
| H | 1.16829394356305 | -3.12740449472322 | 1.50885689074909 |
| H | 2.74602204478512 | -5.81922153296456 | 4.03110727285091 |
| H | 2.35597573904081 | -6.88459241523605 | 1.70016787371417 |
| H | 2.40159163438822 | -4.37908389933080 | 1.70171351402559 |
| H | 0.81942369020422 | -7.71354111134332 | 1.37611330833935 |
| H | 1.08328910491483 | -5.80687676409762 | -0.14859929196089 |
| H | -1.38227258417207 | -7.74089424888984 | -2.21189649220886 |
| C | 1.67501518302967 | -5.32762550225928 | -0.92860976390519 |
| H | 2.72697340872604 | -5.59097307173379 | -0.77977741226349 |
| H | 1.56860890525436 | -4.24217348746978 | -0.80453635436454 |
| C | -0.40558375069547 | -8.14901347385430 | -1.93046801556555 |
| H | -0.47487259854401 | -9.24133199501344 | -2.00733739904808 |
| H | -0.22026575013875 | -7.90174209195968 | -0.87898620908490 |
| H | 2.86261028746056 | -8.07216460824242 | -3.12742974539678 |
| H | -3.01515545101514 | -3.78242642156080 | -5.21028212396452 |
| Si | 1.10090205208289 | -5.78468917910786 | -2.61129634354620 |
| C | 2.02497886333670 | -8.38670613301116 | -2.49543550030278 |
| C | 0.71593555501924 | -7.64151067519976 | -2.85527721551412 |
| C | -2.08485360862874 | -4.04906792203389 | -4.71289004826306 |
| C | -2.07934062141329 | -5.10827873708236 | -1.13754753429438 |
| C | -1.60086875229586 | -4.78582032149077 | -2.49362446715988 |
| O | -2.49479511493455 | -4.37088712056530 | -3.34115855113241 |
| O | -0.41640686088860 | -4.93583185935716 | -2.93193980487096 |
| H | 2.30649436047809 | -8.22517214209820 | -1.44861756147681 |
| H | -1.38780999204343 | -3.20939078246527 | -4.69549726980258 |
| H | -3.12211426374331 | -5.42881989678401 | -1.20355100973609 |
| H | -1.46733535330681 | -5.92715930311860 | -0.74840074487484 |
| H | 1.88036719821835 | -9.46376567450810 | -2.64477057775112 |
| H | -1.62311262112147 | -4.92890060899573 | -5.16571176901960 |
| C | 0.33978564430830 | -7.95072314042385 | -4.31593929632158 |
| C | 2.15486963656296 | -5.10884183804643 | -3.98795385972102 |
| H | 1.14662755648400 | -7.69002095117333 | -5.00631633771933 |
| H | 0.13496378163699 | -9.02283079630239 | -4.42388164946541 |
| H | 3.15552439931401 | -5.55458905580785 | -3.97648100637092 |

H 1.71153044774995 -5.27938031372167 -4.97378385220951
H -0.56137317887087 -7.40775030761376 -4.62079168463869
H 2.26899033944106 -4.02884859574104 -3.84601064197617
548
RC-2 ($r^2$SCAN-3c/xTB)
P 8.21368592759115 0.84882174251346 1.03452502909644
O 9.59162519630138 0.46013454211154 0.35920359611402
O 8.64357179479899 1.90187070018336 2.20278844557147
C 10.78284532319079 0.64641997950952 1.05951026037082
C 11.53924450575018 -0.48242853993510 1.45272316773456
C 12.77726070617354 -0.27584436808274 2.01579851606010
C 13.27670089502512 1.01243005783064 2.27453461963286
C 14.54239060583783 1.20509870158062 2.87019120392454
C 14.97802322441745 2.45773572620326 3.19788671540520
C 14.15124615083550 3.56731721499523 2.96498573812993
C 12.92708029894153 3.41393743358176 2.37393970724133
C 12.46688666734419 2.13904240003361 1.97595853586687
C 11.21837040488579 1.92921933667166 1.32159203869760
C 10.34022123886641 3.08147475860343 1.02831730941378
C 10.76788843583485 4.17978096950699 0.23465712979198
C 11.97033324801093 4.16623110252751 -0.50559065390779
C 12.33125716101952 5.23916856911824 -1.27400810397753
C 11.52080663163110 6.38405236751865 -1.32376677422687
C 10.34564419299859 6.42250514146125 -0.62692958918336
C 9.92582833596986 5.31784606707012 0.14564986694665
C 8.70913649027401 5.33123363552537 0.85046494251920
C 8.25841443511058 4.24515144262022 1.56914741390112
C 9.09033413387293 3.09621380121686 1.61031076363580
H 13.38173805658757 -1.12997435163005 2.28639305108510
H 15.15359714604517 0.33685256246559 3.07008798386317
H 15.94694055552286 2.60024169120805 3.65264128289114
H 14.48619805264835 4.55030217741898 3.26126838073600
H 12.29800009533320 4.27617767853101 2.21713721422041
H 12.60307647339731 3.29319040849535 -0.46356196501428
H 13.24917187412637 5.21402107596170 -1.84317701088563
H 11.83412335746292 7.23050720983475 -1.91719380867752
H 9.71145912684894 7.29623220798037 -0.65957134491103
H 8.11691023399236 6.23444683673441 0.82171514143152
N 7.35521736625388 1.72057570731455 0.01738120539449
P 5.88214563424575 1.39065465187078 -0.47398045252942
O 5.90300327914796 1.58874799871324 -2.08084523842945
O 4.90195414820850 2.51627577931444 0.06457218473885
C 6.42780008672526 2.85666789566805 -2.37862848721149
C 7.77528977052570 3.00616785269787 -2.78218907424393
C 8.25927147486510 4.28527476204948 -2.93971362753407
C 7.46327137064570 5.42167498183647 -2.70817125806157
C 7.97657465314255 6.72709060483901 -2.86836878842027
C 7.19514619954829 7.82242733313066 -2.62800271457063
C 5.86324574824183 7.65842911002465 -2.21764610926825
C 5.32906462630248 6.40648126354271 -2.07862918263974

| | | | |
|---|---|---|---|
| C | 6.10497441845962 | 5.25538047564257 | -2.33272456073417 |
| C | 5.59647309796312 | 3.93440745114841 | -2.19166190432379 |
| C | 4.17483615206189 | 3.70002522763709 | -1.85309862848360 |
| C | 3.16698578886687 | 4.15410089851469 | -2.74563689395519 |
| C | 3.44544705653328 | 4.69141510963731 | -4.01990348762963 |
| C | 2.42792641112738 | 5.08180283661555 | -4.84695621806092 |
| C | 1.08985261498140 | 4.95801977206532 | -4.44274657727765 |
| C | 0.79036643670577 | 4.43251301410047 | -3.21784254888870 |
| C | 1.81787538458980 | 4.01272519058324 | -2.34614450534131 |
| C | 1.53461719984175 | 3.48557486047512 | -1.07674652237534 |
| C | 2.49389155680177 | 2.98490486355573 | -0.21745659152166 |
| C | 3.84397085937780 | 3.03984995809362 | -0.68729099067289 |
| H | 9.27995063820057 | 4.43004143644402 | -3.26409327994405 |
| H | 9.00306908950756 | 6.83826467844518 | -3.18530497661190 |
| H | 7.59469114233241 | 8.81833208051251 | -2.75114615134821 |
| H | 5.25693954932113 | 8.53003518211324 | -2.01865231278872 |
| H | 4.30139531219421 | 6.28746320903095 | -1.77317236834080 |
| H | 4.47108273683402 | 4.77492593948281 | -4.34592533377280 |
| H | 2.65229291824008 | 5.48492706288637 | -5.82343659569155 |
| H | 0.30137803118242 | 5.27517462776813 | -5.10846891513315 |
| H | -0.23487656211120 | 4.31519150714435 | -2.90152499313620 |
| H | 0.49723964245041 | 3.49339284330760 | -0.79016970280701 |
| N | 7.65831350249928 | -0.45549570021073 | 1.82469589480608 |
| N | 5.22484780255321 | -0.06667060556875 | -0.22458875843289 |
| S | 6.36493745444450 | -0.16649438126265 | 2.62507470748914 |
| O | 5.70821250307402 | 1.07637345340619 | 2.34902637412856 |
| O | 5.52239710529635 | -1.31379769486817 | 2.86926233477757 |
| S | 6.15900272696953 | -1.23656472518502 | -0.63941591023048 |
| O | 7.53166798731992 | -0.92531048368741 | -0.91240718876730 |
| O | 5.84826634008490 | -2.45344014040286 | 0.06622982901446 |
| C | 5.48921843025096 | -1.73210017858482 | -2.37243982794082 |
| C | 7.00505808561587 | 0.03837488016232 | 4.43069234968828 |
| F | 4.20990463408473 | -2.11502412325256 | -2.27585952449843 |
| F | 5.53040973339803 | -0.77473636232444 | -3.30262582981717 |
| F | 6.16106672555447 | -2.77481004763491 | -2.87787064320919 |
| F | 7.98297328586510 | 0.92082277546496 | 4.64568178798835 |
| F | 7.46732302839756 | -1.14112204686076 | 4.87778601733792 |
| F | 5.98807480074731 | 0.38065878208383 | 5.24171091207020 |
| C | 1.54572791492286 | 4.81981200527318 | 5.95023886354971 |
| C | 0.85256393439102 | 5.65393127692117 | 5.08698313199400 |
| C | 1.44685952126844 | 6.12800376400561 | 3.92410207382926 |
| C | 2.73823022570121 | 5.74355685282811 | 3.63255325983638 |
| C | 3.43547000909724 | 4.88960670996511 | 4.49848516909445 |
| C | 2.84581360211794 | 4.43382998361360 | 5.66602126866453 |
| H | 1.06479677437940 | 4.46307371293964 | 6.84972049742741 |
| H | -0.16004218980702 | 5.94606378445918 | 5.32668378261307 |
| H | 0.89836376725697 | 6.78180333766112 | 3.26117797088244 |
| H | 3.38622666108859 | 3.78500319254687 | 6.33961965253059 |
| C | 3.60067211399900 | 6.13214132367505 | 2.45665059251800 |
| C | 4.81621678317683 | 5.25609668277017 | 2.66909915177612 |

| | | | |
|---|---|---|---|
| C | 5.92926022166822 | 5.10036037123121 | 1.87297592496868 |
| C | 7.00849803209737 | 4.33754773698291 | 2.33773758148463 |
| C | 6.93533289930687 | 3.75243992834886 | 3.60204275691687 |
| C | 5.79892485379133 | 3.85926775332889 | 4.38229055932321 |
| C | 4.73555858177930 | 4.61051486977444 | 3.91361604598605 |
| H | 5.97428303754851 | 5.55240585834323 | 0.89152515784072 |
| H | 7.79187295636207 | 3.21997119775750 | 3.98219596818328 |
| H | 5.75665306537155 | 3.37590646685689 | 5.34830595297369 |
| C | 2.90463364022997 | 5.98089310350759 | 1.08886791237557 |
| H | 3.56618519012386 | 5.44373748102968 | 0.41069953427687 |
| H | 1.98072777330446 | 5.41085766063854 | 1.17647828411359 |
| C | 3.99937879667805 | 7.63510862630188 | 2.53535973957498 |
| H | 5.00161015990513 | 7.75977252489439 | 2.94436706627333 |
| H | 3.29918753081047 | 8.16032563810870 | 3.18750879752686 |
| C | 2.66857764650238 | 7.40084546712321 | 0.57332233818892 |
| H | 1.75313312047297 | 7.81238208565607 | 1.00189078852349 |
| H | 2.58274593253107 | 7.43711803295836 | -0.51252649703032 |
| C | 3.87483430033697 | 8.16355005407173 | 1.10866682990411 |
| H | 3.74308429081861 | 9.24477209751080 | 1.07962430642487 |
| H | 4.76394536665747 | 7.90349519229874 | 0.53231498678029 |
| C | 8.11509188076021 | 0.87451004034574 | -3.98823433946784 |
| C | 8.62558152137263 | 1.86540475789238 | -3.15062732230728 |
| C | 9.97906382907522 | 1.84906418728106 | -2.80307055824984 |
| C | 10.80185617096333 | 0.88415407760134 | -3.34065434458319 |
| C | 10.28517922919215 | -0.08211027099658 | -4.21607165298102 |
| C | 8.93319468478387 | -0.10591247636067 | -4.51852959884791 |
| H | 7.06973572672543 | 0.89891125823963 | -4.25298890179207 |
| H | 10.36059010770927 | 2.59867699365710 | -2.12409450256158 |
| H | 8.52426420065733 | -0.85820469698836 | -5.17765984927383 |
| C | 12.29457699411005 | 0.71013545596046 | -3.17840120186050 |
| C | 12.57366299818481 | -0.45128680135843 | -4.10429215867443 |
| C | 13.77159887851082 | -1.06152547508622 | -4.41343930257002 |
| C | 13.77706869834871 | -2.12070378774371 | -5.31303720092120 |
| C | 12.60177337072485 | -2.56143381227174 | -5.90080463413342 |
| C | 11.39215493114473 | -1.95670070639226 | -5.59685999382180 |
| C | 11.38277497050631 | -0.90635279808353 | -4.69331796287159 |
| H | 14.69963318574812 | -0.72920948562055 | -3.97495863809464 |
| H | 14.71125478569400 | -2.60358155815555 | -5.56126860688543 |
| H | 12.63027719960383 | -3.38187906077826 | -6.60390656735889 |
| H | 10.47471837995261 | -2.29650845409974 | -6.05487870845564 |
| C | 13.09407721051838 | 1.97984322685942 | -3.52728445470413 |
| H | 13.26778432019784 | 2.05513568703229 | -4.60090677447340 |
| H | 12.51730978182239 | 2.85136995811625 | -3.21536664081119 |
| C | 12.71165252436206 | 0.42834356533198 | -1.72504366313575 |
| H | 12.59029714029706 | -0.62333749346167 | -1.46591801275113 |
| H | 12.06843009080929 | 1.00953593251589 | -1.06744084280895 |
| C | 14.40440979358940 | 1.89942316001536 | -2.73261938116233 |
| H | 14.67675391711737 | 2.87861453113716 | -2.33894251436881 |
| H | 15.22063169425803 | 1.57009710928910 | -3.37561247943339 |
| C | 14.16783802330587 | 0.88645177232499 | -1.59736119366424 |

| | | | |
|---|---|---|---|
| H | 14.34911415858464 | 1.32632775420754 | -0.61554219712490 |
| H | 14.84106342157770 | 0.03485091619645 | -1.69722001853244 |
| C | 0.29328785909013 | 1.08305407601273 | 7.30688147603224 |
| C | -1.07566459408854 | 1.11405616331525 | 7.08876340218620 |
| C | -1.59443461942755 | 1.40888194659470 | 5.83383977427260 |
| C | -0.72524009901631 | 1.66989547095510 | 4.79414621308428 |
| C | 0.66171705804245 | 1.61446493105447 | 5.01259062033433 |
| C | 1.17623303428249 | 1.33082968250439 | 6.26791137432923 |
| H | 0.67439883126718 | 0.86275390566974 | 8.29375360537886 |
| H | -1.74863712770611 | 0.90833222079858 | 7.90813019239122 |
| H | -2.66276744179987 | 1.42791209601029 | 5.68896244434294 |
| H | 2.24428109928235 | 1.30907037196519 | 6.43008916051438 |
| C | -1.01757076540882 | 2.04726385288438 | 3.35868148803073 |
| C | 0.37072022640206 | 2.10757042964496 | 2.76860617556212 |
| C | 0.73807550519627 | 2.39917503278512 | 1.47587237532376 |
| C | 2.09549310302959 | 2.54539643752035 | 1.13659822856158 |
| C | 3.03769039765429 | 2.35443820098940 | 2.15116562855212 |
| C | 2.67876225395169 | 1.99613473266716 | 3.43814126565249 |
| C | 1.33861624257846 | 1.89355102202643 | 3.76024889147683 |
| H | -0.04131324075551 | 2.52234324298018 | 0.73967495772122 |
| H | 4.08258658419884 | 2.47780028260893 | 1.95337708013766 |
| H | 3.45503482236582 | 1.82525381229690 | 4.16885765973426 |
| C | -2.01220981768101 | 1.10835494292279 | 2.65671760254269 |
| H | -1.82118785135722 | 1.10260375089914 | 1.58248385504752 |
| H | -1.90641492730017 | 0.08560973809839 | 3.01603646825039 |
| C | -1.71609252480417 | 3.41814807100517 | 3.25980790764177 |
| H | -1.52008050309904 | 3.84000243961854 | 2.27423499599697 |
| H | -1.33694952327651 | 4.11149102901631 | 4.00954906564972 |
| C | -3.41186564596021 | 1.68276413000781 | 2.93019402698747 |
| H | -3.93445304786316 | 1.09081527531680 | 3.68269324894399 |
| H | -4.01086714185952 | 1.65766615602191 | 2.01872801704978 |
| C | -3.20699585435039 | 3.12588286749525 | 3.42341388009600 |
| H | -3.49017469597092 | 3.21915118035523 | 4.47117106237403 |
| H | -3.80985097001949 | 3.83075087660539 | 2.85383136493167 |
| C | 11.10066976946154 | -2.66433674733045 | 2.47816065255303 |
| C | 11.04801767574900 | -1.86627131835938 | 1.33275644999275 |
| C | 10.62039723307687 | -2.41224521931092 | 0.12316586441348 |
| C | 10.30155510098978 | -3.75267660118029 | 0.07715203261944 |
| C | 10.36229031608285 | -4.54807972619594 | 1.23026788856880 |
| C | 10.74654092144583 | -4.00006533941151 | 2.44326662422127 |
| H | 11.41031378192717 | -2.21730859662118 | 3.41262217016428 |
| H | 10.54607545222397 | -1.78984921074048 | -0.75570944948220 |
| H | 10.77997037792928 | -4.60056192382026 | 3.34124642169116 |
| C | 9.89039608205610 | -4.57802302011643 | -1.11497009140619 |
| C | 9.61556761441640 | -5.91825726786442 | -0.47931157398034 |
| C | 9.13499667319556 | -7.07503562632149 | -1.05709369481757 |
| C | 8.98264943309130 | -8.20921614051652 | -0.26832209357742 |
| C | 9.32565791501001 | -8.19134310838744 | 1.07495012595420 |
| C | 9.81178466387333 | -7.03411340353463 | 1.66417451633323 |
| C | 9.94049121437022 | -5.89534648918206 | 0.88557535255203 |

| | | | |
|---|---|---|---|
| H | 8.87720430155002 | -7.10592816065521 | -2.10612143784656 |
| H | 8.59886917294314 | -9.11801019685090 | -0.70887812974429 |
| H | 9.21054432477075 | -9.08701751379167 | 1.66756813652682 |
| H | 10.08554885092856 | -7.02161727827618 | 2.70927843467416 |
| C | 8.68440370516388 | -3.99774258261509 | -1.88506121724267 |
| H | 7.89330018677848 | -4.74406689634346 | -1.95310787038992 |
| H | 8.27689487543928 | -3.12860403767733 | -1.37249007977222 |
| C | 11.02992728647388 | -4.67798425695857 | -2.16329236947640 |
| H | 11.63160418490715 | -5.57563435809733 | -2.01745617860551 |
| H | 11.68342356689377 | -3.80857562920725 | -2.07185459224252 |
| C | 9.19962334364146 | -3.64755321339244 | -3.28257576993906 |
| H | 9.59600213412100 | -2.63264545474999 | -3.28400649259541 |
| H | 8.41181084367187 | -3.71123449173082 | -4.03184358915149 |
| C | 10.32941265697278 | -4.64477623829254 | -3.51717195294838 |
| H | 11.00320294179668 | -4.34112613413167 | -4.31713768628517 |
| H | 9.92126430171569 | -5.62737238620796 | -3.75910794267516 |
| H | 0.15272416445351 | -1.02922115997099 | -1.31308436885263 |
| H | 3.37537569139702 | 0.41362156802600 | -2.08448815990349 |
| H | 3.95904620388629 | 1.36835838386375 | -4.24485639186476 |
| C | 2.57703531210913 | 0.65900169465404 | -2.77940633334811 |
| C | 2.90891628367086 | 1.21228937665752 | -4.01534632783230 |
| C | 0.83907023081446 | -0.19460076118764 | -1.11951317879777 |
| C | 1.24492687399403 | 0.42161356950632 | -2.43813675169317 |
| C | 1.91338662844842 | 1.53988526879164 | -4.92858804265432 |
| H | 2.17803054610412 | 1.96170546858332 | -5.89181893453282 |
| C | 0.25029669440435 | 0.77230952365642 | -3.36007534045282 |
| C | 0.57807125236315 | 1.32290103545756 | -4.59299971771456 |
| H | 0.25088797048055 | 0.54177657804325 | -0.55587395651461 |
| H | 2.70524310023569 | 0.10239976833647 | -0.03802398304227 |
| C | 1.98081958008000 | -0.69455211788743 | -0.24050854648536 |
| H | 2.54952960512712 | -1.47986434403370 | -0.75129231475662 |
| H | -0.79081628340391 | 0.61055722190289 | -3.08782309549473 |
| H | -0.20726818086640 | 1.57525726518150 | -5.29513757270253 |
| C | 1.47487310398754 | -1.22105348614535 | 1.09385848521265 |
| H | 0.80237819011423 | -0.47853763061636 | 1.54259252586846 |
| H | 2.15598724972803 | -1.44920428671803 | 3.12818629978925 |
| H | 0.90046000033082 | -2.14348653784466 | 0.95207962423714 |
| H | -0.31566151344131 | -4.84483289439350 | 3.88873830989998 |
| C | 2.59528863422362 | -1.43907908062687 | 2.13351839398324 |
| H | -1.41283293680954 | -2.48445999373507 | 6.61730127415364 |
| H | 3.29577982869715 | -0.59655469611441 | 2.09034181757517 |
| H | -0.40413809143682 | -5.22120802198953 | 5.61949562680605 |
| C | -0.61634920842489 | -4.45630520484707 | 4.86720647397735 |
| H | 0.15133141116860 | -1.69824541643880 | 6.86179496886745 |
| C | -0.32848344740983 | -2.64826334203405 | 6.60017979864481 |
| H | 3.38794072537136 | -1.83652348203100 | 6.52709319673091 |
| H | -1.70185554305318 | -4.29851823642914 | 4.84107128140799 |
| C | 3.44383617615613 | -2.64682585771625 | 1.96956486232112 |
| C | 0.08902709803391 | -3.12338836247389 | 5.19192812234049 |
| H | 2.37141657773741 | -0.90831602546454 | 5.40868065485238 |

| | | | |
|---|---|---|---|
| C | 2.97103772664536 | -1.81596205624678 | 5.51633251075139 |
| H | -0.08873822926859 | -3.38734797666016 | 7.37042950702429 |
| Si | 1.98174797278999 | -3.35196726473667 | 5.16776470075925 |
| H | 4.26750947977845 | -2.69151547077905 | 1.26014485238234 |
| O | 2.20549787999780 | -3.85382575332665 | 3.50489470794532 |
| N | 3.29742730210219 | -3.67900231657567 | 2.73784195804862 |
| H | 0.09348621564410 | -1.08624660700550 | 4.35191966259905 |
| C | -0.36022428891681 | -2.06780040369002 | 4.16718186393825 |
| H | 3.81281482355301 | -1.72704853771891 | 4.81969987430545 |
| H | 4.46127324838839 | -8.32311686802774 | 4.90590453659178 |
| H | -1.44990266958343 | -1.94736974129687 | 4.23064627950548 |
| H | 4.73925558656676 | -6.73952300133442 | 5.64751011400564 |
| O | 4.00500648387242 | -4.82127449007234 | 2.60560920813314 |
| H | -0.11659795697702 | -2.36953174000072 | 3.14436094609145 |
| C | 4.49106068893703 | -7.24418942194268 | 4.70947609661482 |
| H | 6.92015060750304 | -3.97527249793136 | 4.99325594607704 |
| C | 2.44105662316222 | -4.76714573807443 | 6.30829352742732 |
| C | 5.88086852247984 | -3.93533414912686 | 4.65466477888613 |
| Si | 5.63558953907947 | -5.07544203396409 | 3.20914438195656 |
| H | 5.22550866176723 | -4.07312359434046 | 5.51491871520890 |
| H | 3.49278888037148 | -6.92463104980129 | 4.39671207134219 |
| H | 1.98829968441879 | -5.71102387142569 | 5.98900451586415 |
| H | 2.08259388334156 | -4.54240425871299 | 7.32066661994177 |
| H | 3.52145916533853 | -4.91741715206528 | 6.37112765545411 |
| C | 5.53632009326537 | -6.94665081613050 | 3.62236354429719 |
| H | 7.21248484201516 | -6.83184660067624 | 5.03898527729189 |
| C | 6.93167638165091 | -7.37226244703892 | 4.12826920586679 |
| H | 5.73755878513145 | -2.92895786717729 | 4.23883712106503 |
| H | 6.92589217890313 | -8.44503805794501 | 4.35839095566119 |
| C | 6.91240034396404 | -4.71573737083793 | 1.92453806755952 |
| C | 5.19304794191474 | -7.76509740702968 | 2.36208462615309 |
| H | 5.17340346333064 | -8.83314405164616 | 2.61390932725172 |
| H | 7.85344326853100 | -4.45425267703501 | 2.41630163560624 |
| H | 6.60477697251139 | -3.83226750183335 | 1.35390547416861 |
| H | 7.69937977025681 | -7.19023841902887 | 3.36933462239011 |
| H | 5.93702956250210 | -7.62042283882450 | 1.57154224675240 |
| H | 7.09545665675585 | -5.53079952033831 | 1.22238223724799 |
| H | 4.20978511476987 | -7.49350171979941 | 1.96378810370199 |
| H | 4.96266501288131 | -6.33173578363173 | -0.16797651008894 |
| H | 2.41768538638457 | -7.52795371757131 | -0.35043332398925 |
| C | 5.31434395119124 | -5.91913446116412 | -1.11510469340856 |
| H | 6.22784369489070 | -6.45730328369094 | -1.39041685231734 |
| H | 5.57979332666838 | -4.86994946373822 | -0.93062469213172 |
| C | 2.89537821120869 | -8.22966814453928 | -1.04042701865912 |
| H | 2.28884362044036 | -9.14470632724781 | -1.07400508428943 |
| H | 3.87531053426898 | -8.49094885783432 | -0.62573234311801 |
| H | 3.78695019484650 | -8.34808751153338 | -4.39147097329956 |
| H | 0.15149524107294 | -2.54896866160934 | -3.26533902952418 |
| Si | 4.03303342371891 | -5.99853066706214 | -2.46909578138896 |
| C | 3.74571411231079 | -8.67044832396559 | -3.34674345284263 |

| | | | |
|---|---|---|---|
| C | 3.02127018762042 | -7.63705795751661 | -2.45694389427159 |
| C | 1.07010696302241 | -3.12823833758133 | -3.17479523971345 |
| C | 2.19331265863028 | -4.71383779109793 | -0.09854838062970 |
| C | 2.11174912611819 | -4.34854729432950 | -1.39216240478360 |
| O | 1.06787275385492 | -3.59417079101108 | -1.80960223563195 |
| O | 2.96028080094166 | -4.68703671597436 | -2.36663788382826 |
| H | 4.77202859110505 | -8.84993162053984 | -3.00539652716583 |
| H | 1.93479569551870 | -2.48607208255179 | -3.36798270261364 |
| H | 1.43666498173232 | -4.39012517334688 | 0.60019912366329 |
| H | 3.04145940929789 | -5.27115048081988 | 0.26518610012555 |
| H | 3.21034108244662 | -9.62785111360505 | -3.30709684325629 |
| H | 1.06853933853671 | -3.97168141088246 | -3.87239393680617 |
| C | 1.61269633768867 | -7.38442076964654 | -3.02869762704692 |
| C | 4.82586703327799 | -5.69816847099983 | -4.13793196023013 |
| H | 1.65696532566046 | -6.93793036295221 | -4.02856428696060 |
| H | 1.06744338872120 | -8.33388418706347 | -3.10810722718845 |
| H | 5.57998442530200 | -6.45582065851110 | -4.37207354012478 |
| H | 4.08853759838764 | -5.68016028426521 | -4.94714462796633 |
| H | 1.03268939865694 | -6.71510138458752 | -2.38502158279954 |
| H | 5.32503280035980 | -4.72333054227540 | -4.10643617424038 |
| P | -8.19930962043712 | -0.94359863936284 | -1.39844701529507 |
| N | -8.70950049588158 | -1.01016451883712 | -2.91173666671502 |
| S | -10.20060418805463 | -1.39196762970643 | -3.22299420468401 |
| O | -11.20420684184429 | -0.57312923637914 | -2.60423831059371 |
| O | -10.48341918859885 | -2.79976112842479 | -3.28515759160497 |
| C | -10.22967787868824 | -0.84721663490351 | -5.03361467653676 |
| F | -9.72529868568761 | 0.37771686388616 | -5.20481751337873 |
| F | -9.53932837773553 | -1.66733858984097 | -5.83028172777652 |
| F | -11.48781362702077 | -0.82163053048889 | -5.49790362424732 |
| N | -7.08902596744782 | 0.20088304874292 | -1.26548648385411 |
| P | -6.65463029122211 | 0.97150376875714 | 0.03880234320559 |
| N | -5.54565262380278 | 2.09431212816228 | -0.37140384680546 |
| S | -4.26821632833925 | 1.59463897898959 | -1.04779279811353 |
| O | -3.48118529181194 | 0.59002631469665 | -0.43235254572661 |
| O | -4.29584787725721 | 1.41559755747899 | -2.53304411068668 |
| Si | -5.22856552245613 | 1.83375041845320 | -3.94555262698529 |
| C | -6.34351962657245 | 3.26491984275314 | -3.41537444853889 |
| H | -7.08282296799093 | 3.50043146841718 | -4.17530889939455 |
| H | -5.79669002641907 | 4.17913881862067 | -3.19676565293847 |
| H | -6.87445667324645 | 2.97244029252234 | -2.51177078490584 |
| C | -6.11873204817126 | 0.25680184998758 | -4.47662169114134 |
| H | -5.43695237923394 | -0.57615467938066 | -4.62220984760232 |
| H | -6.85231904793427 | -0.04670079073273 | -3.73235794869655 |
| H | -6.65028712085644 | 0.41089796930845 | -5.41097551001631 |
| C | -3.87530803227028 | 2.36150063358960 | -5.23574905308508 |
| C | -2.81511451339273 | 3.30358929490464 | -4.64885196368647 |
| H | -2.10649912341778 | 3.59211783817997 | -5.42405664902698 |
| H | -2.26163101956368 | 2.80952324735173 | -3.85414040854988 |
| H | -3.27171988134349 | 4.20708218169452 | -4.25276271546897 |
| C | -3.16286363713855 | 1.12857710188482 | -5.81048528464894 |

| | | | |
|---|---|---|---|
| H | -3.86611480511959 | 0.46945968836281 | -6.31206306521690 |
| H | -2.41600374014637 | 1.43544010426800 | -6.54131084461411 |
| H | -2.66641078169502 | 0.56473662343013 | -5.02567788893536 |
| C | -4.58282538517192 | 3.08526018898373 | -6.38978804997223 |
| H | -5.34309724240442 | 2.44711691904318 | -6.83056414413169 |
| H | -5.06303446996449 | 3.99592793915112 | -6.04393445124496 |
| H | -3.86382547550624 | 3.34628340841792 | -7.16385569171899 |
| C | -3.25807958610659 | 3.18343024393034 | -0.93299651700150 |
| F | -3.27225072271926 | 3.66318252145807 | 0.30607893180859 |
| F | -3.73423985050945 | 4.13258187555931 | -1.73204519191195 |
| F | -1.98191304222762 | 2.97270098182680 | -1.26520943904813 |
| O | -7.85214591128249 | 1.71547619407143 | 0.75072244320727 |
| C | -7.44239100217414 | 2.55897929013210 | 1.80188453757620 |
| C | -7.10685470441899 | 1.98654899324304 | 3.00678435121024 |
| C | -6.59347334440259 | 2.81038557859051 | 4.05464219943324 |
| C | -6.07522708557647 | 2.29263787251344 | 5.26241707910936 |
| C | -5.61992363762209 | 3.12663333667289 | 6.24692658339997 |
| C | -5.65435178054133 | 4.51927257505224 | 6.07729809336437 |
| C | -6.10113953021285 | 5.04997460000798 | 4.90072259908146 |
| C | -6.55570211522520 | 4.21551400914759 | 3.85582015325737 |
| C | -6.94167501032012 | 4.74910436823010 | 2.61268174968110 |
| C | -7.34508923516014 | 3.94868935554042 | 1.56745371587159 |
| C | -7.62493225638738 | 4.53117176183754 | 0.24853219414718 |
| C | -6.74663896486777 | 5.49936202546742 | -0.24454473825909 |
| C | -6.94511294401495 | 6.08616690305373 | -1.47935985424936 |
| C | -8.02845735687338 | 5.67785751779970 | -2.24123889658269 |
| C | -8.91445902964969 | 4.70384515956625 | -1.75716440711122 |
| C | -8.73147930779709 | 4.14405400240712 | -0.51086624245107 |
| H | -9.42760737922228 | 3.41644219167639 | -0.12059485788410 |
| C | -9.98166556784595 | 4.40209485301930 | -2.77887099491423 |
| C | -9.55026404331285 | 5.26771800973119 | -3.94070371554898 |
| C | -10.08417178407295 | 5.37467992838875 | -5.20869286487477 |
| C | -9.51071255460640 | 6.26340651837125 | -6.10972925073552 |
| C | -8.41463711297195 | 7.03416528674758 | -5.75388893107987 |
| C | -7.85778745186958 | 6.92201741273891 | -4.49050225158396 |
| C | -8.42797773515803 | 6.03632664139772 | -3.59044077465154 |
| H | -6.99602633556266 | 7.51126448943926 | -4.21417673487253 |
| H | -7.98844379804558 | 7.72062529611116 | -6.47101966076860 |
| H | -9.92547730642914 | 6.35324677356463 | -7.10341300387017 |
| H | -10.92676885391031 | 4.77507789097603 | -5.51422220166675 |
| C | -10.10873345663606 | 2.90777585298832 | -3.13223710874055 |
| H | -9.40574125400101 | 2.61809683861102 | -3.91184647123755 |
| H | -9.88739222602728 | 2.30106743192669 | -2.25257953471008 |
| C | -11.56604107820011 | 2.68706209554799 | -3.56855152018804 |
| H | -11.95878534340371 | 1.78655381283157 | -3.09737099397410 |
| H | -11.62717947077436 | 2.53427356591955 | -4.64653537514915 |
| C | -12.34714657579616 | 3.94647090338436 | -3.15223671916650 |
| H | -13.26543985731855 | 3.69721322971174 | -2.61983465480685 |
| H | -12.63522480807507 | 4.52797045362044 | -4.02866229325398 |
| C | -11.39296927656262 | 4.75970620582921 | -2.27698227071957 |

| | | | |
|---|---|---|---|
| H | -11.58497608576893 | 5.83170243297380 | -2.33591401239951 |
| H | -11.47280297750113 | 4.45396262326984 | -1.23447723769436 |
| H | -6.25653698364096 | 6.83163770123373 | -1.84837821055628 |
| H | -5.88171103853704 | 5.77134066483562 | 0.34234953932259 |
| H | -6.90712119841667 | 5.81981710299994 | 2.47708730623438 |
| H | -6.10392867295015 | 6.11820777616163 | 4.74267340609977 |
| H | -5.30745573017084 | 5.16156846420615 | 6.87264777489998 |
| H | -5.22472971972196 | 2.71305279391181 | 7.16299767241468 |
| H | -6.02896914096383 | 1.22417257563312 | 5.40513463757324 |
| C | -7.23120147348505 | 0.52662753520711 | 3.20023495706153 |
| C | -6.60177288419650 | -0.34870807834380 | 2.33985540545209 |
| C | -6.49963657452187 | -1.73028615277213 | 2.63488175989609 |
| C | -7.14769284563538 | -2.21554212157233 | 3.74626357133554 |
| C | -7.88663329810803 | -1.37946118428727 | 4.60083637939597 |
| C | -8.52016741648543 | -1.88452402284227 | 5.75695624506022 |
| C | -9.22888899125581 | -1.06008448335989 | 6.58440713900634 |
| C | -9.33152123147834 | 0.30692536897726 | 6.28777826205637 |
| C | -8.69107741090382 | 0.83096951987088 | 5.19847521772662 |
| C | -7.93496521400499 | 0.01137266554851 | 4.33107723356502 |
| H | -8.75552348213874 | 1.88860838492631 | 4.99820856856046 |
| H | -9.91127453179057 | 0.95065235753169 | 6.93284832795081 |
| H | -9.71280315520362 | -1.45248703794995 | 7.46621293698674 |
| H | -8.43186776431431 | -2.93931234788060 | 5.96897661678595 |
| H | -7.05296544114557 | -3.26165131846022 | 3.99872438180934 |
| C | -5.54814647189772 | -2.61831388182129 | 1.94610292931486 |
| C | -4.21803058264076 | -2.20807501309854 | 1.84888086588061 |
| C | -3.22563757155833 | -3.10296874410793 | 1.49329925889998 |
| C | -3.57745871332341 | -4.41281234767854 | 1.21236935827733 |
| C | -4.92219949090029 | -4.81409062128690 | 1.22978516822964 |
| C | -5.90525852835787 | -3.92498157819250 | 1.60496930379854 |
| H | -6.94207285152264 | -4.22199817277323 | 1.65152010431190 |
| C | -5.04982446823149 | -6.27331201920103 | 0.85408709926726 |
| C | -3.59992109524423 | -6.66512732484175 | 0.67468673931732 |
| C | -3.06330375628350 | -7.89221545401564 | 0.34414430702819 |
| C | -1.68388499489972 | -8.01279015639398 | 0.22916258268182 |
| C | -0.85300234434238 | -6.92327969905872 | 0.44132461032432 |
| C | -1.38366454870239 | -5.68769297487994 | 0.77109211752263 |
| C | -2.75745552479469 | -5.56419846758916 | 0.88353149870908 |
| H | -0.74277607611500 | -4.83416961544884 | 0.92772951504469 |
| H | 0.21671065994840 | -7.03330673765303 | 0.34429761297073 |
| H | -1.25256214413690 | -8.96861977799914 | -0.02982530147384 |
| H | -3.69694525979644 | -8.75022777637681 | 0.17468074041438 |
| C | -5.87321801979826 | -6.49578075948023 | -0.44309599216390 |
| C | -7.09319272821695 | -7.32885554284988 | -0.04595033802074 |
| H | -7.92019465999851 | -6.67405396193031 | 0.23194465766148 |
| H | -7.43617565082333 | -7.96589100881178 | -0.86014076414775 |
| C | -6.62091547344139 | -8.12318999653611 | 1.16777868739295 |
| H | -6.01227745423120 | -8.96916183836260 | 0.84619202043655 |
| H | -7.44743886240709 | -8.50489749251689 | 1.76640632186731 |
| C | -5.76701852253394 | -7.11642302551358 | 1.93506863148978 |

| | | | |
|---|---|---|---|
| H | -5.05072087288118 | -7.59581383378495 | 2.60203135214212 |
| H | -6.40895775024958 | -6.46744949483844 | 2.53286612073105 |
| H | -5.26563951628255 | -7.03763446936719 | -1.16951615802024 |
| H | -6.16531191807629 | -5.54833359815556 | -0.89241066620380 |
| H | -2.19537824715349 | -2.78451175900845 | 1.45344362655303 |
| H | -3.95769971874373 | -1.18843635622766 | 2.08680648008247 |
| O | -5.95106570402880 | 0.10970712062472 | 1.18794290625032 |
| O | -9.45736022457261 | -0.76502589534998 | -0.43648619911730 |
| C | -9.78746165415042 | -1.46590365016146 | 0.72083475841047 |
| C | -9.61409618437084 | -2.83013061675771 | 0.84561680279500 |
| C | -9.85409677013532 | -3.44247459690718 | 2.11542147709644 |
| C | -9.51353929292494 | -4.78572393649909 | 2.38515601406123 |
| C | -9.80047112990770 | -5.35957893665136 | 3.59340397047932 |
| C | -10.46811639546454 | -4.62837657944291 | 4.58665949214395 |
| C | -10.78294008175190 | -3.31620007840877 | 4.36988432209545 |
| C | -10.45286182150631 | -2.68450406367141 | 3.15168792683817 |
| C | -10.75421182229606 | -1.33140771014282 | 2.91768434720383 |
| C | -10.41952434732286 | -0.70370400497861 | 1.74120795551242 |
| C | -10.89560405159643 | 0.67374178893208 | 1.52020221230670 |
| C | -11.58458455964046 | 0.99539462196937 | 0.34989708091137 |
| C | -12.22916949926657 | 2.21323493231019 | 0.21932866371249 |
| C | -12.17022610571047 | 3.12846074393656 | 1.25817038712182 |
| C | -11.44496760451593 | 2.83037363531976 | 2.42106230682210 |
| C | -10.82158252478364 | 1.61044586727883 | 2.55558878635366 |
| H | -10.28472550003409 | 1.36039539976031 | 3.45703085527278 |
| C | -11.54412391086192 | 3.95874042171015 | 3.42163797771788 |
| C | -12.39503272295873 | 4.95586098927417 | 2.66623071954503 |
| C | -12.81298245636473 | 6.21325931909370 | 3.04966989710691 |
| C | -13.62219598490367 | 6.94605217637182 | 2.19067673196974 |
| C | -14.00875923142442 | 6.42917901181574 | 0.96407142763594 |
| C | -13.58202276370801 | 5.17387428535303 | 0.56214856506988 |
| C | -12.76801035222096 | 4.44418831364589 | 1.41344049429191 |
| H | -13.88282679594760 | 4.77404808820046 | -0.39465839594104 |
| H | -14.64540083096658 | 7.01198932457880 | 0.31405998428422 |
| H | -13.95713643456854 | 7.93044449070141 | 2.48463359665256 |
| H | -12.51939089601132 | 6.63582249605459 | 3.99871052050506 |
| C | -10.17812296448025 | 4.54303651704638 | 3.85608911138666 |
| H | -10.04785234662990 | 5.53439636380590 | 3.42172516030104 |
| H | -9.37030612016625 | 3.91523403675036 | 3.48463877113264 |
| C | -10.17297693292046 | 4.60539940978682 | 5.39170136240953 |
| H | -9.75402175103367 | 5.54292435692015 | 5.75536040094276 |
| H | -9.56801252261293 | 3.79558145809654 | 5.80186401244029 |
| C | -11.63187820283553 | 4.42613730650502 | 5.81203253331359 |
| H | -12.15212070205867 | 5.38366669644143 | 5.80652874249594 |
| H | -11.72550700541768 | 3.99982259129564 | 6.81074586006634 |
| C | -12.20970922114521 | 3.50681100074145 | 4.74086612994627 |
| H | -13.29650601057572 | 3.56043968737469 | 4.67934607446154 |
| H | -11.92748528511522 | 2.47278512621214 | 4.94682566078597 |
| H | -12.79234587722342 | 2.42256385061799 | -0.67642746921767 |
| H | -11.64598872331342 | 0.27973765729113 | -0.45532676166460 |

| | | | |
|---|---|---|---|
| H | -11.28932727752853 | -0.78331398024355 | 3.67925205262320 |
| H | -11.28512254011297 | -2.73495316907494 | 5.12888475635121 |
| H | -10.72290256263309 | -5.10663447107116 | 5.52068743391998 |
| H | -9.52359958186327 | -6.38636622826698 | 3.78184152102990 |
| H | -9.02038739155381 | -5.36820804332988 | 1.62230152870810 |
| C | -9.32423649527158 | -3.69508272878691 | -0.31794871721655 |
| C | -8.27301303355202 | -3.43582438351008 | -1.16483356931877 |
| C | -8.00498944027283 | -4.21723706547874 | -2.30781084024962 |
| C | -8.76115256871514 | -5.34699414523583 | -2.50693233079101 |
| C | -9.85858339111560 | -5.65551315243845 | -1.68524363931806 |
| C | -10.67626379372882 | -6.77411446148028 | -1.95524625478691 |
| C | -11.81792795687325 | -6.99735921057230 | -1.23948715016933 |
| C | -12.20125000466884 | -6.08991475625617 | -0.24122254823086 |
| C | -11.41374193229662 | -5.01339517704775 | 0.06291971849334 |
| C | -10.19574502954089 | -4.78811451707805 | -0.61403368604226 |
| H | -11.74187757731291 | -4.31508880640347 | 0.81701616950900 |
| H | -13.13281275144886 | -6.24026869100803 | 0.28434998172733 |
| H | -12.44365001236923 | -7.85097712014332 | -1.45428776155318 |
| H | -10.38982291000860 | -7.43790589444365 | -2.75822954135904 |
| H | -8.54786723058780 | -5.98393908706204 | -3.35306783387323 |
| C | -7.03510348856052 | -3.79788902575045 | -3.33364612654176 |
| C | -7.51554841783723 | -3.71775573785804 | -4.64167015016702 |
| C | -6.69301800463638 | -3.33257571439589 | -5.68224141024113 |
| C | -5.36113404027187 | -3.06699880953252 | -5.40949034784051 |
| C | -4.85974504552368 | -3.17123817002042 | -4.10097387305324 |
| C | -5.69837627145709 | -3.50856827317061 | -3.05808028285921 |
| H | -5.33546283847960 | -3.56543250335977 | -2.04381138379390 |
| C | -3.37206878093522 | -2.90159821958010 | -4.09262095612539 |
| C | -3.11264449987643 | -2.52679875532988 | -5.53054318757272 |
| C | -1.93251747863193 | -2.13147959680675 | -6.12662013141305 |
| C | -1.93159478694245 | -1.85124263204446 | -7.48672610770871 |
| C | -3.09042864396441 | -1.97095365001297 | -8.23903043483998 |
| C | -4.27850805278915 | -2.37277741311939 | -7.64958200975986 |
| C | -4.28370837381013 | -2.64986608061529 | -6.29186884075210 |
| H | -5.18094790189712 | -2.46732759094586 | -8.23500011625760 |
| H | -3.06567710678456 | -1.74944826636428 | -9.29606411408732 |
| H | -1.01456383722149 | -1.53742032057147 | -7.96329471667180 |
| H | -1.01885879277293 | -2.03422972588589 | -5.55677034998264 |
| C | -2.91652207260047 | -1.84742293764062 | -3.07197028055634 |
| H | -1.99778429518843 | -1.38556063872614 | -3.43873172913764 |
| H | -3.65861112240338 | -1.06128176198846 | -2.93292594703661 |
| C | -2.64580472830264 | -2.60893633035669 | -1.76964914540438 |
| H | -3.47927824366302 | -2.47673880067462 | -1.08121642905482 |
| H | -1.75830743285707 | -2.22102430121453 | -1.27129482482891 |
| C | -2.46568381015211 | -4.08896442461745 | -2.15241584545885 |
| H | -3.24225666377732 | -4.70103348464810 | -1.69510857979762 |
| C | -2.57234671504179 | -4.15428634945056 | -3.67738979615325 |
| H | -1.58250412169711 | -4.10689748657855 | -4.13199567605580 |
| H | -3.05832446025440 | -5.06758537247614 | -4.02041538557113 |
| H | -1.50371696024328 | -4.47140625106897 | -1.81292286433055 |

| | | | |
|---|---|---|---|
| H | -7.08576893688651 | -3.24602701313975 | -6.68384122457935 |
| H | -8.55925572425262 | -3.92485120497916 | -4.82258839483571 |
| O | -7.48839931157170 | -2.29713685885894 | -0.92559884915395 |

548

TS-2 (r$^2$SCAN-3c/xTB)

| | | | |
|---|---|---|---|
| P | 8.20845151959862 | 0.89243648695224 | 0.93574435984346 |
| O | 9.56093860245185 | 0.45852681994302 | 0.23444754155842 |
| O | 8.70124376463122 | 1.90524265818047 | 2.11717182075531 |
| C | 10.77364236921074 | 0.60021541417996 | 0.90644625741046 |
| C | 11.50877626698156 | -0.55628564509991 | 1.26073421009930 |
| C | 12.76693591199450 | -0.39203384339428 | 1.79291632972528 |
| C | 13.30837574934029 | 0.87716357797640 | 2.06052456890427 |
| C | 14.59392782575014 | 1.02477506166051 | 2.62577332130299 |
| C | 15.07244393287401 | 2.25921224850147 | 2.96257298344570 |
| C | 14.27120869113425 | 3.39486446924509 | 2.76939506699357 |
| C | 13.02873965499439 | 3.28519089634144 | 2.20751834819825 |
| C | 12.52314361694006 | 2.03032832962727 | 1.80106772403058 |
| C | 11.25339925380371 | 1.86613828322708 | 1.17526973416685 |
| C | 10.40487460068868 | 3.04952800601287 | 0.91957842609061 |
| C | 10.84626388345409 | 4.14504628714897 | 0.12921572746256 |
| C | 12.03017791866754 | 4.10570622608123 | -0.63939192475975 |
| C | 12.40345305123168 | 5.17590102155544 | -1.40584558217026 |
| C | 11.62457196062038 | 6.34327160515906 | -1.42636594521606 |
| C | 10.46791304350341 | 6.40761047801683 | -0.70100738883443 |
| C | 10.03579942248761 | 5.30796654169006 | 0.07179828053233 |
| C | 8.83764423152772 | 5.34987730102306 | 0.80702127432563 |
| C | 8.37480283495915 | 4.27050476639751 | 1.52700590091388 |
| C | 9.17128841123725 | 3.09546577957998 | 1.53361916583747 |
| H | 13.35594898511496 | -1.26644804628528 | 2.03089771648577 |
| H | 15.18568070469947 | 0.13679882804872 | 2.79526100872713 |
| H | 16.05632751536756 | 2.36740838971867 | 3.39409611005007 |
| H | 14.64085015354709 | 4.36308173954603 | 3.07301512022775 |
| H | 12.42006755764050 | 4.16685628743929 | 2.08082030117569 |
| H | 12.63896559083518 | 3.21505007191760 | -0.62088204428746 |
| H | 13.30677381939991 | 5.13082044822522 | -1.99676319245301 |
| H | 11.94733786026536 | 7.18622033774667 | -2.01976273822615 |
| H | 9.85787709411532 | 7.29895326734639 | -0.70997632610877 |
| H | 8.26999545451786 | 6.26934638799739 | 0.80129074852172 |
| N | 7.36638544931397 | 1.80950541057862 | -0.05697450682118 |
| P | 5.86926823775112 | 1.51866637504747 | -0.50153323327074 |
| O | 5.85032723240445 | 1.73664859150125 | -2.10750378876617 |
| O | 4.92235834624052 | 2.65862334680057 | 0.06788107889934 |
| C | 6.39055678748966 | 2.99772710153421 | -2.40202891034590 |
| C | 7.73108950337032 | 3.12587559730624 | -2.83541106832454 |
| C | 8.23927499790173 | 4.39689658096856 | -2.97999927849036 |
| C | 7.47164143296116 | 5.54503581539025 | -2.71164864662358 |
| C | 8.00931512572670 | 6.84248284085044 | -2.85561242877385 |
| C | 7.25517618134444 | 7.94910987045575 | -2.58150111501142 |
| C | 5.92593788310118 | 7.80540047921046 | -2.15509963801559 |
| C | 5.36778612311420 | 6.56236563317113 | -2.03175515430479 |

| | | | |
|---|---|---|---|
| C | 6.11653576394665 | 5.40039701293890 | -2.31554801819855 |
| C | 5.58414592765567 | 4.08854558802687 | -2.18271668037060 |
| C | 4.16575468339048 | 3.86816988983059 | -1.82014948341269 |
| C | 3.14539448473023 | 4.33703959762939 | -2.69050016614580 |
| C | 3.40628683438245 | 4.89771159658002 | -3.95848160009004 |
| C | 2.37740104717262 | 5.29161313847251 | -4.76969675887928 |
| C | 1.04442812235182 | 5.14801131950383 | -4.35537288506235 |
| C | 0.76185419410953 | 4.60331068226396 | -3.13481993191840 |
| C | 1.80127515838156 | 4.18036704573026 | -2.27896573226786 |
| C | 1.53458357621280 | 3.62431674014781 | -1.01826522106528 |
| C | 2.50613479767609 | 3.10907523406706 | -0.18219108622287 |
| C | 3.85132531376126 | 3.18694671220433 | -0.66174498638606 |
| H | 9.25612960761075 | 4.52640312167946 | -3.32252340468923 |
| H | 9.03260386005319 | 6.93862712075680 | -3.18766037686394 |
| H | 7.67413048057784 | 8.93861690261348 | -2.69130305000093 |
| H | 5.34094798125477 | 8.68552086918409 | -1.93117763647977 |
| H | 4.34167801397436 | 6.45803211601348 | -1.71598091505702 |
| H | 4.42782299330892 | 4.99531897284215 | -4.29341467511905 |
| H | 2.58869617544435 | 5.71149442372370 | -5.74208576328885 |
| H | 0.24663127978227 | 5.46468623390527 | -5.01016911862569 |
| H | -0.25954794499066 | 4.47027282053632 | -2.81199506911441 |
| H | 0.50075557073211 | 3.61561485098547 | -0.71857212876699 |
| N | 7.59608088462020 | -0.39970951299009 | 1.69834826837250 |
| N | 5.18191573013886 | 0.07668671262344 | -0.24573589375046 |
| S | 6.32575386609163 | -0.05038278145311 | 2.51555558597753 |
| O | 5.79538543120149 | 1.26733464489007 | 2.32876495377794 |
| O | 5.38028277469394 | -1.12119853445472 | 2.70036411162383 |
| S | 6.06840317934109 | -1.11604967031347 | -0.70137400317534 |
| O | 7.44085941540388 | -0.83967614329151 | -1.01410627907977 |
| O | 5.73592434849159 | -2.34265023104589 | -0.02626747897321 |
| C | 5.36349171389946 | -1.54837252722051 | -2.44250908169752 |
| C | 7.03626443637819 | -0.01335422318986 | 4.30910531054417 |
| F | 4.09978472943514 | -1.98917265468380 | -2.35595859007545 |
| F | 5.35711123071285 | -0.55442930053103 | -3.33304130605286 |
| F | 6.06482645278387 | -2.54240986136313 | -3.00519291907214 |
| F | 7.85938595660530 | 0.99240662717178 | 4.61463975438094 |
| F | 7.72909816055719 | -1.14079482771495 | 4.54310392540496 |
| F | 6.04228621201433 | 0.02272434655288 | 5.21807044453952 |
| C | 1.69877186038701 | 4.70984557440395 | 5.96850955396056 |
| C | 0.98637316559431 | 5.54438753839056 | 5.12156897811198 |
| C | 1.57562597188567 | 6.06977266970337 | 3.97829703726063 |
| C | 2.87974695140483 | 5.73165048325978 | 3.68663644173717 |
| C | 3.59623246978965 | 4.87527384127376 | 4.53424261898454 |
| C | 3.01272005104188 | 4.37244665593711 | 5.68557344736903 |
| H | 1.22176528466528 | 4.31307092112997 | 6.85330854509433 |
| H | -0.03835992155065 | 5.79434121744080 | 5.35721828106058 |
| H | 1.01205883179466 | 6.72410981733720 | 3.32858979802694 |
| H | 3.56671162089324 | 3.72064904731521 | 6.34556614761284 |
| C | 3.73648886768122 | 6.16964626483616 | 2.52409950650117 |
| C | 4.96126203120609 | 5.29878199552249 | 2.70495152869577 |

| | | | |
|---|---|---|---|
| C | 6.06225539507913 | 5.15579738310009 | 1.88973758271308 |
| C | 7.14723261790955 | 4.38566392578000 | 2.32772301275511 |
| C | 7.10285686477241 | 3.80046120207140 | 3.59259627053772 |
| C | 5.97833681328974 | 3.89331210597729 | 4.39128931188621 |
| C | 4.90030861426772 | 4.63437021304566 | 3.94116720362806 |
| H | 6.09146481671969 | 5.61701553694872 | 0.91178066666431 |
| H | 7.96852508284819 | 3.27005820134124 | 3.95398016542820 |
| H | 5.95525118512505 | 3.40212206062879 | 5.35412772132916 |
| C | 3.03808742293924 | 6.06900269790833 | 1.15379320543992 |
| H | 3.70023714774669 | 5.56226118980386 | 0.45355314127707 |
| H | 2.11733248860523 | 5.49133709908719 | 1.22111221018957 |
| C | 4.12582499291723 | 7.67147974938836 | 2.66162391057998 |
| H | 5.12741566229240 | 7.78680592955626 | 3.07496880058263 |
| H | 3.42233435107239 | 8.16643306990250 | 3.33373984549432 |
| C | 2.79255255733941 | 7.50693234886688 | 0.69549522519440 |
| H | 1.87670313277678 | 7.89637580567250 | 1.14356829309513 |
| H | 2.70211771371435 | 7.58647779986074 | -0.38761437124216 |
| C | 3.99672801100591 | 8.25397953740308 | 1.25665071730982 |
| H | 3.86062453677807 | 9.33498041906366 | 1.26983702388580 |
| H | 4.88556295999224 | 8.01967303517399 | 0.66877226659717 |
| C | 7.98229162602165 | 1.00829445433978 | -4.08472932755865 |
| C | 8.54213122456369 | 1.97054231892323 | -3.24492439082064 |
| C | 9.90247658698663 | 1.90859832547938 | -2.93116993201947 |
| C | 10.68229818271661 | 0.92718984440119 | -3.50233402359802 |
| C | 10.11570043644491 | -0.00985180262311 | -4.37861568712280 |
| C | 8.75700070749225 | 0.01185212482255 | -4.64897161078608 |
| H | 6.93203578393039 | 1.06759671558795 | -4.32343141864160 |
| H | 10.32265170707828 | 2.63585702003091 | -2.25067365314368 |
| H | 8.30964288829391 | -0.71815179651027 | -5.30821204951051 |
| C | 12.17174757699603 | 0.70241133881892 | -3.37585375704499 |
| C | 12.39376573303172 | -0.44949439342780 | -4.32867381629902 |
| C | 13.56525577698264 | -1.08790871114508 | -4.67943484657794 |
| C | 13.51734144424652 | -2.12959045636871 | -5.59799673272784 |
| C | 12.31524428512521 | -2.52574481606119 | -6.16282871740447 |
| C | 11.13182377818844 | -1.89236924753965 | -5.81706492152488 |
| C | 11.17581671731630 | -0.85891590939055 | -4.89528761045311 |
| H | 14.51336860866982 | -0.79016350101105 | -4.25942403190965 |
| H | 14.43060172313479 | -2.63403228373510 | -5.87908745655102 |
| H | 12.30223103870193 | -3.33337478296630 | -6.88108194434099 |
| H | 10.19351621456225 | -2.19725316601556 | -6.25703431818895 |
| C | 13.00706099080069 | 1.95112997309766 | -3.71553377945098 |
| H | 13.16509036552800 | 2.04037138929492 | -4.79053804393324 |
| H | 12.46495213037755 | 2.83531556014368 | -3.37825183325625 |
| C | 12.60677715742038 | 0.37706616160573 | -1.93689105538877 |
| H | 12.45200713052814 | -0.67430019905205 | -1.69493638130857 |
| H | 11.99870881481537 | 0.96853883290739 | -1.25548721603426 |
| C | 14.32764511266724 | 1.81319886313892 | -2.94558164914171 |
| H | 14.63421800723568 | 2.77402541955472 | -2.53224800840394 |
| H | 15.12376710926230 | 1.47750303495954 | -3.61014862760342 |
| C | 14.08089092483680 | 0.77926408279297 | -1.83162331313230 |

| | | | |
|---|---|---|---|
| H | 14.30071225229106 | 1.18560432356246 | -0.84325097660520 |
| H | 14.71998143041425 | -0.09303770193549 | -1.97003777537331 |
| C | 0.38340689027301 | 0.82542549394794 | 7.25689082912884 |
| C | -0.98444665115676 | 0.78766853252777 | 7.03331015096226 |
| C | -1.51567656454946 | 1.11070640943024 | 5.79028213449702 |
| C | -0.65936397284777 | 1.46487580172088 | 4.76750881761884 |
| C | 0.72810152373536 | 1.47166175207695 | 4.98791832155292 |
| C | 1.25413754171666 | 1.16644315871712 | 6.23345595698347 |
| H | 0.77415540418920 | 0.58247563393127 | 8.23456759915590 |
| H | -1.64747352933940 | 0.50750091318717 | 7.83872350572304 |
| H | -2.58310402634192 | 1.07687980087201 | 5.64140591826970 |
| H | 2.32150349388900 | 1.19355042767518 | 6.39732549192312 |
| C | -0.96868767605215 | 1.89328948635061 | 3.35026247709939 |
| C | 0.41590073056713 | 2.03605949741378 | 2.76352347028930 |
| C | 0.77167826874115 | 2.39869135568318 | 1.48547169820805 |
| C | 2.12215424690629 | 2.61554307168808 | 1.15676106766105 |
| C | 3.07122185715405 | 2.43217881946206 | 2.16589605000343 |
| C | 2.72639178849591 | 2.00953312164260 | 3.43680718134018 |
| C | 1.39196381974831 | 1.83078199115783 | 3.74921892804420 |
| H | -0.00958264568422 | 2.51913423571733 | 0.75068395111918 |
| H | 4.10923792188686 | 2.61180768875417 | 1.97450958179583 |
| H | 3.50724025842469 | 1.84608056313835 | 4.16422164543361 |
| C | -1.93865843361655 | 0.95408862136120 | 2.61477319941736 |
| H | -1.75106152974211 | 0.99243737806214 | 1.54070058494437 |
| H | -1.80806223989747 | -0.07869509717563 | 2.93738183538362 |
| C | -1.70900742246981 | 3.24584031004470 | 3.31561538664455 |
| H | -1.53213886710647 | 3.71619680656846 | 2.34862392921114 |
| H | -1.34606521233820 | 3.91709588350171 | 4.09332221644194 |
| C | -3.35388638562419 | 1.47861386956733 | 2.91239142557251 |
| H | -3.86195889362938 | 0.83852852720110 | 3.63512733013227 |
| H | -3.95232555435438 | 1.48095652333819 | 2.00031340599031 |
| C | -3.18869566477775 | 2.90154270260392 | 3.47399626351109 |
| H | -3.46491470890647 | 2.93449833371069 | 4.52724633438475 |
| H | -3.81692490254537 | 3.61481772878384 | 2.94343563979984 |
| C | 11.06950159439332 | -2.75671932850429 | 2.24892130792214 |
| C | 10.98474138997694 | -1.92721519462131 | 1.12752057402946 |
| C | 10.50186212335419 | -2.43643324326001 | -0.07738789003090 |
| C | 10.16071741064067 | -3.77062244086675 | -0.14420328491482 |
| C | 10.25397056766484 | -4.59731794382156 | 0.98447332279293 |
| C | 10.69469883988932 | -4.08626151281352 | 2.19437756920021 |
| H | 11.42084272671767 | -2.33920084188167 | 3.18233789434620 |
| H | 10.40245720366374 | -1.79116721390765 | -0.93703384540317 |
| H | 10.75436715322343 | -4.70898284877705 | 3.07561833523247 |
| C | 9.69862454871561 | -4.55997659199362 | -1.34209948328441 |
| C | 9.41912565574617 | -5.91005360067186 | -0.73020387755313 |
| C | 8.89678351182645 | -7.04250712988478 | -1.32019235740298 |
| C | 8.75749439215942 | -8.19653717876131 | -0.55810166489559 |
| C | 9.15505087648853 | -8.22140662104368 | 0.76986261041059 |
| C | 9.68226777486017 | -7.08879297967659 | 1.37101331168448 |
| C | 9.79664261561568 | -5.92883054246083 | 0.62151584841948 |

| | | | |
|---|---|---|---|
| H | 8.59616387469184 | -7.03894607791888 | -2.35810502669281 |
| H | 8.34226615678910 | -9.08666668773008 | -1.00811991021358 |
| H | 9.05147419185793 | -9.13163023520694 | 1.34168756674187 |
| H | 10.00102779665561 | -7.11487539213746 | 2.40237133049884 |
| C | 8.48587349555817 | -3.94300337162439 | -2.07076980249816 |
| H | 7.67443811217935 | -4.66922243497222 | -2.12277431197405 |
| H | 8.11555998258097 | -3.07001399727022 | -1.53687066803920 |
| C | 10.81137028052861 | -4.65880581690959 | -2.41935002661809 |
| H | 11.40664552489597 | -5.56461169218250 | -2.29991389135096 |
| H | 11.47623035207517 | -3.79757130918143 | -2.33272763782541 |
| C | 8.96961191236386 | -3.58826173786523 | -3.47902077218843 |
| H | 9.38082655972418 | -2.57932216440069 | -3.48181643765149 |
| H | 8.16227494379915 | -3.63241638949297 | -4.20834417254636 |
| C | 10.07757768283328 | -4.59949601315676 | -3.75410129042861 |
| H | 10.73526264043793 | -4.29599711448004 | -4.56739193642577 |
| H | 9.64931293366364 | -5.57325387413024 | -3.99688681943359 |
| H | 0.09522132794745 | -0.94489561566877 | -1.30154213874188 |
| H | 3.33619350689730 | 0.58423406026664 | -1.94675598608312 |
| H | 3.95810620305447 | 1.59286373413338 | -4.07432599095692 |
| C | 2.55545940619982 | 0.80841169996270 | -2.66878285340539 |
| C | 2.90784609545341 | 1.39333368627700 | -3.88436854245508 |
| C | 0.79683258473037 | -0.12960412282450 | -1.08105281605639 |
| C | 1.22335371598874 | 0.51120156638112 | -2.38006928368495 |
| C | 1.93261236413663 | 1.69445918777400 | -4.82791480870228 |
| H | 2.21425763311510 | 2.14213577778610 | -5.77455504169676 |
| C | 0.24846322714731 | 0.83171818330568 | -3.33376049558316 |
| C | 0.59671888888541 | 1.41603739431142 | -4.54537236485777 |
| H | 0.21045287457617 | 0.59975338279575 | -0.50721729809198 |
| H | 2.61867850132733 | 0.11624988049966 | 0.09856640788482 |
| C | 1.91067264792785 | -0.67094592415237 | -0.18737590504375 |
| H | 2.51707911487183 | -1.41184567206525 | -0.72103880285942 |
| H | -0.79393241684045 | 0.62202907927907 | -3.10268095717240 |
| H | -0.17180081907350 | 1.64594262408434 | -5.27300104328485 |
| C | 1.33220729050995 | -1.28880521600025 | 1.07713724881474 |
| H | 0.68736224822327 | -0.54505936849177 | 1.56363298823769 |
| H | 1.85825154716142 | -1.80360573761069 | 3.08613742450496 |
| H | 0.70197703256343 | -2.14742231034686 | 0.82178488497482 |
| H | -0.24245880338442 | -5.26543597589573 | 3.75993570614076 |
| C | 2.36876381351063 | -1.68440889916890 | 2.13316571584337 |
| H | -1.71001630164502 | -3.07759973738937 | 6.45097294335362 |
| H | 3.09518725897206 | -0.86589358735690 | 2.25400360184004 |
| H | -0.39758141764522 | -5.68564658622152 | 5.47485741264764 |
| C | -0.63496802169026 | -4.92621147774860 | 4.72418522045959 |
| H | -0.24375312353112 | -2.15042615228462 | 6.80068022386621 |
| C | -0.61549174386715 | -3.13618593711912 | 6.49698782337062 |
| H | 3.02874747374690 | -1.94859176738563 | 6.61427493075865 |
| H | -1.72715057475463 | -4.86629249771522 | 4.63523627771512 |
| C | 3.25792550029893 | -2.87222648738627 | 1.92480791578059 |
| C | -0.07038942908901 | -3.54506635777682 | 5.11221538522654 |
| H | 1.89495836698335 | -1.13856809752132 | 5.51927358302332 |

| | | | |
|---|---|---|---|
| C | 2.61336229001840 | -1.95893819132447 | 5.60270021198560 |
| H | -0.35109094628098 | -3.86292482851910 | 7.27109297781431 |
| Si | 1.83536374910214 | -3.60305676771695 | 5.20434062751520 |
| H | 4.12346483955134 | -2.80657844245009 | 1.27031351238342 |
| O | 2.23360079569905 | -4.10456273661520 | 3.59040687598883 |
| N | 3.35987636174780 | -3.74526089352406 | 2.91511155624349 |
| H | -0.18595806894511 | -1.49824654663904 | 4.30428716608918 |
| C | -0.54647510112780 | -2.50979944141464 | 4.07732523645419 |
| H | 3.43479853902086 | -1.73703503246696 | 4.91395722818816 |
| H | 5.37483146980049 | -8.02056996469198 | 5.39432675498821 |
| H | -1.64447771477340 | -2.47888690873760 | 4.07091012217434 |
| H | 5.29723666437707 | -6.37141772016279 | 6.03426799524918 |
| O | 4.22850855263257 | -4.79924226699634 | 2.83171663426001 |
| H | -0.21500952817730 | -2.76833666349899 | 3.06662185595807 |
| C | 5.17077227191525 | -6.97523365640740 | 5.13060992243554 |
| H | 6.76689758046103 | -3.39115950453264 | 5.37038002253340 |
| C | 2.36926338631937 | -4.95479534139794 | 6.38961676838073 |
| C | 5.77084506782197 | -3.50815563281184 | 4.93354713610317 |
| Si | 5.83477270294743 | -4.69780580840675 | 3.50587023507749 |
| H | 5.05928367831092 | -3.74029936157781 | 5.72725115916709 |
| H | 4.12875717233313 | -6.90101535360635 | 4.80628677216431 |
| H | 2.01479263825563 | -5.93793812349880 | 6.06362257765348 |
| H | 1.96948238082194 | -4.75967037831375 | 7.39169188743567 |
| H | 3.45800772888093 | -5.00709918448005 | 6.47420499848826 |
| C | 6.13363093538695 | -6.52311881461217 | 4.02036286365262 |
| H | 7.72574854974999 | -5.97467821431239 | 5.43187307373724 |
| C | 7.58342494578287 | -6.60308327473530 | 4.54553115371663 |
| H | 5.51286581983201 | -2.54292468600379 | 4.48112801044434 |
| H | 7.81936021701657 | -7.63878456828036 | 4.82123302154941 |
| C | 7.07552378716782 | -4.12852575300162 | 2.26107940760563 |
| C | 5.98419810380112 | -7.47149505055830 | 2.81537309373977 |
| H | 6.23514188129817 | -8.49539130412070 | 3.12115067191472 |
| H | 7.93667726981107 | -3.68575671228324 | 2.76930773965756 |
| H | 6.63637918405554 | -3.33255228966710 | 1.65125330501529 |
| H | 8.29740599756628 | -6.27863262172525 | 3.78222543685388 |
| H | 6.65024047470841 | -7.19514030378434 | 1.99070227696341 |
| H | 7.42467982519090 | -4.91421609730688 | 1.58899385679248 |
| H | 4.95595983609793 | -7.47326000300261 | 2.43942606329910 |
| H | 5.02192658970549 | -6.02027565281183 | 0.46618362920927 |
| H | 2.43185571701700 | -7.34934650767392 | 0.32825670747440 |
| C | 5.38326826026523 | -5.70916286275984 | -0.51506589548209 |
| H | 6.29257071474359 | -6.27953060037867 | -0.73272101969135 |
| H | 5.65289584763745 | -4.64747207654212 | -0.43714062183007 |
| C | 2.96667893771882 | -8.06026074777222 | -0.30837578212920 |
| H | 2.40643024844596 | -9.00483338718770 | -0.30976978510730 |
| H | 3.94255893700665 | -8.25005792377617 | 0.15161333316332 |
| H | 3.95709622040750 | -8.36107931906739 | -3.61732689567295 |
| H | 0.05206729894830 | -2.76785635202798 | -2.91575664451770 |
| Si | 4.10511812961111 | -5.89712213730709 | -1.84872085227090 |
| C | 3.89925939091518 | -8.61295643422449 | -2.55456819689592 |

| | | | |
|---|---|---|---|
| C | 3.11808542616584 | -7.54669494934125 | -1.75343636027956 |
| C | 1.01403570317457 | -3.25703064151130 | -2.77023641711797 |
| C | 2.15201352095232 | -4.53003489247830 | 0.46010085388458 |
| C | 2.09574739884319 | -4.25157806111357 | -0.87668332415883 |
| O | 1.03873362554850 | -3.58188261172958 | -1.35817203084868 |
| O | 2.99147839765873 | -4.59675572387749 | -1.78311686334526 |
| H | 4.92110004994386 | -8.73484310062765 | -2.17618603939877 |
| H | 1.83125552885588 | -2.57626462173702 | -3.02249332462144 |
| H | 1.27632072384665 | -4.37516317462106 | 1.07372643819275 |
| H | 2.93681236925419 | -5.17617004559547 | 0.82156134647520 |
| H | 3.39266199120880 | -9.58181640398875 | -2.46122224632887 |
| H | 1.08549552354216 | -4.16871274211584 | -3.36983511480124 |
| C | 1.72386862478520 | -7.37164786792531 | -2.38548002812227 |
| C | 4.84354342097983 | -5.66481764880987 | -3.54926981627666 |
| H | 1.79151734322265 | -7.00076589888873 | -3.41425415958775 |
| H | 1.20224927618927 | -8.33690892400458 | -2.41008055312939 |
| H | 5.62660813752206 | -6.40302073159812 | -3.74894887498929 |
| H | 4.09055371730043 | -5.73568996183407 | -4.34103470307454 |
| H | 1.10530950331645 | -6.67153146238707 | -1.81421093906774 |
| H | 5.29853023801717 | -4.67025511154565 | -3.59774863401046 |
| P | -8.19872564151542 | -0.94060482842385 | -1.47357401342240 |
| N | -8.69106599317948 | -0.99167712059653 | -2.99309444769126 |
| S | -10.18525431492484 | -1.33808699858644 | -3.32897815353659 |
| O | -11.18008730317501 | -0.50992990177639 | -2.70863243530638 |
| O | -10.49438656218532 | -2.73868597817795 | -3.41943726184520 |
| C | -10.17721654546231 | -0.76571415916361 | -5.13077710855902 |
| F | -9.65711840186873 | 0.45606727841364 | -5.27558217009233 |
| F | -9.47961464303840 | -1.58245555040829 | -5.92466800803804 |
| F | -11.42579190283630 | -0.72080648957239 | -5.61859944372545 |
| N | -7.06640994966896 | 0.17995089558096 | -1.32283444309366 |
| P | -6.63329433703092 | 0.92628538045707 | -0.00381258634495 |
| N | -5.50622769970490 | 2.04020083862817 | -0.38515112828715 |
| S | -4.22844518066282 | 1.54036364598253 | -1.05978654219455 |
| O | -3.45912573503050 | 0.51361350619528 | -0.45894560624600 |
| O | -4.24567238340221 | 1.39146858234258 | -2.54851487351565 |
| Si | -5.17537245291963 | 1.82812121427144 | -3.95749953293099 |
| C | -6.28617931418549 | 3.25517821073411 | -3.41039637144302 |
| H | -7.01004024259315 | 3.51831661457843 | -4.17599788070012 |
| H | -5.73280564831707 | 4.15695031889478 | -3.15895659024249 |
| H | -6.83481169478088 | 2.94560659923051 | -2.52326726518139 |
| C | -6.06452672300448 | 0.25978845212741 | -4.51585968468744 |
| H | -5.38272768796523 | -0.57119201511040 | -4.67242835929364 |
| H | -6.80181809631358 | -0.05439966813086 | -3.77975123450254 |
| H | -6.59232291390184 | 0.42940401355257 | -5.44969425933778 |
| C | -3.81517026836427 | 2.37041249658220 | -5.23295509063271 |
| C | -2.77178580735965 | 3.32129616325365 | -4.63006848311843 |
| H | -2.06083806801783 | 3.62639742183985 | -5.39666069581323 |
| H | -2.21787600369130 | 2.82900942310805 | -3.83461107730868 |
| H | -3.24383977964394 | 4.21525236420771 | -4.23021325202765 |
| C | -3.08624755507658 | 1.14550456173117 | -5.80431256138931 |

| | | | |
|---|---|---|---|
| H | -3.77828724694185 | 0.48421076685701 | -6.31863391557093 |
| H | -2.33197662939584 | 1.46137289945504 | -6.52347403366576 |
| H | -2.59708330654747 | 0.58105332410060 | -5.01532993193414 |
| C | -4.51714289885964 | 3.09227457830859 | -6.39148285675335 |
| H | -5.26732093571255 | 2.44949856185128 | -6.84269187349014 |
| H | -5.00882258355698 | 3.99728194986976 | -6.04681959115760 |
| H | -3.79257149588116 | 3.36296141046973 | -7.15699081004852 |
| C | -3.20663468211216 | 3.11704666261229 | -0.90224294893936 |
| F | -3.23057710543085 | 3.56794872584872 | 0.34791164297095 |
| F | -3.67158502837825 | 4.08611908938221 | -1.68398969744075 |
| F | -1.92885554980733 | 2.90801261824266 | -1.22771312275383 |
| O | -7.82799407186660 | 1.67765788791517 | 0.70467940123212 |
| C | -7.41795528294070 | 2.50070118480076 | 1.77175337222845 |
| C | -7.10115527491262 | 1.90522475242838 | 2.97040070699193 |
| C | -6.58855967201970 | 2.70541091908333 | 4.03636636317651 |
| C | -6.09015922295213 | 2.16162852346075 | 5.24085460391779 |
| C | -5.63836995250612 | 2.97411443761111 | 6.24474848351352 |
| C | -5.65467974526628 | 4.36958295782263 | 6.09755757000109 |
| C | -6.08035720543381 | 4.92486394030907 | 4.92448231844517 |
| C | -6.53264211803522 | 4.11310903580748 | 3.86091825831141 |
| C | -6.89910597660829 | 4.67107119783174 | 2.62251158303425 |
| C | -7.30153572509251 | 3.89256218217095 | 1.56033093569824 |
| C | -7.56124655885946 | 4.50034662749443 | 0.24862887878033 |
| C | -6.66509433071121 | 5.46449617120575 | -0.21960040074621 |
| C | -6.84284961675509 | 6.07384175643610 | -1.44661652201005 |
| C | -7.92368047992187 | 5.69266626565486 | -2.22593021037143 |
| C | -8.82838380062224 | 4.72388981155064 | -1.76642746818353 |
| C | -8.66559149889028 | 4.14133723201972 | -0.52767270402164 |
| H | -9.37536072070538 | 3.41709775001923 | -0.15612071301039 |
| C | -9.88916598299312 | 4.45424344845313 | -2.80388499237372 |
| C | -9.43035039661061 | 5.32808639730768 | -3.94893570228754 |
| C | -9.94623937587521 | 5.45885501906263 | -5.22213893826658 |
| C | -9.34879652001544 | 6.35094045840302 | -6.10408467030959 |
| C | -8.24699684012024 | 7.10174353420182 | -5.72410095198314 |
| C | -7.70808340803182 | 6.96537031564936 | -4.45530706481913 |
| C | -8.30205513896556 | 6.07611129459961 | -3.57435593409221 |
| H | -6.84182713228489 | 7.53877520821983 | -4.16032106629194 |
| H | -7.80200034161419 | 7.79136795132540 | -6.42662343355915 |
| H | -9.74930519215080 | 6.45938795284492 | -7.10174082819961 |
| H | -10.79276792987750 | 4.87479353663697 | -5.54637761581313 |
| C | -10.04056309809862 | 2.96750396633744 | -3.17855369585907 |
| H | -9.33578011916786 | 2.67538308546218 | -3.95555051981961 |
| H | -9.83891706684269 | 2.34506802550072 | -2.30522596162589 |
| C | -11.49735214544981 | 2.77948921382894 | -3.63167919834961 |
| H | -11.91198493174437 | 1.88167710990065 | -3.17420993969455 |
| H | -11.55059907524045 | 2.63922753688579 | -4.71178611193161 |
| C | -12.25829112057484 | 4.04927034320263 | -3.20963584819142 |
| H | -13.18826532466068 | 3.81247112345519 | -2.69201090836485 |
| H | -12.52312224742087 | 4.64708070315066 | -4.08240995614441 |
| C | -11.29903320676329 | 4.83235150630640 | -2.31274917539901 |

| | | | |
|---|---|---|---|
| H | -11.46980581830226 | 5.90852594610904 | -2.35955439516601 |
| H | -11.39628631645527 | 4.51470883928845 | -1.27535194203131 |
| H | -6.14011223209518 | 6.81526620188654 | -1.79662911937468 |
| H | -5.80254066111797 | 5.71495505362767 | 0.38025079105086 |
| H | -6.85071376721120 | 5.74337396666287 | 2.50477359217177 |
| H | -6.06820460899834 | 5.99551328583643 | 4.78400747381011 |
| H | -5.31033127366922 | 4.99472236202133 | 6.90755046948311 |
| H | -5.25882288509873 | 2.54110644845584 | 7.15843105454860 |
| H | -6.05586594597026 | 1.09044650466145 | 5.36481560639514 |
| C | -7.25177235830713 | 0.44493516913623 | 3.14066426841100 |
| C | -6.62763170395761 | -0.42934708087527 | 2.27514680661496 |
| C | -6.55894895307798 | -1.81722619581354 | 2.54903983363224 |
| C | -7.23255767358605 | -2.30505631447461 | 3.64440816080970 |
| C | -7.96549105130980 | -1.46754488911886 | 4.50238987106808 |
| C | -8.62564633824484 | -1.97737568264256 | 5.64140694473135 |
| C | -9.32854713402266 | -1.15115553786099 | 6.47197321762302 |
| C | -9.39868012069222 | 0.22217426592479 | 6.19553433406431 |
| C | -8.73252228898644 | 0.74960044686307 | 5.12346716022446 |
| C | -7.98139963128288 | -0.07215795988718 | 4.25384280653243 |
| H | -8.77234461132283 | 1.81128442400286 | 4.93879284392042 |
| H | -9.97398606844640 | 0.86772964595272 | 6.84278112621661 |
| H | -9.83265913718213 | -1.54684065342674 | 7.34089892764063 |
| H | -8.56241854377034 | -3.03697048614648 | 5.83789118819440 |
| H | -7.16390879566322 | -3.35671401239348 | 3.88182142922932 |
| C | -5.62283069562051 | -2.72269210924038 | 1.86123107149113 |
| C | -4.27905634545848 | -2.35498242968158 | 1.78525902808723 |
| C | -3.31003934022656 | -3.28149959876793 | 1.44407632458586 |
| C | -3.69745584281121 | -4.58009747957021 | 1.15771626477414 |
| C | -5.05521881337075 | -4.93621334760933 | 1.14854464603669 |
| C | -6.01523483292331 | -4.01587733183370 | 1.50667729462295 |
| H | -7.06170473670456 | -4.27941642548539 | 1.53338326327766 |
| C | -5.22448508393883 | -6.39104813565773 | 0.77356886656481 |
| C | -3.78520670081565 | -6.83615740566568 | 0.64380207252602 |
| C | -3.28685578110839 | -8.08973283332550 | 0.35574354800636 |
| C | -1.91057124987713 | -8.26566674925876 | 0.29194775210175 |
| C | -1.04581286801568 | -7.20329770764247 | 0.50694718710250 |
| C | -1.53792721163303 | -5.93973146951292 | 0.78853213903546 |
| C | -2.90933951010748 | -5.76257041081742 | 0.85843427806408 |
| H | -0.86930352351581 | -5.10758255754971 | 0.94652456937017 |
| H | 0.02054441234492 | -7.36309027782485 | 0.45452980326102 |
| H | -1.50849497993083 | -9.24399918277402 | 0.07301075014249 |
| H | -3.94826051669642 | -8.92597735920616 | 0.18418851890209 |
| C | -6.00841117836273 | -6.58707528530962 | -0.55255063794113 |
| C | -7.27892995061102 | -7.35914458733646 | -0.19388115531547 |
| H | -8.08122174588117 | -6.66404395957325 | 0.05671435832648 |
| H | -7.62638914666294 | -7.98107657091965 | -1.01777714676456 |
| C | -6.88395551836665 | -8.17175079826313 | 1.03513029120990 |
| H | -6.30534879886585 | -9.04592202767968 | 0.73400966312188 |
| H | -7.74646497776167 | -8.51355353615675 | 1.60649374061246 |
| C | -6.01005611560627 | -7.20393027954434 | 1.82973327813058 |

| | | | |
|---|---|---|---|
| H | -5.33562366748517 | -7.71517875210845 | 2.51643911837248 |
| H | -6.64045692770558 | -6.52880127941850 | 2.41046634038944 |
| H | -5.40010284599951 | -7.16439628811050 | -1.25051019768431 |
| H | -6.23923586568757 | -5.63181878637279 | -1.02096881398025 |
| H | -2.26973622608117 | -2.99630396529937 | 1.42219145436070 |
| H | -3.98983431075349 | -1.34433406275689 | 2.02890055712039 |
| O | -5.95433229495896 | 0.03575781825609 | 1.13882823030037 |
| O | -9.46145997176159 | -0.74024655300627 | -0.52212789664381 |
| C | -9.82791393877432 | -1.45246783233775 | 0.61725056640309 |
| C | -9.68766128543749 | -2.82203094020548 | 0.72467631308955 |
| C | -9.96146263177265 | -3.44706201150400 | 1.98155461483598 |
| C | -9.65709029638528 | -4.80187367573780 | 2.23648826788886 |
| C | -9.97593996580484 | -5.38618074171017 | 3.43165463833413 |
| C | -10.64023043075435 | -4.65369382798102 | 4.42618399866678 |
| C | -10.92010443854523 | -3.33125201256951 | 4.22431945939879 |
| C | -10.55764988120733 | -2.69039248839987 | 3.02025429154889 |
| C | -10.82592364811997 | -1.32801470667975 | 2.80102056188469 |
| C | -10.45980107135257 | -0.69163022218559 | 1.63875790544099 |
| C | -10.90661929714023 | 0.69739339609546 | 1.43011242294041 |
| C | -11.57601062783742 | 1.04555142307788 | 0.25606049765753 |
| C | -12.20056301781861 | 2.27474936071218 | 0.13488260802817 |
| C | -12.13954920374485 | 3.17491502159739 | 1.18672785358468 |
| C | -11.43253326651404 | 2.85008664671700 | 2.35371292697713 |
| C | -10.82994707260698 | 1.61875127909308 | 2.47898079433679 |
| H | -10.30826221402932 | 1.34793091814247 | 3.38340435499312 |
| C | -11.52494458370055 | 3.96686379507651 | 3.36778328925095 |
| C | -12.35094130730457 | 4.98731421712129 | 2.61597119690541 |
| C | -12.75347659143259 | 6.24589989983274 | 3.01179397188304 |
| C | -13.54145103655470 | 7.00272150873855 | 2.15390732887689 |
| C | -13.92279159701418 | 6.50799658130235 | 0.91657675620282 |
| C | -13.51179707267004 | 5.25131334867259 | 0.50273824391229 |
| C | -12.71846791129703 | 4.49778510244993 | 1.35280255148938 |
| H | -13.80895864938277 | 4.86877645059587 | -0.46222329285258 |
| H | -14.54301838496585 | 7.10934297616461 | 0.26758032803356 |
| H | -13.86394457028526 | 7.98846191404899 | 2.45708477212684 |
| H | -12.46413656954539 | 6.65089501085586 | 3.96979585459308 |
| C | -10.15428176605512 | 4.52245557236603 | 3.82525010205812 |
| H | -10.00946792224318 | 5.52302024490830 | 3.41734201993860 |
| H | -9.35135596242833 | 3.89464878017862 | 3.44331509944995 |
| C | -10.15943065322170 | 4.54681996024430 | 5.36159395351912 |
| H | -9.71680713742269 | 5.46249223284248 | 5.75193218612098 |
| H | -9.58189576245180 | 3.70961028537802 | 5.75598554013794 |
| C | -11.62632887123705 | 4.39835640885163 | 5.76459948765659 |
| H | -12.12211244827854 | 5.36882119303176 | 5.76769822709900 |
| H | -11.74149212633743 | 3.95977454059895 | 6.75571418395505 |
| C | -12.21430410052662 | 3.50971141832869 | 4.67320159325940 |
| H | -13.29887822590634 | 3.58957855412734 | 4.60173405835313 |
| H | -11.95823126201898 | 2.46631968660019 | 4.86569408341848 |
| H | -12.75044049445838 | 2.50481037256687 | -0.76399102900037 |
| H | -11.63852929878038 | 0.34148910013569 | -0.55932943818315 |

| | | | |
|---|---|---|---|
| H | -11.36054577211336 | -0.77877308158944 | 3.56209606953377 |
| H | -11.41916632159026 | -2.74892910443871 | 4.98455870094791 |
| H | -10.92003206635641 | -5.13911449965752 | 5.34927389301065 |
| H | -9.72669843166846 | -6.42204408958655 | 3.60871412193613 |
| H | -9.16683827111502 | -5.38525184238689 | 1.47254956634882 |
| C | -9.39754808289208 | -3.67700812075641 | -0.44633450083538 |
| C | -8.32465272028165 | -3.43034316300881 | -1.26925159586043 |
| C | -8.05299230342721 | -4.19995962386200 | -2.41935314229238 |
| C | -8.83194751445185 | -5.30809786713763 | -2.65036340804629 |
| C | -9.95217019827705 | -5.60236234277129 | -1.85447480049672 |
| C | -10.79168485659630 | -6.69586370945922 | -2.15806793464873 |
| C | -11.95184302326342 | -6.90227834354441 | -1.46742103827099 |
| C | -12.33134027827649 | -6.00260300254617 | -0.46069992509285 |
| C | -11.52367536763324 | -4.95103677683382 | -0.12394922209700 |
| C | -10.28865238735347 | -4.74441186633117 | -0.77535464949265 |
| H | -11.84819404208033 | -4.25757791711571 | 0.63617885852423 |
| H | -13.27569117424066 | -6.13892572551857 | 0.04552938288433 |
| H | -12.59420285044631 | -7.73639784944851 | -1.70816381456551 |
| H | -10.50690035181533 | -7.35317005925265 | -2.96694206027686 |
| H | -8.61815155265397 | -5.93719956976108 | -3.50216667709313 |
| C | -7.05304102995827 | -3.78670370789832 | -3.41810625007918 |
| C | -7.49705113240184 | -3.69130416121056 | -4.73794218594371 |
| C | -6.64165318228398 | -3.31046926385396 | -5.75331114600374 |
| C | -5.31457420829447 | -3.06229252896464 | -5.44310046454629 |
| C | -4.85074586119169 | -3.17785913394899 | -4.12175818866462 |
| C | -5.72110071065010 | -3.51354632480836 | -3.10471642500568 |
| H | -5.38819495486192 | -3.58088310436768 | -2.08092433751122 |
| C | -3.36072848099444 | -2.92370936375567 | -4.07127628296280 |
| C | -3.05704509530500 | -2.54937554318395 | -5.50064539768114 |
| C | -1.85655905798994 | -2.16403321801267 | -6.06135822485904 |
| C | -1.81483558276120 | -1.87738854116107 | -7.41948334203036 |
| C | -2.95374115744416 | -1.98071980023964 | -8.20393572194225 |
| C | -4.16229787710985 | -2.37147393210971 | -7.64961987258459 |
| C | -4.20832639727340 | -2.65479159498947 | -6.29398622775694 |
| H | -5.04933376685635 | -2.45209472577175 | -8.26002371990369 |
| H | -2.89729410494237 | -1.75455978966934 | -9.25874168431770 |
| H | -0.88174517387773 | -1.57090024197469 | -7.86877776098516 |
| H | -0.95854668052840 | -2.07875211949251 | -5.46520821671565 |
| C | -2.92231853851925 | -1.87210386046059 | -3.04045276351909 |
| H | -1.98434846635108 | -1.42570965828720 | -3.37763297824230 |
| H | -3.65544366532528 | -1.07381064476579 | -2.92922450992887 |
| C | -2.70979395008085 | -2.63316337801713 | -1.72736725786560 |
| H | -3.56923269842706 | -2.49030173142342 | -1.07424311945916 |
| H | -1.84183990550786 | -2.24962743719079 | -1.19197946644149 |
| C | -2.53426364255908 | -4.11658282926990 | -2.10142203616827 |
| H | -3.33675549437145 | -4.71631403691261 | -1.67391324418465 |
| C | -2.58779804006190 | -4.18404494702767 | -3.62961645732934 |
| H | -1.58382264130667 | -4.14861574696141 | -4.05463666131782 |
| H | -3.07283965830263 | -5.09219198514463 | -3.98684705446729 |
| H | -1.59388843805032 | -4.51638307500272 | -1.72343083593782 |

| | | | |
|---|---|---|---|
| H | -7.00566643762336 | -3.21337852644270 | -6.76472609043586 |
| H | -8.53807007902637 | -3.88313805395115 | -4.94858987156844 |
| O | -7.52185326888332 | -2.31136054313872 | -0.99954466209885 |

548

P-2 ($r^2$SCAN-3c/xTB)

| | | | |
|---|---|---|---|
| P | 8.22935106598445 | 0.92477882065677 | 0.81536601810130 |
| O | 9.57262148310043 | 0.50245609792842 | 0.08554719532581 |
| O | 8.74751762945797 | 1.92547733586836 | 1.99748076848994 |
| C | 10.78954923108597 | 0.62712627329754 | 0.75044417719392 |
| C | 11.50957797823026 | -0.53869149558136 | 1.10275784804836 |
| C | 12.77430229645230 | -0.39253151308294 | 1.62363736623060 |
| C | 13.33764357227773 | 0.86992045270151 | 1.87912624429299 |
| C | 14.63228252799867 | 1.00057977747352 | 2.42742285605894 |
| C | 15.13247766253327 | 2.22858177117695 | 2.75635346991861 |
| C | 14.34446233434942 | 3.37498414470997 | 2.57288283416480 |
| C | 13.09318696201592 | 3.28172763061491 | 2.02783213002969 |
| C | 12.56514566063353 | 2.03390025270274 | 1.62839954238818 |
| C | 11.28554881739245 | 1.88685885387189 | 1.01907331226445 |
| C | 10.43861774895707 | 3.07606475204159 | 0.78721398233821 |
| C | 10.86977116098888 | 4.17823327242731 | 0.00117617419833 |
| C | 12.03826013525702 | 4.14148796508705 | -0.79075223484518 |
| C | 12.40249504056773 | 5.21827187201293 | -1.55227807572240 |
| C | 11.62975054653325 | 6.38987592601789 | -1.54407787147308 |
| C | 10.48766428438419 | 6.45184373318615 | -0.79566921052257 |
| C | 10.06417459201751 | 5.34555053390051 | -0.02762744814174 |
| C | 8.87941167542928 | 5.38392516066577 | 0.72926286036135 |
| C | 8.42274207342453 | 4.29690019060029 | 1.44181108481239 |
| C | 9.21377309255533 | 3.11801970667744 | 1.42013666712930 |
| H | 13.35032412298768 | -1.27511916074007 | 1.86379850463738 |
| H | 15.21424108495464 | 0.10482589054001 | 2.59016208421532 |
| H | 16.12333660340331 | 2.32350301843366 | 3.17498254050264 |
| H | 14.73153258796059 | 4.33832191694012 | 2.87045587288556 |
| H | 12.49497360318158 | 4.17157014747375 | 1.90859224247205 |
| H | 12.64247823841539 | 3.24745442119521 | -0.79350184146188 |
| H | 13.29363438251741 | 5.17511232944259 | -2.16166618991583 |
| H | 11.94557681447482 | 7.23811711645404 | -2.13376434803569 |
| H | 9.88217833857538 | 7.34633492170398 | -0.78346327492163 |
| H | 8.31683778845342 | 6.30646178081051 | 0.74498212822168 |
| N | 7.37086453939767 | 1.84903802987759 | -0.15834572974394 |
| P | 5.86654903571765 | 1.54778765431403 | -0.57316205962094 |
| O | 5.82088458618073 | 1.78180957223157 | -2.17973023181776 |
| O | 4.91590590969852 | 2.67525913115695 | 0.01536511109111 |
| C | 6.36607575818408 | 3.04034933232335 | -2.47011622999868 |
| C | 7.70131806417781 | 3.16146929825772 | -2.92233035259102 |
| C | 8.21708583610634 | 4.42949083861368 | -3.06626499595081 |
| C | 7.46374858443576 | 5.58158132595516 | -2.77564019828149 |
| C | 8.01064489258094 | 6.87548611035423 | -2.91619803798489 |
| C | 7.27195126449734 | 7.98600786604848 | -2.61701114044886 |
| C | 5.94923411339989 | 7.84963307456222 | -2.16865093407883 |
| C | 5.38187651348518 | 6.61040367609732 | -2.04900037682846 |

| | | | |
|---|---|---|---|
| C | 6.11459633974325 | 5.44455070675317 | -2.35754535390909 |
| C | 5.57265123165233 | 4.13599574849174 | -2.22832475168336 |
| C | 4.15770639676746 | 3.92907820547766 | -1.84533756126651 |
| C | 3.13168717317292 | 4.42557587043023 | -2.69408933264833 |
| C | 3.38220317794754 | 4.98828822544873 | -3.96327609417106 |
| C | 2.34821666256871 | 5.40792304058070 | -4.75501901892197 |
| C | 1.01977859420912 | 5.28988749879260 | -4.31864370094857 |
| C | 0.74711385187307 | 4.74475949122630 | -3.09595821032705 |
| C | 1.79176681943957 | 4.29485214606404 | -2.26051457739917 |
| C | 1.53597848344839 | 3.73553911323826 | -0.99904205196155 |
| C | 2.51025330299234 | 3.18811290892766 | -0.18727374753693 |
| C | 3.84914689923591 | 3.23834909210663 | -0.69069431190950 |
| H | 9.22939446628145 | 4.55416333603483 | -3.42386841762796 |
| H | 9.02910809689839 | 6.96596752443580 | -3.26444639469227 |
| H | 7.69811656977709 | 8.97285613080913 | -2.72361747074087 |
| H | 5.37652801093407 | 8.73254929062831 | -1.92451445146625 |
| H | 4.36053781113316 | 6.51260544868016 | -1.71628465034409 |
| H | 4.39973281923308 | 5.06668736499249 | -4.31524078189960 |
| H | 2.55174761810447 | 5.82860320334906 | -5.72881263633289 |
| H | 0.21770881213057 | 5.62669428108804 | -4.95816058006943 |
| H | -0.27139305961265 | 4.63146199272112 | -2.75638987387579 |
| H | 0.50847816191945 | 3.74960053551148 | -0.67790345353400 |
| N | 7.62472961508076 | -0.37693385892262 | 1.56149461057032 |
| N | 5.19670708311958 | 0.09311833007160 | -0.33759330007941 |
| S | 6.31768917635729 | -0.05492220917817 | 2.33927760908092 |
| O | 5.82802752741939 | 1.28650286953820 | 2.19453294455731 |
| O | 5.35597523012735 | -1.11385898590822 | 2.43214004899226 |
| S | 6.09769005235907 | -1.08379624385905 | -0.80364476676009 |
| O | 7.45505693890225 | -0.78558970388370 | -1.15818554777901 |
| O | 5.79478505885206 | -2.33943808974641 | -0.17390508978592 |
| C | 5.33935705726778 | -1.49442729189157 | -2.53807962122401 |
| C | 6.97139179230837 | -0.06756604662588 | 4.15536215357061 |
| F | 4.09700722813292 | -1.99945874917325 | -2.38744746443161 |
| F | 5.23286778562934 | -0.49461221590291 | -3.41298241249288 |
| F | 6.03880587549444 | -2.45455662727916 | -3.16466015839842 |
| F | 7.82549559190414 | 0.91713704157027 | 4.45870706773867 |
| F | 7.62059866894206 | -1.21030859767285 | 4.43013616985193 |
| F | 5.96038074178670 | 0.02692537907307 | 5.04020433880387 |
| C | 1.78205691770931 | 4.65893625448119 | 5.94230370337563 |
| C | 1.06770223079312 | 5.51669396337702 | 5.12060087838500 |
| C | 1.65302432530118 | 6.07000115316089 | 3.98846914023837 |
| C | 2.95476893600950 | 5.73627019528407 | 3.68199552338158 |
| C | 3.67360815848638 | 4.85768678817211 | 4.50452024052535 |
| C | 3.09436241486267 | 4.32695675956054 | 5.64542404429864 |
| H | 1.30804720256497 | 4.23937922141887 | 6.81829416090853 |
| H | 0.04395414467885 | 5.76125907581484 | 5.36625521255786 |
| H | 1.08764135495035 | 6.74200103221331 | 3.35839804713459 |
| H | 3.64842570834521 | 3.65523579959136 | 6.28510255507526 |
| C | 3.80510027090639 | 6.19794041914020 | 2.52397381897406 |
| C | 5.02783627611292 | 5.31893799184864 | 2.67655883914139 |

| | | | |
|---|---|---|---|
| C | 6.12105612927341 | 5.18843800093209 | 1.84920080046257 |
| C | 7.20608449444857 | 4.40335429610611 | 2.26027719830421 |
| C | 7.16879363132703 | 3.79015058322504 | 3.51231667872742 |
| C | 6.05132204246202 | 3.87129908473037 | 4.32226713957544 |
| C | 4.97322067614955 | 4.62712831759440 | 3.89783423246150 |
| H | 6.14302465476251 | 5.66930995599433 | 0.88044630788799 |
| H | 8.03198192547641 | 3.24299775335677 | 3.85465265424409 |
| H | 6.03269625511402 | 3.35663598022794 | 5.27290280037090 |
| C | 3.09718873461248 | 6.12992412344586 | 1.15650751326580 |
| H | 3.74853648121495 | 5.62736453861501 | 0.44334973031959 |
| H | 2.17013264430206 | 5.56220611477237 | 1.22007259562190 |
| C | 4.20135225536202 | 7.69458082846173 | 2.69161087954811 |
| H | 5.20314817069890 | 7.79728713431413 | 3.10793970995836 |
| H | 3.49953117650383 | 8.17981285001554 | 3.37262801387527 |
| C | 2.86452666528216 | 7.57844547990370 | 0.72506881419369 |
| H | 1.95283055916857 | 7.96794679248507 | 1.18164671547852 |
| H | 2.77360802862215 | 7.67932977199341 | -0.35624756514035 |
| C | 4.07577889044073 | 8.30469772812742 | 1.29814310298350 |
| H | 3.94908332118668 | 9.38647366600321 | 1.33160855033714 |
| H | 4.96184857940825 | 8.07374260018104 | 0.70476325018583 |
| C | 7.92369119884176 | 1.03763765306611 | -4.17091406737012 |
| C | 8.50010406782189 | 2.00277358429990 | -3.34568462839531 |
| C | 9.86591291365895 | 1.93931772469907 | -3.05649154903595 |
| C | 10.63400898202912 | 0.95572883144624 | -3.63922080796686 |
| C | 10.05037175168206 | 0.01636124552014 | -4.50158850291316 |
| C | 8.68676039733479 | 0.03831550846824 | -4.74590301561314 |
| H | 6.86904628101618 | 1.09709258244340 | -4.38990334434673 |
| H | 10.29964737543344 | 2.66769350750977 | -2.38572464885999 |
| H | 8.22648203860975 | -0.69424207857622 | -5.39337088139061 |
| C | 12.12524833505136 | 0.72983731840864 | -3.53910368345723 |
| C | 12.32926276795833 | -0.42245402225791 | -4.49536021034296 |
| C | 13.49416065111023 | -1.05995802893565 | -4.86891970594641 |
| C | 13.42911860320723 | -2.10264798531795 | -5.78532726740867 |
| C | 12.21631199684851 | -2.50072852073519 | -6.32531482868002 |
| C | 11.03936530726409 | -1.86830242588889 | -5.95639223469514 |
| C | 11.10048901474329 | -0.83351098421227 | -5.03707734511942 |
| H | 14.45005312863793 | -0.76075152337309 | -4.46794410523783 |
| H | 14.33725315038793 | -2.60608526851337 | -6.08435460635406 |
| H | 12.18988635734396 | -3.30909559225842 | -7.04238904821004 |
| H | 10.09288644826582 | -2.17458889920433 | -6.37734941560295 |
| C | 12.95571084377958 | 1.97790720379275 | -3.89268317979004 |
| H | 13.09610897000273 | 2.06719086300073 | -4.97017548489662 |
| H | 12.41996844175867 | 2.86254719900456 | -3.54647023093916 |
| C | 12.58469096630693 | 0.40413998939206 | -2.10787319795609 |
| H | 12.43223380996120 | -0.64676251058414 | -1.86232183718192 |
| H | 11.98969164309259 | 0.99702113574763 | -1.41641448813499 |
| C | 14.28888950861065 | 1.83963593863505 | -3.14460410691925 |
| H | 14.60136120801656 | 2.80005048030529 | -2.73468347178966 |
| H | 15.07455893567636 | 1.50572886315800 | -3.82244913361993 |
| C | 14.06100417861926 | 0.80380089530173 | -2.02837312710533 |

| | | | |
|---|---|---|---|
| H | 14.29929531570492 | 1.20775162527301 | -1.04326271503189 |
| H | 14.69634315791614 | -0.06907686661282 | -2.18016031202114 |
| C | 0.48010438303028 | 0.67047649038524 | 7.20092699486783 |
| C | -0.89158454788092 | 0.65486454631763 | 7.00034337764813 |
| C | -1.44032279755492 | 1.02752327641048 | 5.77880848097740 |
| C | -0.59786753414359 | 1.41487459818447 | 4.75680153683353 |
| C | 0.79265313671425 | 1.40593193207873 | 4.95534052138460 |
| C | 1.33686887802855 | 1.04247007344085 | 6.17644882123522 |
| H | 0.88478529957299 | 0.38168219200313 | 8.16040663922472 |
| H | -1.54393111788849 | 0.34968344662373 | 7.80584845478952 |
| H | -2.51054193430272 | 1.00454961329231 | 5.64664924995583 |
| H | 2.40750210616349 | 1.03923753470981 | 6.31871993299637 |
| C | -0.92781925127980 | 1.88585179668884 | 3.35748235913156 |
| C | 0.44961408833845 | 2.06113556843382 | 2.76046888903809 |
| C | 0.78966232962135 | 2.46765224320450 | 1.49137718634931 |
| C | 2.13784388352830 | 2.68057390105955 | 1.14932980948094 |
| C | 3.09928938240191 | 2.46589882552165 | 2.13999890048606 |
| C | 2.76919601161402 | 2.00514386604545 | 3.40130985917649 |
| C | 1.43901087325970 | 1.81543901047686 | 3.72344014964071 |
| H | -0.00024620455210 | 2.61982345266969 | 0.77131113391532 |
| H | 4.13555599251889 | 2.64492194264898 | 1.93867409821938 |
| H | 3.55809229893084 | 1.81351889497987 | 4.11283100911267 |
| C | -1.88544794491786 | 0.94497881448949 | 2.60614896716926 |
| H | -1.70508785010511 | 1.01044362771941 | 1.53213561442666 |
| H | -1.73470221758355 | -0.09274937167119 | 2.90520562094940 |
| C | -1.69382439017874 | 3.22341680861336 | 3.36414834659401 |
| H | -1.52713711483609 | 3.72732365138304 | 2.41219026474954 |
| H | -1.34258042644197 | 3.87736186071306 | 4.16183586723753 |
| C | -3.30859233813976 | 1.43520078203015 | 2.92336942688829 |
| H | -3.79816913968511 | 0.77084961625210 | 3.63708052069324 |
| H | -3.91417313989110 | 1.44402681381681 | 2.01579505951971 |
| C | -3.16776731037278 | 2.84932999986638 | 3.51329094752283 |
| H | -3.44624529153336 | 2.85583889926041 | 4.56644176796705 |
| H | -3.80821358484196 | 3.56223382259248 | 2.99653101927032 |
| C | 11.00472503749376 | -2.70877256654243 | 2.12395562293111 |
| C | 10.95421521530127 | -1.89895487599990 | 0.98664901431521 |
| C | 10.47355073965480 | -2.41751024786324 | -0.21478179037320 |
| C | 10.10349774411538 | -3.74477737640513 | -0.26262025252335 |
| C | 10.15982711809969 | -4.55132563116124 | 0.88278605915526 |
| C | 10.59610031335867 | -4.02874005989989 | 2.08916584193580 |
| H | 11.35750109873116 | -2.28286365815524 | 3.05318250564078 |
| H | 10.40124409923166 | -1.78509713166389 | -1.08673163517854 |
| H | 10.62910283548304 | -4.63609507529657 | 2.98237164213567 |
| C | 9.64730910700343 | -4.54705636751676 | -1.45474429070071 |
| C | 9.35974840928135 | -5.88905146244587 | -0.82922173576379 |
| C | 8.86110072344231 | -7.03310191898345 | -1.41755927304491 |
| C | 8.70143733880516 | -8.17570010696245 | -0.64199976087759 |
| C | 9.04877343913323 | -8.17451395770030 | 0.70013101146673 |
| C | 9.55029714972380 | -7.02930730425656 | 1.30006067990186 |
| C | 9.69507992691120 | -5.88389505493288 | 0.53361024386346 |

| | | | |
|---|---|---|---|
| H | 8.59687096804478 | -7.04765332645167 | -2.46516405821575 |
| H | 8.30973717293017 | -9.07653480646457 | -1.09188169560611 |
| H | 8.92578097462033 | -9.07395911494244 | 1.28500085709059 |
| H | 9.82211835187618 | -7.03450912823499 | 2.34484228965048 |
| C | 8.43069429429907 | -3.93818375815831 | -2.18592490053949 |
| H | 7.63091948480053 | -4.67734486757762 | -2.24789817913178 |
| H | 8.04584497469008 | -3.07459361529422 | -1.64701911929488 |
| C | 10.75789378086498 | -4.65394707202810 | -2.53276838392898 |
| H | 11.34077737372973 | -5.56836071137020 | -2.41807450312542 |
| H | 11.43475069764212 | -3.80296497330396 | -2.44033889184425 |
| C | 8.92103185245066 | -3.56698613343207 | -3.58710662194934 |
| H | 9.33410917618511 | -2.55904387712552 | -3.57419872571264 |
| H | 8.11745391907024 | -3.59905946570619 | -4.32113908739498 |
| C | 10.02744145038029 | -4.57802065489251 | -3.86899398775097 |
| H | 10.68823043221880 | -4.26760369361235 | -4.67704092913200 |
| H | 9.59740246586514 | -5.54811657325233 | -4.12353487828351 |
| H | 0.05555134103135 | -0.87674705299573 | -1.16588419212338 |
| H | 3.31185832692402 | 0.60473064566461 | -1.84791294409437 |
| H | 3.90858065555712 | 1.63990915452544 | -3.97350596005940 |
| C | 2.52239412021546 | 0.84989722514848 | -2.55423836408102 |
| C | 2.86016815687831 | 1.44889963278891 | -3.76777598502014 |
| C | 0.77690192861451 | -0.07698792194683 | -0.94581919468051 |
| C | 1.19127176796069 | 0.56901527533189 | -2.24581035550444 |
| C | 1.87241332713799 | 1.77654016492704 | -4.68867522590184 |
| H | 2.14331188418670 | 2.23796691240082 | -5.63180131257175 |
| C | 0.20307930451377 | 0.91513130139732 | -3.17753675426651 |
| C | 0.53826134040673 | 1.51143171294699 | -4.38697147971406 |
| H | 0.20218384959305 | 0.65191443507323 | -0.36028771110795 |
| H | 2.59004344789461 | 0.13884551351943 | 0.25976840969465 |
| C | 1.88369049613430 | -0.63913872686665 | -0.05387756787126 |
| H | 2.51518436084641 | -1.35063073526769 | -0.60361142397842 |
| H | -0.83839279287440 | 0.72096796539016 | -2.92832233374542 |
| H | -0.23841109147970 | 1.76597792148675 | -5.09677504435444 |
| C | 1.28144791469253 | -1.28325131411378 | 1.19154192286653 |
| H | 0.73733345445154 | -0.50194634838633 | 1.73819158832047 |
| H | 1.73576313175148 | -2.04893579318813 | 3.11475423094328 |
| H | 0.52591814873142 | -2.02645773190265 | 0.90487353112692 |
| H | -0.31654730512893 | -5.68361392217491 | 3.64748918823714 |
| C | 2.27616987683608 | -1.88282423942479 | 2.18087703657702 |
| H | -2.01600151981098 | -3.63806587406501 | 6.32163748607880 |
| H | 3.07641099163833 | -1.16512556717936 | 2.39749211467001 |
| H | -0.53833138588034 | -6.16029364801576 | 5.33852330983431 |
| C | -0.77691790089124 | -5.39146773847374 | 4.59699874580493 |
| H | -0.60076557121551 | -2.66796874561766 | 6.76456338204654 |
| C | -0.92233597135588 | -3.65617476609603 | 6.41475528598574 |
| H | 2.45881219849750 | -2.29695654754055 | 6.84297788990950 |
| H | -1.86526078153327 | -5.38326693866518 | 4.45120647538181 |
| C | 3.00932332436275 | -3.19783340002660 | 1.84382888547049 |
| C | -0.29322581364415 | -4.00136015307422 | 5.05017885131041 |
| H | 1.50411595565255 | -1.50243451814573 | 5.58078373294579 |

| | | | |
|---|---|---|---|
| C | 2.22817079166756 | -2.29895505879132 | 5.77321418403327 |
| H | -0.66325741342479 | -4.39388044689358 | 7.18075329413193 |
| Si | 1.61264637178402 | -3.97277708444030 | 5.21899735477344 |
| H | 3.96402653969739 | -2.97338217460316 | 1.35250444198727 |
| O | 2.15107478834309 | -4.38870233591984 | 3.65045411819441 |
| N | 3.34857910602900 | -3.78972381551173 | 3.16302410327502 |
| H | -0.44779877208933 | -1.93715171554130 | 4.29618083390295 |
| C | -0.75856038612688 | -2.95368881239148 | 4.02339814698300 |
| H | 3.14286264306931 | -2.04108534409785 | 5.23282642880452 |
| H | 5.61443508859263 | -7.98948127197741 | 5.65276632965380 |
| H | -1.85502263914074 | -2.96163408376737 | 3.95390304876461 |
| H | 5.39378443869427 | -6.33611399203643 | 6.25029559472199 |
| O | 4.24591135211100 | -4.88741064453601 | 2.97663234919803 |
| H | -0.35593819635086 | -3.16425221247571 | 3.02719186527614 |
| C | 5.32977814509658 | -6.97029924639721 | 5.36044283871259 |
| H | 6.61679700944133 | -3.39802915421725 | 5.64373690872194 |
| C | 2.16563007239193 | -5.31762598328030 | 6.41098323678777 |
| C | 5.64481036976535 | -3.51573664003733 | 5.15422061182969 |
| Si | 5.78889513101565 | -4.67649959104284 | 3.69793477108564 |
| H | 4.89832596009312 | -3.79340984548040 | 5.90151042634734 |
| H | 4.28880516228951 | -6.98537166664860 | 5.02488296626326 |
| H | 1.93380260315151 | -6.31533788398690 | 6.02495714424456 |
| H | 1.68459647353465 | -5.20331800506138 | 7.38887543474918 |
| H | 3.24789654056998 | -5.26766722409726 | 6.56573011714216 |
| C | 6.26130460267759 | -6.46273895085984 | 4.24722769165242 |
| H | 7.79736988199544 | -5.76923013078528 | 5.65423291965115 |
| C | 7.70896892131922 | -6.42196075884995 | 4.77831478506811 |
| H | 5.37599113282173 | -2.54100526615459 | 4.73257702496953 |
| H | 8.02817622875005 | -7.43038861820640 | 5.07338828084553 |
| C | 7.04119344067791 | -4.00707774047854 | 2.50120054818813 |
| C | 6.18994044380072 | -7.44339732583791 | 3.06259735513751 |
| H | 6.51928474175851 | -8.44102642759801 | 3.38255251248632 |
| H | 7.85668109311661 | -3.51838377549352 | 3.04201203219054 |
| H | 6.57303692228773 | -3.23273473809730 | 1.88386237694953 |
| H | 8.40023205370493 | -6.04978558846730 | 4.01531459893626 |
| H | 6.83073762522880 | -7.12743513540834 | 2.23210228487719 |
| H | 7.46768047141126 | -4.75662454327813 | 1.83188952542998 |
| H | 5.16334636474325 | -7.52733374319544 | 2.69193860539075 |
| H | 5.19280670170448 | -5.87013816257494 | 0.82301611631323 |
| H | 2.46377470246533 | -7.24518108244133 | 0.77404338678681 |
| C | 5.54934591515687 | -5.65976064301472 | -0.18608357215328 |
| H | 6.42678952647297 | -6.28293608135247 | -0.38639398636463 |
| H | 5.85379548780860 | -4.60513290087251 | -0.20712640356639 |
| C | 3.01913385109991 | -7.95815291104504 | 0.15689834404609 |
| H | 2.47044282122716 | -8.90845010487320 | 0.17240193941749 |
| H | 3.98946439596115 | -8.12012680120828 | 0.63626230491140 |
| H | 4.03085901527174 | -8.35863425229972 | -3.13806773436307 |
| H | 0.10639038136657 | -2.88543436264627 | -2.56423136331507 |
| Si | 4.22686672845249 | -5.88195975345641 | -1.44262138332957 |
| C | 3.96381798869163 | -8.58008358693992 | -2.06938555332355 |

| | | | |
|---|---|---|---|
| C | 3.18316440232670 | -7.48525257362936 | -1.30053095875717 |
| C | 1.07605700661292 | -3.35436506795895 | -2.41030246633077 |
| C | 2.21060875732250 | -4.28417551000342 | 1.02005731659936 |
| C | 2.19314739456841 | -4.12539879615382 | -0.44443558744296 |
| O | 1.15172690084792 | -3.53589764983124 | -0.95451789499197 |
| O | 3.07506674937467 | -4.55590822967076 | -1.25109561496793 |
| H | 4.98040504605598 | -8.70347063146191 | -1.67773146772788 |
| H | 1.89025429454705 | -2.70464523287070 | -2.73538334888579 |
| H | 1.18278427063975 | -4.31343989523839 | 1.38651485923922 |
| H | 2.71472308942381 | -5.22822071068390 | 1.25167830475264 |
| H | 3.44488680283991 | -9.53889899861112 | -1.95160717540987 |
| H | 1.13768024363861 | -4.33231620201197 | -2.89344706855281 |
| C | 1.79762052132278 | -7.31456064342584 | -1.95157582820443 |
| C | 4.78582625579748 | -5.60197763898338 | -3.19510218765111 |
| H | 1.87612695047013 | -6.98907770795403 | -2.99426642253997 |
| H | 1.26347408023385 | -8.27238547346426 | -1.94002419121924 |
| H | 5.57272650209580 | -6.31335949201931 | -3.46803681045353 |
| H | 3.96982535149707 | -5.69691727554863 | -3.91893620937287 |
| H | 1.17682075980533 | -6.58924179597594 | -1.41326022716917 |
| H | 5.20597657078041 | -4.59458923669086 | -3.27658905297696 |
| P | -8.17150956516344 | -0.91485671532662 | -1.51323336909145 |
| N | -8.64974924317221 | -0.96489741171647 | -3.03705011699902 |
| S | -10.14026525483388 | -1.30834287568612 | -3.39124818709904 |
| O | -11.14188174381493 | -0.48954199757357 | -2.76979689831171 |
| O | -10.44526646644434 | -2.70818770551726 | -3.50319898941664 |
| C | -10.11122982368222 | -0.71645498978155 | -5.18589201722460 |
| F | -9.60585200409103 | 0.51369042351583 | -5.31013011583267 |
| F | -9.38683327462139 | -1.51624293387878 | -5.97319691489047 |
| F | -11.35095165172923 | -0.68500048680243 | -5.69644364237322 |
| N | -7.04227923174889 | 0.20681060529790 | -1.34923152809186 |
| P | -6.62029125821703 | 0.94547608326211 | -0.02199698918122 |
| N | -5.48835535686147 | 2.05953476162352 | -0.38575801105313 |
| S | -4.20605422645237 | 1.56580553162849 | -1.05542149851283 |
| O | -3.44132985714749 | 0.53087832600024 | -0.46239814863560 |
| O | -4.21251055840314 | 1.43093498812096 | -2.54584361079934 |
| Si | -5.15013641483067 | 1.85868515466015 | -3.95269956709656 |
| C | -6.28043635661179 | 3.26678044066711 | -3.39942229946672 |
| H | -6.98971834561271 | 3.54404483425620 | -4.17357543539976 |
| H | -5.73522113444728 | 4.16433434585845 | -3.11679218500731 |
| H | -6.84523523216045 | 2.93611554724933 | -2.53028872493214 |
| C | -6.00583090024319 | 0.27573114546325 | -4.52214975255538 |
| H | -5.30847405858270 | -0.54513406891225 | -4.66279352062917 |
| H | -6.75246608697325 | -0.04659863676668 | -3.79923685309381 |
| H | -6.51845241499824 | 0.43429660411761 | -5.46635059415509 |
| C | -3.79581665215687 | 2.42531742644054 | -5.22279310304258 |
| C | -2.77971260749843 | 3.40253223040038 | -4.61549558705935 |
| H | -2.07160178433203 | 3.72461504794808 | -5.37782616951243 |
| H | -2.22008683573833 | 2.92696855488353 | -3.81403875145600 |
| H | -3.27681760769744 | 4.28528614921010 | -4.22085365376637 |
| C | -3.03803743655604 | 1.21391466776779 | -5.78596700593938 |

| | | | |
|---|---|---|---|
| H | -3.71372284980239 | 0.53938850466310 | -6.30493219767238 |
| H | -2.28434222170914 | 1.54312946262831 | -6.49984127574634 |
| H | -2.54505945051324 | 0.65930461043616 | -4.99218662594838 |
| C | -4.50635119446064 | 3.12803480717843 | -6.38779591316810 |
| H | -5.24114924894831 | 2.46874725367879 | -6.84047959817917 |
| H | -5.01801054150277 | 4.02420315998067 | -6.04912300228224 |
| H | -3.78350968077403 | 3.41082543860538 | -7.15057922772147 |
| C | -3.18736136934705 | 3.14123136037718 | -0.87687008255178 |
| F | -3.21207016191479 | 3.57432362437123 | 0.37962762200590 |
| F | -3.65992294198599 | 4.11766592072080 | -1.64531881458406 |
| F | -1.90976424550436 | 2.94336721802905 | -1.20814431983773 |
| O | -7.82029480297620 | 1.69343023834192 | 0.68099592842479 |
| C | -7.41664309543202 | 2.50892984891697 | 1.75678910460270 |
| C | -7.10720488354010 | 1.90467191476890 | 2.95299598942921 |
| C | -6.59757339320970 | 2.69639550149112 | 4.02640150493071 |
| C | -6.10640406146631 | 2.14287673884215 | 5.22926325302317 |
| C | -5.65731039231024 | 2.94731155503589 | 6.24079287818133 |
| C | -5.66865857403681 | 4.34374927396010 | 6.10274335195085 |
| C | -6.08729501572877 | 4.90828814055456 | 4.93152093016527 |
| C | -6.53747687177025 | 4.10513747506954 | 3.86060883106726 |
| C | -6.89851533216384 | 4.67249309071398 | 2.62484223596119 |
| C | -7.29807820325606 | 3.90210289878659 | 1.55571014597237 |
| C | -7.55283113565175 | 4.51864419539921 | 0.24709492643299 |
| C | -6.65423928044355 | 5.48454362259603 | -0.21258884576312 |
| C | -6.82649190367477 | 6.09979882528900 | -1.43749510584040 |
| C | -7.90406987087529 | 5.72270285180085 | -2.22333160019704 |
| C | -8.81195085034046 | 4.75311043920036 | -1.77188672579095 |
| C | -8.65471693133993 | 4.16483672474632 | -0.53513376457631 |
| H | -9.36657186806463 | 3.43952408046530 | -0.16961718324999 |
| C | -9.86840288791827 | 4.48899978704126 | -2.81528616634272 |
| C | -9.40288467397748 | 5.36639909995502 | -3.95493408363484 |
| C | -9.91239866093257 | 5.50260992918306 | -5.23013090963123 |
| C | -9.30933234064044 | 6.39695272887934 | -6.10595636219317 |
| C | -8.20831016676793 | 7.14476475620925 | -5.71793214628674 |
| C | -7.67590165310426 | 7.00315265258538 | -4.44694992943199 |
| C | -8.27547746290576 | 6.11161149975317 | -3.57212655746657 |
| H | -6.81039897431180 | 7.57439432894305 | -4.14557148284761 |
| H | -7.75880254407540 | 7.83622198385481 | -6.41576735158541 |
| H | -9.70482874314182 | 6.50961915596109 | -7.10513947680597 |
| H | -10.75835535773147 | 4.92124453715132 | -5.56062587062065 |
| C | -10.02064545466970 | 3.00394350886013 | -3.19603056737243 |
| H | -9.31156931683246 | 2.71287081955818 | -3.96936537798037 |
| H | -9.82646197781676 | 2.37834632486942 | -2.32329465528036 |
| C | -11.47463626347809 | 2.82094194836442 | -3.65966319535358 |
| H | -11.89354861211205 | 1.92060461416011 | -3.21107054856365 |
| H | -11.52093954599820 | 2.68792994215698 | -4.74099972674107 |
| C | -12.23623705081755 | 4.08931681828685 | -3.23438859082765 |
| H | -13.16862325699413 | 3.85099922799220 | -2.72179752021817 |
| H | -12.49693100544589 | 4.69165652717677 | -4.10528209150459 |
| C | -11.28010265233139 | 4.86732726906037 | -2.32960273417218 |

| | | | |
|---|---|---|---|
| H | -11.44906536271397 | 5.94392603750466 | -2.37295566972232 |
| H | -11.38288138699165 | 4.54588404680525 | -1.29388743342735 |
| H | -6.12166175758893 | 6.84231905561759 | -1.78100843537464 |
| H | -5.79402964907081 | 5.73156680048876 | 0.39207357683123 |
| H | -6.84779966105098 | 5.74554874797276 | 2.51493830275124 |
| H | -6.07096222978991 | 5.97987920434801 | 4.79853925830346 |
| H | -5.32593615576663 | 4.96237153616113 | 6.91844271923250 |
| H | -5.28316506149186 | 2.50715256151986 | 7.15332603986985 |
| H | -6.07473206637764 | 1.07076924692154 | 5.34554181279181 |
| C | -7.26580529150137 | 0.44399155549625 | 3.11257714651119 |
| C | -6.64080416235624 | -0.42684727874660 | 2.24458167804308 |
| C | -6.58741665820619 | -1.81812300625388 | 2.50385981986612 |
| C | -7.27498720651911 | -2.31130983043243 | 3.58808576649958 |
| C | -8.00655471458876 | -1.47577942663078 | 4.44916317156580 |
| C | -8.68034853324049 | -1.99152859408938 | 5.57747148740725 |
| C | -9.38222101364483 | -1.16772678145014 | 6.41128314563864 |
| C | -9.43712748612463 | 0.20912702182554 | 6.14922510308420 |
| C | -8.75773488349309 | 0.74172149282328 | 5.08803578368643 |
| C | -8.00765830970749 | -0.07775559368832 | 4.21535975714426 |
| H | -8.78656554103747 | 1.80560223566309 | 4.91461069055379 |
| H | -10.01126170673641 | 0.85311444486729 | 6.79909415375285 |
| H | -9.89693381238689 | -1.56784137473221 | 7.27195103474399 |
| H | -8.62798707726011 | -3.05359027490472 | 5.76366799770013 |
| H | -7.21796778795250 | -3.36582934292073 | 3.81565998960908 |
| C | -5.64991858868383 | -2.72342969270300 | 1.81824141057196 |
| C | -4.30477858631488 | -2.35882778701558 | 1.75605865003038 |
| C | -3.33466381195913 | -3.28916980142586 | 1.42973285700161 |
| C | -3.72115435477729 | -4.58893108863496 | 1.14885104730040 |
| C | -5.07995441333060 | -4.93976016892583 | 1.11499915715582 |
| C | -6.04193106042328 | -4.01465912421108 | 1.45541829182710 |
| H | -7.08951926047515 | -4.27414919017531 | 1.46688909660132 |
| C | -5.24670335944900 | -6.39601286841136 | 0.74364893942121 |
| C | -3.80716795359749 | -6.85182106526921 | 0.66650359685381 |
| C | -3.30843954465530 | -8.11542977806225 | 0.42725811992841 |
| C | -1.93284557172522 | -8.30566982862082 | 0.43492915987056 |
| C | -1.06837052734027 | -7.24687018543724 | 0.66836999221044 |
| C | -1.56039971930952 | -5.97117690525608 | 0.88931374685939 |
| C | -2.93181273828953 | -5.78006222863083 | 0.89149104457240 |
| H | -0.89553488181896 | -5.13947264948072 | 1.06984769370918 |
| H | -0.00396989522080 | -7.42598662711543 | 0.69340064165180 |
| H | -1.53130594919378 | -9.29362814984724 | 0.26450034121334 |
| H | -3.96989340235049 | -8.94975715080315 | 0.24810972914556 |
| C | -5.98518519555671 | -6.59390877368032 | -0.60855967258489 |
| C | -7.27779447418447 | -7.34501992954341 | -0.28746426237224 |
| H | -8.07643351504352 | -6.63654767210866 | -0.06465619016544 |
| H | -7.60862976812758 | -7.96499128669079 | -1.11958439095834 |
| C | -6.93351752813753 | -8.15826972448319 | 0.95617001714430 |
| H | -6.35753077551022 | -9.04134788725707 | 0.67664055658208 |
| H | -7.81835501740605 | -8.48644058954382 | 1.50062405573841 |
| C | -6.07311569735227 | -7.19909479203454 | 1.77577667523430 |

| | | | |
|---|---|---|---|
| H | -5.42561706933780 | -7.71676146502269 | 2.48331290969573 |
| H | -6.71281251782380 | -6.51645422366196 | 2.33698073140689 |
| H | -5.36061165838384 | -7.18747854451865 | -1.27799051273870 |
| H | -6.18608100171987 | -5.64040373779305 | -1.09432116647879 |
| H | -2.29439065286083 | -3.00496838694138 | 1.42042817998019 |
| H | -4.01341597176710 | -1.34974672123254 | 2.00288474041551 |
| O | -5.95309332389060 | 0.04480446583647 | 1.11953284534978 |
| O | -9.44024960516762 | -0.71905263746198 | -0.56970778575528 |
| C | -9.82239289306069 | -1.44618364842017 | 0.55477127501751 |
| C | -9.68613990618731 | -2.81717685604133 | 0.64688278899990 |
| C | -9.97606237733884 | -3.45703238391692 | 1.89286062778100 |
| C | -9.67916041014695 | -4.81600753368873 | 2.13440587688088 |
| C | -10.01021303586881 | -5.41314617463625 | 3.31990052607476 |
| C | -10.67970691229631 | -4.68996252815796 | 4.31769230749704 |
| C | -10.95405228699402 | -3.36447434767428 | 4.12854373888627 |
| C | -10.57959018972417 | -2.71096580859943 | 2.93503245179779 |
| C | -10.84270718994657 | -1.34540670589489 | 2.72946902599923 |
| C | -10.46386969665468 | -0.69651990820334 | 1.57843434986227 |
| C | -10.90597155857252 | 0.69556371562164 | 1.38073390549011 |
| C | -11.56701878038953 | 1.05624850987133 | 0.20574094204103 |
| C | -12.18932952267874 | 2.28738775540661 | 0.09301681353366 |
| C | -12.13307467873963 | 3.17719612973133 | 1.15396831975767 |
| C | -11.43225989165201 | 2.84058905834063 | 2.32132249196555 |
| C | -10.83335029490938 | 1.60667176367033 | 2.43866110431502 |
| H | -10.31754759728628 | 1.32609590607427 | 3.34350931309451 |
| C | -11.52712397972478 | 3.94843138166039 | 3.34483995144378 |
| C | -12.34911696331755 | 4.97658159151628 | 2.59908845815333 |
| C | -12.75114443330366 | 6.23248118218328 | 3.00390863172028 |
| C | -13.53449679475166 | 6.99789694567429 | 2.14939813679204 |
| C | -13.91164340130695 | 6.51449150822000 | 0.90633860528603 |
| C | -13.50043727238861 | 5.26100224483186 | 0.48317430856426 |
| C | -12.71156602128637 | 4.49900228576229 | 1.32983120436962 |
| H | -13.79405534780681 | 4.88745098025065 | -0.48637866162451 |
| H | -14.52851667879786 | 7.12230608164201 | 0.26019174900835 |
| H | -13.85657693525270 | 7.98152157239489 | 2.45979291189740 |
| H | -12.46468065045178 | 6.62916471437964 | 3.96620512229840 |
| C | -10.15761595970543 | 4.49863087349338 | 3.81163003756136 |
| H | -10.00261474536574 | 5.49603570888434 | 3.39977434074943 |
| H | -9.35545242422225 | 3.86385944447978 | 3.43962881539905 |
| C | -10.17488921731330 | 4.52961811590179 | 5.34810187214192 |
| H | -9.74598882101450 | 5.45226498617104 | 5.73736884184712 |
| H | -9.58959774174252 | 3.70165734338521 | 5.75054977110428 |
| C | -11.64272922317016 | 4.36633787825692 | 5.74317369113330 |
| H | -12.14664200849325 | 5.33254707350356 | 5.75105262355953 |
| H | -11.75827910040860 | 3.92042078044315 | 6.73098636393137 |
| C | -12.21996846315257 | 3.48015328385349 | 4.64407379707128 |
| H | -13.30467755518522 | 3.55394742900609 | 4.56793929013910 |
| H | -11.95839326287231 | 2.43729315651948 | 4.83202229781486 |
| H | -12.73417615315794 | 2.52708536226377 | -0.80635451314328 |
| H | -11.62441994128387 | 0.36025736821720 | -0.61705706863308 |

| | | | |
|---|---|---|---|
| H | -11.38259270993905 | -0.80349720505349 | 3.49211043597538 |
| H | -11.45724723331634 | -2.78919386350757 | 4.89144505096179 |
| H | -10.96836785258465 | -5.18501594309115 | 5.23290993504049 |
| H | -9.76611570697578 | -6.45188797710065 | 3.48704367882888 |
| H | -9.18521334503380 | -5.39279233011933 | 1.36790502057110 |
| C | -9.38068715315259 | -3.65850146956976 | -0.53028520117581 |
| C | -8.29423354117187 | -3.40457664149368 | -1.33309561388211 |
| C | -8.00540121814570 | -4.16194215506204 | -2.48714858644225 |
| C | -8.78279804669281 | -5.26595672453496 | -2.74285350646145 |
| C | -9.91668410539651 | -5.56636819518927 | -1.96893962739407 |
| C | -10.75393322791009 | -6.65395493889686 | -2.29882622659053 |
| C | -11.92539716826733 | -6.86532309128487 | -1.62909757121233 |
| C | -12.31821907014788 | -5.97720384459012 | -0.61719355409805 |
| C | -11.51360812073256 | -4.93171729570571 | -0.25524130187955 |
| C | -10.26852287727884 | -4.72011854912552 | -0.88547433162250 |
| H | -11.84756230875365 | -4.24717904065817 | 0.50888981160162 |
| H | -13.27059910781532 | -6.11732832417571 | -0.12731051444238 |
| H | -12.56589435784157 | -7.69489883617968 | -1.88964992737474 |
| H | -10.45858087625056 | -7.30192387955001 | -3.11139173750961 |
| H | -8.55666620296600 | -5.88622170290318 | -3.59789149974776 |
| C | -6.98623706628304 | -3.74358734668772 | -3.46426297671363 |
| C | -7.40151355720383 | -3.64422937990927 | -4.79307585399208 |
| C | -6.52308530689363 | -3.26445355148403 | -5.78897679581025 |
| C | -5.20201210809773 | -3.02158851346184 | -5.45022720802664 |
| C | -4.76723497806553 | -3.13876355067255 | -4.11914207886354 |
| C | -5.66098892100072 | -3.47229111161170 | -3.12169836339039 |
| H | -5.35444847724573 | -3.53755227607855 | -2.08970345961621 |
| C | -3.27711724215286 | -2.88770184538989 | -4.03750436849522 |
| C | -2.94361163781485 | -2.50788987231452 | -5.45906734767843 |
| C | -1.73306650018360 | -2.11524258768482 | -5.99207782248079 |
| C | -1.66326656159175 | -1.82361563655972 | -7.34798519544087 |
| C | -2.78441644136984 | -1.92948932646610 | -8.15710444347894 |
| C | -4.00336321149953 | -2.32575806939501 | -7.63012592080462 |
| C | -4.07764239260620 | -2.61327547860645 | -6.27670545290174 |
| H | -4.87713715119139 | -2.40627455543788 | -8.25916310097127 |
| H | -2.70617313077573 | -1.69961566485354 | -9.20962439262003 |
| H | -0.72241607706420 | -1.51016670571596 | -7.77553845383721 |
| H | -0.84918519413080 | -2.02475831746424 | -5.37595507223187 |
| C | -2.85490322287717 | -1.84338286224342 | -2.99288423084642 |
| H | -1.90466091414285 | -1.40364568352269 | -3.30436902939105 |
| H | -3.58291185573795 | -1.03873574092052 | -2.89757898141379 |
| C | -2.68675169634662 | -2.61273352790346 | -1.67767785489001 |
| H | -3.55999406734128 | -2.45715802517926 | -1.04686018832308 |
| H | -1.82942695516137 | -2.24133145257789 | -1.11646124373213 |
| C | -2.52994593545805 | -4.09857830902257 | -2.05378286495845 |
| H | -3.36821786014292 | -4.67854619784026 | -1.67040674587182 |
| C | -2.51789386792983 | -4.15191370520082 | -3.58338255344676 |
| H | -1.49796510867869 | -4.10799990652454 | -3.96983266616056 |
| H | -2.98646571485246 | -5.05647447008681 | -3.96946647449081 |
| H | -1.62299503563295 | -4.53322664487197 | -1.63361281572894 |

| | | | |
|---|---|---|---|
| H | -6.86464083302943 | -3.16405714915103 | -6.80776469638776 |
| H | -8.43842395854844 | -3.83170350667581 | -5.02637954723351 |
| O | -7.49686354555005 | -2.28756715242484 | -1.04006621764308 |

32

2 (r$^2$SCAN-3c)

| | | | |
|---|---|---|---|
| C | -1.97761812763973 | 0.99326988048435 | -0.42522058055537 |
| C | -0.68409962826248 | 0.63793371769297 | -0.39746506924234 |
| H | -2.23303350322073 | 2.02735760122518 | -0.24058378914239 |
| H | -2.76514306806779 | 0.28295864932777 | -0.62454554715618 |
| O | 0.29746713229706 | 1.51561090042339 | -0.14890113132774 |
| O | -0.18202960041525 | -0.60895693809045 | -0.60577141111002 |
| C | -1.12347618953228 | -1.65219397749031 | -0.87938835479227 |
| H | -0.53310724964091 | -2.55863409690928 | -1.01578328154665 |
| H | -1.81442670919959 | -1.77868559312723 | -0.03812884428040 |
| H | -1.68857162471969 | -1.43297153032852 | -1.79249641864997 |
| Si | 1.97467111587160 | 1.25885727866353 | -0.08562687835107 |
| C | 2.38303043193992 | 0.03997797327746 | 1.27482806429479 |
| H | 2.06711599815144 | -0.96903379435475 | 0.99471541204858 |
| H | 3.46172589901092 | 0.01734454473560 | 1.46517097067608 |
| H | 1.88078491970523 | 0.30671412417567 | 2.21046158487431 |
| C | 2.59073827751341 | 0.65299859816143 | -1.74643942135926 |
| H | 2.23902127723276 | 1.29656528348146 | -2.55941212867409 |
| H | 3.68588639621063 | 0.64112669556470 | -1.77431445197089 |
| H | 2.23669001373119 | -0.36376610313979 | -1.93946607435946 |
| C | 2.61577670321283 | 2.99811254733064 | 0.31189276723025 |
| C | 2.17638993239640 | 3.98193534920277 | -0.78499391180781 |
| C | 2.05379452769443 | 3.46595913808687 | 1.66446885256992 |
| C | 4.15175020585636 | 2.97632110119678 | 0.38319515957633 |
| H | 2.58561262216343 | 3.70743097987918 | -1.76364640025418 |
| H | 1.08584612879134 | 4.02634949780764 | -0.87203370774830 |
| H | 2.53708026346982 | 4.99194363535116 | -0.54658092153586 |
| H | 2.37335742154792 | 2.81243044108234 | 2.48378352127277 |
| H | 2.41430075146946 | 4.47932047065029 | 1.88823867980532 |
| H | 0.95904212894932 | 3.49289041454246 | 1.65714936188829 |
| H | 4.52842379619548 | 3.98322623860871 | 0.60902396364895 |
| H | 4.51563499291491 | 2.30554352608336 | 1.16968886170929 |
| H | 4.59958046437256 | 2.66288381640458 | -0.56647454573065 |
